# Integration of heterogeneous molecular networks to unravel gene-regulation in *Mycobacterium tuberculosis*

**DOI:** 10.1186/s12918-014-0111-5

**Published:** 2014-09-26

**Authors:** Jesse CJ van Dam, Peter J Schaap, Vitor AP Martins dos Santos, María Suárez-Diez

**Affiliations:** Laboratory of Systems and Synthetic Biology, Wageningen University, Dreijenplein 10, 6703 HB Wageningen, The Netherlands; LifeGlimmer GmbH, Markelstrasse 38, Berlin, Germany

**Keywords:** Meta-study, Data integration, Regulatory networks, Reverse engineering, *Mycobacterium tuberculosis*, Dormancy, devR, Zinc uptake regulation, DNA repair

## Abstract

**Background:**

Different methods have been developed to infer regulatory networks from heterogeneous omics datasets and to construct co-expression networks. Each algorithm produces different networks and efforts have been devoted to automatically integrate them into consensus sets. However each separate set has an intrinsic value that is diluted and partly lost when building a consensus network. Here we present a methodology to generate co-expression networks and, instead of a consensus network, we propose an integration framework where the different networks are kept and analysed with additional tools to efficiently combine the information extracted from each network.

**Results:**

We developed a workflow to efficiently analyse information generated by different inference and prediction methods. Our methodology relies on providing the user the means to simultaneously visualise and analyse the coexisting networks generated by different algorithms, heterogeneous datasets, and a suite of analysis tools. As a show case, we have analysed the gene co-expression networks of *Mycobacterium tuberculosis* generated using over 600 expression experiments. Regarding DNA damage repair, we identified SigC as a key control element, 12 new targets for LexA, an updated LexA binding motif, and a potential mismatch repair system. We expanded the DevR regulon with 27 genes while identifying 9 targets wrongly assigned to this regulon. We discovered 10 new genes linked to zinc uptake and a new regulatory mechanism for ZuR. The use of co-expression networks to perform system level analysis allows the development of custom made methodologies. As show cases we implemented a pipeline to integrate ChIP-seq data and another method to uncover multiple regulatory layers.

**Conclusions:**

Our workflow is based on representing the multiple types of information as network representations and presenting these networks in a synchronous framework that allows their simultaneous visualization while keeping specific associations from the different networks. By simultaneously exploring these networks and metadata, we gained insights into regulatory mechanisms in *M. tuberculosis* that could not be obtained through the separate analysis of each data type.

**Electronic supplementary material:**

The online version of this article (doi:10.1186/s12918-014-0111-5) contains supplementary material, which is available to authorized users.

## Background

Current biology research generates an ever-increasing deluge of omics derived data. Each type of omics data pertains to a single level of the biological system under investigation (transcriptomics, proteomics, metabolomics, lipidomics, etc.). While detailed knowledge of the individual genes, transcripts, proteins, metabolites and other cellular components remains important, understanding a biological system requires considering the networks into which these components are embedded and of their functioning as a (dynamic) whole. A major challenge in Systems Biology lies thus on developing effective and efficient methods to optimally extract the information contained in the aggregate of these datasets.

The increasing availability of genome-scale expression data has boosted the development of methods to infer the underlying regulatory networks. A broad range of alternative methods are available, see [1–3] for reviews, and each of them uses different mathematical tools and/or biological assumptions. A class of methods use differential equations to express transcript changes as a function of the transcript levels of the corresponding transcription factors [4,5]. A second class of methods rely on Bayesian networks to analyse the joint probability distributions obtained from the experimental data [6,7]. Other methods use the similarity between gene expression profiles to detect associations and to reconstruct a genome scale transcriptional regulatory network [8–10]. Another class of methods use a combination of machine learning techniques to produce prioritized lists of transcription factors regulating each target gene [11,12]. Each method has different strengths and weaknesses, even methods using similar conceptual tools. For example C3NET [13] uses mutual information (MI) to reconstruct the core regulatory interactions in the network, that is, to recover the strongest interactions. Within this core, C3NET was shown to outperform other methods also using MI such as CLR [8] and ARANCE [9]. Each method provides different results, therefore much effort has been devoted to generate consensus networks from the multiple solutions. It has been shown that in many instances, an integrative approach combining the outcome of each algorithm produces the best result [14], however, a detailed analysis shows that for some interactions individual methods perform better than the consensus network [15].

### Co-expression networks and module identification algorithms

Co-expression networks contain genes as nodes and the edges of the network represent significant co-expression levels across the studied data set. An open problem is still how these connexions are to be defined and how an adequate threshold is to be imposed [16]. In differential network analysis two networks obtained using the same algorithm but alternative datasets are compared to identify interactions appearing only under a subset of conditions [17–19]. For example, such an approach was used to analyse prostate cancer datasets by comparing the networks inferred using C3NET from normal or tumour samples datasets and was able to successfully identify cancer specific interactions [20]. Here, we construct and analyse co-expression networks extracted from the same dataset but using alternative algorithms to identify relevant interactions.

Biclustering or module learning algorithms [21] aim at the identification of functionally related genes showing co-expression patterns. In some cases, such as the cMonkey algorithm [22], additional biological information (databases or sequence analysis) is also considered. The results of the genome-scale reconstruction methods can be displayed and visualized as one network whereas an identified module or bicluster contains a set of functionally related genes that might be differently regulated in different conditions. Therefore, the genes within a module might not form a cluster when considering all the conditions and would remain undetectable for network inference methods. Network inference methods and module prediction algorithms are highly complementary and new information can be obtained by combining the outcome of both approaches.

### Analysis of ChIP-seq data

There are experimental methods to directly reconstruct regulatory networks. The knowledge of transcriptional regulatory events and specifically the transcription factors (TF) binding sites can be greatly improved by chromatin immunoprecipitation and sequencing, ChIP-seq. Powerful techniques have been developed to process the sequencing data and to isolate the binding sites from the background noise generated by non-specific sequences, such as statistical tests, use of controls, techniques for signal de-convolution or lag-analysis among others [23]. In the classical model of transcription regulation in prokaryotes a binding site in the promoter region of a gene is linked to a regulatory interaction between the corresponding TF and the gene. However, the collection of ChIP-seq data for *Mycobacterium tuberculosis* (*Mtb*) hosted in the TB Database (TBDB) [24,25] shows that not all identified TF binding sites can be linked to regulatory interactions. For example, in some instances the TF binding is much weaker than expected, in other cases this association is complicated by the occurrence of divergons, which are pairs of divergently transcribed operons or genes, or by the presence of binding sites within coding regions. This lack of a one-to-one relationship between TF binding sites and regulatory events can be due to multiple reasons such as non-specific binding, cumulative effects of sites with weaker binding to regulate overall promoter affinity, specific binding generated in non-biological or non natural conditions, false positives as a result of the experimental procedure, or, simply errors in the genome annotation. We propose that it is through the integration with additional data sources, specifically through the integration with expression data, that this challenge can be overcome.

### Integration of heterogeneous molecular networks

Each inference algorithm has its weaknesses and strengths and each network holds its own intrinsic value and can be used to gain more specific insights on various aspects of the biological system [26]. In a sense, using multiple algorithms to extract the networks and presenting them to the user is a similar approach to that followed by annotation pipelines, such as Microscope [27], DIYA [28] or BASys [29] among others. These pipelines present the user a list of gene centred information, with different annotation sub-fields, to enable supervised annotation. Integration efforts require a common layout for the data, therefore, we have chosen to represent the available layers of information, such as operon structure, known interactions between genes or proteins, enzymatic activity (metabolic map) or functional similarity, among others through network representations. The networks themselves are represented in a common format, XGMML (eXtensible Graph Markup and Modeling Language), that allows their simultaneous exploration.

**Figure 1:**
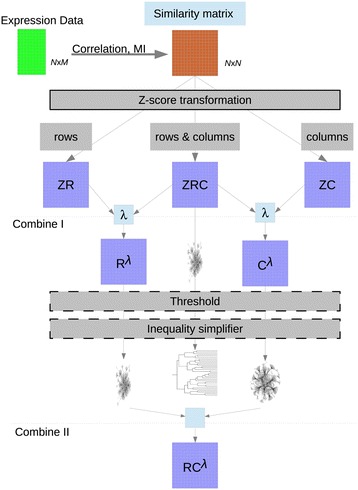
**Schematic of the pipeline to obtain co-expression networks.** From top to bottom the following steps are applied: (1) calculation of the similarity matrix, (2) z-transformation, (3) Combine I, (4) threshold setting (5) inequality simplifier and (6) combine II. Note that when applying the Inequality simplifier to the ZRC network the result will be a tree.

In addition to the simultaneous visualization of different networks, additional analysis tools such as motif search and identification, GO-enrichment analysis, and tools to overlay expression data or analyse expression profiles of multiple genes across different conditions can be used. We have developed a pipeline for the generation of co-expression networks that is easily tunable to produce alternative networks (holding their own intrinsic value). The pipeline is based on using the similarity between gene expression profiles to detect associations and contains a higher order extension of the data processing inequality method [9] to reduce the number of redundant or possible spurious links. This pipeline is presented in Figure 1.

**Figure 2:**
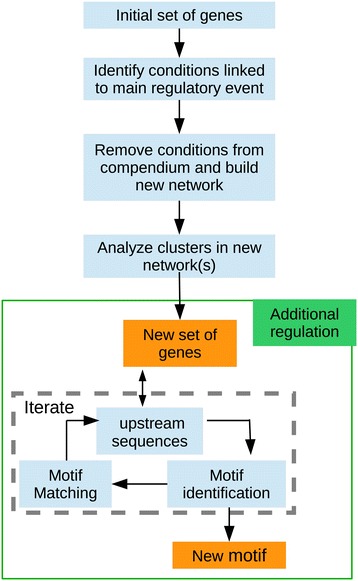
**Pipeline to uncover additional regulatory layers.** Step 1: Identify conditions linked to the main regulatory event for the initial gene set. This can be done using biclustering techniques or by direct comparison with the expression levels of the regulator (if known). Step 2: Build co-expression networks in the remaining conditions. Step 3: Identify the closest neighbours of the selected genes in the new networks. Step 4: iterative round of motif identification/matching to identify the secondary motif and the set of genes with this motif in their upstream regions.

**Figure 3:**
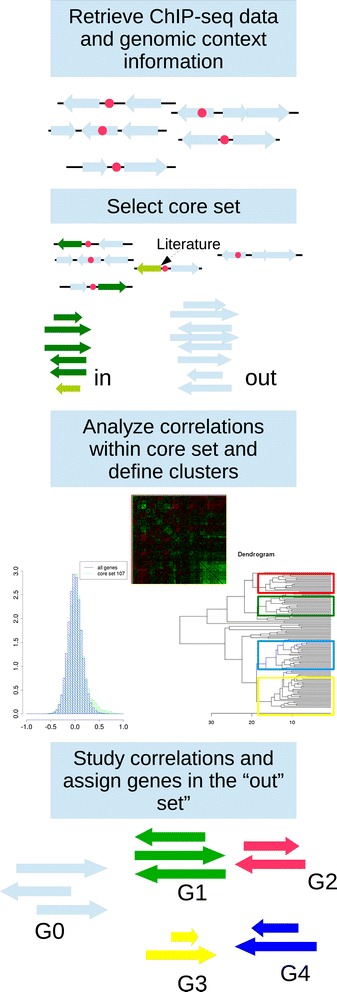
**Pipeline to analyze ChIP-seq data.** After the locations of the ChIP-seq binding sites have been retrieved, their genomic context is analysed. A core set is defined by selecting targets with i) literature evidence or ii) a hit in the upstream region of not divergently transcribed genes. The expression levels of these genes are analysed and they are categorized through (bi)clustering. Finally the rest of the putative targets are assigned to these groups (if possible) based on the similarities of their expression patterns.

Through the exploration of the multiple networks, the user gains new knowledge of the biological system, but it can also lead to the discovery of new strategies to integrate the information stored in the networks. These newly gained strategies can then be translated into new pipelines. As result of our exploration, we have developed a pipeline to uncover multiple layers of regulation, presented in Figure 2 and another one to analyse ChIP-seq data and assign regulatory interactions to the detected binding sites, presented in Figure 3.

### Show case: Deployment of the framework to unravel regulatory mechanisms in Mycobacterium tuberculosis

We have analysed regulatory events in *Mtb*. Due to its implications to human health this highly successful pathogen has been extensively studied and there is already a substantial body of information on *Mtb* and its underlying regulatory networks, but still much remains to be learned. We have analysed not only networks extracted from literature [30–32] but also networks extracted from publicly available databases such as STRING [33], MetaCyc [34], KEGG [35], TBDB [24] and Tuberculist [36]. Expression data from publicly available repositories and corresponding to 287 perturbations, have been used to generate the co-expression networks and to further analyse ChIP-seq data [25]. We have finally combined the inferred networks with the outcome of biclustering algorithms, to further explain the functionalities of the modules.

We have analysed the regulation of DNA repair systems within *Mtb*, particularly we have focussed on two alternative regulatory mechanisms: the ‘RecA-LexA dependent DNA repair system’ and ‘RecA non dependent regulation’. We will show that the integration of expression data and its use to guide the exploration of upstream sequences allows to analyse alternative regulatory mechanisms for the same set of genes. In addition, we have explored some of the regulatory mechanisms that allow *Mtb* to survive within the host, such as DevR (DosR) regulon, which is a key element for understanding the dormant state, and the regulation of the response to changes in zinc availability (ZuR regulon).

## Methods

### Gene expression data

565 two-colour microarrays for *Mtb* (strain H37Rv) were retrieved from the Gene Expression Omnibus Database [37]. 454 of them aimed to capture the effect of 75 drugs targeting metabolic pathways [38,39] whereas 111 captured stress induced dormancy in the wild-type and in DevR activation genes knockout mutants [40,41]. We followed Boshoff *et al*. [38] to classify the conditions in the compendium into 14 categories according to the experimental perturbation: (1) Aromatic amides intracellularly hydrolyzed, low pH; (2) mutants of DevR activation pathways; (3) Translation inhibition; (4) Acidified medium; (5) Cell wall synthesis inhibition; (6) Respiration inhibition (except conditions with NO); (7) Nutrient starvation; (8) DNA damage; (9) Transcription inhibition; (10) Iron scavengers; (11) Multiple stress sources applied simultaneously (low oxygen, low pH, glycerol-deprived); (12) Minimal medium (succinate/palmitate as carbon source); (13) not classified; and (14) conditions associated with DevR upregulation. A list of the used datasets is presented in Additional file 1. We applied a common normalization method, loess, to the arrays from each experiment and each of the six independently designed platforms. Linear models were constructed to consider biological and/or technical replicates (when available); within-array replicate probes were averaged; and an additional between-array quantile-quantile normalization was performed to ensure the comparability between experiments. These manipulations were performed using the R limma package [42]. A common locus tag format was introduced and missing values were filled up using knn-imputation from the impute R package [43] with *k* = 3 and eliminating genes and experiments with more than 30% and 50% missing values respectively. The resulting compendium contained information on 4223 open reading frames across 287 different conditions (175 steady state situations and 100 conditions within 30 time series).

### Biclustering

We adapted the original cMonkey R code to *Mtb* data [22] and we increased the number of biological information sources that the algorithm can consider. For the biclustering process, we selected a subset of the interactions present at the STRING database [33]. We selected specifically interactions obtained based on co-occurrence of linked proteins across different species, on curated databases, and on their association in the abstracts of scientific literature. From ProLinks [44] we collected interactions obtained by the phylogenetic profile method. In addition, we included the network based on the similarity among the annotated GO terms (for the ontologies biological process’ and cellular component’), which was constructed using the Sleipnir library [45]. Upstream sequences for *Mtb* genes were retrieved using RSAT [46] and used for the motif detection and motif matching steps. For the automatic biclustering algorithm, we used the 1000 bp region upstream of the translation start site, avoiding overlapping with upstream neighbour genes when present. In the initial rounds we used a reduced matrix where only the leaders of the operons, as defined by Roback and co-workers [47], were kept. Multiple runs of the algorithm were performed, which used the default parameters except for: initial size of seed clusters (10); number of iterations (1000); maximal number of clusters (300). We obtained a set of 1527 biclusters. The Jaccard similarity coefficient between each pair of biclusters was computed (number of shared genes between both biclusters over the number of different genes in both biclusters). To reduce the redundancy in the set of biclusters, we merged the pairs that showed a Jaccard similarity higher that 0.7. The biclusters in the merged set were enlarged by adding genes based on the predicted operon structure combined with the expression level measurements. This new set was used to seed the biclustering algorithm in two subsequent optimization rounds one biased towards the detection of concurrent motifs in the upstream regions and a second one biassed towards the identification of sets linked to highly related biological processes (GO terms). We performed an additional manual merging step, that also considered the similarity between the detected motifs using the matrix comparison tools from RSAT [46] and we obtained a final set of 76 biclusters. Expression plots for these biclusters are in Additional file 2.

### Pipeline for the generation of co-expression networks

The pipeline to generate co-expression networks is presented in Figure 1. Departing from an *NxM* matrix containing the expression profile of *N* genes in *M* conditions, we construct a symmetric *NxN* similarity matrix *S*. The pipeline allows to choose between correlation (Pearson, Kendall and Spearman) and MI as similarity measurements. MI is computed using an estimator based on the entropy of the empirical probability distribution with initial data discretization into n (default = 10) equal sized bins from the Bioconductor package minet [48].

In the second step, a z-score transformation is performed on the distribution of the scores from the similarity matrix. The transformation is done by rows, to obtain the ZR matrix; by columns, to obtain the ZC matrix; or by both rows and columns simultaneously, to obtain the ZRC matrix. The z-score transformation allows for each possible interaction to be weighted regarding the background of interactions in which each member of the interacting pair is involved [8]. Therefore, in the ZC case each element *S*_*ij*_ becomes ZC_*ij*_, which is the z-score of *S*_*ij*_ regarding the distribution *S*_*i1*_, *S*_i2_, …, *S*_*in*_. In the ZR case each element *S*_*ij*_ becomes ZR_*ij*_, which is the z-score of *S*_*ij*_ regarding the distribution *S*_*1j*_, *S*_*2j*_, …, *S*_*nj*_. In the ZRC case each element *S*_*ij*_ becomes

1 $$ \sqrt{\left(Z{C}_{ij}^2+Z{R}_{ij}^2\right)/2} $$

In the following step the matrices are combined into two new matrices: *C*^*λ*^ and *R*^*λ*^:

$$ \begin{array}{c}{C}_{ij}^{\lambda }= \max \left(Z{C}_{ij},\lambda \cdotp ZR{C}_{ij}\right)\\ {}{R}_{ij}^{\lambda }= \max \left(Z{R}_{ij},\lambda \cdotp ZR{C}_{ij}\right)\end{array} $$

with *λ* (default = 1) a positive real number and *i* and *j* denoting the rows and columns of each matrix. *λ* allows to fine-tune the results, since higher values of *λ* will lead to *C*^*λ*^ and *R*^*λ*^ matrices that are similar to each other and to ZRC. Considering *λ* = √2 means that, for those cases where one of the elements in eq. 1 is zero, both *λ* ZRC_*ij*_ and ZC_*ij*_ will be identical. However, using the default *λ* = 1 will ensure that when the values of ZC_*ij*_ and ZR_*ij*_ differ then the highest one will be selected either through ZC_*ij*_ (or ZR_*ij*_) or ZRC_*ij*_.

In the following step a threshold can be applied to remove interactions with low weight (and therefore low likelihood). Any value below the threshold is set to 0. This step produces a more sparse network, which might be needed to obtain a neat visualization. The default threshold value is chosen so that the number of non zero edges is equal to a predefined value (default = 10000).

Afterwards the inequality simplifier can be used to remove, possibly spurious, links from the network. The inequality simplifier is an extended version of the data processing inequality (DPI). In simple terms, the DPI states that given two interdependent random variables *A*_*1*_ and *A*_*2*_ and a third one *A*_*3*_ that only depends on one of them, for example *A*_*2*_, then *A*_*1*_ cannot contain more information about *A*_*3*_ than *A*_*2*_ does. This statement is mathematically represented by the following inequality among MI values

$$ M{I}_{A_1,{A}_3}\le \min \left(M{I}_{A_1,{A}_2},\ M{I}_{A_2,{A}_3}\right) $$

The DPI must hold whenever *A*_*3*_ does not depend on *A*_*1*_, so it can be used to remove spurious interactions from the network [9], possibly caused by feed forward loops or mutually dependent regulators. The spurious links are removed according to:

$$ if\kern1em M{I}_{A_1,{A}_3}\le M{I}_{A_1,{A}_2}\ \&\ M{I}_{A_1,{A}_3}\le M{I}_{A_2,{A}_3},\kern0.5em  then\kern1em  set\kern1em M{I}_{A_1,{A}_3}=0 $$

The DPI can be extended to higher-order interactions, therefore allowing a recursive implementation [49]. The DPI is derived from the triangle inequality satisfied by any metric or distance, such as the MI or Kendall rank distance. Our extension to remove spurious edges by considering higher order interactions contains two parts: the first part is the identification of alternative pathways connecting the same nodes and the second part is to apply the inequality simplifier to decide whether a link is spurious and should be removed. The first part is based on Dijkstra's shortest route algorithm [50]. Here, instead of searching for the shortest route between two nodes (e.g. genes), we use it to find the alternative path between those two nodes with the best throughput. The throughput of a path is defined as the value of the edge with the lowest throughput (similarity) value. Given two connected nodes, the link between them is considered spurious and removed if an alternative path is found with a higher throughput value. For a given pair of nodes, *A*_*i*_ and *A*_*j*_, *(i < j)*, so that the best alternative path of length (n + 1) passes through *A*_*1*_*, A*_*2*_*,* …, *A*_*n*-1_ and *A*_*n*_, the following rule (inequality simplifier) is applied:

$$ \begin{array}{c} if\ M{I}_{A_i,{A}_j}\le \min \left(M{I}_{A_i,{A}_1},M{I}_{A_1,{A}_2},\dots,\ M{I}_{A_{n-1},{A}_n},M{I}_{A_n,{A}_j}\right)\\ {} then\  set\ M{I}_{A_i,{A}_j}=0\  where\ n\in \left\{1,\dots, N-2\right\}\end{array} $$

This rule is applied for each possible pair of nodes and for each possible value of *n.* The alternative pathway joins *A*_*i*_ and *A*_*j*_ and does not contain any cross-linking, so that therms such as *A*_*2*_*A*_*4*_ or *A*_*1*_*A*_*3*_, do not need to be considered. This is a consequence of using the path with the best throughput so that no triangle inequality of the metric is required to derive this property.

**Figure 4:**
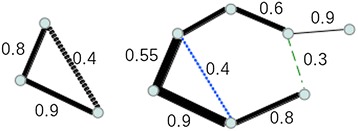
**The inequality simplifier.** The similarity values among the nodes (genes) connected by the different edges have been indicated. Dotted lines represent the spurious links removed by the inequality simplifier. *Left* application of order two, which is equivalent to a direct application of the DPI. *Right* higher order application, both dotted lines are removed. The DPI would only remove the blue dotted line.

Figure 4 shows how the inequality simplifier acts to remove, possibly spurious, links from the network when applied to higher order terms. While applying this rule, an additional *NxN* matrix, *T*, is built to keep track of how many links a particular edge has caused to be removed. In this case, the links *A*_*i*_*A*_*1*_, *A*_*1*_*A*_*2*_, *A*_*2*_*A*_*3*_, …, *A*_*n-1*_*A*_*n*_ and *A*_*n*_*A*_*k*_ have caused the removal of link *A*_*i*_*A*_*j*_, therefore, in the *T* matrix, the elements *T*_*Ai,A1*_*, T*_*A1,A2*_*,T*_*A2,A3*_*,*…*, T*_*An-1,An*_ and *T*_*An,Aj*_ are increased by 1∕(*n + 1)*.

Finally the two networks C_*ij*_ and R_*ij*_ are combined into one final network RC_*ij*_.

$$ R{C}_{ij}^{\lambda }= \max \left(Z{C}_{ij},ZR\right) $$

The presented pipeline can be used to obtain different outputs, such as the RC or the ZRC networks and their variants through the application of thresholds or the inequality simplifier. The final output is a square matrix that represents a network through a weighted adjacency matrix. This adjacency matrix is finally exported into a tabular format that can be imported into Cytoscape [51] to obtain a graphical representation. The weights in the adjacency matrix or the *T* matrix produced by the inequality simplifier can be imported as edge attributes and used to set the thickness of the edges. Finally, a force directed algorithm can be used to generate the appropriate layout and the graphical representation of the network, which then can be exported to an XGMML file.

Applying the inequality simplifier to a symmetric matrix, such as ZRC, removes any possible loop in the network, therefore it results in a forest graph as depicted in Figure 1. Applying the inequality simplifier to an initial matrix containing around 3000 nodes takes at most five minutes in a standard desktop computer (2.80GHz Intel machine).

The networks used for our exploration of gene regulation networks in *Mtb* were the result of applying the inequality simplifier to ZR, ZC and ZRC (obtained with *λ* = 1). Belcastro e*t al.* [52] have shown that from the total number of possible interactions a selection of 5% (4*10^5^ for a network with 4000 genes) would be a sensible choice according to the assumption that biological network are sparse. Here, one of our goals is to obtain a clear visualization of the network, therefore, we have chosen an even lower number (10000) so that the number of nodes included in the network is as high as possible without having a too crowded visual representation of the network. An additional threshold was imposed so the number on non zero edges of ZR, ZC and ZRC networks is equal to 10000. The ZRC derived network contained 2293 nodes and 10000 edges whereas the mixed network contained 2693 nodes and 5898 edges.

These manipulations were implemented in R and Java, the corresponding scripts can be found, together with an example data set in Additional file 3.

### Additional networks and analysis tools

#### Metabolic networks

The nodes in the metabolic networks are of two different types: reactions and metabolites, and additional information, such as the stoichiometry, directionality and the catalysing proteins of the reactions, also has to be stored. We kept this information by using a combination of two file types: a SVG (Scalable Vector Graphics) file that contains the graphical representation of the networks and an RDF (Resource Description Framework) file containing the additional information (see Additional file 4 for technical details).

#### Other networks

A network formed by multiple disjoint interlinked clusters was generated using the predicted *Mtb* operon structure [47]. In addition, the information stored in the STRING database [33] was used to generate networks based on: known protein-protein interactions; distance of the genes in the genome and that of orthologous genes in other species; co-occurrence in other species; orthologous genes being fused together in other species; association in the abstracts of scientific literature; and other databases, such as MetaCyc [34] and KEGG [35].

To identify regions duplicated in the genome or homologous genes, a network was built by considering the sequence similarity between each pair of genes. The similarity was computed using Megablast [53] and an edge was created for matches with E-values lower than of 10^−10^.

#### Sequence analysis tools

We have used MEME [54] for motif elicitation within a set of genes and FIMO [55] to find occurrences of the chosen motif. A post processing step ranks, using the FIMO q-value, the genes (matching upstream region) for each identified motif (MEME). An iterative cycle of motif identification and motif matching can be established to further refine each motif. The computational time required by each iteration greatly depends on the length of the upstream sequences, which has to be selected for each particular analysis. For the selected show cases the motifs accumulated in the 200 bp region upstream of the translation start site.

#### Gene ontology (GO) enrichment analysis

On selected set of genes was performed using a hypergeometric function to model the probability density, as implemented in the GOHyperGAll R function [56]. The GO annotation for *Mtb* was obtained from the UniProt-GOA database [57], the Mtb-GOA database (http://www.ark.in-berlin.de/Site/MTB-GOA.html), TBDB [24] and the recent re-annotation of the *Mtb* genome [58].

#### Operons

We developed a putative operon extension tool which is only based on the gene orientation, so that genes that are downstream of the selected gene (or genes) and with the same orientation are added to the putative operon until a gene with the reverse orientation is found. The user is then free to decided whether the expression data sustains the extension of the operon. This approach allows to easily combine expression information with genomic context information.

Venn diagrams have been created using the utilities from the VennDiagram R package [59].

#### Expression plots

Generated with a solid line representing the mean expression of the selected genes in the conditions included in the compendium and dots marking the conditions in the bicluster where the genes show high correlation (bicluster). The classification of the experimental conditions, previously mentioned, was included in the expression plots through an horizontal colour line that allows to quickly associate the behaviour of the genes with the type of perturbation. To select the conditions (if any) in which the selected genes show co-expression there is an initial step where all conditions are included in the bicluster, then an iterative loop starts were each condition is removed at a time and the new correlation values are computed, finally the condition leading upon removal to the highest correlation values in the remaining set, is removed from the set. This process is iterated until the correlation in the remaining set of conditions is higher or equal to 0.8.

### Discovery of additional regulatory layers

Whenever two alternative regulators regulate the expression of genes in a given set, it might be that one overshadows the detection of the other. We have developed the work flow to analyse these events (Figure 2). Given a set of genes under control of two regulatory events, the first step is to identify the conditions were the first event is active or taking place. How these conditions are identified depends on the particular example. One way would be to identify conditions with up/down regulation of the corresponding transcription factor. To identify conditions were *recA* expression is not regulated by the RecA/LexA mediated mechanism we built a model linking the time dynamics of *recA* to *lexA* expression levels using the Inferrelator algorithm [4]. The output of the algorithm is a prediction or fit of *recA* levels based on *lexA* levels (see Additional file 5). Those conditions with a poor agreement between the measured and fitted levels are the conditions (most likely) without LexA mediated induction of *recA*.

Once the new set of conditions has been selected, the following step is to reconstruct the co-expression network(s), using only the expression data corresponding to this subset of conditions and analyse the location of the original set of genes in the newly built network(s). Furthermore an iterative round of motif identification and motif matching can be run to identify a possible motif in the upstream region of the cluster. This iterative round can be performed either using cMonkey [22] (restricting the expression data to the selected conditions) or manually using alternatively MEME [54] and FIMO [55].

### Analysis of ChIP-seq data

Publicly available ChIP-seq data were obtained from TBDB [25]. We have developed the workflow, presented in Figure 3, to analyse these data. The ChIP-seq data had already been analysed with a peak calling algorithm. For each of the considered TFs a list of predicted targets was obtained from TBDB, together with information about the peak location. A reduced list, or core set, was obtained by selecting those targets that show a hit in their adjacent upstream intergenic regions. An additional filtering was done to select only those hits where the peak is not flanked by divergently transcribed genes/operons. Additionally, the genes for which literature evidence supported the regulatory interaction with the TF were included in the core set. In the following step, the expression levels of the genes in the core set were analysed and the matrix of correlations across the conditions in the expression compendium was computed. The appearance of negatively correlated genes in this set is a signal of a dual repressing/activation function of the regulator and therefore two (or more) subsets can be defined by hierarchical clustering of the set. This process can be further enlarged to encompass additional groups that would be linked to alternative regulatory mechanisms or to the effect of additional TFs. Once this/these group(s) were defined, the rest of the putative targets predicted by ChIP-seq were assigned to either one of these groups based on their average correlation with the members of the group (0.7 threshold).

### Topological overlap

The topological overlap between two genes *i*, *j* in an unweighed network is defined as [60,61]:

$$ {t}_{i,j}=\left\{\begin{array}{cc}\hfill \frac{\left|N(i)\cap N(j)\right|}{ \min \left\{\left|N(i)\right|,\left|N(j)\right|\right\}+1-{a}_{i,j}}\hfill & \hfill i\ne j\hfill \\ {}\hfill 1\hfill & \hfill i=j\hfill \end{array}\right. $$

Where *N*(x) is the set of direct neighbours of gene *x* (excluding itself); |x| represents the number of elements in set x and a_*i,j*_ is the adjacency matrix of the network (1 if there exists a link between genes *i* and *j* and 0 otherwise). The topological overlap is bounded between 0 and 1. Two genes will have high topological overlap if there exists a connection between them and if they are connected to the same group of genes. For weighted networks, a_*i,j*_ represents the weight of the interaction between genes *i* and *j* and takes continuous values between 0 and 1. In these cases, the previous formula can be generalized to [16]:

$$ {t}_{i,j}=\frac{\varSigma_k{a}_{i,k}{a}_{k,j}+{a}_{i,j}}{ \min \left\{{\varSigma}_k{a}_{i,k},{\varSigma}_k{a}_{j,k}\right\}+1-{a}_{i,j}} $$

The topological overlap of a group of genes was defined as the average of their mutual topological overlap, which was computed using the R WGCNA package [62]. Only groups with more than 5 genes were considered. An empirical p-value for these scores was calculated by randomly sampling (10000 times) gene groups of the same size in the respective network.

### Network visualization

We have developed a visualization tool that allows the simultaneous visualization of networks in XGMML format and the sharing of identifiers between them. Technical characteristics of this tool are available in Additional file 4.

## Results

### Co-expression networks

We have developed a pipeline to generate co-expression networks that allows for a myriad of possible networks. Choosing a subset of them highly depends on the available data and the process the user wants to explore, since different clusters appear in each of them. This reflects the inherent modularity of biological networks and the dependency of regulation on the chosen conditions.

The pipeline presented in Figure 1 includes some already well established and tested methods for genome-scale network inference. For example, the similarity matrix computed using either correlation or MI is commonly used to analyse gene expression data [63], but also other methods are contained in the pipeline. When working with mutual inference as a similarity measure the matrix denoted ZRC corresponds to the output of the CLR algorithm [8]. Additionally, applying the inequality simplifier only up to order two on a MI matrix amounts to using the DPI to prune spurious interactions from the networks, which is a key element of the ARACNE algorithm [9].

A comparative analysis of methods to reverse-engineer transcriptional regulatory networks was done using the results of the DREAM5 challenge. In this challenge the teams had to reconstruct genome-scale transcriptional regulatory networks from expression data. The networks proposed by the different teams, were evaluated through their comparison with a gold standard, a set of experimentally verified regulatory interactions in the target organisms. In addition, Marbach *et al.* [15] constructed a consensus network integrating the predictions from the multiple methods. Whole network performance estimators such as Area Under Receiver Operating Characteristic Curve (AUROC) or Area Under Precision Recall Curve (AUPR) show that over the entire network the consensus network outperforms individual methods due to their inherent complementarity. However, for some interactions individual methods perform better than the consensus network (see Additional file 6A). The loss of information when building the consensus network does not affect equally the different transcription factors (see Additional file 6B). For example, for PurR or LexA, a significant fraction of the total number of known interaction is better recovered by the individual methods than by the consensus network.

**Figure 5:**
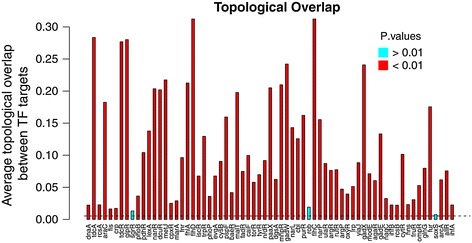
**Topological overlap of TF targets in the** ***E. coli*** **ZRC co-expression network.** The ZRC co-expression network was reconstructed using our pipeline using *E. coli* expression data from the DREAM5 challenge [15]. Only the 67 TF with more than 5 experimentally verified targets (in the gold standard) were considered. Dashed line represents the average topological overlap in this network (0.0053).

To evaluate the performance of our algorithm we have generated co-expression networks corresponding to the synthetic, *Escherichia coli* and *Saccharomyces cerevisiae* datasets used in the DREAM5 challeng. For each dataset, two networks (ZRC and mixed) were built (see Methods section). In the challenge the goal was to identify links between regulators and target genes. In co-expression networks no special emphasis is done on the regulators. To evaluate the networks we have calculated the topological overlap [60,61] of the known targets of the transcriptions factors (also provided in the challenge). The topological overlap measures how similar the neighbourhoods of two genes are [16]. For 95% (64 out of 67) of *E.coli* Tfs considered (those with more than 5 experimentally verified targets) the topological overlap of the target genes in the ZRC network is significantly higher than for the overall network (see Figure 5), which means that they form cohesive modules in the network. The relative number of cohesive clusters identified for the other datasets (yeast and synthetic set) are lower (57% and 40% respectively, see Additional file 7) Overall the ZRC networks maintain a higher degree of cohesiveness than the mixed networks (89%, 31% and 32% for the *E.coli*, yeast and synthetic datasets).

### Systematic analysis of regulation in *Mtb*. Integration and simultaneous exploration of heterogeneous networks

We considered co-expression networks obtained with different approaches. Additional information is represented through networks, such as information on operons, GO annotation similarity, data base stored knowledge and sequence homology among others.

This integrative approach allows to systematically explore functional modules in the network and it is highly complementary to existing bi-clustering and other module identification methods. Even when additional biological information is included, biclustering methods have to face the challenge of interpreting the function of each groups of genes. Therefore, a general approach to detect and understand functional modules in a given organism is to simultaneously explore the location of the genes in a given bicluster in the co-expression networks. We have done so a to analyze the output of the cMonkey algorithm [22] on *Mtb* data. We have run multiple instances of the algorithm to bias the search towards the detection of both putative co-regulated and functionally related sets of genes. The obtained bi-clusters are presented in Additional file 2. To further investigate these biclusters, we have projected them in the multiple networks and genes proceed to their in-depth analysis. An example of how this is done is shown in the following section when we analyse the regulation of DNA damage repair systems in *Mtb* by LexA.

In a given co-expression networks it might very well happen that the effect of one regulator on a given set of genes overshadows the identification of other regulators. However, these mechanisms can be uncovered when comparing different networks generated under different experimental conditions. We have developed the pipeline presented in Figure 2 to uncover additional layers of regulation based on the assumption that regulation proceeds differently upon different perturbations, therefore some regulatory events will only be detectable in subset of conditions. This pipeline is only useful when the user suspects or has prior knowledge of an additional regulatory interaction affecting the same set of target genes. Therefore, we have used it to further explore the regulation of DNA repair systems in *Mtb*, where additional regulators are known to exist [64].

In addition, our approach allows to systematically explore and interpret additional data such as ChIP-seq data, through the pipeline presented in Figure 3. Among the available data we have chosen to focus on one of the key subsystems key for *Mtb* survival in the host, the regulation of the response to hypoxia and the induction of dormancy program via DevR.

Furthermore, the simultaneous network visualization, allows the exploration of common trends in the networks. Instead of focussing on clusters of genes appearing in the co-expression networks and how these clusters are reflected in, for example, the network liked to the GO biological process annotation. The alternative approach can be taken and for each set of genes linked to a particular GO or COG category we can trace back their location in the network. This analysis can point to interesting effects. For example, we explored the genes coding for ribosomal proteins and in the different co-expression networks these genes appear to form a highly interlinked cluster. However, in all versions of the networks, the *rpmB2-rpmG1-rpsN2-rpsR2* operon (*Rv2058c-Rv2055c*) formed by genes coding for ribosomal proteins (S18-S14-L33-L28) appear as a separated set (see Additional file 8). The combination of this information with the network of interactions extracted from literature [30] led us to the analysis of the zinc uptake regulator, ZUR, and its targets.

### DNA repair systems in *Mtb*

#### RecA-LexA dependent DNA repair system: the SOS box

LexA is a repressor known to be involved in the control of mechanisms for DNA repair. Under neutral conditions LexA, dimerizes and binds to the SOS box, repressing its own expression and of other genes related to DNA repair. The consensus sequence of the SOS box for *Mtb* had been identified as the palindromic motif TCGAAC(N)^4^GTTCGA [65]. Upon DNA damage, RecA binds single stranded DNA (ssDNA). The complex RecA-ssDNA, stimulates autocatalytic cleavage of LexA, so genes repressed by LexA are induced. Acidic conditions trigger a similar response, since LexA can no longer dimerize, effectively preventing it from binding its target DNA sequence [66].

**Figure 6:**
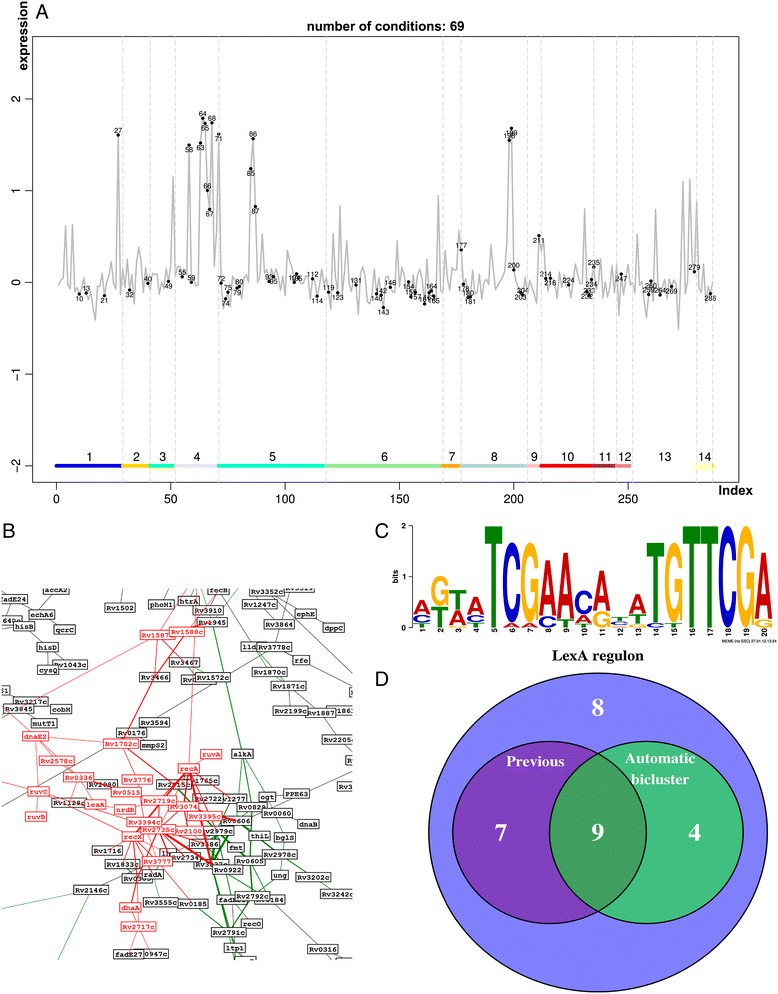
**LexA regulon. A)** Plot of the average expression level of the members of the LexA regulon across the different conditions. Red dots mark conditions with high (*>*0*.*8) correlation between the genes in LexA regulon. The horizontal bar and its different regions indicated by numbers refer to the classification of the conditions as described in Materials and Methods. High expression levels are observed in conditions corresponding to low pH or UV light. **B)** Clusters of genes involved in DNA repair mechanisms in the co-expression network (obtained from the combination of *R* ^*λ*^ and *C* ^*λ*^ with *λ* = √2). Genes regulated by LexA are marked red. **C)** Refined LexA identified binding motif, positions 14 and 15 were previously non specific. **D)** Number of genes identified to be regulated by LexA. *Previous* indicates genes previously reported in the literature as LexA regulated [65], whereas *Automatic* refers to the genes initially identified by the automatic biclustering algorithm.

The iteratively mapping of the genes in each of the biclusters presented in Additional file 2 to the multiple co-expression networks, showed that bicluster 36 is a 13 gene module that shares many of the characteristics of the LexA regulon. To further investigate this bicluster, we proceed to analyse the location of these genes in the multiple networks and to identify genes that might be linked to them through the systematic analysis of their expression patterns (see Figure 6A, B). For each of the candidates to be included in the regulon we combined information from the different information layers: literature, databases, functional annotation, and upstream sequence among others. We were able to identify a total of 28 genes putatively in the regulon, listed in Additional file 9: Table S1. This includes the 16 genes reported by Davis *et al.* [65] plus 12 additional ones, resulting in an effective enlargement of the regulon size by 75%, (see Figure 6D). Based on this extended regulon, we identified for the SOS box in *Mtb* a more specific motif: MKWMTCGAAMRYWTGTTCGA (depicted in Figure 6C.

To verify our predictions about the 12 genes not previously assigned to this regulon, we compared our predictions with the LexA binding regions identified by Smollett *et al.* [67]. In that work ChIP-seq analyses of LexA binding sites was complemented with experimental measurements of gene expression upon DNA damage induced by mytomycin C. The 12 genes that we assign to the LexA regulon contain a site in their upstream regions where LexA binding was detected. In addition, these genes also show dis-regulation in DNA damaging conditions. It is important to stress that the results from Smollett *et al.* were used in our analysis only to verify our predictions. Therefore, we can conclude that we have successfully reconstructed the regulatory interactions of LexA.

Davis *et al.* identified a single SOS box in the upstream region of the divergently transcribed genes *whiB2* and *fbiA*, however none of them were listed as likely to be regulated by LexA. The analysis of the expression profiles of these two genes, *fbia* and *whib2* led us to conclude that *fbiA*, a probable 2-phospho-L-lactate transferase involved in coenzyme F420 biosynthesis, does not belong to the regulon, since no significant expression changes are observed upon conditions related with induction of the LexA regulon (DNA damage or acidic pH). The expression pattern of *whiB2* gene shows anti-correlation with the rest of the genes in the LexA regulon in all the conditions in our compendium that show upregulation of LexA. Therefore, we concluded that LexA regulation of *whiB2* expression proceeds through a different mechanism than the previously described so that the dimerized form of LexA acts as an inductor of *whiB2* expression. This has also been independently confirmed in experimental conditions were mytomycin C was added to the medium [67].

WhiB2 has been hypothesised to be involved in the regulation of cell division [68]. Functional analysis of the genes co-expressed with *whiB2* further supports the idea that WhiB2 is involved in the regulation of cell division. We also found that not only *Rv2719c*, as reported by Davis and co-workers, but the complete *Rv2719c-nrdR-Rv2717c* operon is under the control of LexA. Information from the STRING database [33] (gene neighbourhood and co-occurrence) allows us to functionally link *Rv2717c* with DNA repair and cell wall synthesis; NrdR is involved in the control of the synthesis of dNTP needed for DNA replication and/or DNA repair [69]; and the cell wall hydrolase *Rv2719c* is involved in suppressing the cell cycle by altering localization of FtsZ rings [70]. Therefore, our analysis has unveiled that repression of *whiB2* and induction of the *Rv2719c-nrdR-Rv2717c* operon are the LexA regulated mechanisms to temporary arrest cell division upon DNA damage.

Furthermore, through the re-annotation of the newly discovered LexA targets, we can identify a putative functional mismatch repair (MR) system. No MR system has been previously identified in *Mtb*. A typical bacterial MR system contains: the mismatch-recognition protein MutS that contains an HNH endonuclease domain; MutH, a nicking endonuclease; and MutL, which acts as a scaffold between these. The MR system additionally contains a DNA helicase; a DNA exonuclease to remove the mismatching nucleotides; and a DNA polymerase together with a DNA ligase to repair and ligate the created gap [71]. The hypothetical MR system that we have identified is formed by: i) some of the 10 HNH endonuclease domain containing genes that belong to the 13E12 family, ii) the Holiday junction DNA helicases RuvA and RuvB together with RuvC, a crossover junction endo deoxyribonuclease, and iii) the DNA polymerase DnaE2, together with ImuA/B that are essential for its function [72]. The existence of this repair system, under the control of LexA, would solve the apparent inconsistency between the low mutation rates in *Mtb* and the absence of an MR system [73].

#### Additional regulation: RecA independent DNA repair system

RecA is key to the correct regulation of the LexA regulon and it is also LexA regulated. However, additional DNA repair mechanims have been described in *Mtb*, particularly the RecA non-dependent (RecA_ND) DNA repair system [64], that also regulates *recA* expression in a LexA independent manner. To analyse this additional regulatory layer, we have used the pipeline in Figure 2.

The conditions not linked to the main regulatory event are those 93 where no relationship was found between *recA* and *lexA* levels (see Additional file 5). Using these conditions we built a new co-expression network and afterwards we compared the original and the newly built networks. The members of the LexA regulon appear in the original network as a tight cluster, however, in the second network only a subset of them appear clustered. We selected the genes in this small cluster and proceed to an iterative round of motif identification and motif matching, to finally identify the genes regulated by the RecA_ND mechanism. Our approach does not allow us to identify the regulator of the set, but previous studies point to ClpR (Rv2745c) [74].

We have verified our predictions for the identified motif and the list of targets genes, (see Additional files 10 and 11) through comparison with literature data, since they match those previously described by Gamulin *et al.* [64] for the RecA_ND DNA repair system. However, there are two striking differences between our results and those previously reported by Gamulin *et al.* We don’t find that *sigG* responds to DNA repair, which matches the result reported by Smollett *et al.* [75], on the other hand, we do find another sigma factor, *sigC*, that seems to be regulated in response to DNA damage. The list of genes in the regulon, show that in this case, regulation of cell cycle arrest upon DNA damage is not linked to *whiB2*, but only to the *Rv2719c-nrdR-Rv2717c* operon.

### Hypoxia and induction of dormancy program: DevR regulon

One of the main characteristics of *Mtb* is its ability to switch to a non replicating or ‘dormant’ state that allows it to survive for a long time within the host and renders it less susceptible to antibiotics. The environment inside the host is hypoxic and it might have high concentration of CO or NO released by the host macrophages. Under either low oxygen concentrations or high concentrations of NO or CO, the heme iron from the kinases DevS (DosS) and DosT becomes ferrous, the kinases become CO or NO bound and they get activated. In the active form, DevS and DosT autophosphorylate and induce phosphorylation of DevR, which in turn, can bind its DNA recognition sequence and induce expression of the DevR regulon resulting in the activation of the dormancy program [76].

All the options to build the co-expression networks that we have explored, result in a tight cluster for the known members of the DevR regulon. This is to be expected since dormancy and DevR regulon induction have been an active research topic in the *Mtb* field. From the 287 distinct conditions present in the expression compendium, almost 10% of them (23) are conditions associated with expression of DevR regulon. However, the fact that these genes always appear forming a tight cluster, points to the absence of additional regulatory elements that might cause a differential expression of some members of this regulon.

**Figure 7:**
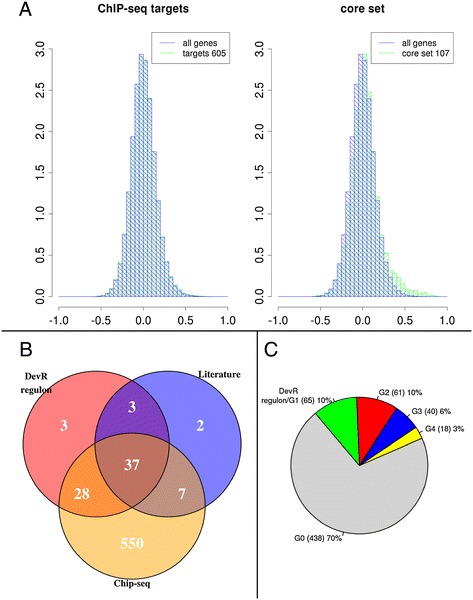
**DevR regulon. A)** *Left*: Histogram in blue represents the correlation among all the genes present in our compendium, whereas the histogram in green is based upon the correlations of the identified targets for which expression data is available within our compendium (605). Both show the same overall distribution. *Right*: Histogram in blue represents the correlation among all the genes present in our compendium, whereas the histogram in green is based upon the correlations of the 107 genes selected in the core set. Note there is a shift towards positive correlation values, pointing to a common regulatory influence over the selected genes. **B)** Number of genes identified in the DevR regulon compared to the number of targets identified through ChIP-seq experiments or the ones cited in literature [32,76–78]. **C)** Group assignment of the 622 targets identified by ChIP-seq for DevR. G0 contains genes for which non discernible expression pattern has been found. G1 correspond to the usually named DevR regulon, whereas G2-4 contains genes that show correlated expression patterns, although these patterns are not consistent with the previously described behaviour of DevR regulon. A detailed list of these genes is available in Additional file 12 and the output of the GO-enrichment analysis is shown in Additional file 16.

We have selected this regulon to validate our methodology for ChIP-seq data analysis. ChIP-seq data corresponding to over-expression of DevR were obtained from TBDB [25]. The processed results for DevR contain 475 detected peaks, that correspond to 622 genes that could be possibly regulated by DevR, although in our compendium expression data for only 605 of them were available. We have analysed this dataset following the methodology shown in Figure 3, and defined a core-set containing 107 genes. The analysis of the correlations in the expression profile among this set as compared to the overall distribution indicates a common regulatory influence over the selected genes, Figure 7A. Further analysis of the genes in this core set and their behaviour across the conditions in the compendium lead us to identify five distinct groups of genes within the identified targets (see Figure 7C and Additional file 12). In four of these groups there is a high correlation among the genes whereas no clear pattern can be identified in the expression of the genes in the fifth group. One of these groups, that from now on we will refer to as the DevR regulon, contains 64 genes that were identified by ChIP-seq, has an expression pattern consistent with the previously described DevR regulon, and 37 of these genes have been previously identified as DevR regulated genes (Figure 7B). Additionally, we have found among the list of targets from TBDB, 7 genes that have been previously reported as DevR regulated [32,76–78] however their expression patterns suggest that either they are not regulated by DevR or there is a secondary regulatory event altering their expression levels, therefore it is arguable whether they can be included in the regulon. A detailed list of members of this regulon is provided in Additional file 13: Table S2. The genes in this table have been assigned to functional categories: cell wall, transport elements, anaerobic respiration, translational machinery, regulatory elements and elements related to stress response. These six elements are linked to some of the main changes observed during growth arrest and dormancy, such as the changes in the cell wall, the arrest in protein synthesis and the adaptation to a hypoxic environment with reactive nitrogen species [79,80].

Interestingly, we found a faint link between the type VII secretion system (Esx-3) genes *Rv0282-Rv0290* and DevR. Although the correlation analysis shows that they are not members of DevR regulon, we indeed find that, in a reduced set of 27 conditions they show a high (0.7) correlation with DevR regulon (see Additional file 14). These genes are also known to be regulated by Zur (Zinc uptake regulator) [81] and IdeR (Iron dependent regulator) [82] and are required for mycobactin-mediated iron acquisition [83] in *Mtb*.

*Rv1734c* belongs to the set of genes that had been previously reported as DevR regulated but for which our analysis shows that their expression pattern is not compatible with this assertion. Our analysis is based on transcript levels, so it could be that the regulation of Rv1734c happens at the post-transcriptional level. However we have found additional evidence, albeit indirect, of our prediction. Chauhan *et al.* [84] analysed the effect of mutations in the DevR binding motif and found that positions 5 (G), 7 (C) and 9 (A/T) are essential and a substitution in any of them dramatically reduces the binding affinity (see Additional file 15 for the DevR binding motif). In addition, our analysis shows that position 6 always contains an A. Motif analysis complements the results from the analysis of expression data and further supports our prediction that *Rv1734c* is not regulated by DevR, since its putative binding site contains a mutation (to G) at position 9.

Among the targets identified by ChIP-Seq four other groups emerge (see Figure 7C and Additional file 12) however none of these groups show significant up or down regulation in the conditions associated with DevR regulon induction. No clear common behaviour can be detected among the genes in group G0. These could be considered as false positives as a result of the *devR* over expression performed prior to the ChIP-seq procedure [25]. However the genes within each of the other groups (G2, G3 and G4) show a consistent co-expression across the conditions in the compendium, therefore we believe that these hits should not be discarded as being caused by non-biological reasons, instead alternative explanations such as weaker binding to regulate overall or regulation through a transcription factor homologous to DevR should be further explored. In addition the genes in each group are functionally related (see Additional file 16), specifically, genes in G2 are mostly linked to translation and might be linked to the translation arrest observed during dormancy. Genes in G3 and G4 are functionally linked to ‘metabolism’, ‘stress’ and ‘cell wall formation’, which are significantly different in the non replicating state.

### Zinc uptake regulator ZUR

**Figure 8:**
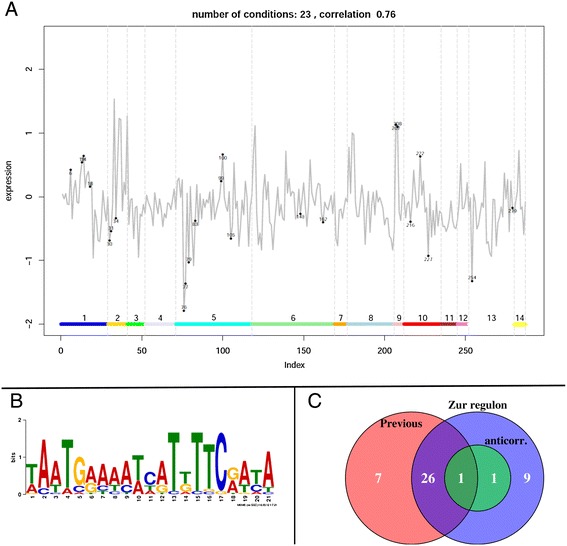
**ZuR regulon. A)** Bicluster formed by members of the ZuR as reported in literature [81]. The grey line represents the average expression levels of the members of ZuR regulon in the conditions in our compendium. The numbers identify the 23 conditions that have been included in the bicluster. The horizontal bar and the different regions indicated by numbers refer to the classification of the conditions as described in Materials and Methods. Notice the clear up-regulation of this set in conditions of type 9: Transcription inhibition, in particular these values correspond to experiments were Rifapentine was added to the medium. For clarity, expression values have been scaled, so that the mean value for each gene when all conditions are considered is zero. **B)** Identified ZuR binding motif. E-value 4.2*10^−49^. **C)** Number of genes in the ZuR regulon (Additional file 17) compared to the ones previously identified [81], the set *anticorr*. contains *Rv0232* and *lpqR*, that show anticorrelation with the rest of the genes in the regulon.

As previously stated, the systematic analysis of genes linked to the different COG categories, showed that in the different co-expression networks the *rpmB2-rpmG1-rpsN2-rpsR2* operon (*Rv2058c-Rv2055c*) appeared forming a distinct cluster, separated from the rest of ribosomal protein coding genes (see Additional file 8). In addition, this operon also appears linked to the *ppe3-Rv0281* operon*.* Therefore, we concluded that these two operons should be regulated by a specific mechanism and respond to a specific type of perturbations. The location of these genes in the network of known regulatory interactions [30], pointed to the zinc uptake regulator ZuR (Rv2359) as the most likely regulator of both operons. Therefore we set forth to study the possible targets of this TF. Initially we selected from the network extracted from the literature, a list of regulatory targets of ZuR that had been verified either by more than one source or by an appreciable upregulation in a *zur* knock out mutant [81]. Once this core set was selected, we proceed to identify the subset of conditions where these genes are co-expressed. The method to construct the expression plots (see Materials and Methods) was used to select 23 conditions were the members of the core set were correlated (average correlation 0.76, see Figure 8A); similarly we selected a core subset set of 35 conditions (average correlation of 0.65) and another subset of 5 conditions (average correlation 0.92). The correlation among the different putative members of the ZurR regulon (identified by motif elicitation and matching) was used as a signature to identify the other members of the regulon. The list of genes identified as belonging to this regulon is provided in Additional file 17 and the ZuR consensus binding motif is depicted in Figure 8B. The correlation analysis shows the importance of the biclustering approach to select only those conditions where no additional regulatory influences hinder the discovery of the members of the regulon. For example, if we analyse the correlation of *rpmB1* with the members of the core set across all conditions present in our compendium, we obtain no correlation (0.02). However when we compute the correlation between *rpmB1* and the genes assigned to the core set but only considering the previously selected subsets of conditions the correlation values raise to 0.63, 0.71 and 0.93 respectively, showing that, as previously reported, expression of *rpmB1* is indeed regulated by ZuR.

Our predictions closely match the list proposed by Maciag *et al.* [81], however some differences in the target assignment appear (Figure 8C). From the 34 previously assigned genes to the ZuR regulon, we can only confirm that 79% (27) of them show correlated expression patterns. In addition, among our predicted targets there are 10 genes for which no experimental evidence can be found in the literature. These predicted additional members of the regulon are: *Rv0223c, lpqR (Rv0838)*, *Rv1057*, *pe15* (*Rv1386), ppe20 (Rv1387)*, *Rv2617c, Rv2618*, *Rv2619c, Rv3018c* and *Rv3018b. Rv0232* and *lpqR* appear anti-correlated with the genes in the previously defined core set. In the case of *lpqR* this anti-correlation clearly increases when restricting the set of conditions. This together with the presence of ZuR binding motif in its upstream region, leads us to conclude that regulation of *lpqR* (and possibly of *Rv0232*) expression proceeds through a different mechanism than that of the rest of the genes in the regulon.

Additionally, it is striking to notice the clear up-regulation of this set of genes in conditions where transcription was inhibited by addition of Rifapentine to the medium (conditions 207 and 208 from Additional file 1). This shows that, at least regarding these genes, the inhibition of transcription cause by Rifapentine has a similar effect as zinc limitation. However, a detailed explanation of this phenomenon is most likely unreachable from the analysis of the present dataset.

## Conclusion

We have shown that by integrating data from different sources and through the combined analysis of data, we are able to obtain insights into the biological system under investigation that go beyond the specific research questions of each experimental design. Our systems level approach has allowed us to analyse in depth publicly available data on *Mtb* and has enabled us to extract valuable new information from the existing datasets. The processes that we have studied (namely: adaptation to hypoxia, low zinc availability and DNA damage repair systems) are paramount in allowing *Mtb* to thrive within the hostile host environment. We generated comprehensive lists of genes involved in the response to such environmental conditions. These compendia summarize and substantially extend and modify the current knowledge. We believe that any further research on these adaptation mechanisms will make extensive use of this new knowledge and the hypotheses generated herewith.

We have developed a framework that allows the user to integrate information from gene or protein expression experiments, genome annotations and existing databases together with analysis tools. Most of the already existing databases and tools provide human user interfaces that only allow for querying one gene at a time and only provide a subset of the total available information. Within our framework this integration is done at once on a genome-scale, by using the gene co-expression networks. Instead of obtaining a network with the majority vote from the algorithms we keep the individual output from each algorithm and rely on the expertise of the user to select which of them contains, for each particular problem, the highest amount of relevant information. This way of presenting the information greatly speeds up the process of analysing new data, since after the study of the different networks and the modules that each of them show, expert users will be able to quickly understand the changes occurring during a new experiment just by overlaying the new expression data over the networks and looking for the networks were the differentially expressed genes or proteins appear clustered together. Our approach is not, in itself a new analytical approach but a general framework that facilitates the consideration of multiple pieces of evidence to pose a hypothesis on a biological system.

Comparison with experimentally verified networks (*E. coli* and *S. cerevisiae*) and with *in silico* generated datasets shows that the co-expression networks generated using our pipeline preserve the desired modularity of transcriptional networks and the regulatory targets of a TF tend to appear in the networks as forming a closely interconnected module. Comparison with *E. coli* data, shows that in 97% of the cases the target genes of a TF form clusters in the network. Modularity was on average less preserved for *S. cerevisiae* networks (57% of the cases), due most likely to the increased complexity of regulatory interactions in eukaryotes. This poorer performance of the inference methods for the yeast dataset was also observed in the DREAM5 challenge were at most a 0.25 recall was obtained by any of the tested methods. On average the ZRC networks perform better that the mixed ones, but our analysis also showed that both types of networks contain complementary information, for example the targets of a yeast's YPR199C are only identified as forming a module in the mixed network (see Additional file 6).

We have established a protocol to assign regulatory interactions to binding sites identified through ChIP-seq experiments via the integration of expression data. We have used this approach to correctly identify the target genes of a given TF (DevR) as a response to an specific type of perturbation (dormancy inducing conditions) among the hundred of candidates from the experimental dataset. Within our framework biclusters can quickly be analysed, interactions between the biclusters can be identified in the overall networks and biclustering methods can easily be transformed into a tool for automatic detection of functionally related modules or underlying layers of regulation. Moreover, we have developed a method that allows to identify additional regulatory layers. The comparison of the networks generated by considering different subsets of conditions allowed us to distinguish various regulatory mechanisms for DNA damage response.

The analysis of ZuR regulon shows the potential of an integrative approach. In our compendium there were no data corresponding to experiments designed to analyse the effects of zinc limitation on *Mtb*. However, the analysis of the expression patterns of the genes in the different conditions and the analysis of their correlations, allowed us to select a set of conditions that would complement the bioinformatic analysis of the upstream sequences of the genes and would allow us to decided which of the regions similar to the motif actually represents a ZuR binding site and characterize the members of the regulon. We were able to compare our predictions with dedicated experiments performed with knock-out mutants and we found a good agreement between computational predictions and experimental data.

Our work extends the already existing knowledge and produces a comprehensive list of the members of the DevR regulon. Among the targets identified by ChIP-seq, we have uncovered three additional sets of genes that show a consistent expression pattern across the conditions in the compendium and are functionally related. Additional studies are required to further understand the regulation of these genes and their possible link to the non-replicative state. The discovery of new regulatory mechanisms involved in dormancy has the potential to deliver a new set of drug targets.

The automatic biclustering was the basis of our analysis of DNA repair systems and specifically LexA regulon. We were able to confirm our computational predictions through the comparison we literature data and recently performed ChIP-seq experiments. In addition, our work has produced a more specific binding motif for LexA through the identification of new members of its regulon. Additionally, the re-annotation of the identified new targets allowed us to identify a putative MR system in *Mtb*. The analysis of mutations and mechanisms to avoid them is of the uttermost importance for the further understanding of the evolution of antibiotic resistances and pathogenicity.

Previously existing data were used to verify our predictions on the regulatory mechanisms of DNA damage response. We have clarified some points previously in dispute, such as the lack of involvement of *sigG* in the response to DNA damage and the regulation of the alternate sigma factor *sigC* in these conditions. Correct identification of the sigma factor up-regulated upon DNA damage is key to understanding the systemic response of *Mtb* to this damage type. In addition, our work has provided further evidence on the mechanisms leading to cell cycle arrest upon DNA damage in *Mtb*.

In addition, we identified a new regulatory mechanism for ZuR, since the analysis of the upstream regions of its target genes and their expression patterns show that *Rv0232* and *lpqR* belong to its regulon, although their regulation must proceed through a different mechanism.

Here, we have presented the results obtained by applying our integrative approach to *Mtb* and whenever additional data was available, we have found good agreement between predictions and experiments. The basic underlying principle of this approach relies on the comparison of the networks obtained using different sources of information or methodologies. This approach can be readily extended to different organisms and to the comparison between different species, by using global identifiers together with a database of orthologous genes between species. This would allow to select a gene or group of genes in one organism and see how they are arranged in the networks corresponding to a different organisms. In addition, other types of data, such as synteny or evolutionary information, and protein structure and families could improve the evolutionary comparison of functional modules.

## Additional files

Additional file 1:
**Microarray data sets considered in this meta-analysis.** Identifiers from the Gene Expression Omnibus Database have been used for each sample, experiment and platform. Additional file 2:
**Identified biclusters.** The following information is presented for each bicluster. **Left**. *Top* *:* expression plot, the grey line represents the average expression levels of the genes in all the conditions in this meta-study, the numbers identify the conditions in the biclusters. The horizontal coloured line shows the classification of the conditions (as detailed in Additional file 1). *Middle* Interactions among the members of the biclusters either from protein-protein interactions (STRING and ProLinks); operons or similarity of annotated COG terms. The nodes in the network represent the genes in the biclusters. The colours are linked to the COG annotation, whereas the shape is linked to the functional classification: square: information storage and processing; rhomboid: cellular processes and signalling; triangle: metabolism; and circle: poorly characterized. *Bottom*: Identified motifs. **Right**: genes in the bicluster, their upstream sequences have been plotted together with a representation of the location of the detected motifs: red, green and blue for the first, second and third motif (if present). Additional file 3:
**Pipeline for the generation of co-expression networks.** The R code that implements the pipeline as depicted in Figure 1. The inequality simplifier is implemented in the joined jar file. Additional file 4:
**Technical characteristics of the visualization tool.** Technical description of the network visualization and comparison tool used. Additional file 5:
**Relationship between the expression levels of** ***recA*** **and** ***lexA.*** The fit corresponds to a model, based on ordinary differential equations, of LexA mediated induction of *recA*. The numbers signal the conditions where this model actually holds. For clarity, expression values have been scaled, so that the mean value for each gene, when all conditions are considered, is zero. The horizontal bar includes the overall categorization of the conditions (see Materials and Methods). Notice that the conditions were LexA is regulating *recA* expression are mainly linked to 1: aromatic amides intra cellularly hydrolysed, low pH; 4: Acidified medium; 5: Cell wall synthesis inhibition; and 8: DNA damage. Additional file 6:
**Comparison of predictions for individual methods to consensus network.** **A)** Overall predictions. The plot shows the number of interactions from the gold standard that were predicted worse, equally or better by each individual method than by the consensus network [15]. The predictions were assessed comparing the rank of the predictions with a 10.000 cutoff. Only the *E. coli* network has been considered. **B)** Predictions for each TF. Fraction of the interactions that are better predicted by each type of method than by the consensus network. Only TF with more than 20 interactions have been considered. These plots have been built using the methods described in [15]. Additional file 7:
**Topological overlap of TF targets in the co-expression networks obtain for the DREAM5 challenge datasets.** For each dataset (synthetic, *Escherichia coli* and *Saccharomyces cerevisiae)* the ZRC and mixed networks were built. Only TF with more than 5 experimentally verified targets (in the gold standards) were considered. Dashed line represents the average topological overlap in each network. Additional file 8:
**Relative locations of the cluster of ribosomal proteins and the operon of ribosomal proteins** ***rpmB2-rpmG1-rpsN2-rpsR2*** **in different co-expression networks.**
Additional file 9: Table S1. LexA regulon. Additional file 10:
**Genes in the RecA_ND regulated DNA damage repair set. Operons and divergons are marked by alternating (grey/white) row colours.** The fifth column indicates whether the targets had been previously identified [64]. Additional file 11:
**Identified motif in the upstream regions of the genes in the RecA_ND DNA repair system.** Logo generated using MEME [54]. E-value 6.0 E-60. The location of the hits is included in Additional file 10. Additional file 12:
**Classification of DevR targets identified by ChIP-seq.** The column TBDB indicates whether the target was listed in the original data set as a possible target for DevR regulation, some genes have been added to the table based on operon/divergon analysis. Hit location indicates the genomic location of the hit. The last column (in core set) refers to the initial classification of the genes within the core set. The column References refers to whether they have been previously identified as members of DevR regulon: **A** identified by Park et al. [76]; **B** identified by Balázsi *et al.* [32]; **C** identified by Bartek *et al*. [77]; **D** identified by Chao and Rubin [78]. 5 groups are present in the table. "The group 0 contains those genes whose expression patterns showed no significant correlation with any others, and we believe are not regulated by DevR, however they have been previously identified as belonging to DevR regulon in literature, therefore we have included them in the table. The first group corresponds to the members of DevR regulon. Additional file 13: Table S2. DevR regulon members. Additional file 14:
**Bicluster formed by members of DevR regulon and the type VII secretion system (Esx-3) genes** ***Rv282-Rv290.*** The grey line represents the average expression levels of these genes in the conditions in our compendium. The numbers identify the 27 conditions that have been included in the bicluster (Additional file 1). Additional file 15:
**DevR Motifs Top: Previously identified primary and secondary motifs for DevR** **[** 84 **] Bottom: Newly proposed motifs.** Main differences in primary motif can be seen at positions 6, 8, 1 and 2. Main differences in secondary motif can be seen at position 13 and 8 to 11. Additional file 16:
**Results of the GO enrichment analysis performed in the classified DevR targets identified by ChIP-seq.** *Node Size* reefers to the number of genes annotated to that particular GO term in the whole genome annotation file, whereas *Sample Match* indicates the number of genes annotated to the GO term in the group (the detailed list is given in the *Sample Key* column). Additional file 17:
**Members of ZurR regulon.** Operons and divergons are marked by alternating (grey/white) row colours. Rows signalled in yellow refer to genes that have been previously identified as belonging to this regulon, but for which our analysis does not support this claim. The fifth column indicates whether the targets had been previously identified by Maciag *et al*. and the sixth column reflects the reported fold change in a zurR knock out mutant [81]. Column 7 contains the definition of the core set of genes, whereas columns 8 to 11 contains the average correlation between the expression level of the each gene and the genes in the core-set across all conditions in the compendium or a subset of them.

## Abbreviations

TBDB: TB database
TF: Transcription factor
MI: Mutual information
*Mtb*: *Mycobacterium tuberculosis*
MR: Mismatch repair
GO: Gene ontology
DPI: Data processing inequality
ssDNA: Single stranded DNA
RecA_ND: RecA non-dependent
AUROC: Area under receiver operating characteristic curve
AUPR: Area under precision recall curve
