# Supplementary material for: Integration of heterogeneous molecular networks to unravel gene-regulation in Mycobacterium tuberculosis
Source: BMC Syst Biol. 2014 Sep 26;8:111. doi: 10.1186/s12918-014-0111-5 (PMC4181829; doi:10.1186/s12918-014-0111-5)
Supplement: Additional file 2: — Identified biclusters. The following information is presented for each bicluster. Left. Top : expression plot, the grey line represents the average expression levels of the genes in all the conditions in this meta-study, the numbers identify the conditions in the biclusters. The horizontal coloured line shows the classification of the conditions (as detailed in Additional file 1). Middle Interactions among the members of the biclusters either from protein-protein interactions (STRING and ProLinks); operons or similarity of annotated COG terms. The nodes in the network represent the genes in the biclusters. The colours are linked to the COG annotation, whereas the shape is linked to the functional classification: square: information storage and processing; rhomboid: cellular processes and signalling; triangle: metabolism; and circle: poorly characterized. Bottom: Identified motifs. Right: genes in the bicluster, their upstream sequences have been plotted together with a representation of the location of the detected motifs: red, green and blue for the first, second and third motif (if present). [file 12918_2014_111_MOESM2_ESM.pdf]

bicluster 1 ; 31 genes and 154 conditions

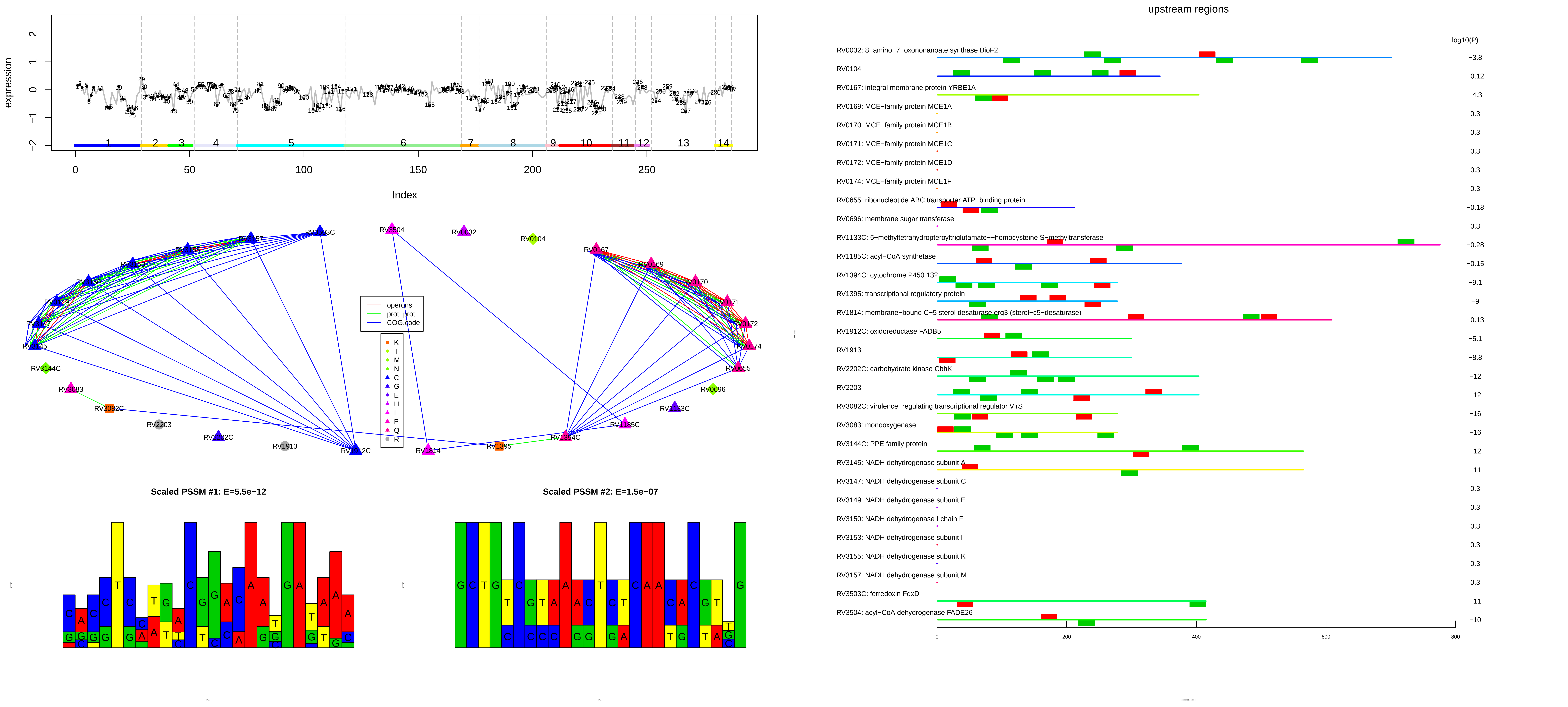

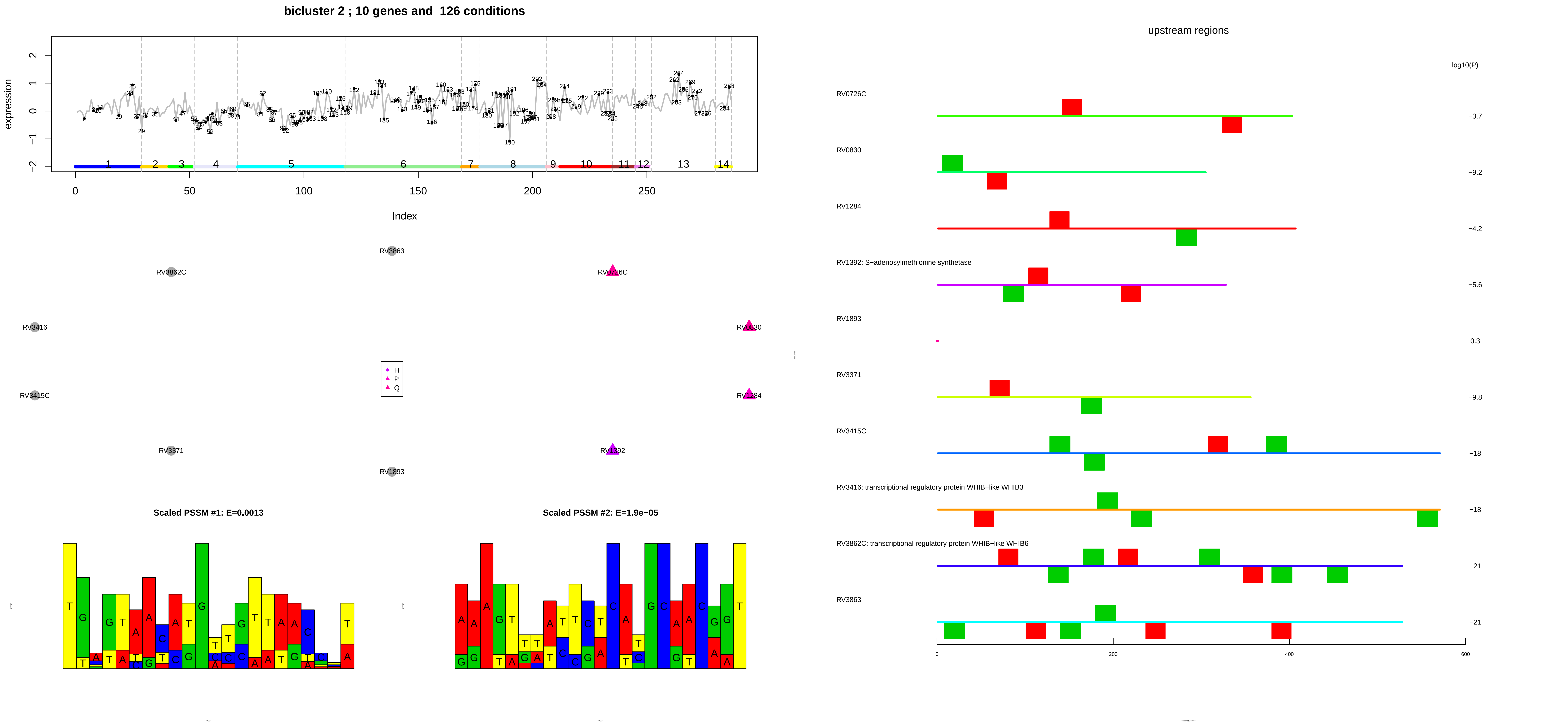
bicluster 2 ; 10 genes and 126 conditions
expression
Index
upstream regions
log10(P)
RV0726C
RV0830
RV1284
RV1392: S−adenosylmethionine synthetase
RV1893
RV3371
RV3415C
RV3416: transcriptional regulatory protein WHIB−like WHIB3
RV3862C: transcriptional regulatory protein WHIB−like WHIB6
RV3863
−3.7
−9.2
−4.2
−5.6
0.3
−9.8
−18
−18
−21
−21
H
P
Q
Scaled PSSM #1: E=0.0013
Scaled PSSM #2: E=1.9e−05

bicluster 3 ; 254 genes and 176 conditions

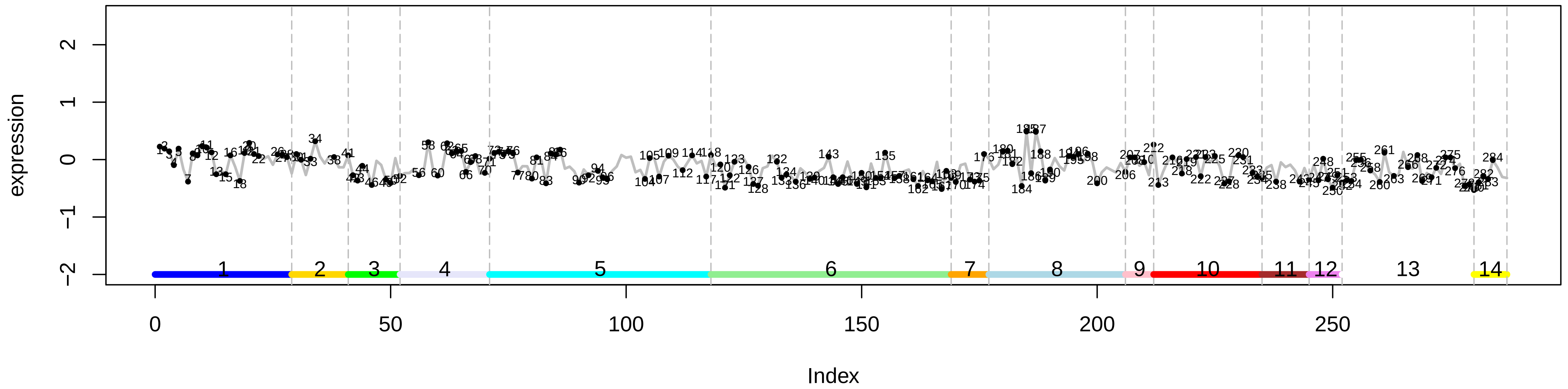

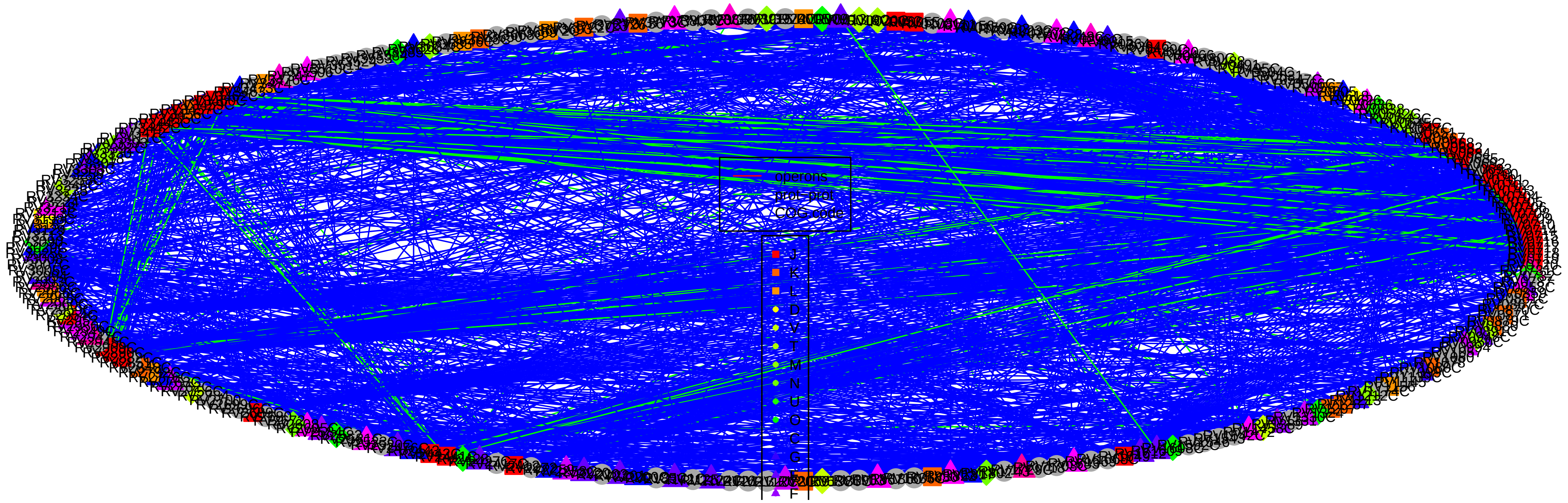

Scaled PSSM #1: E=1.8e−187

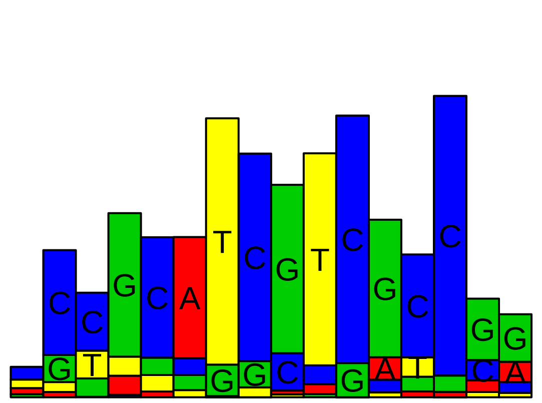

Scaled PSSM #2: E=3.6e−97

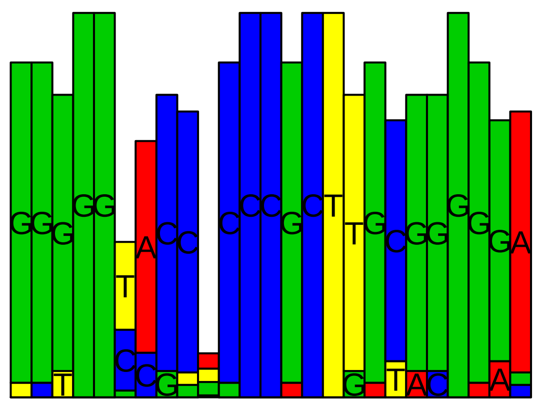

Scaled PSSM #3: E=7.3e−113

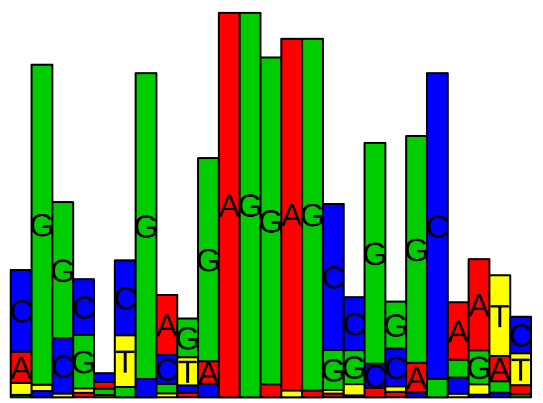

upstream regions

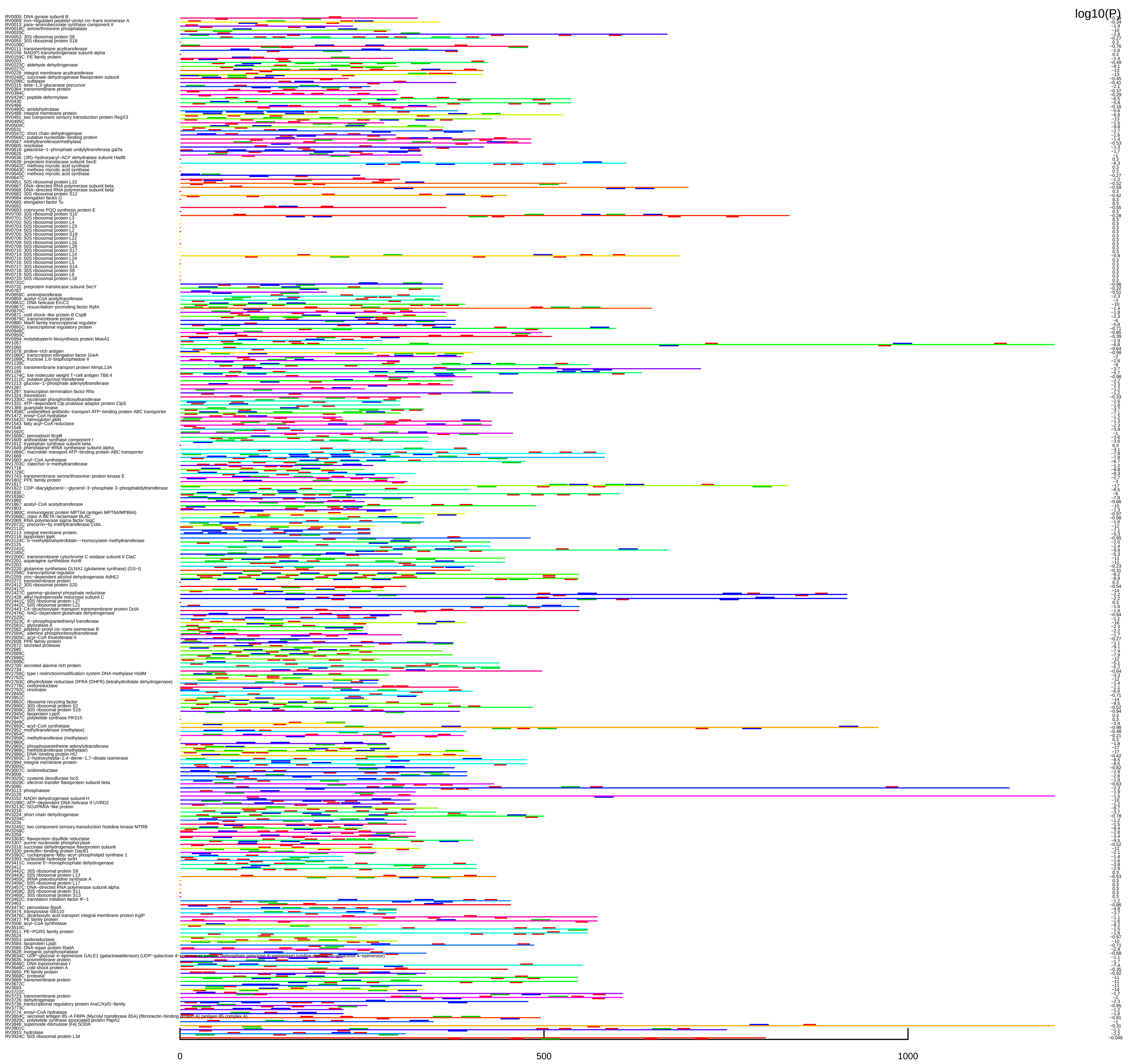

bicluster 4 ; 6 genes and 136 conditions

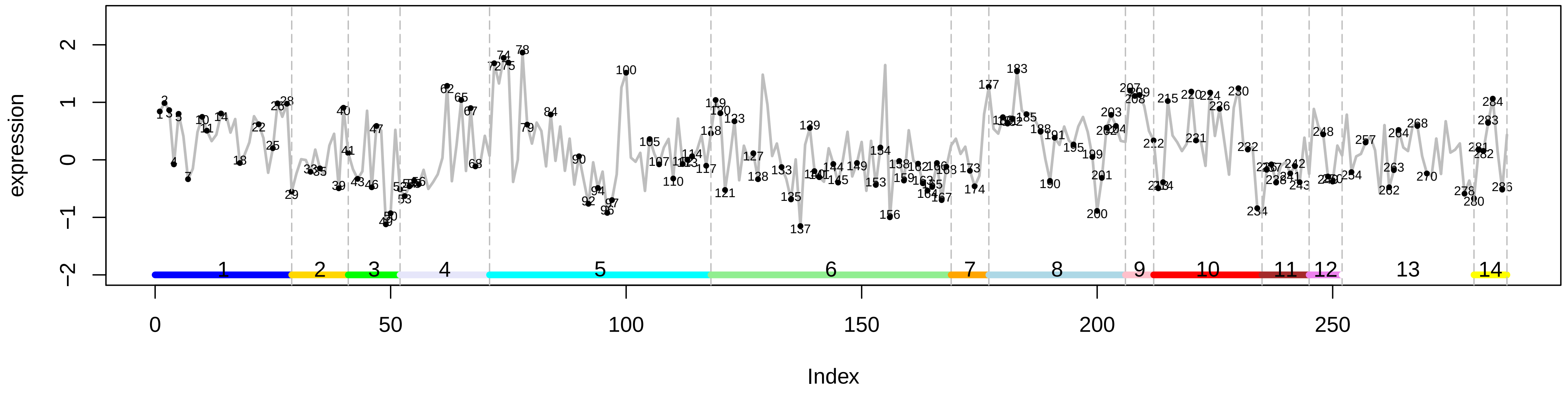

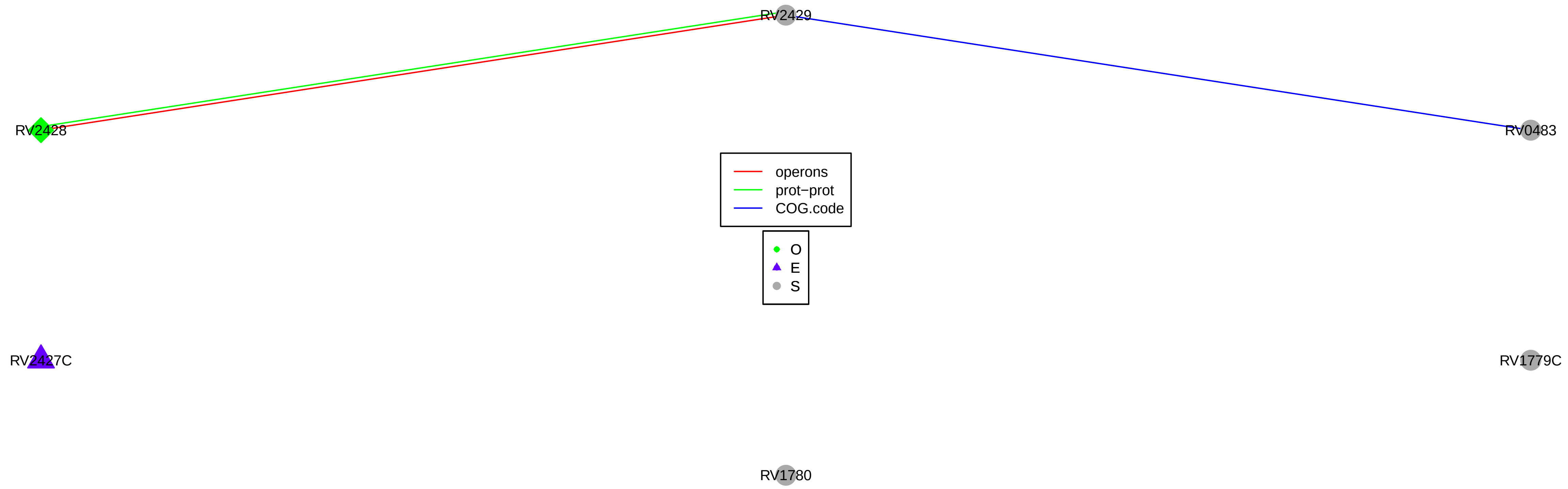

Scaled PSSM #1: E=5.4e−05

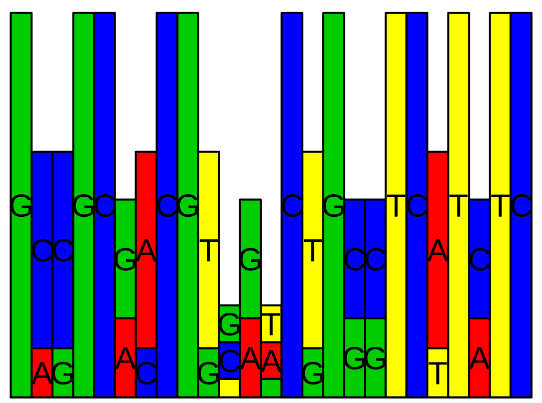

Scaled PSSM #2: E=2.4e−05

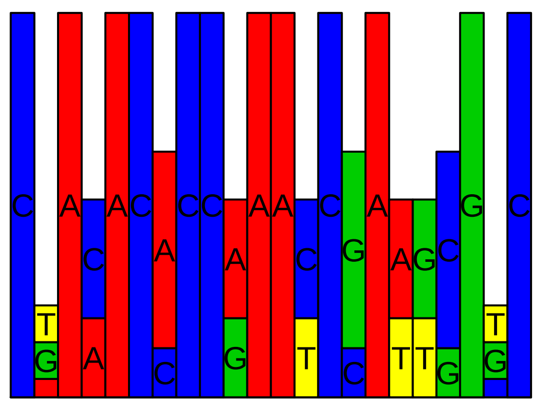

Scaled PSSM #3: E=0.013

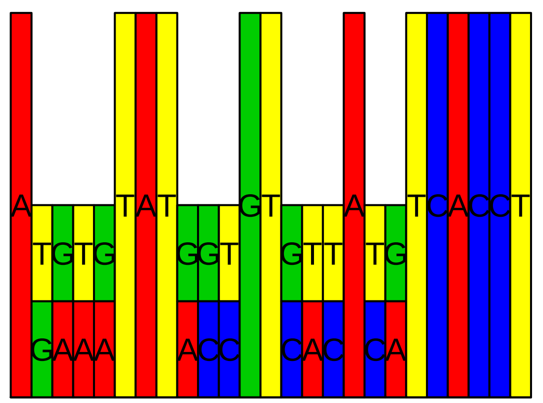

upstream regions

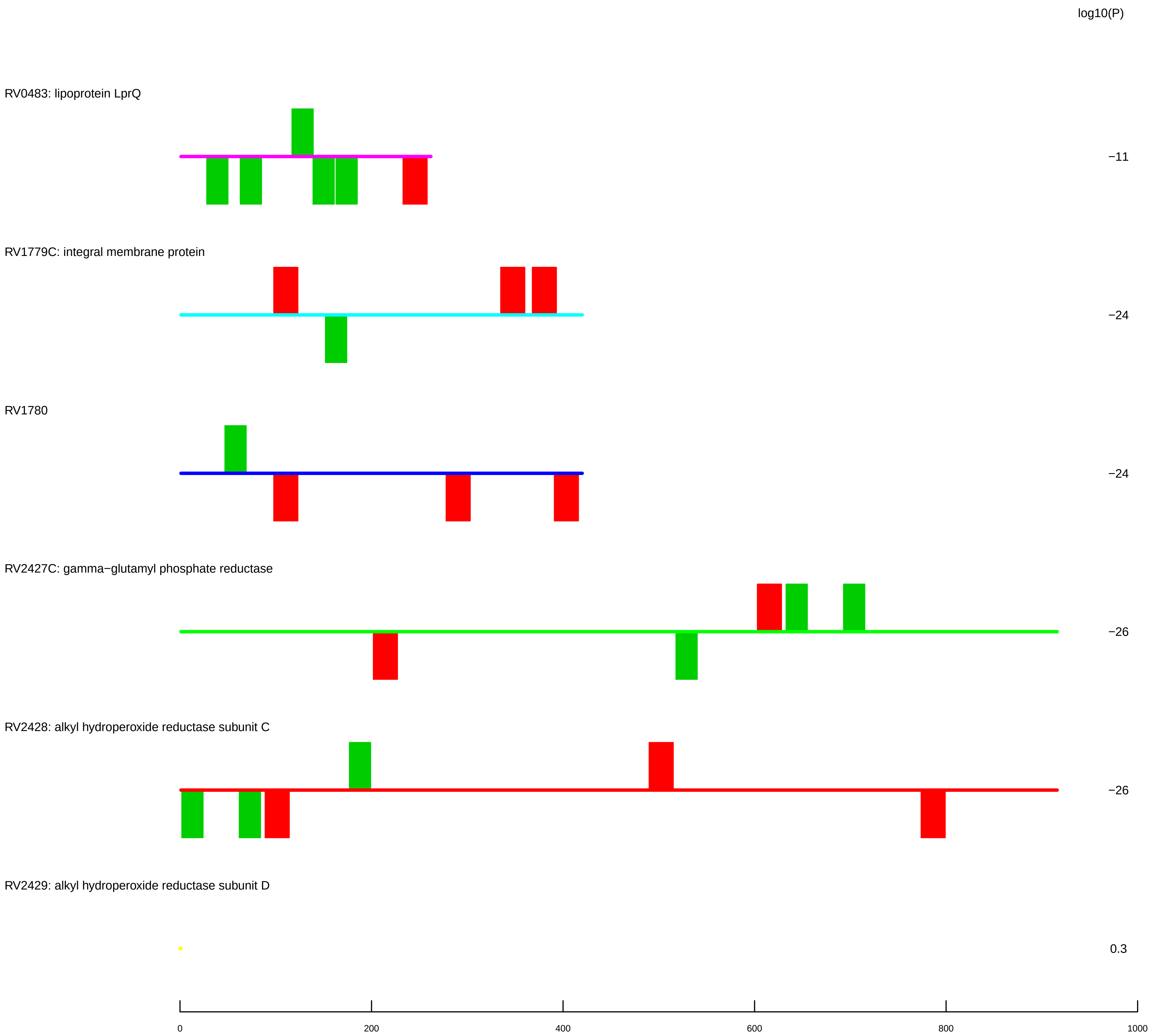

bicluster 5 ; 24 genes and 164 conditions

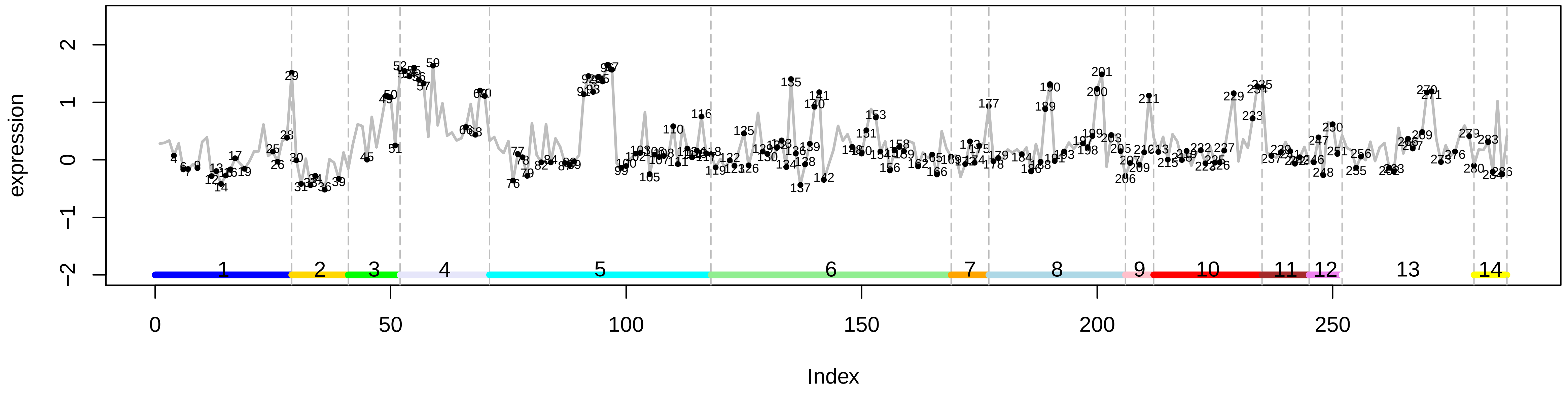

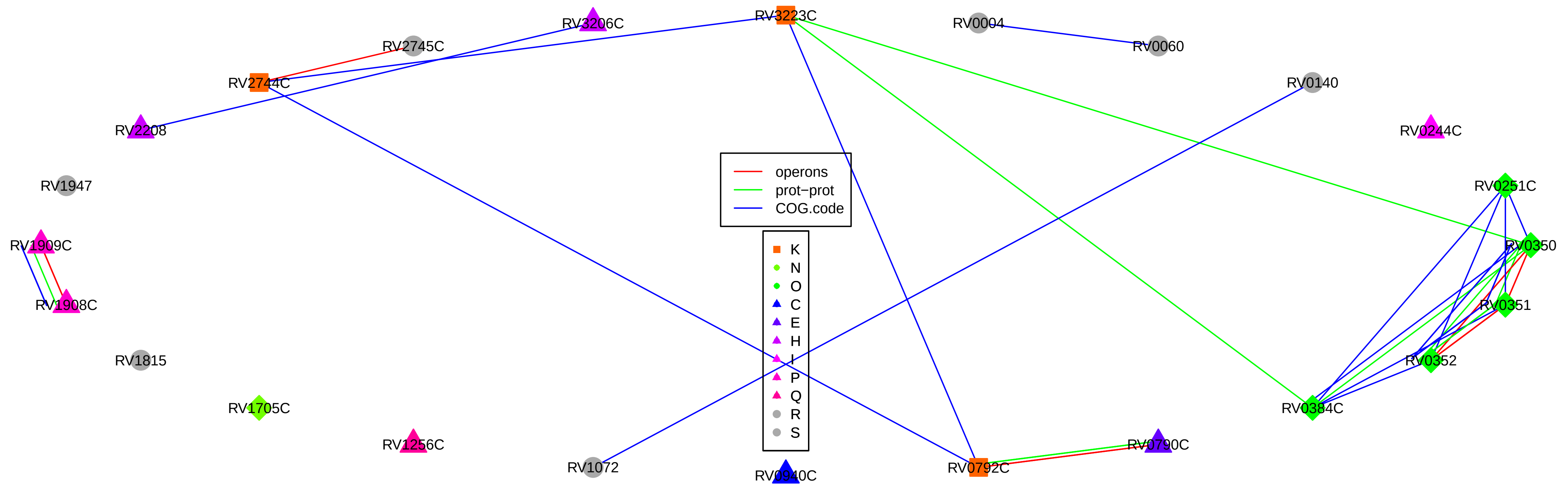

Scaled PSSM #1: E=0.00088

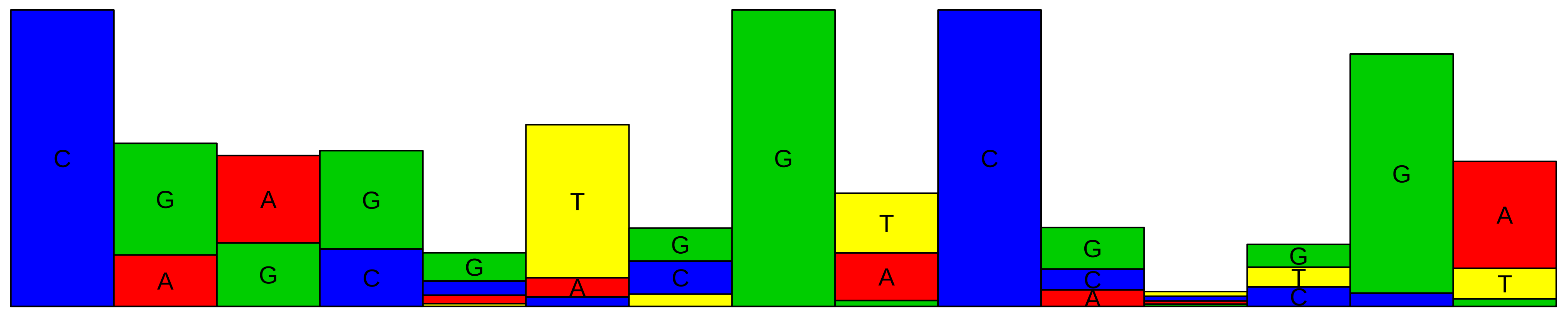

upstream regions

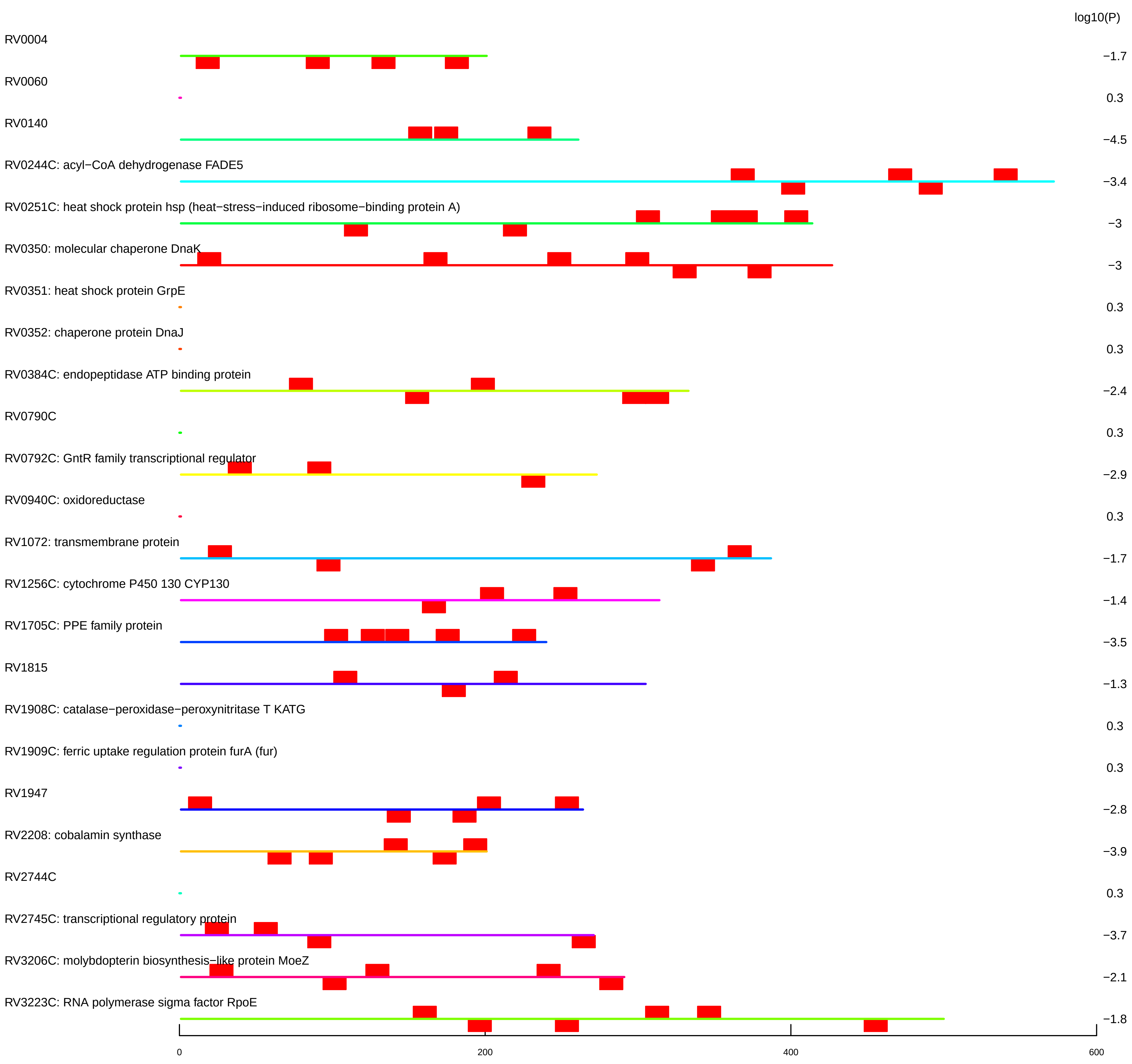

bicluster 6 ; 49 genes and 74 conditions

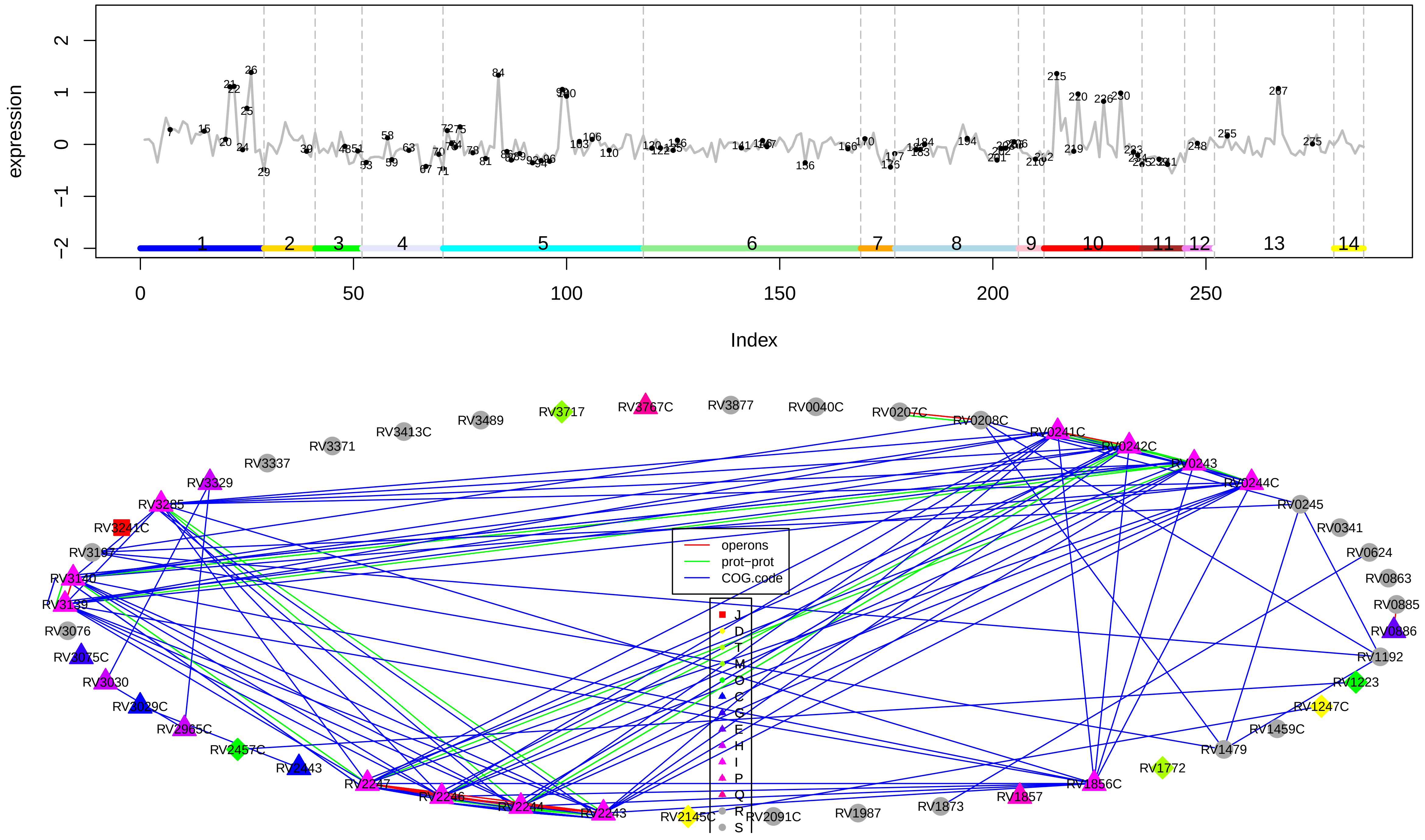

Scaled PSSM #1: E=4.5e−14

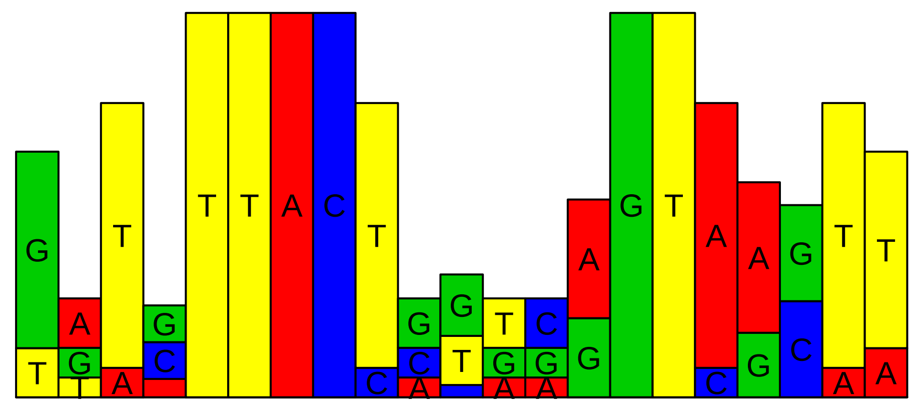

Scaled PSSM #2: E=0.0094

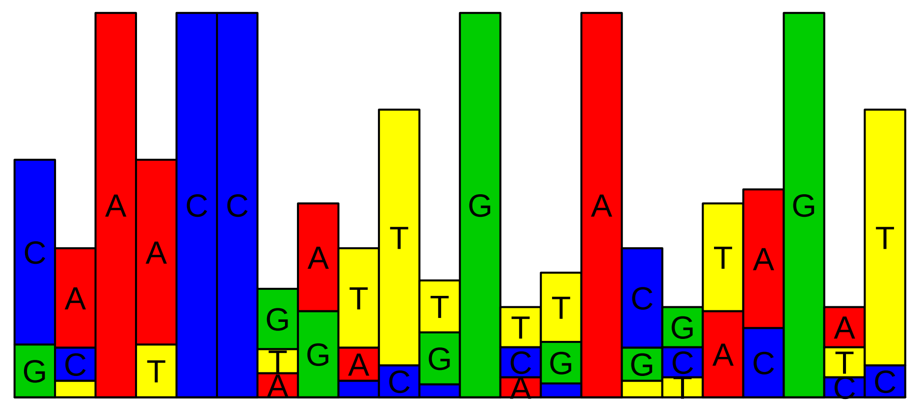

upstream regions

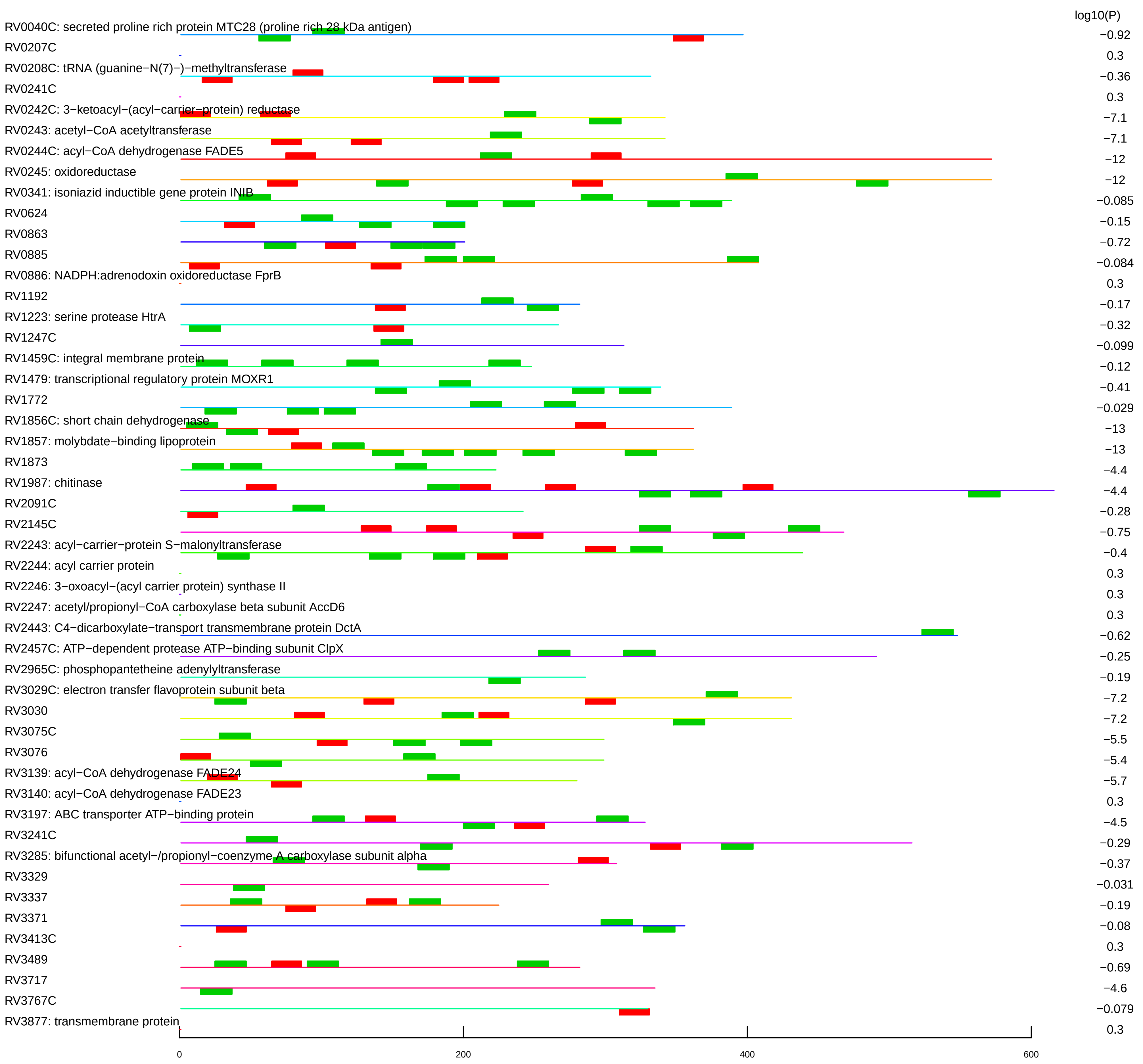

bicluster 7 ; 21 genes and 117 conditions

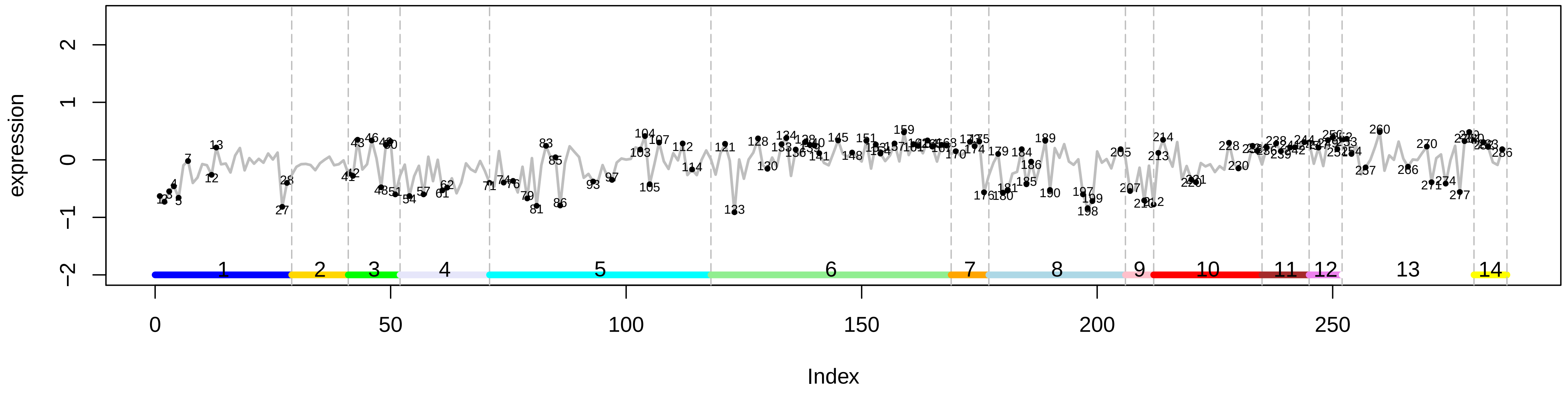

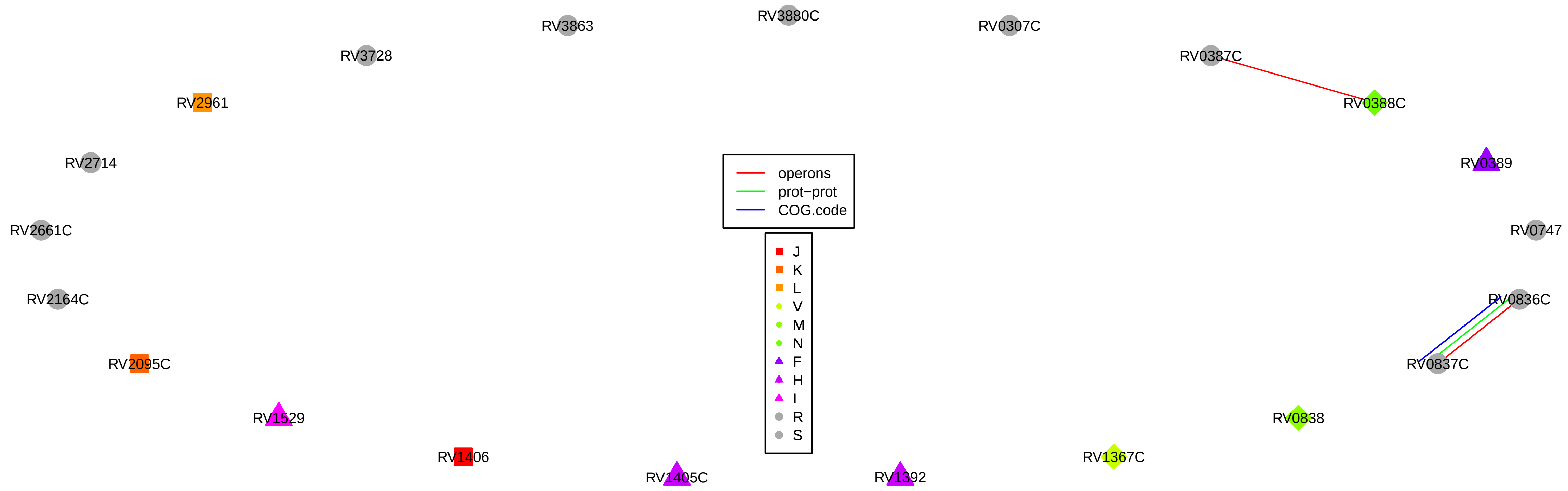

Scaled PSSM #1: E=0.00031

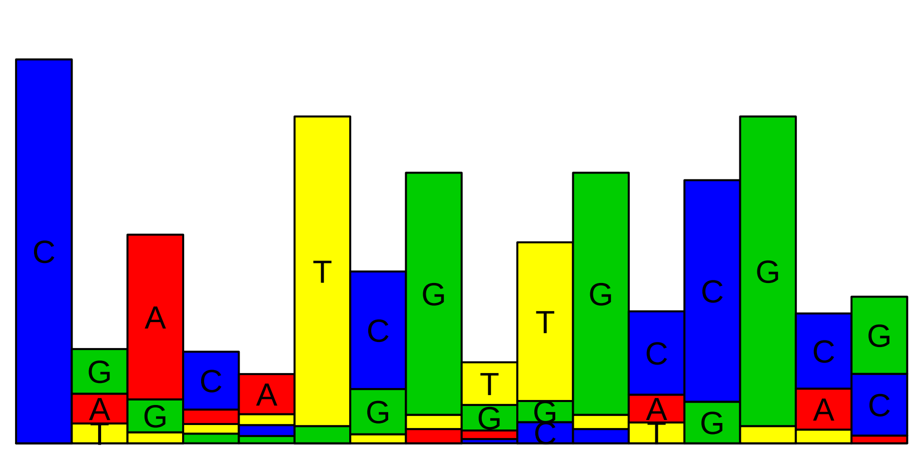

Scaled PSSM #2: E=4e−06

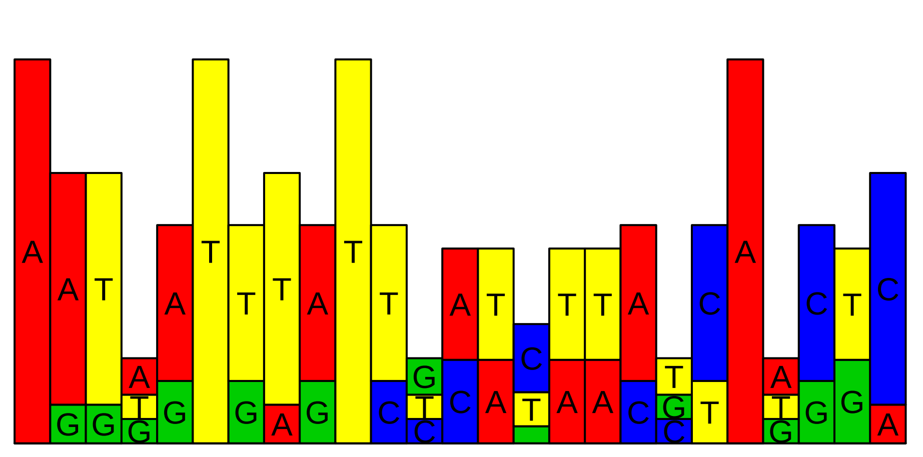

upstream regions

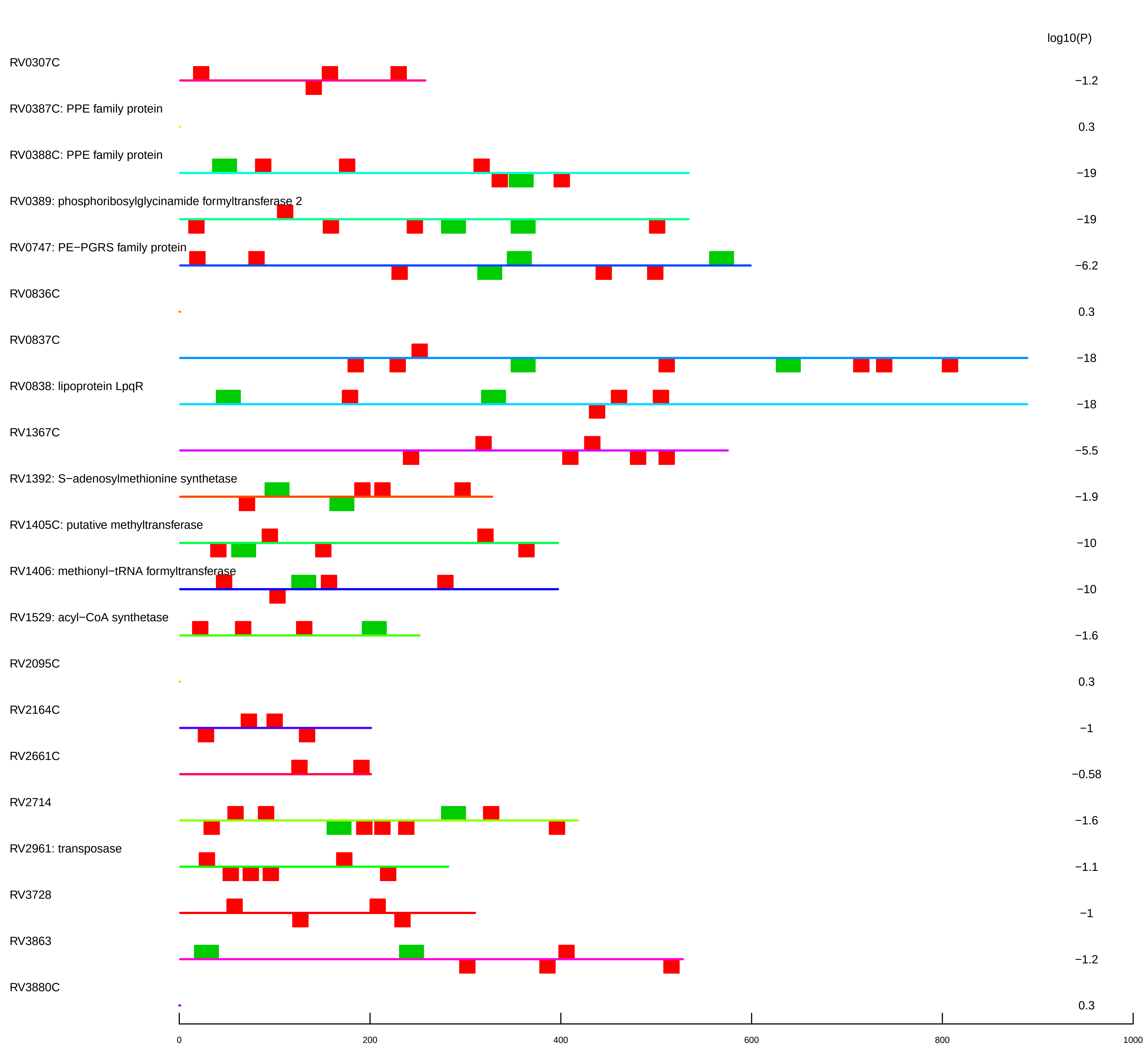

bicluster 8 ; 79 genes and 184 conditions

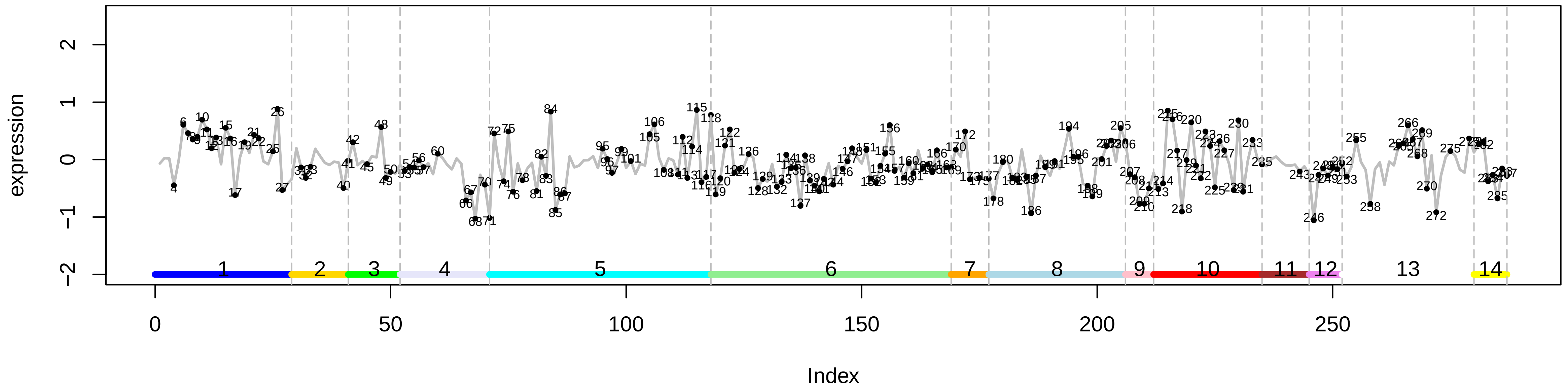

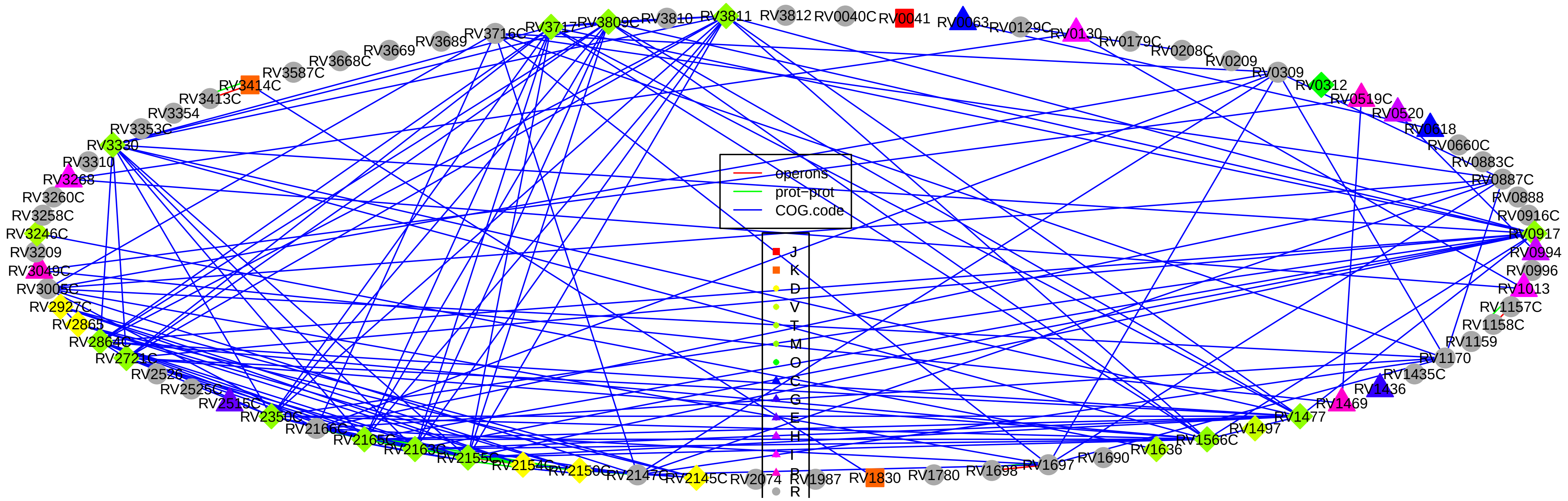

Scaled PSSM #1: E=6.9e−20

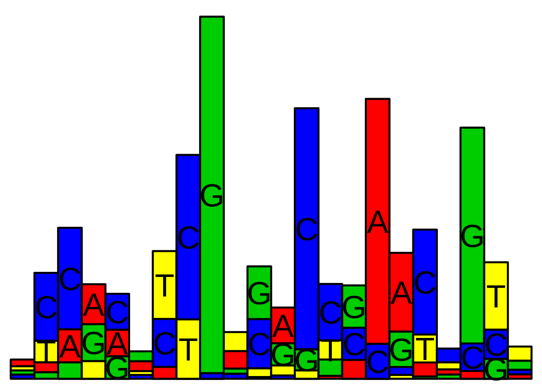

Scaled PSSM #2: E=0.0059

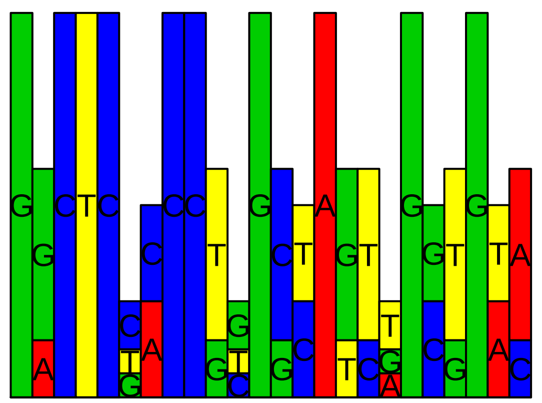

Scaled PSSM #3: E=3.7e−08

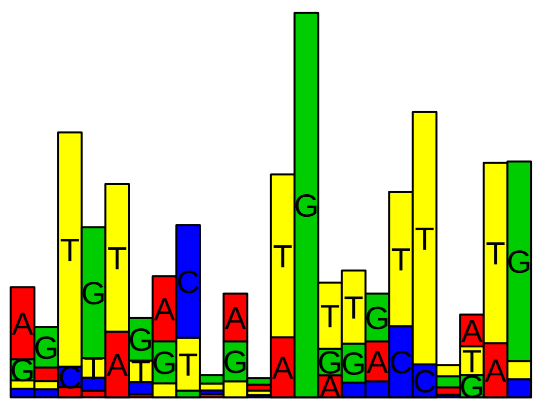

upstream regions

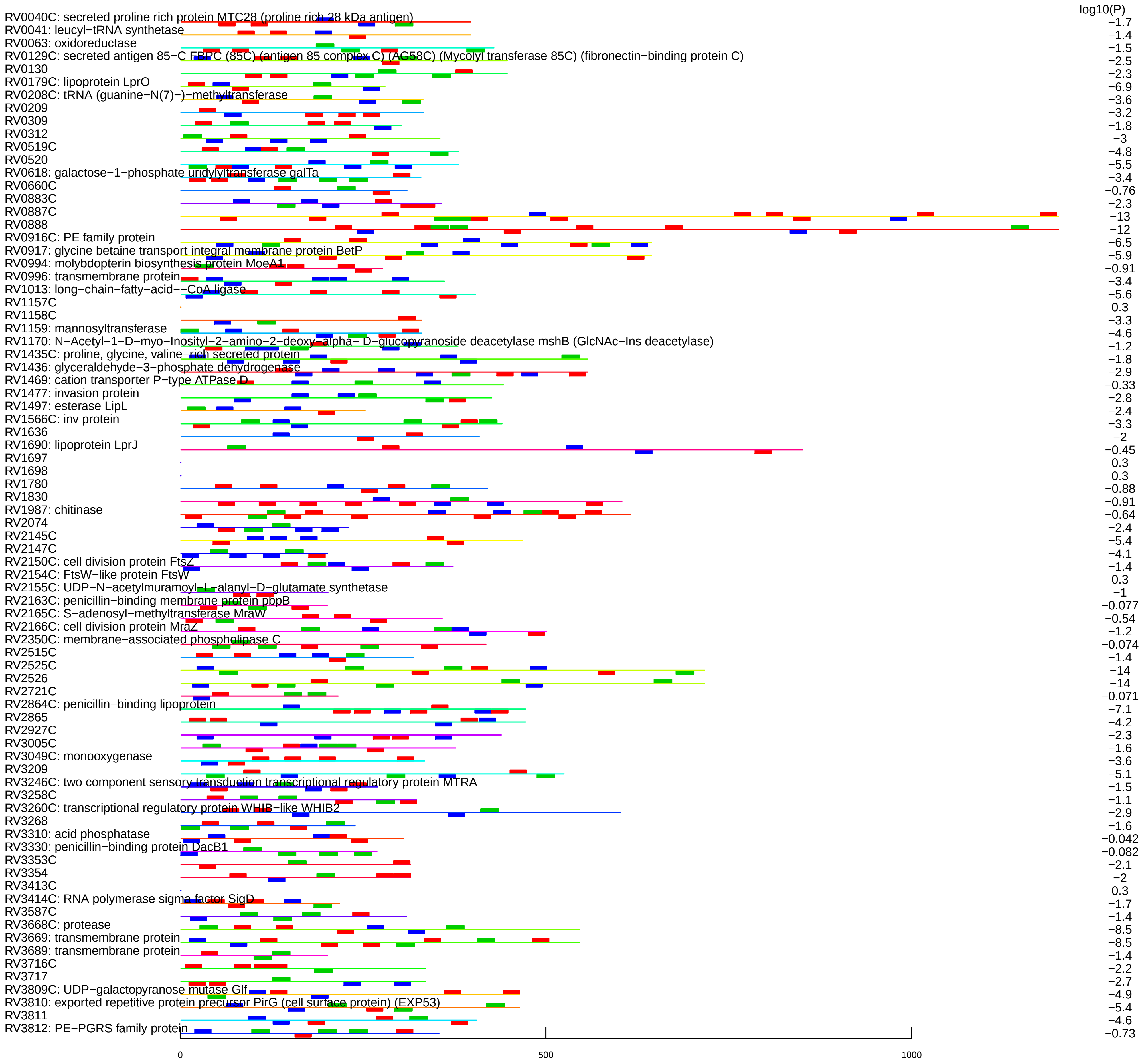

bicluster 9 ; 31 genes and 270 conditions

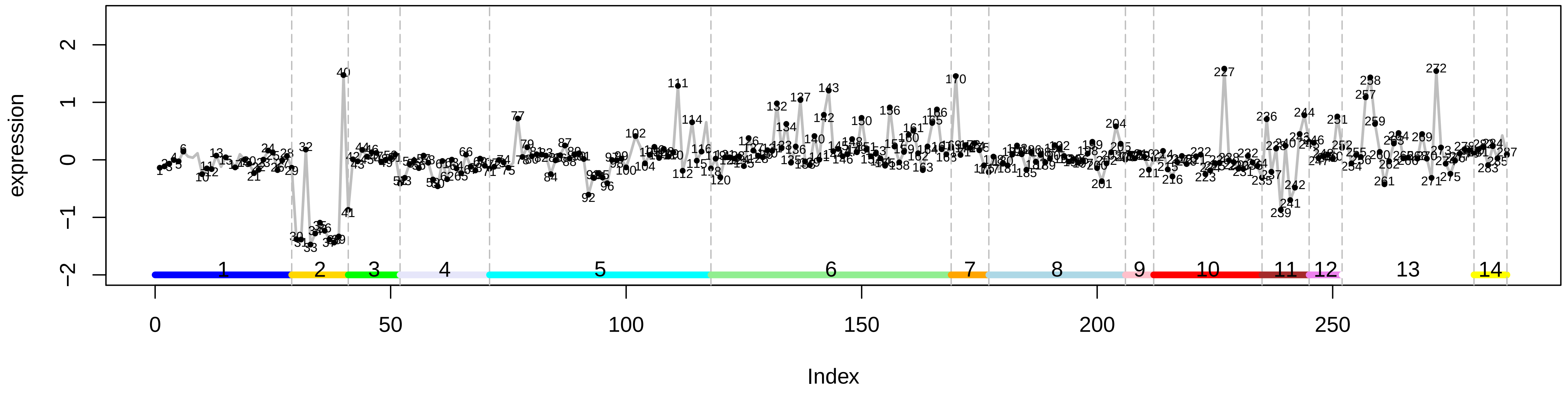

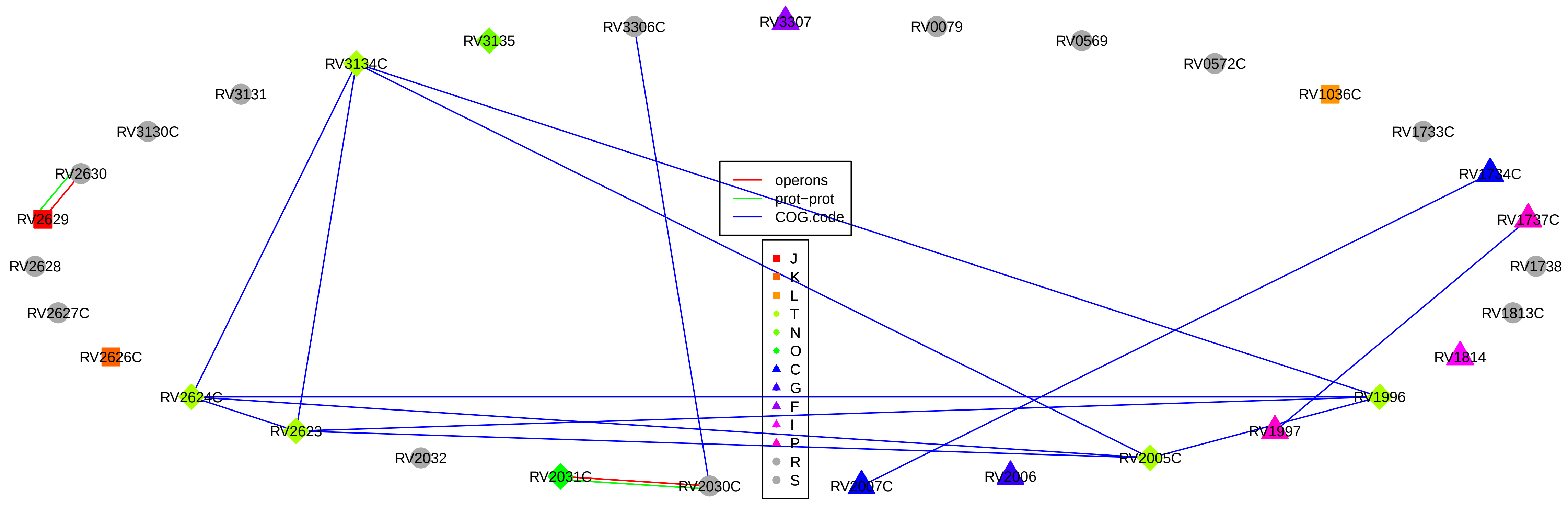

Scaled PSSM #1: E=1.1e−33

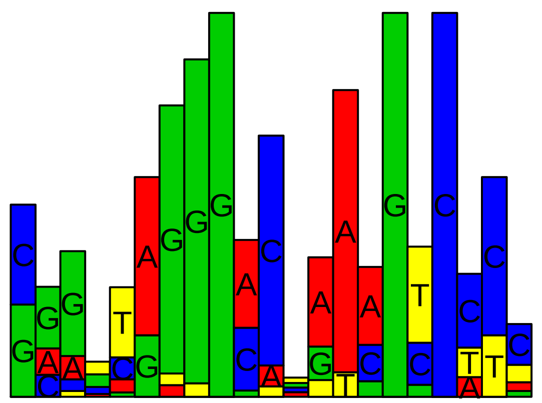

Scaled PSSM #2: E=0.00048

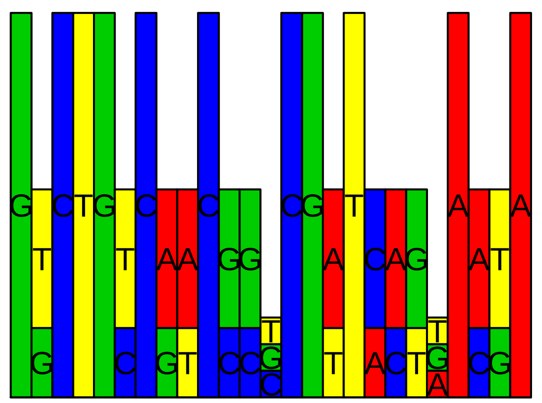

Scaled PSSM #3: E=0.0025

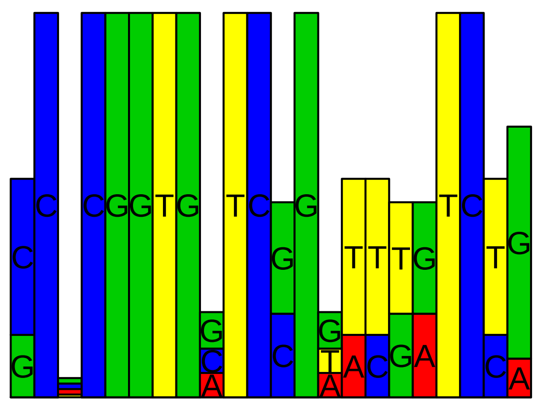

upstream regions

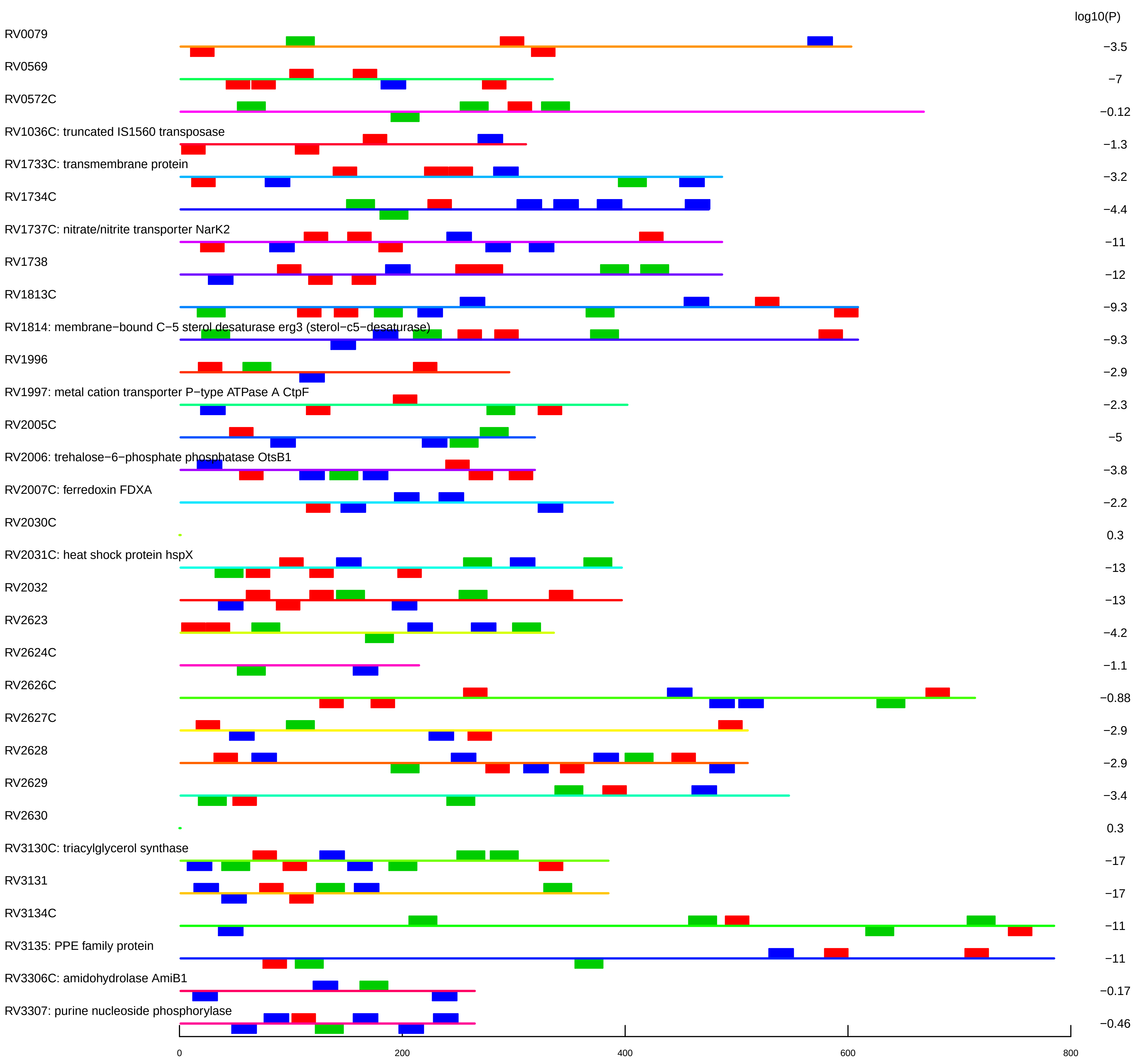

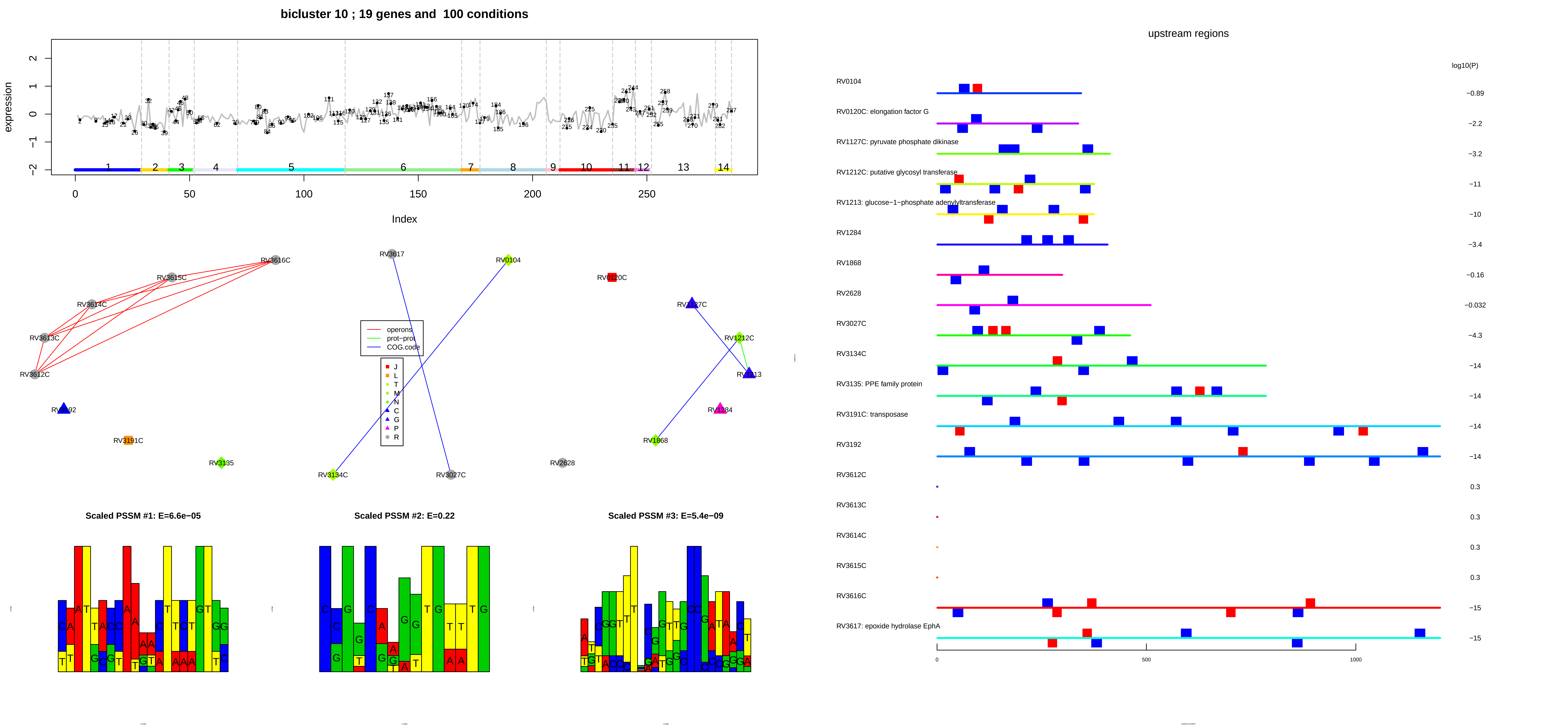
bicluster 10 ; 19 genes and 100 conditions
expression
Index
upstream regions
log10(P)
RV0104 −0.89
RV0120C: elongation factor G −2.2
RV1127C: pyruvate phosphate dikinase −3.2
RV1212C: putative glycosyl transferase −11
RV1213: glucose−1−phosphate adenylyltransferase −10
RV1284 −3.4
RV1868 −0.16
RV2628 −0.032
RV3027C −4.3
RV3134C −14
RV3135: PPE family protein −14
RV3191C: transposase −14
RV3192 −14
RV3612C 0.3
RV3613C 0.3
RV3614C 0.3
RV3615C 0.3
RV3616C −15
RV3617: epoxide hydrolase EphA −15
operons
prot−prot
COG.code
J
L
T
M
N
C
G
P
R
Scaled PSSM #1: E=6.6e−05
Scaled PSSM #2: E=0.22
Scaled PSSM #3: E=5.4e−09

bicluster 11 ; 64 genes and 119 conditions

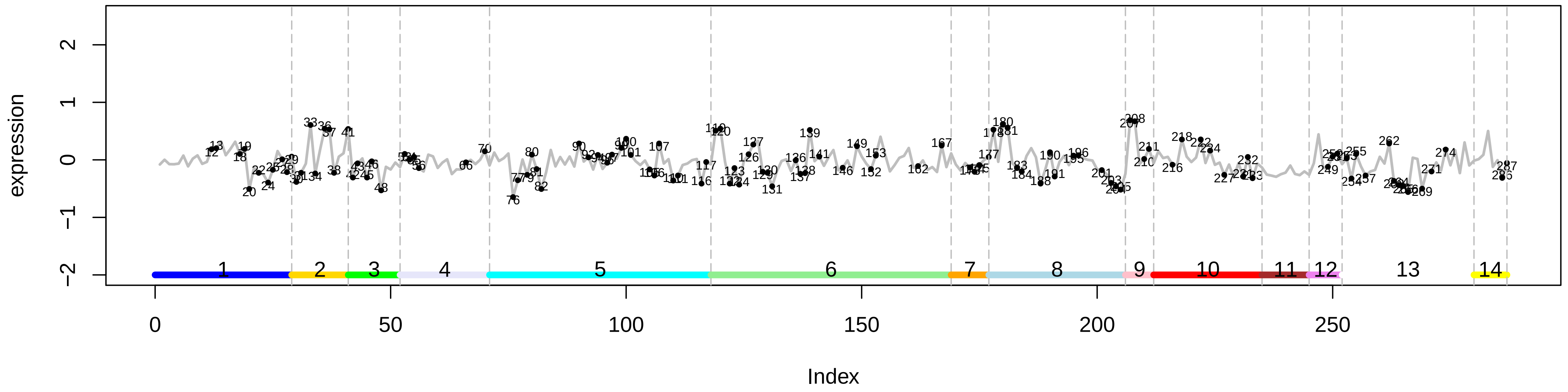

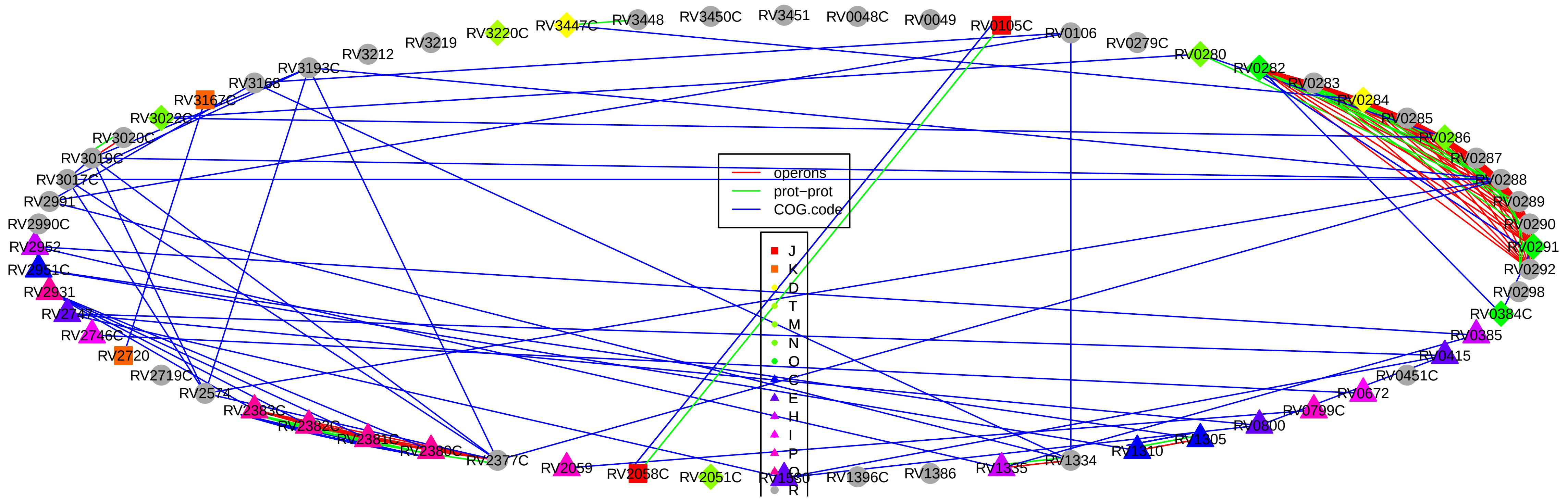

Scaled PSSM #1: E=4.2e−36

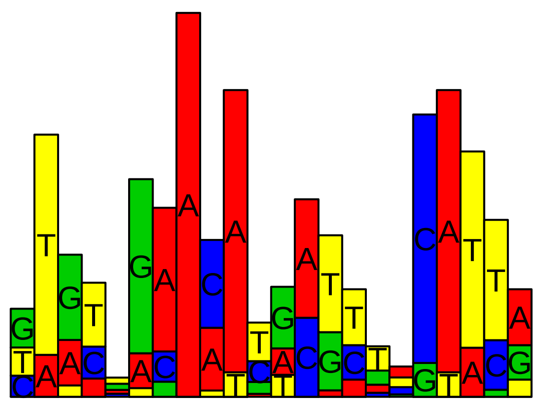

Scaled PSSM #2: E=0.00016

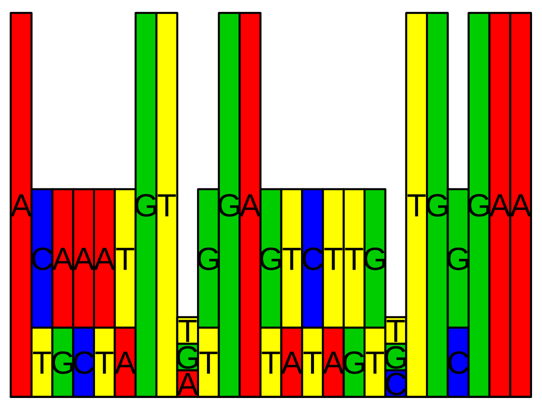

Scaled PSSM #3: E=8e−08

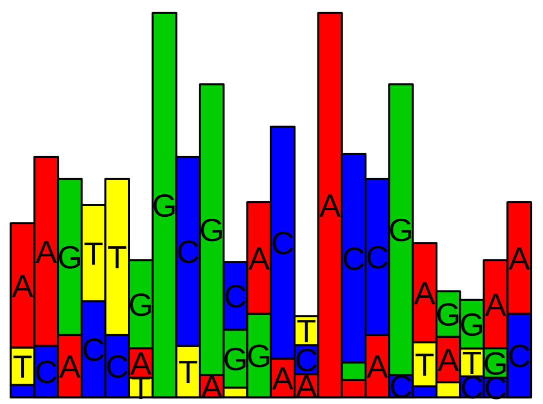

upstream regions

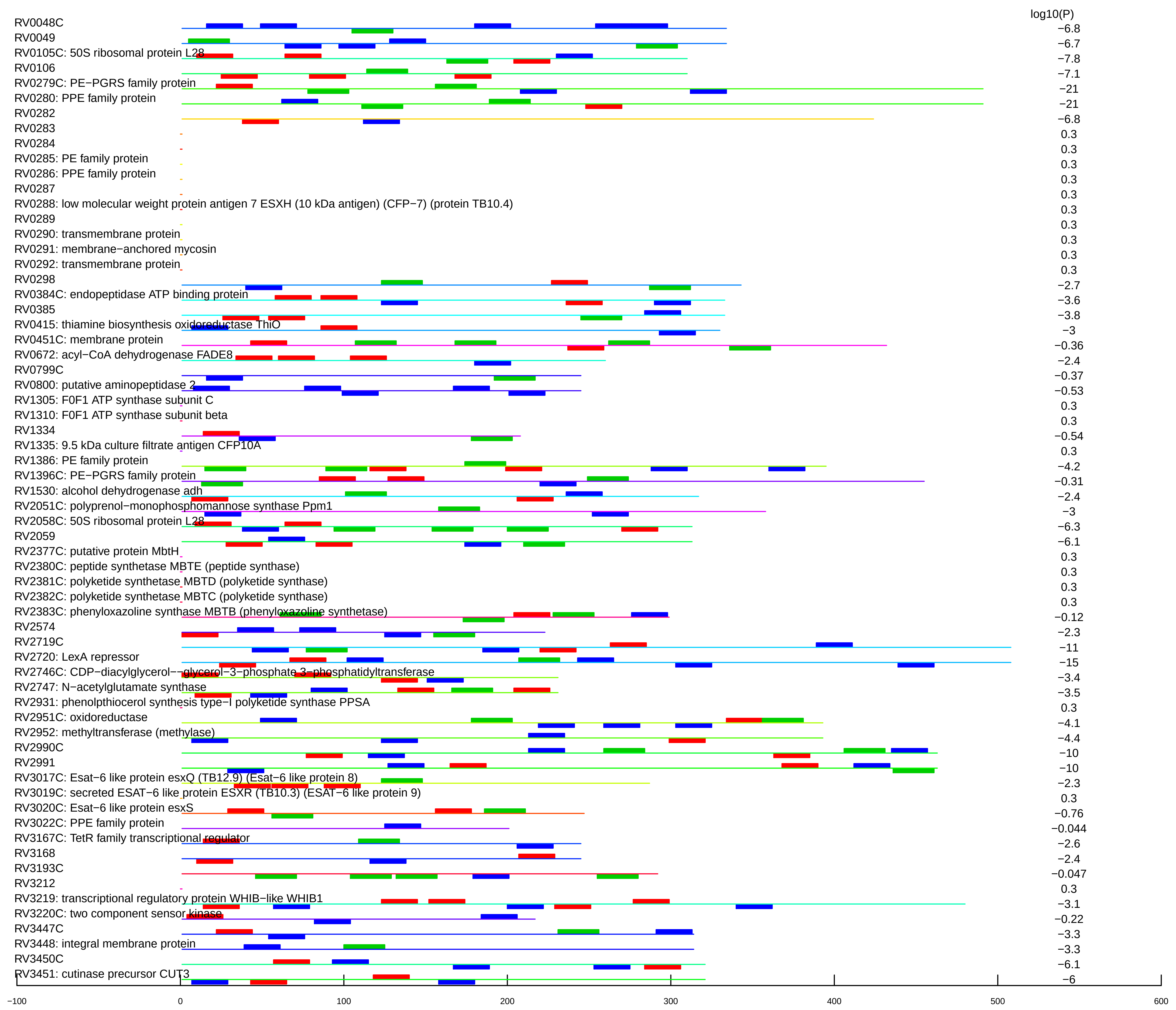

bicluster 12 ; 28 genes and 107 conditions

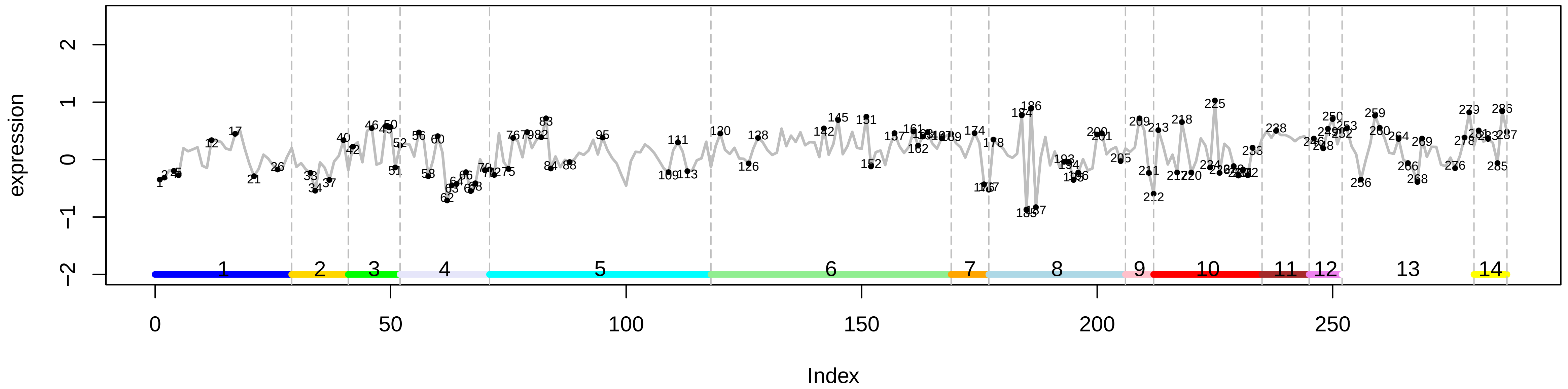

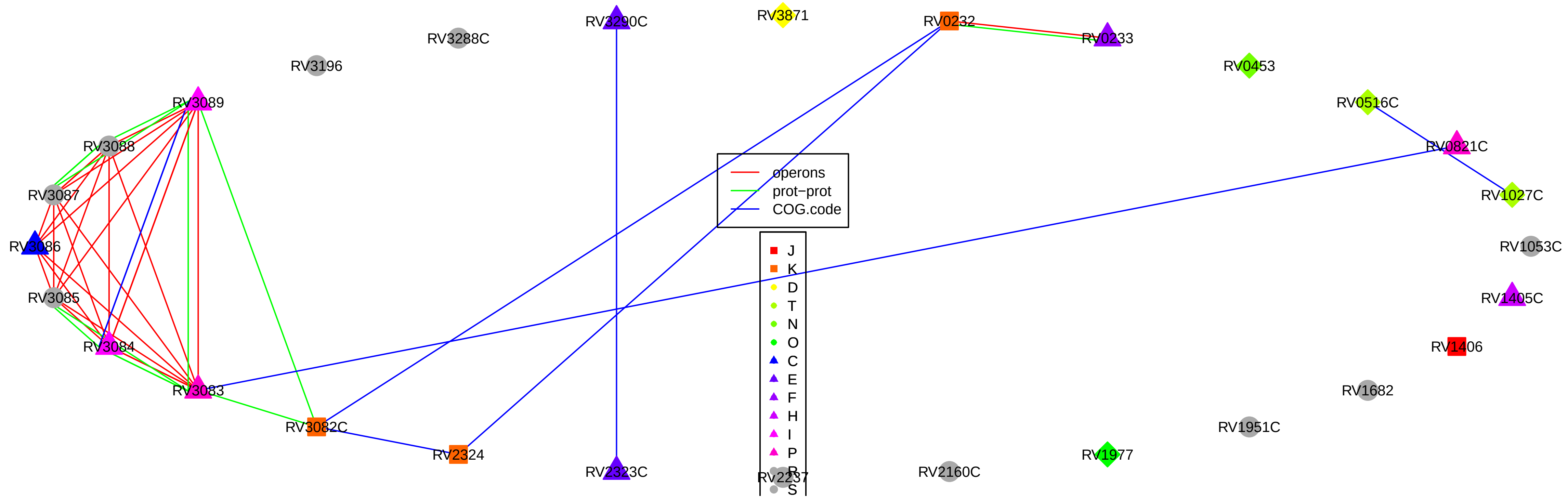

Scaled PSSM #1: E=2.5e−10

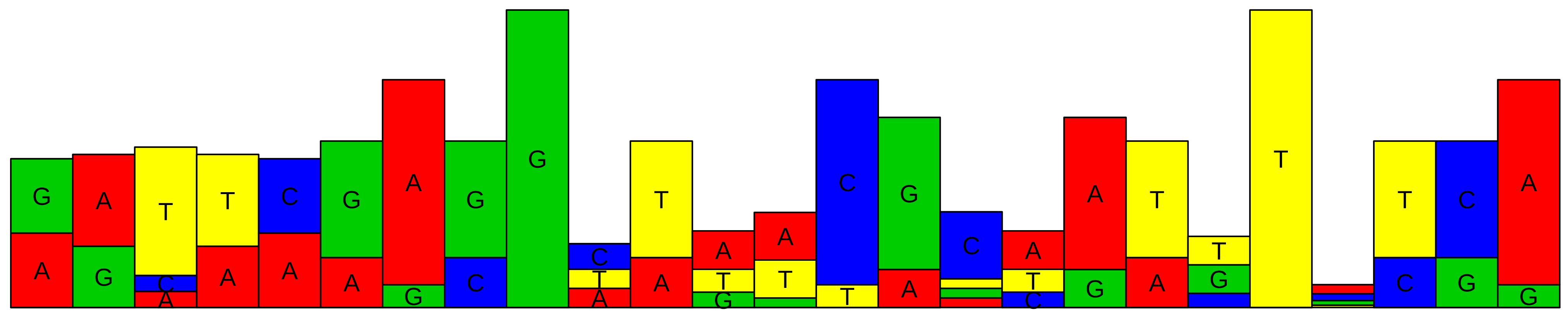

upstream regions

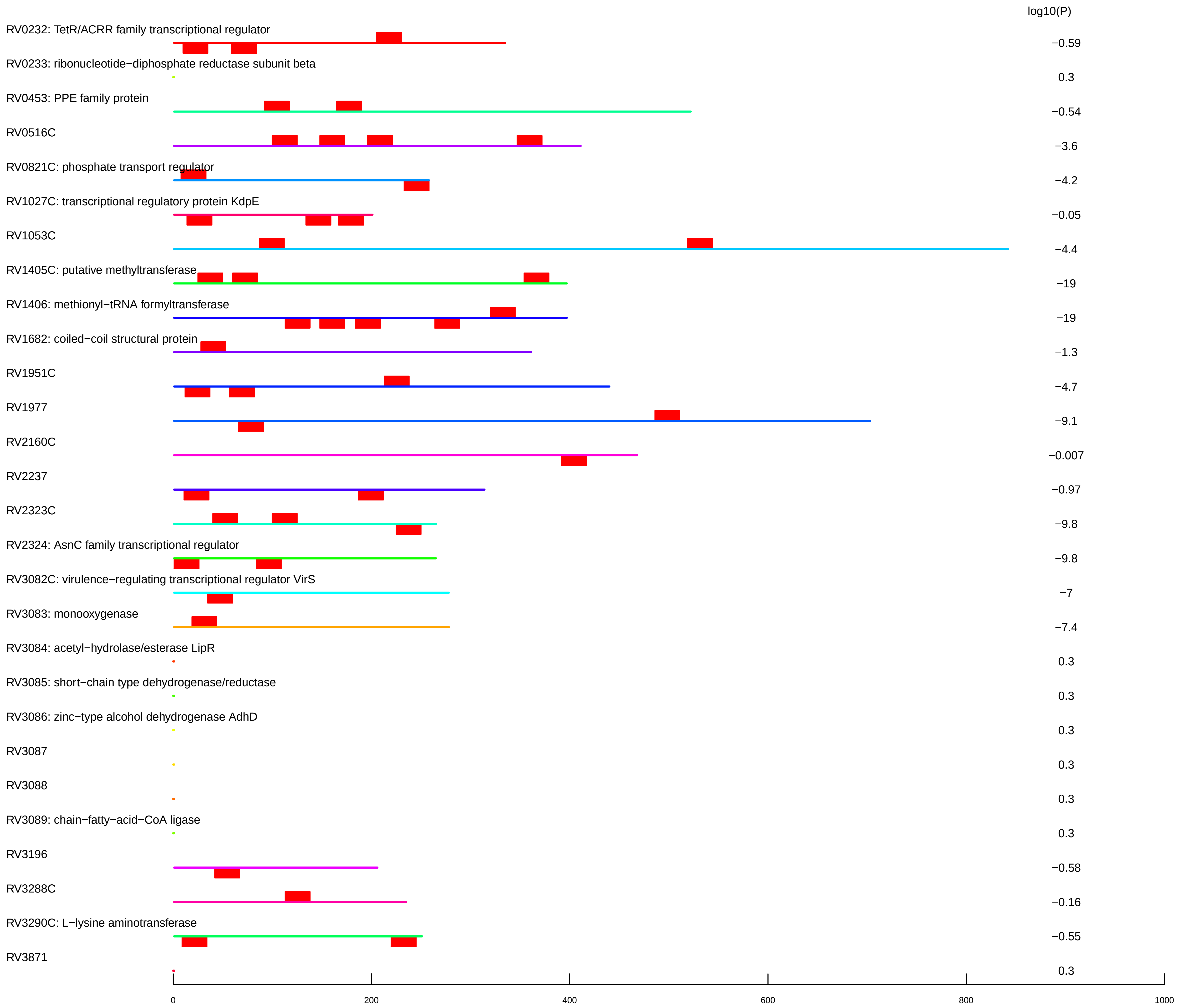

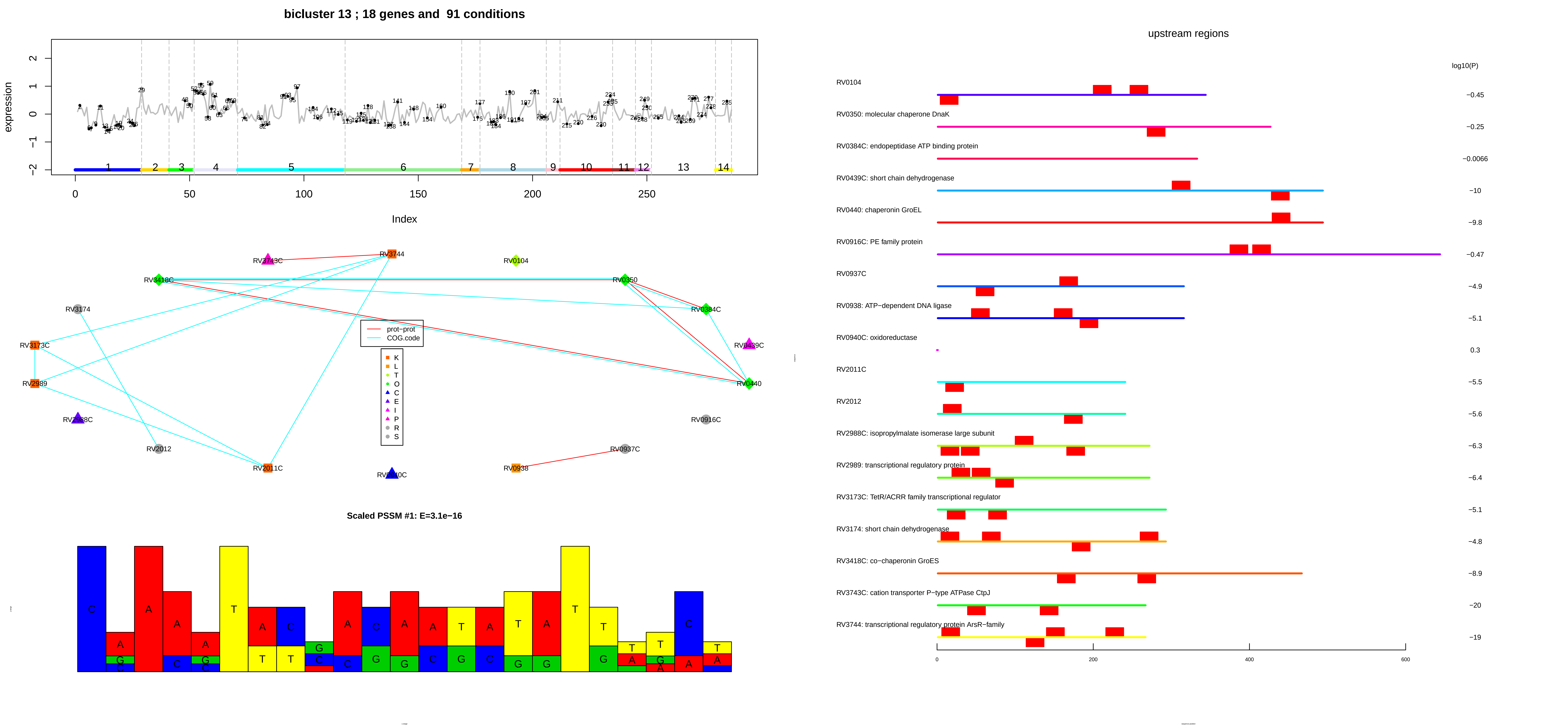

bicluster 13 ; 18 genes and 91 conditions
expression
Index
prot−prot
COG.code
K
L
T
O
C
E
I
P
R
S
RV3743C
RV3744
RV0104
RV3418C
RV0350
RV3174
RV0384C
RV3173C
RV0439C
RV2989
RV0440
RV2988C
RV0916C
RV2012
RV0937C
RV2011C
RV0938
Scaled PSSM #1: E=3.1e−16
upstream regions
log10(P)
RV0104
−0.45
RV0350: molecular chaperone DnaK
−0.25
RV0384C: endopeptidase ATP binding protein
−0.0066
RV0439C: short chain dehydrogenase
−10
RV0440: chaperonin GroEL
−9.8
RV0916C: PE family protein
−0.47
RV0937C
−4.9
RV0938: ATP−dependent DNA ligase
−5.1
RV0940C: oxidoreductase
0.3
RV2011C
−5.5
RV2012
−5.6
RV2988C: isopropylmalate isomerase large subunit
−6.3
RV2989: transcriptional regulatory protein
−6.4
RV3173C: TetR/ACRR family transcriptional regulator
−5.1
RV3174: short chain dehydrogenase
−4.8
RV3418C: co−chaperonin GroES
−8.9
RV3743C: cation transporter P−type ATPase CtpJ
−20
RV3744: transcriptional regulatory protein ArsR−family
−19
0
200
400
600

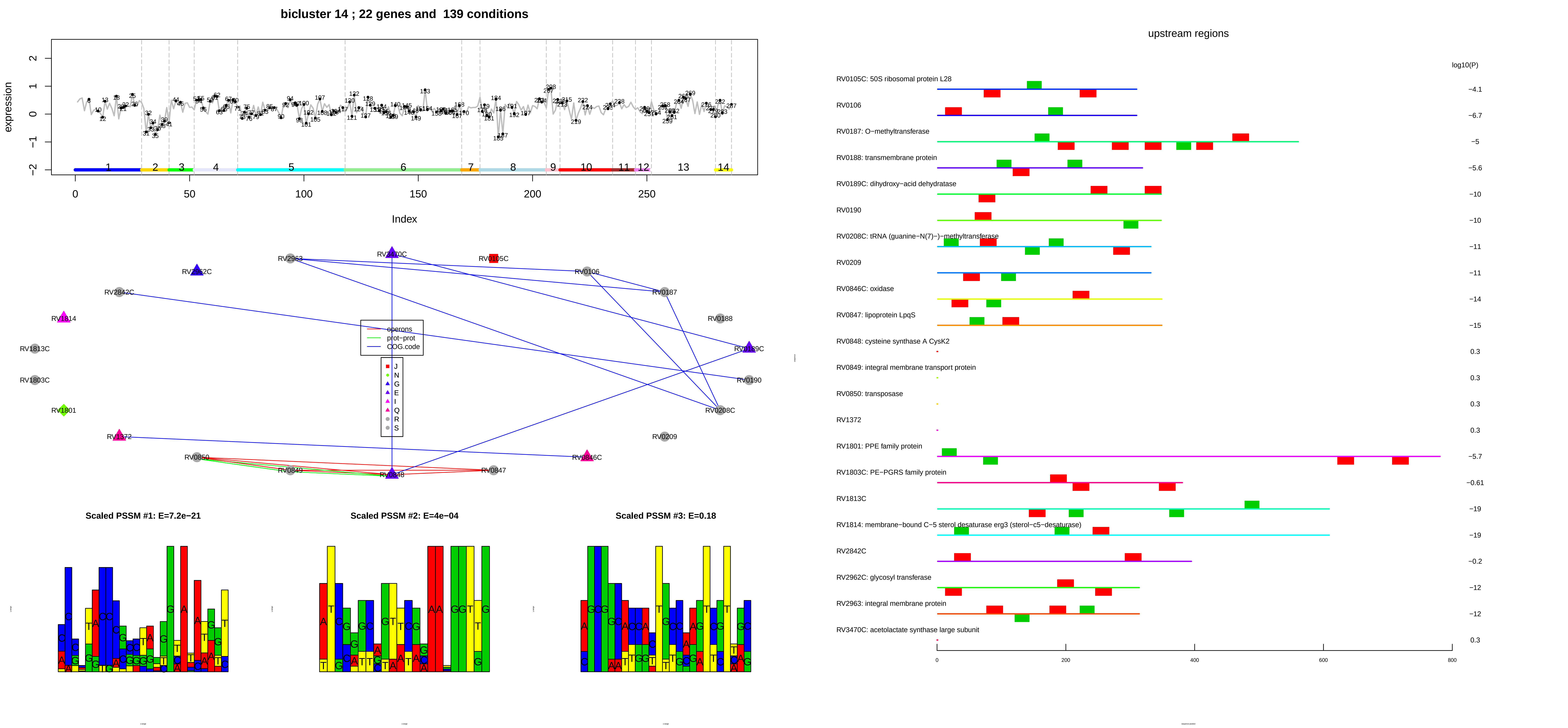

bicluster 14 ; 22 genes and 139 conditions
expression
Index
operons
prot−prot
COG.code
J
N
G
E
I
Q
R
S
Scaled PSSM #1: E=7.2e−21
Scaled PSSM #2: E=4e−04
Scaled PSSM #3: E=0.18
upstream regions
log10(P)
RV0105C: 50S ribosomal protein L28
RV0106
RV0187: O−methyltransferase
RV0188: transmembrane protein
RV0189C: dihydroxy−acid dehydratase
RV0190
RV0208C: tRNA (guanine−N(7)−)−methyltransferase
RV0209
RV0846C: oxidase
RV0847: lipoprotein LpqS
RV0848: cysteine synthase A CysK2
RV0849: integral membrane transport protein
RV0850: transposase
RV1372
RV1801: PPE family protein
RV1803C: PE−PGRS family protein
RV1813C
RV1814: membrane−bound C−5 sterol desaturase erg3 (sterol−c5−desaturase)
RV2842C
RV2962C: glycosyl transferase
RV2963: integral membrane protein
RV3470C: acetolactate synthase large subunit

bicluster 15 ; 14 genes and 116 conditions

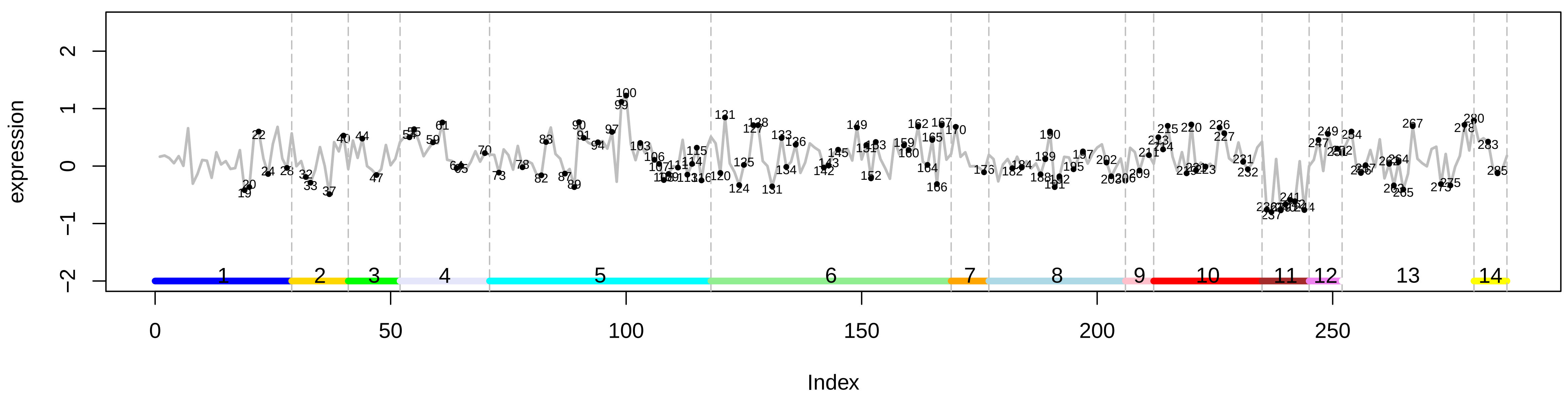

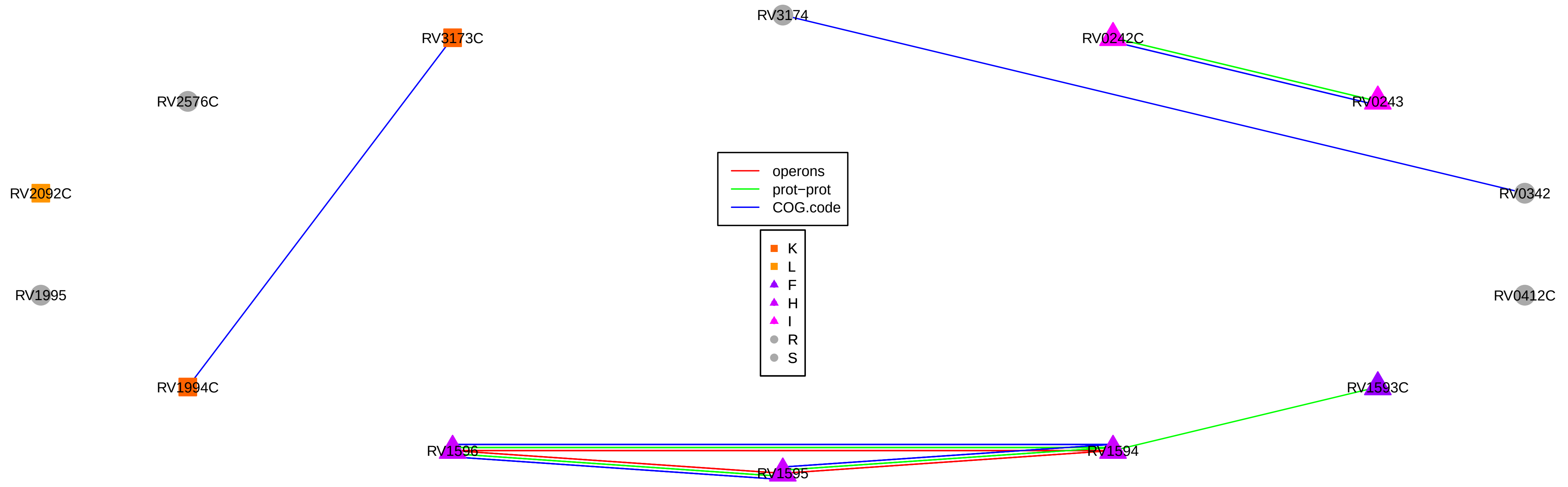

Scaled PSSM #1: E=9.2e−10

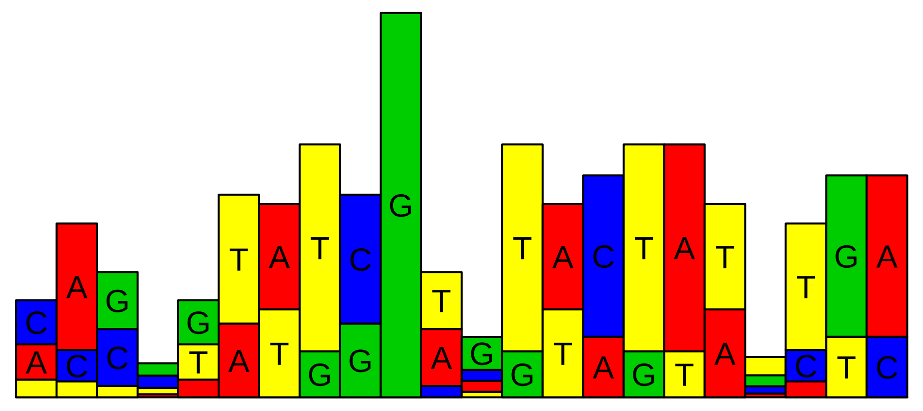

Scaled PSSM #2: E=0.071

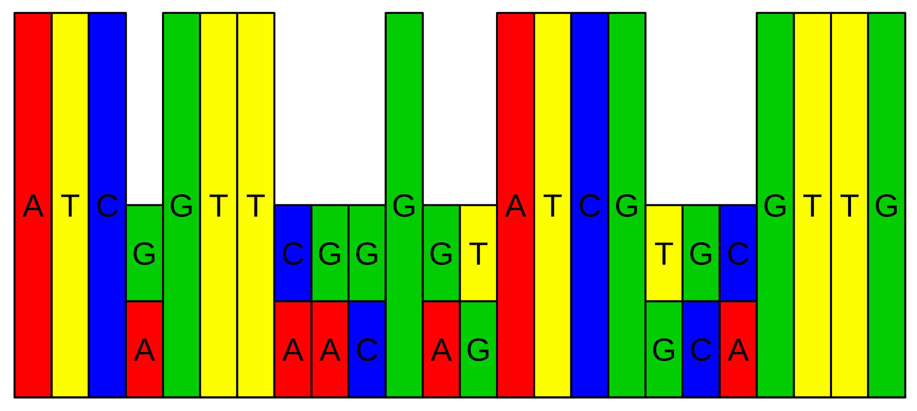

upstream regions

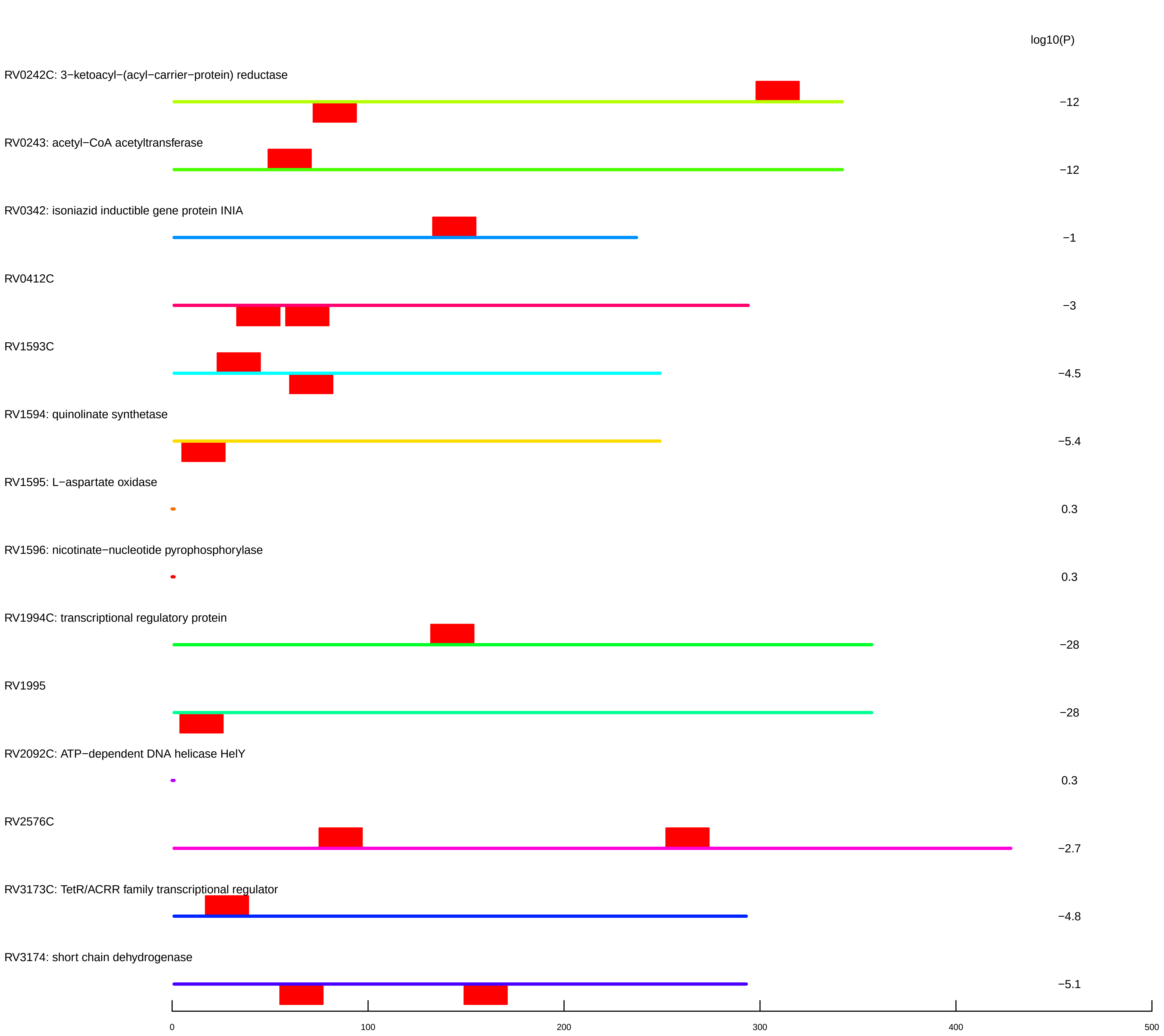

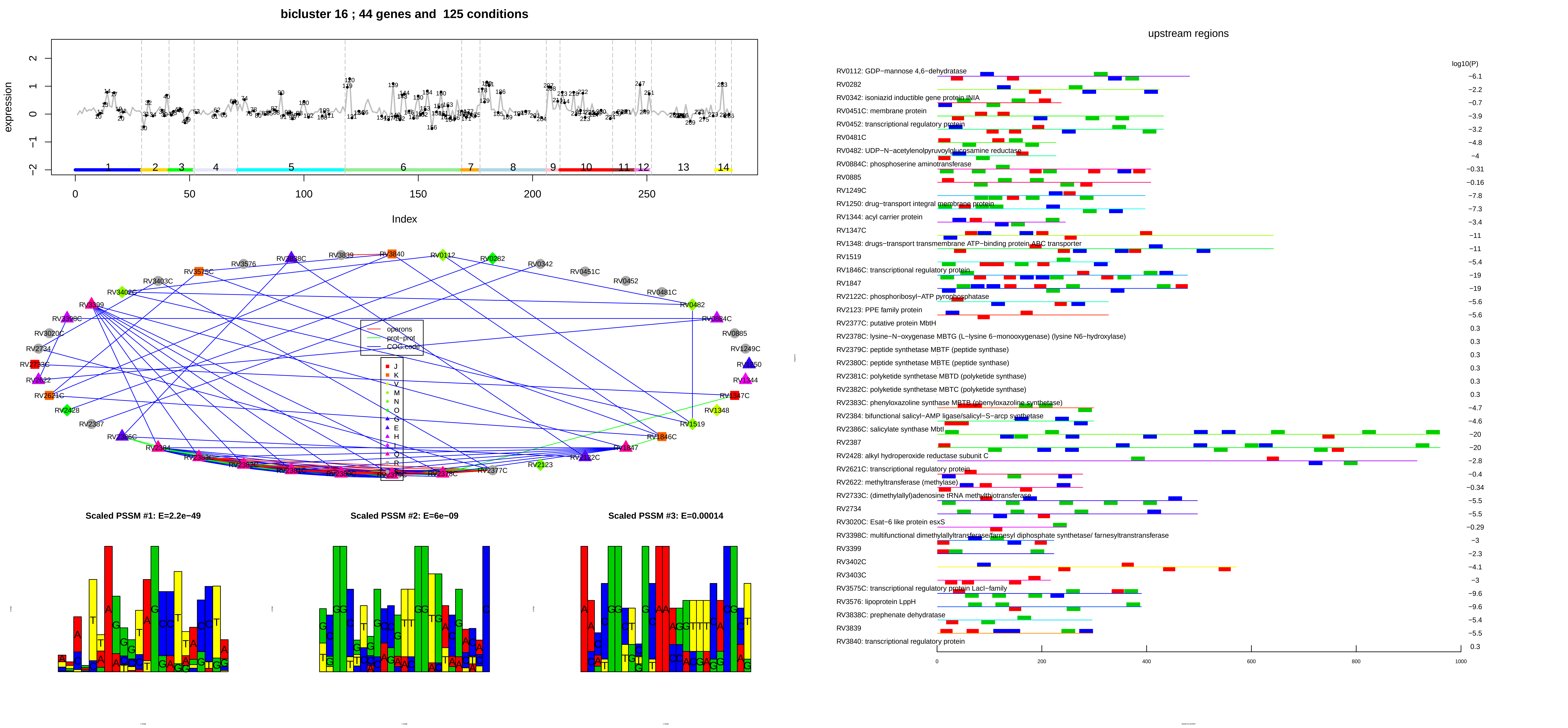

bicluster 16 ; 44 genes and 125 conditions
expression
Index
operons
prot−prot
COG.code
Scaled PSSM #1: E=2.2e−49
Scaled PSSM #2: E=6e−09
Scaled PSSM #3: E=0.00014
upstream regions
log10(P)
RV0112: GDP−mannose 4,6−dehydratase
RV0282
RV0342: isoniazid inductible gene protein INIA
RV0451C: membrane protein
RV0452: transcriptional regulatory protein
RV0481C
RV0482: UDP−N−acetylenolpyruvoylglucosamine reductase
RV0884C: phosphoserine aminotransferase
RV0885
RV1249C
RV1250: drug−transport integral membrane protein
RV1344: acyl carrier protein
RV1347C
RV1348: drugs−transport transmembrane ATP−binding protein ABC transporter
RV1519
RV1846C: transcriptional regulatory protein
RV1847
RV2122C: phosphoribosyl−ATP pyrophosphatase
RV2123: PPE family protein
RV2377C: putative protein MbtH
RV2378C: lysine−N−oxygenase MBTG (L−lysine 6−monooxygenase) (lysine N6−hydroxylase)
RV2379C: peptide synthetase MBTF (peptide synthase)
RV2380C: peptide synthetase MBTE (peptide synthase)
RV2381C: polyketide synthetase MBTD (polyketide synthase)
RV2382C: polyketide synthetase MBTC (polyketide synthase)
RV2383C: phenyloxazoline synthase MBTB (phenyloxazoline synthetase)
RV2384: bifunctional salicyl−AMP ligase/salicyl−S−arcp synthetase
RV2386C: salicylate synthase MbtI
RV2387
RV2428: alkyl hydroperoxide reductase subunit C
RV2621C: transcriptional regulatory protein
RV2622: methyltransferase (methylase)
RV2733C: (dimethylallyl)adenosine tRNA methylthiotransferase
RV2734
RV3020C: Esat−6 like protein esxS
RV3398C: multifunctional dimethylallyltransferase/farnesyl diphosphate synthetase/ farnesyltranstransferase
RV3399
RV3402C
RV3403C
RV3575C: transcriptional regulatory protein LacI−family
RV3576: lipoprotein LppH
RV3838C: prephenate dehydratase
RV3839
RV3840: transcriptional regulatory protein
−6.1
−2.2
−0.7
−3.9
−3.2
−4.8
−4
−0.31
−0.16
−7.8
−7.3
−3.4
−11
−11
−5.4
−19
−19
−5.6
−5.6
0.3
0.3
0.3
0.3
0.3
0.3
0.3
−4.7
−4.6
−20
−20
−2.8
−0.4
−0.34
−5.5
−5.5
−0.29
−3
−2.3
−4.1
−3
−9.6
−9.6
−5.4
−5.5
0.3

bicluster 17 ; 10 genes and 126 conditions

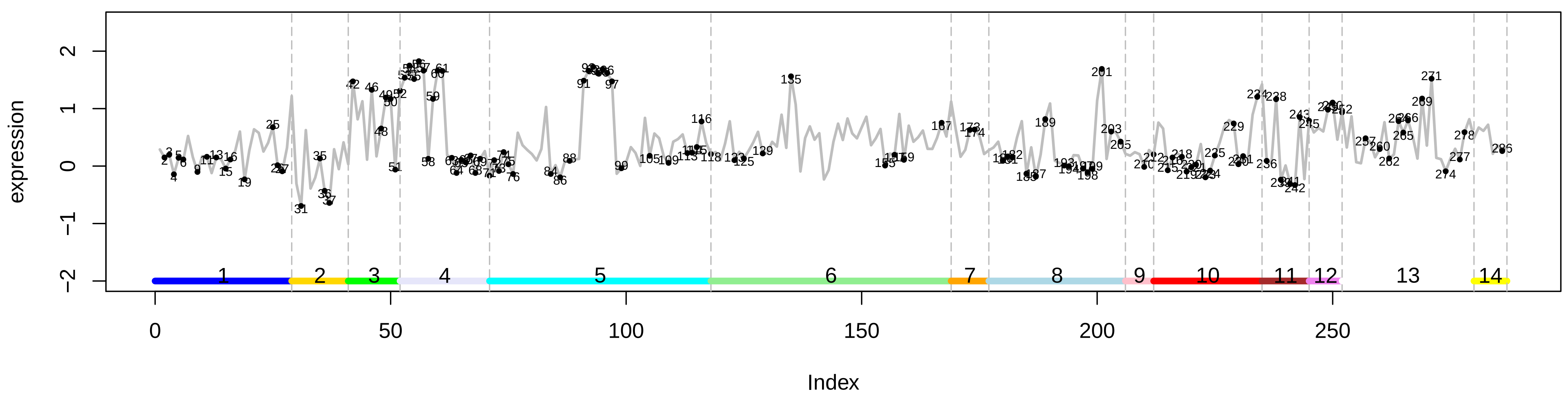

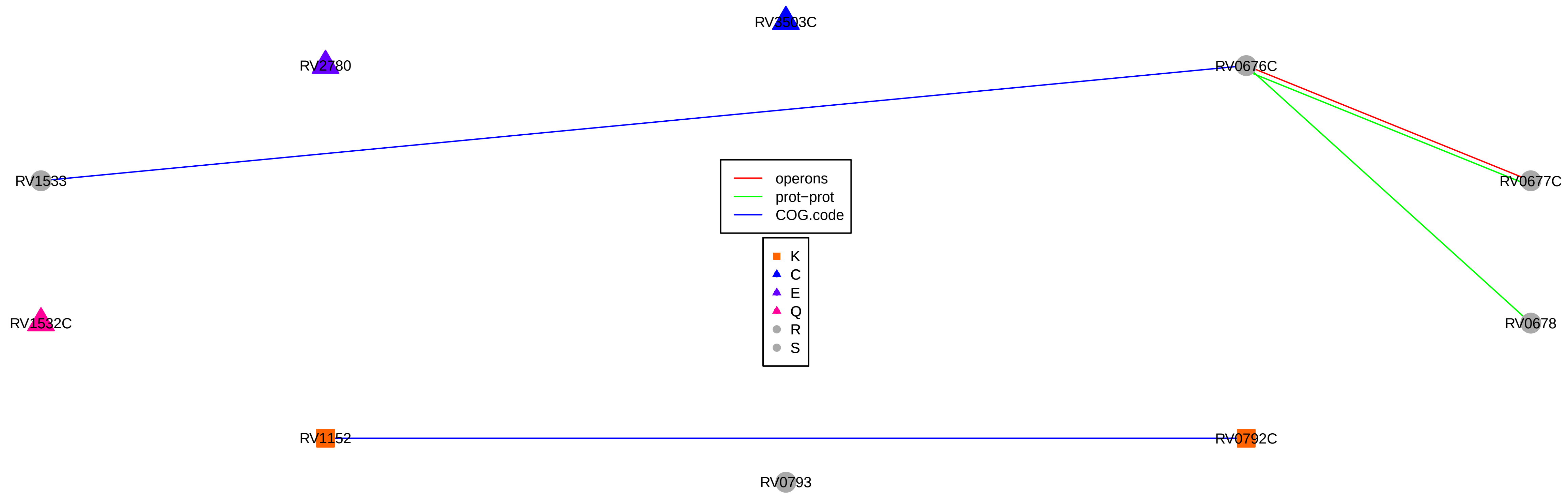

Scaled PSSM #1: E=1.9e−08

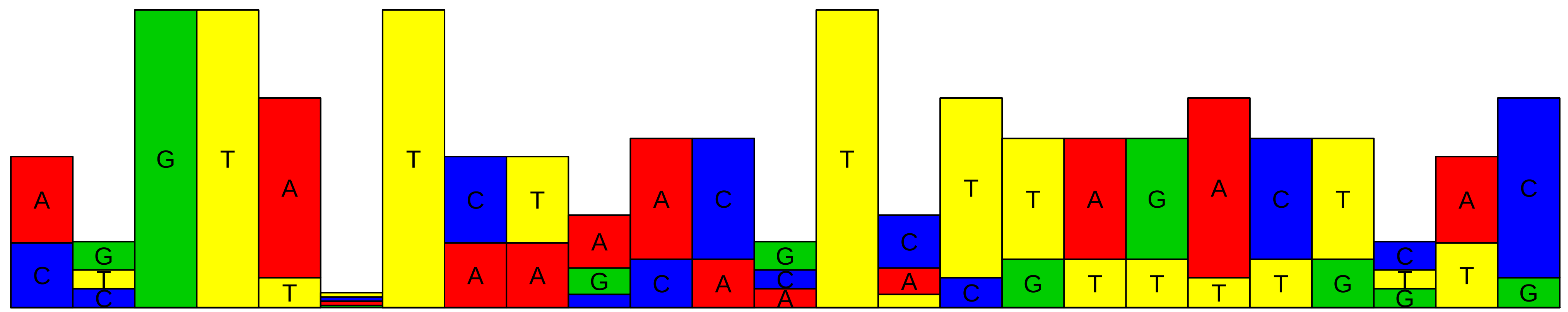

upstream regions

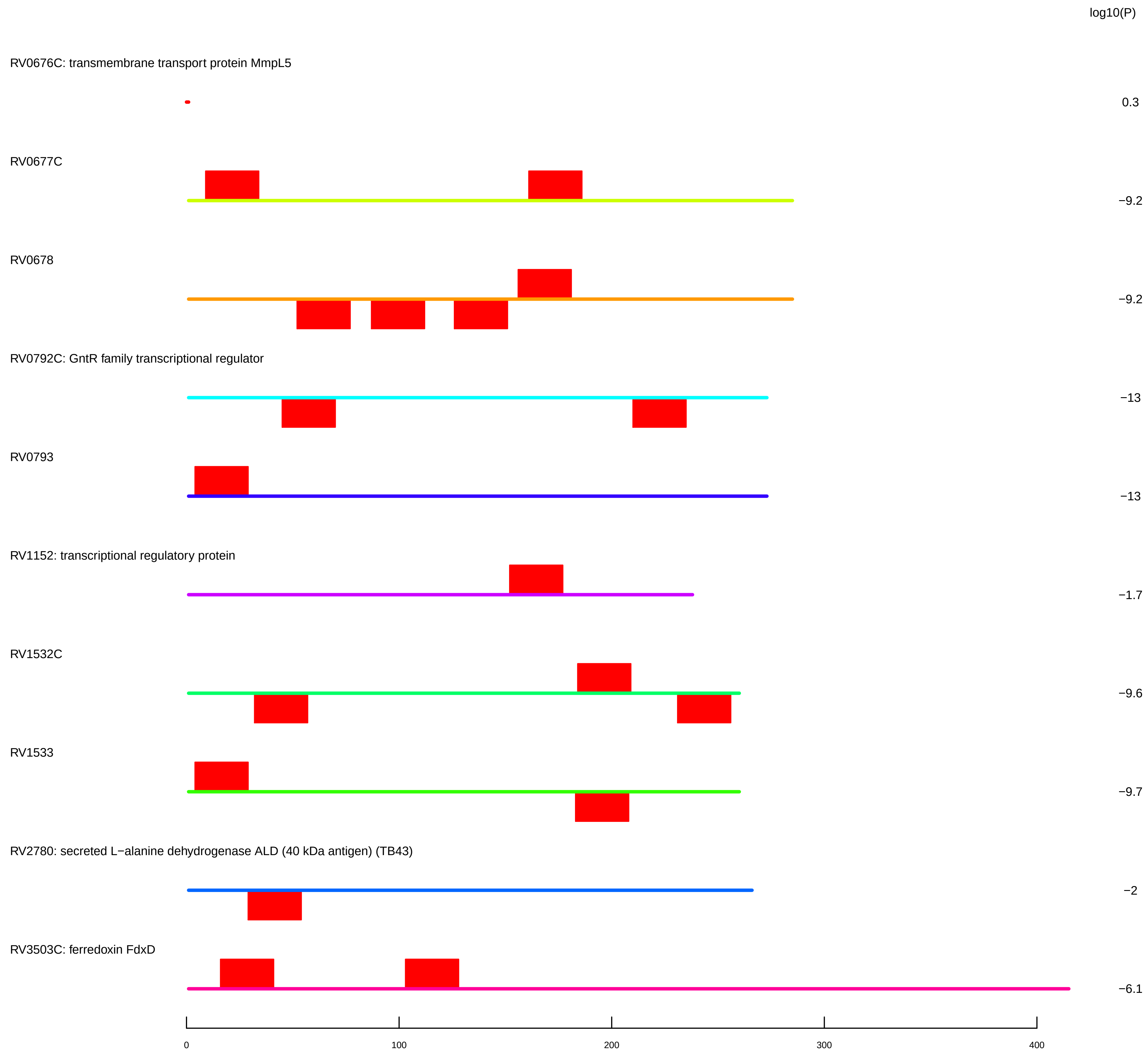

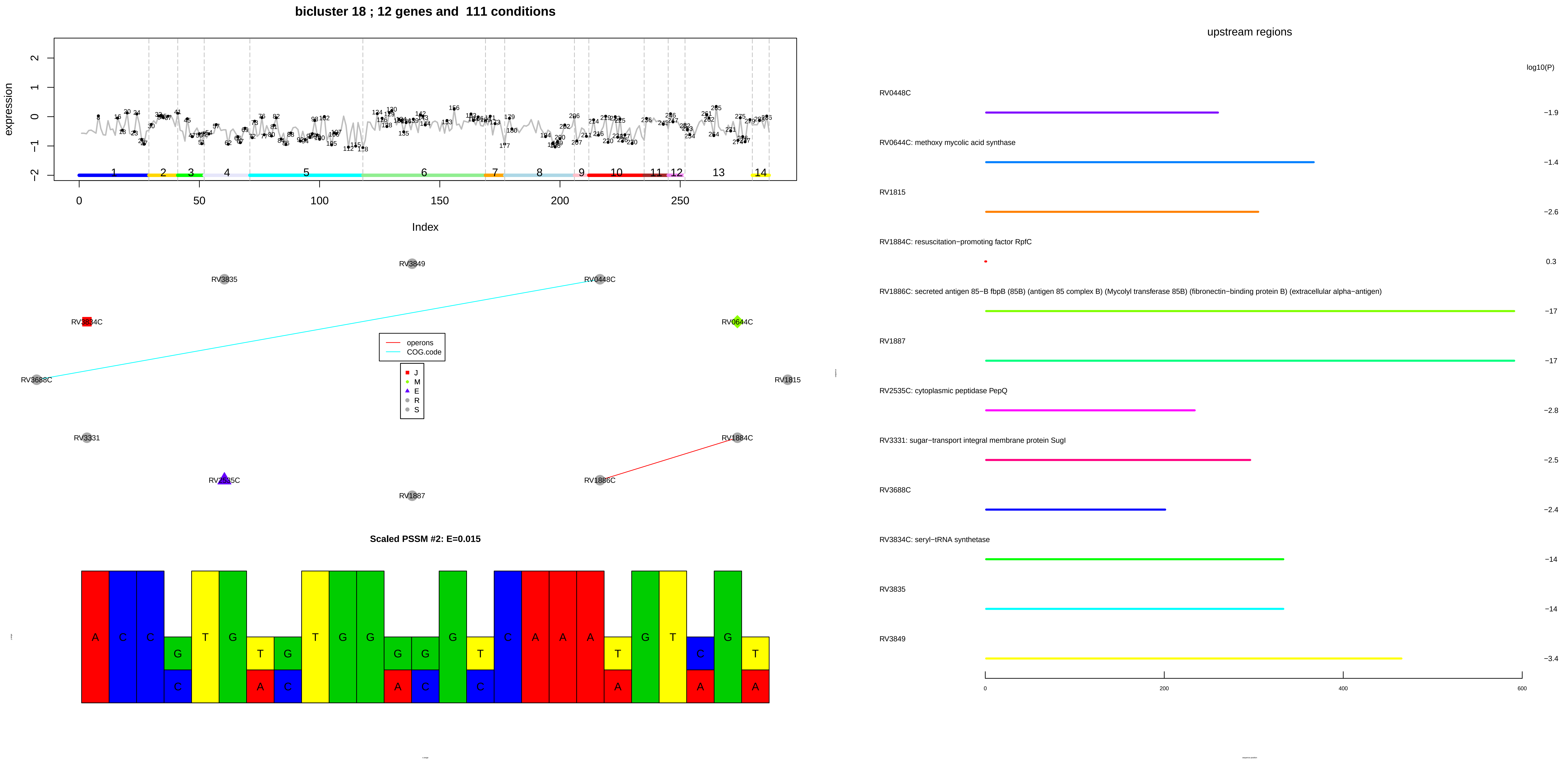
bicluster 18 ; 12 genes and 111 conditions
expression
Index
operons
COG.code
J
M
E
R
S
RV3849
RV3835
RV0448C
RV3834C
RV0644C
RV3688C
RV1815
RV3331
RV1884C
RV2535C
RV1886C
RV1887
Scaled PSSM #2: E=0.015
upstream regions
log10(P)
RV0448C
-1.9
RV0644C: methoxy mycolic acid synthase
-1.4
RV1815
-2.6
RV1884C: resuscitation-promoting factor RpfC
0.3
RV1886C: secreted antigen 85-B fbpB (85B) (antigen 85 complex B) (Mycolyl transferase 85B) (fibronectin-binding protein B) (extracellular alpha-antigen)
-17
RV1887
-17
RV2535C: cytoplasmic peptidase PepQ
-2.8
RV3331: sugar-transport integral membrane protein SugI
-2.5
RV3688C
-2.4
RV3834C: seryl-tRNA synthetase
-14
RV3835
-14
RV3849
-3.4

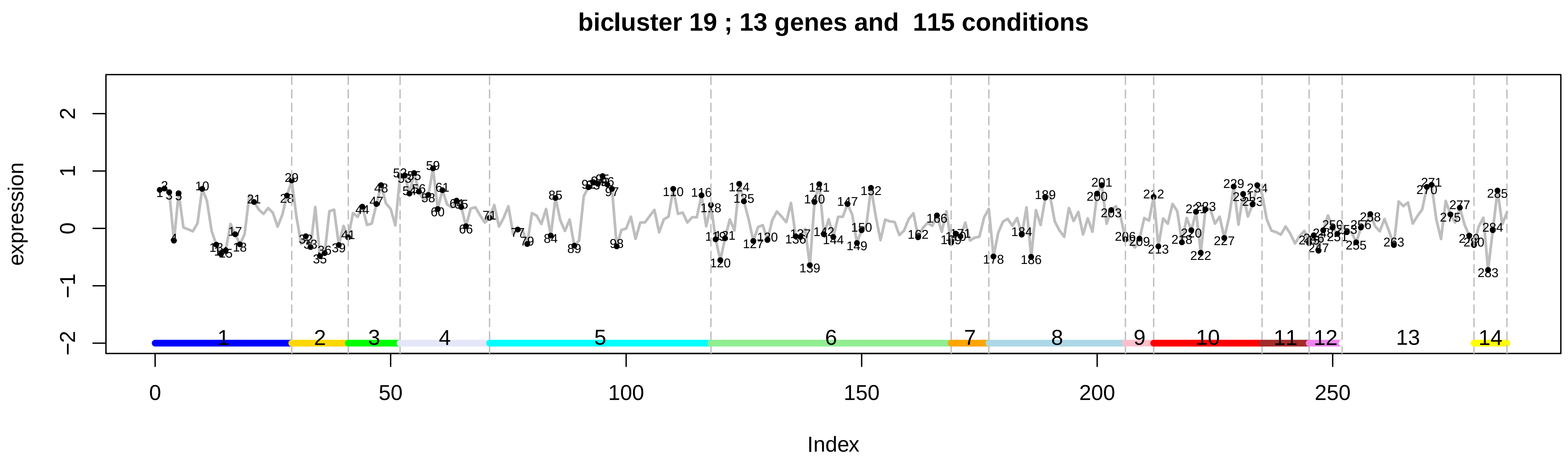
bicluster 19 ; 13 genes and 115 conditions
expression
Index

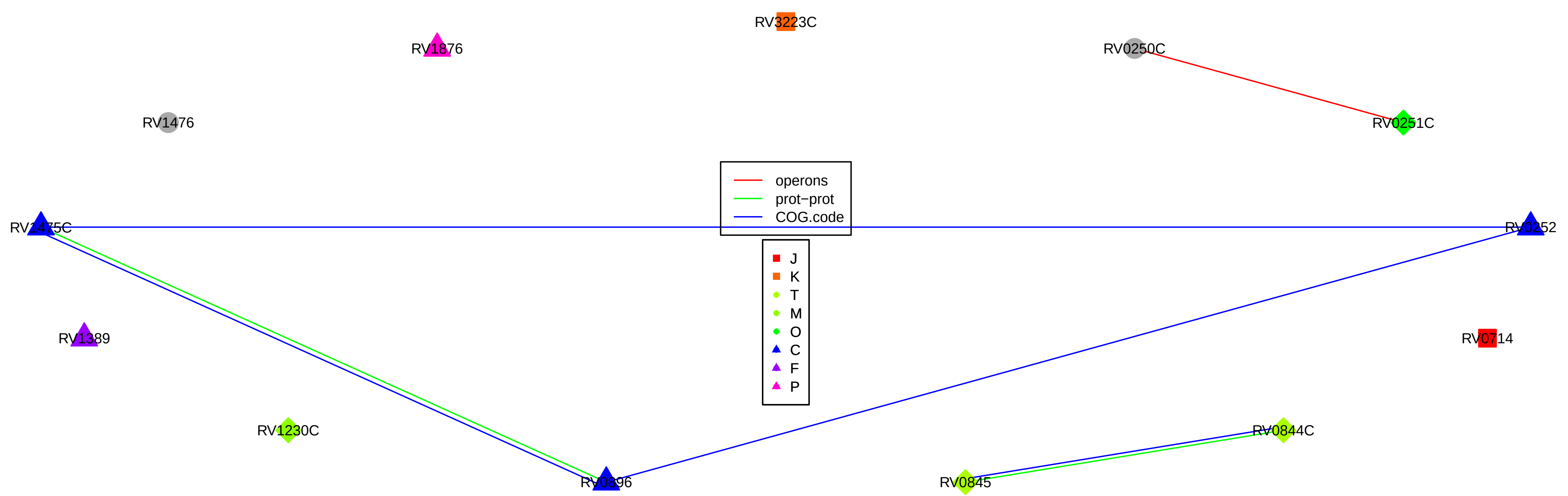
RV3223C
RV1876
RV0250C
RV1476
RV0251C
operons
prot−prot
COG.code
J
K
T
M
O
C
F
P
RV1389
RV0714
RV1230C
RV0844C
RV0845

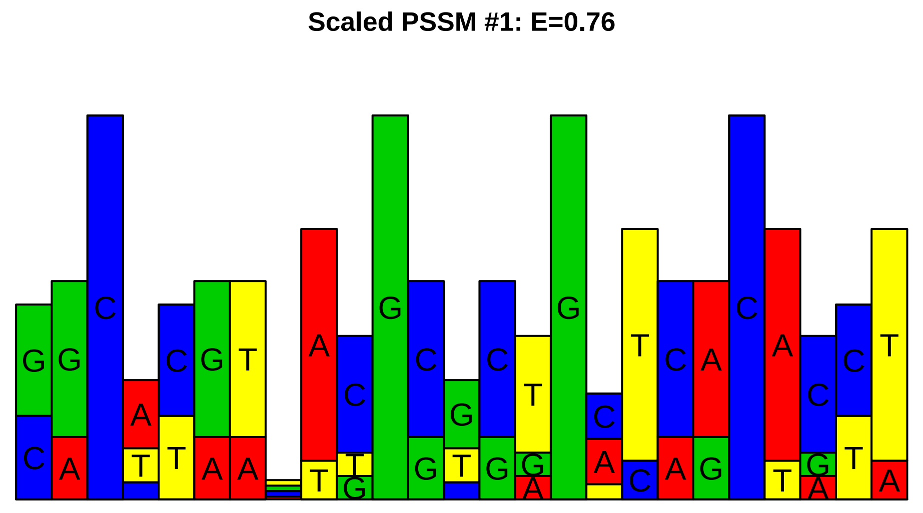
Scaled PSSM #1: E=0.76

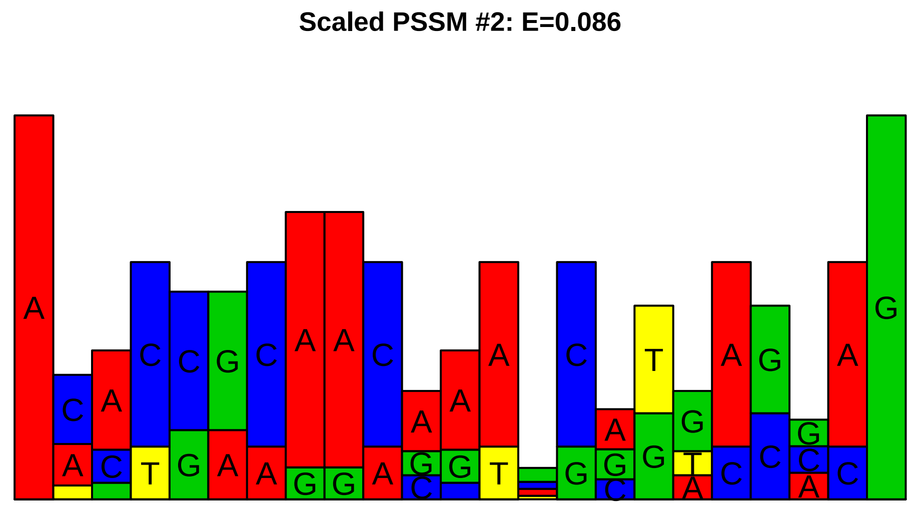
Scaled PSSM #2: E=0.086

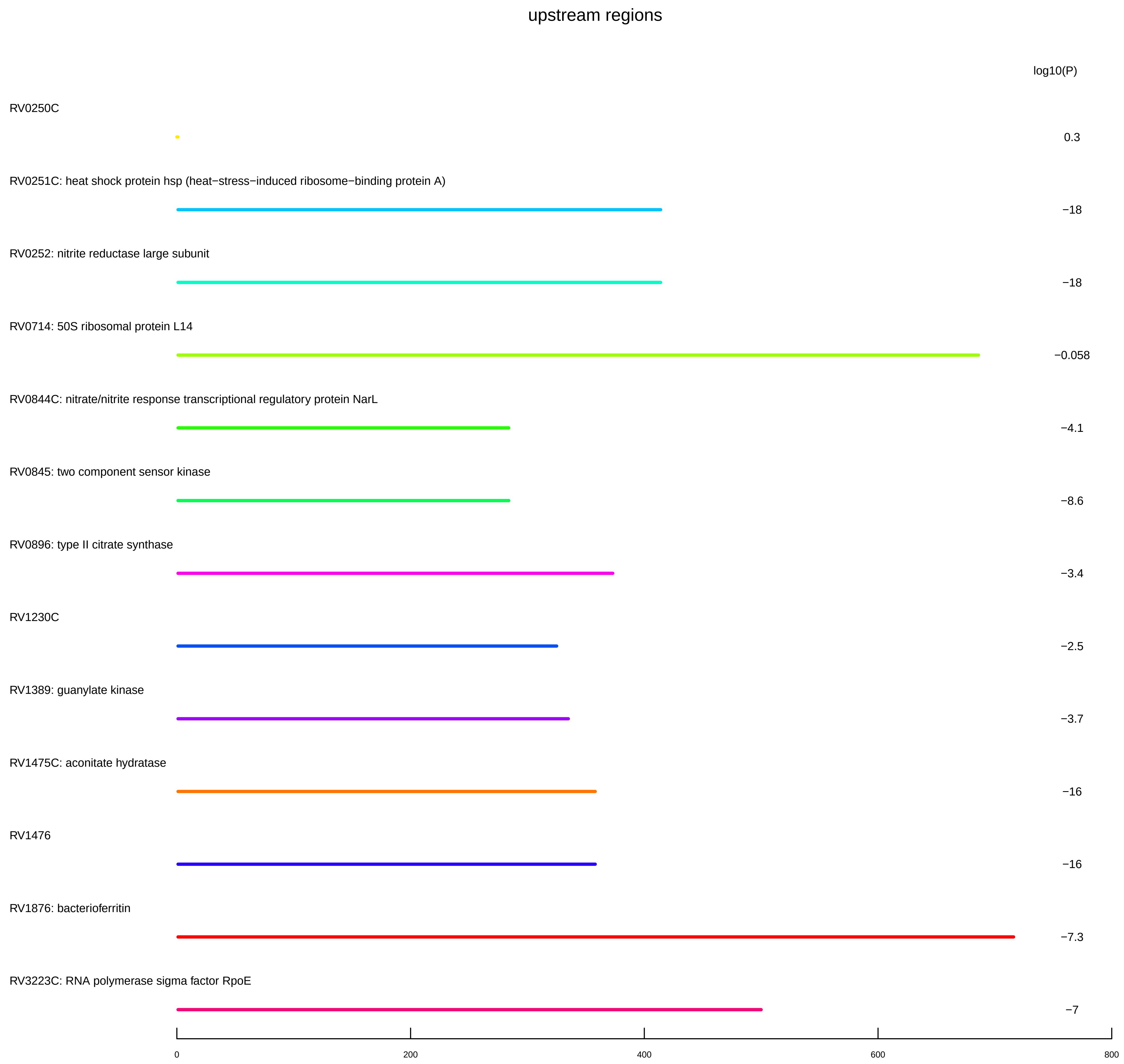
upstream regions
log10(P)
RV0250C
RV0251C: heat shock protein hsp (heat−stress−induced ribosome−binding protein A)
RV0252: nitrite reductase large subunit
RV0714: 50S ribosomal protein L14
RV0844C: nitrate/nitrite response transcriptional regulatory protein NarL
RV0845: two component sensor kinase
RV0896: type II citrate synthase
RV1230C
RV1389: guanylate kinase
RV1475C: aconitate hydratase
RV1476
RV1876: bacterioferritin
RV3223C: RNA polymerase sigma factor RpoE
0.3
−18
−18
−0.058
−4.1
−8.6
−3.4
−2.5
−3.7
−16
−16
−7.3
−7

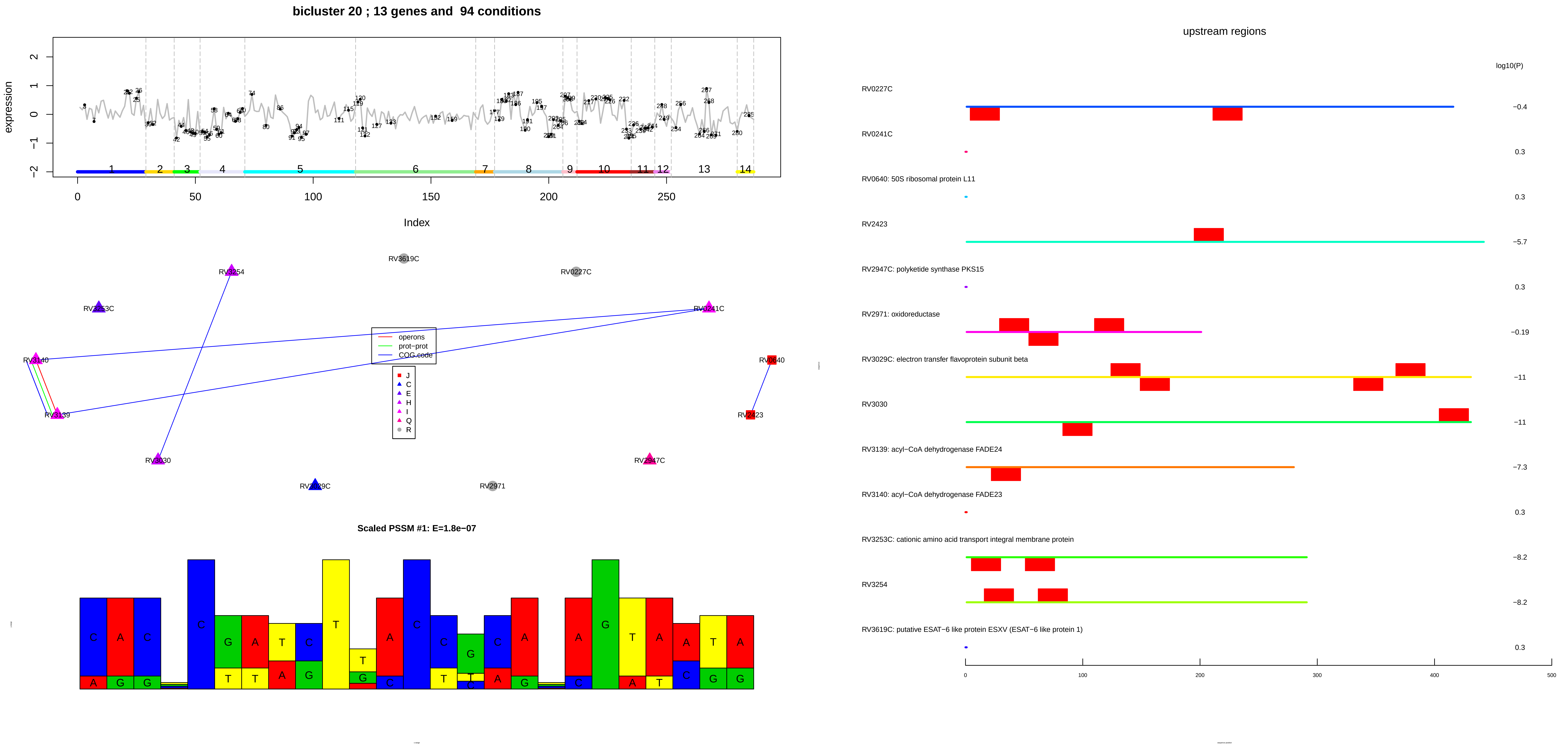
bicluster 20 ; 13 genes and 94 conditions
expression
Index
operons
prot−prot
COG code
RV3619C
RV3254
RV0227C
RV3253C
RV0241C
RV3140
RV0640
RV3139
RV2423
RV3030
RV2947C
RV3029C
RV2971
Scaled PSSM #1: E=1.8e−07
upstream regions
log10(P)
RV0227C
RV0241C
RV0640: 50S ribosomal protein L11
RV2423
RV2947C: polyketide synthase PKS15
RV2971: oxidoreductase
RV3029C: electron transfer flavoprotein subunit beta
RV3030
RV3139: acyl−CoA dehydrogenase FADE24
RV3140: acyl−CoA dehydrogenase FADE23
RV3253C: cationic amino acid transport integral membrane protein
RV3254
RV3619C: putative ESAT−6 like protein ESXV (ESAT−6 like protein 1)

bicluster 21 ; 17 genes and 108 conditions

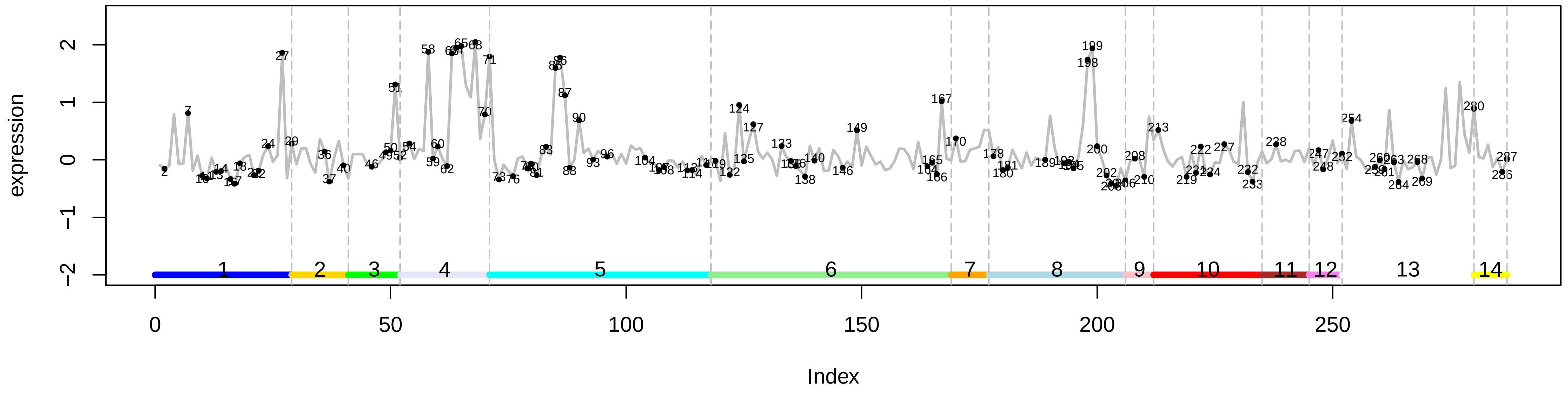

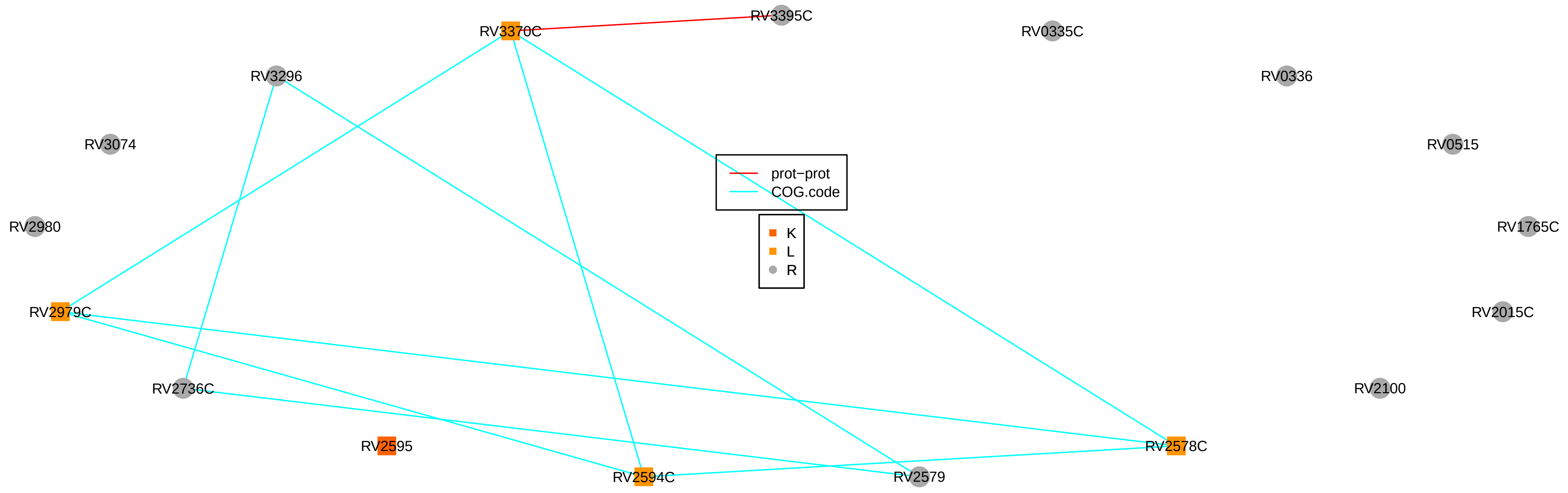

Scaled PSSM #1: E=1.9e−41

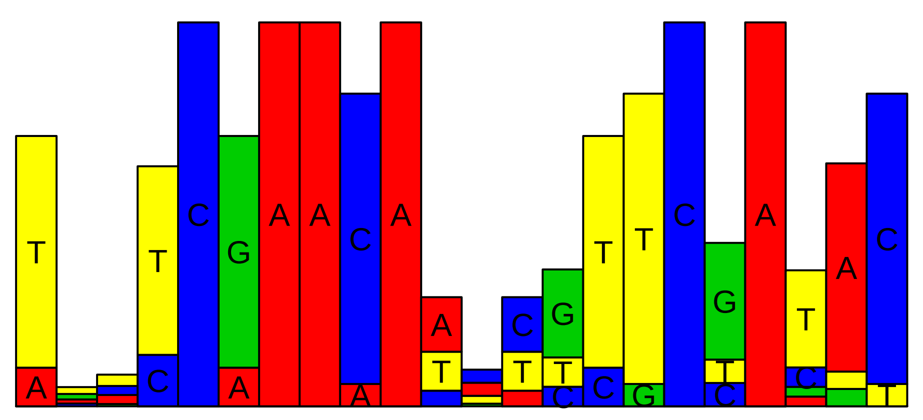

Scaled PSSM #2: E=0.13

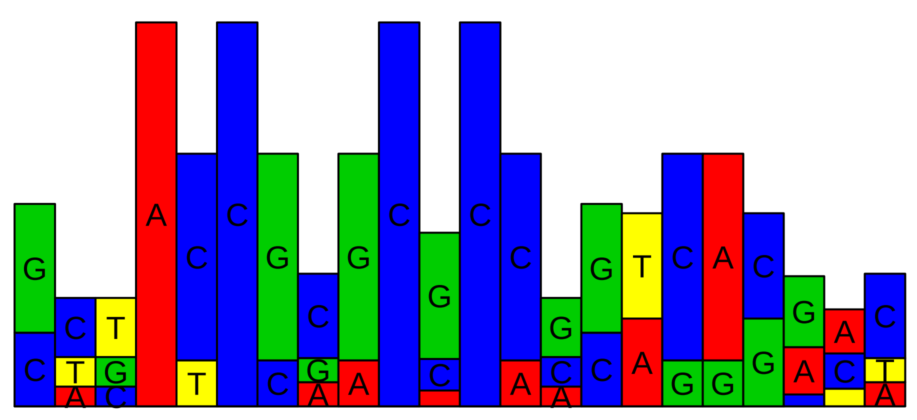

upstream regions

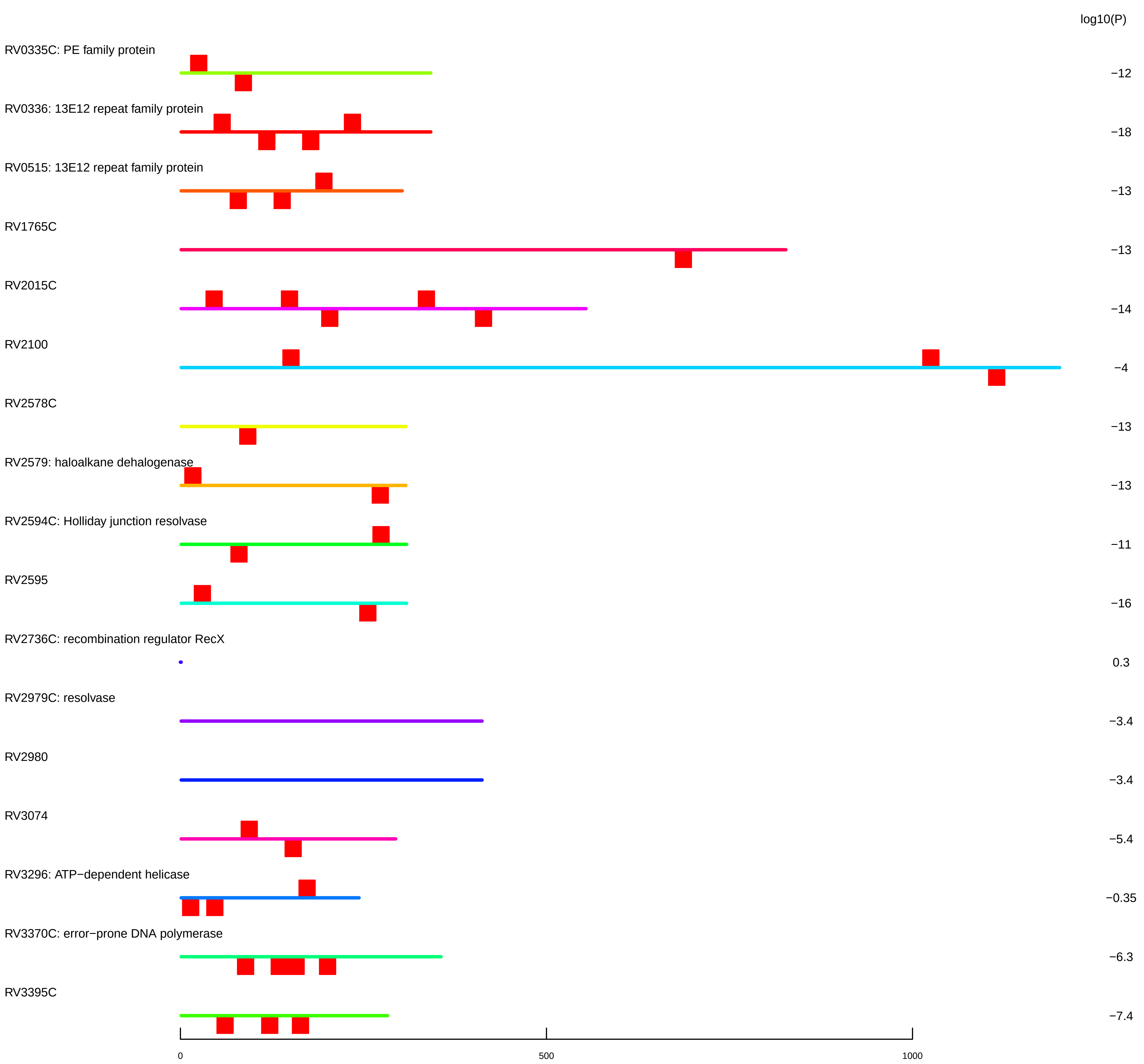

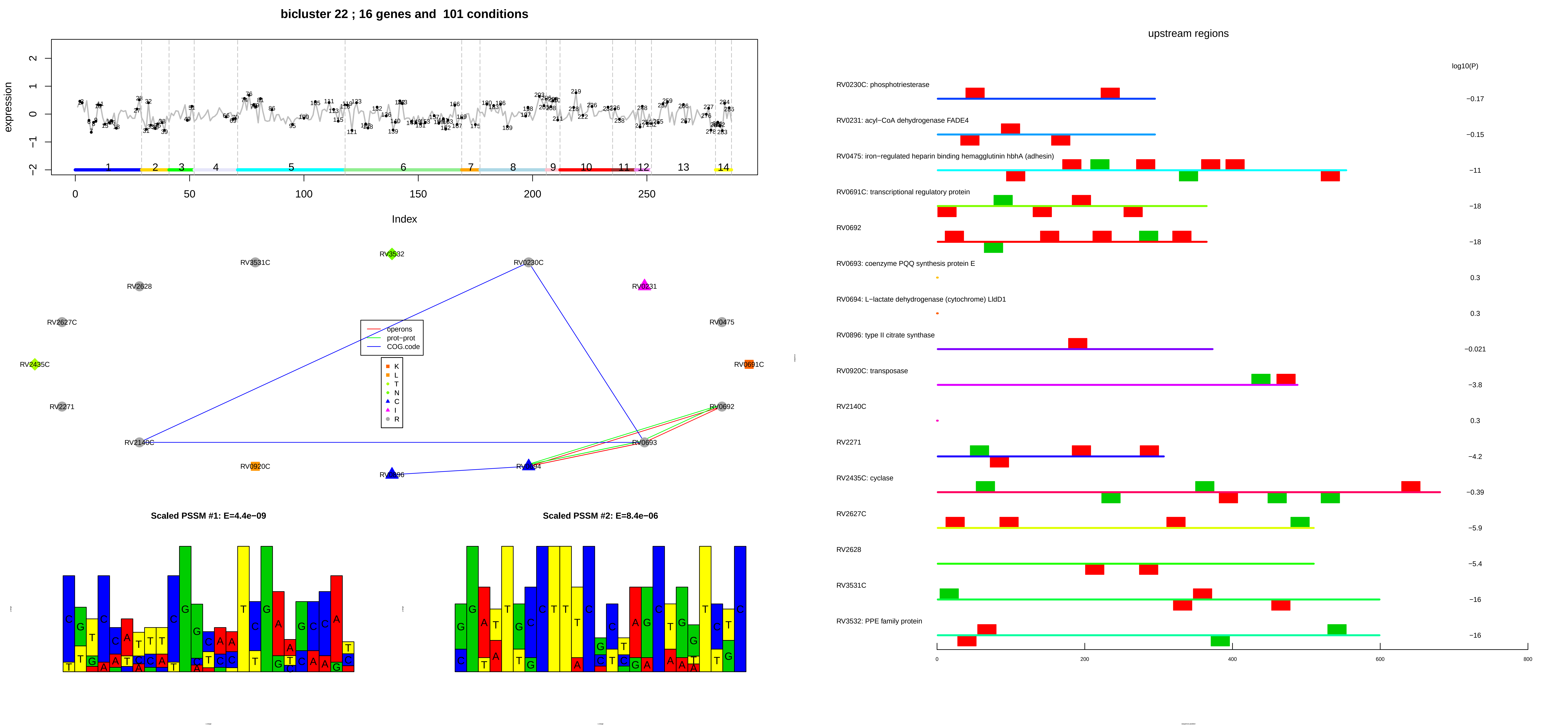

bicluster 22 ; 16 genes and 101 conditions
expression
Index
operons
prot−prot
COG.code
K
L
T
N
C
I
R
RV3531C
RV3532
RV0230C
RV2628
RV0231
RV2627C
RV0475
RV2435C
RV0691C
RV2271
RV0692
RV2140C
RV0693
RV0920C
RV0896
RV0694
Scaled PSSM #1: E=4.4e−09
Scaled PSSM #2: E=8.4e−06
upstream regions
log10(P)
RV0230C: phosphotriesterase −0.17
RV0231: acyl−CoA dehydrogenase FADE4 −0.15
RV0475: iron−regulated heparin binding hemagglutinin hbhA (adhesin) −11
RV0691C: transcriptional regulatory protein −18
RV0692 −18
RV0693: coenzyme PQQ synthesis protein E 0.3
RV0694: L−lactate dehydrogenase (cytochrome) LldD1 0.3
RV0896: type II citrate synthase −0.021
RV0920C: transposase −3.8
RV2140C 0.3
RV2271 −4.2
RV2435C: cyclase −0.39
RV2627C −5.9
RV2628 −5.4
RV3531C −16
RV3532: PPE family protein −16

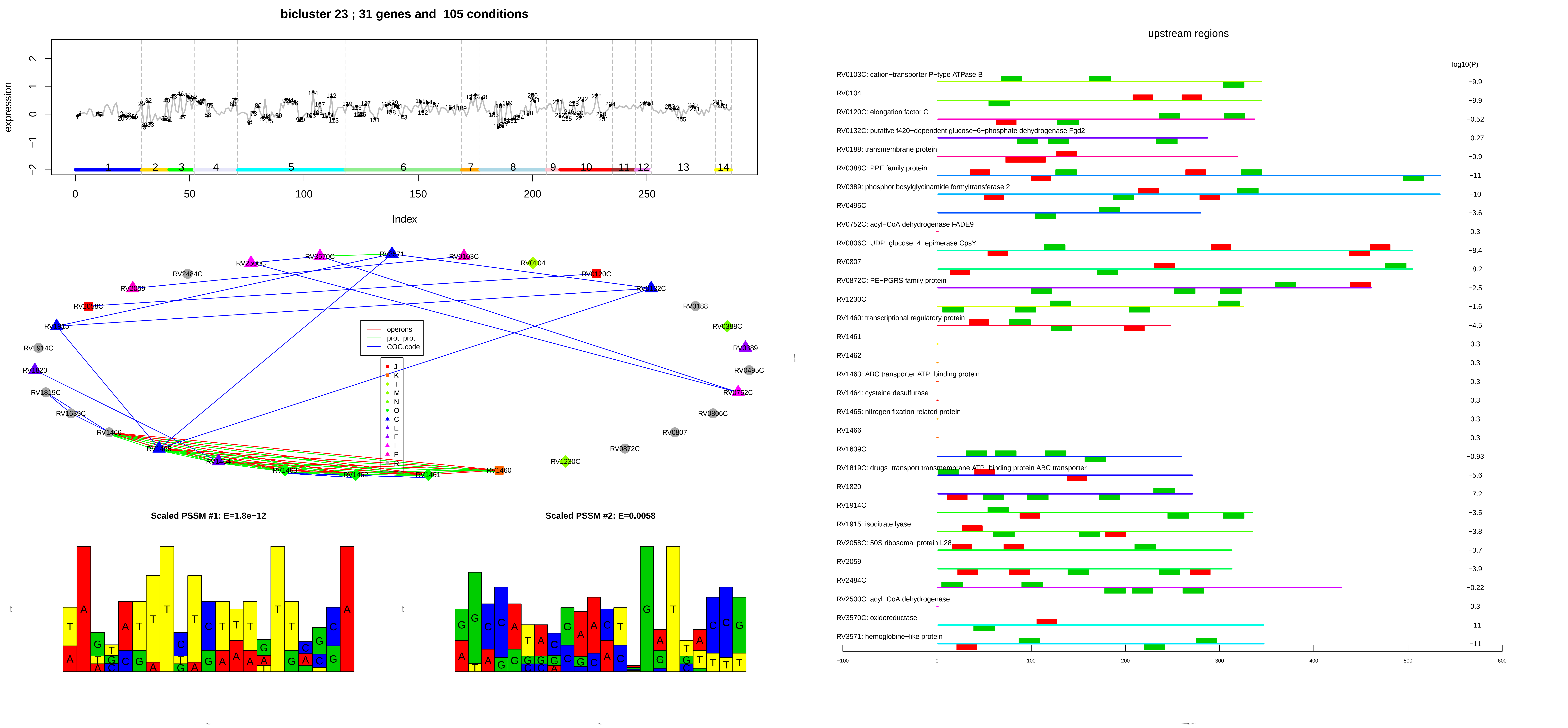

bicluster 23 ; 31 genes and 105 conditions
expression
Index
operons
prot−prot
COG.code
Scaled PSSM #1: E=1.8e−12
Scaled PSSM #2: E=0.0058
upstream regions
log10(P)
RV0103C: cation−transporter P−type ATPase B
RV0104
RV0120C: elongation factor G
RV0132C: putative f420−dependent glucose−6−phosphate dehydrogenase Fgd2
RV0188: transmembrane protein
RV0388C: PPE family protein
RV0389: phosphoribosylglycinamide formyltransferase 2
RV0495C
RV0752C: acyl−CoA dehydrogenase FADE9
RV0806C: UDP−glucose−4−epimerase CpsY
RV0807
RV0872C: PE−PGRS family protein
RV1230C
RV1460: transcriptional regulatory protein
RV1461
RV1462
RV1463: ABC transporter ATP−binding protein
RV1464: cysteine desulfurase
RV1465: nitrogen fixation related protein
RV1466
RV1639C
RV1819C: drugs−transport transmembrane ATP−binding protein ABC transporter
RV1820
RV1914C
RV1915: isocitrate lyase
RV2058C: 50S ribosomal protein L28
RV2059
RV2484C
RV2500C: acyl−CoA dehydrogenase
RV3570C: oxidoreductase
RV3571: hemoglobine−like protein

bicluster 24 ; 32 genes and 82 conditions

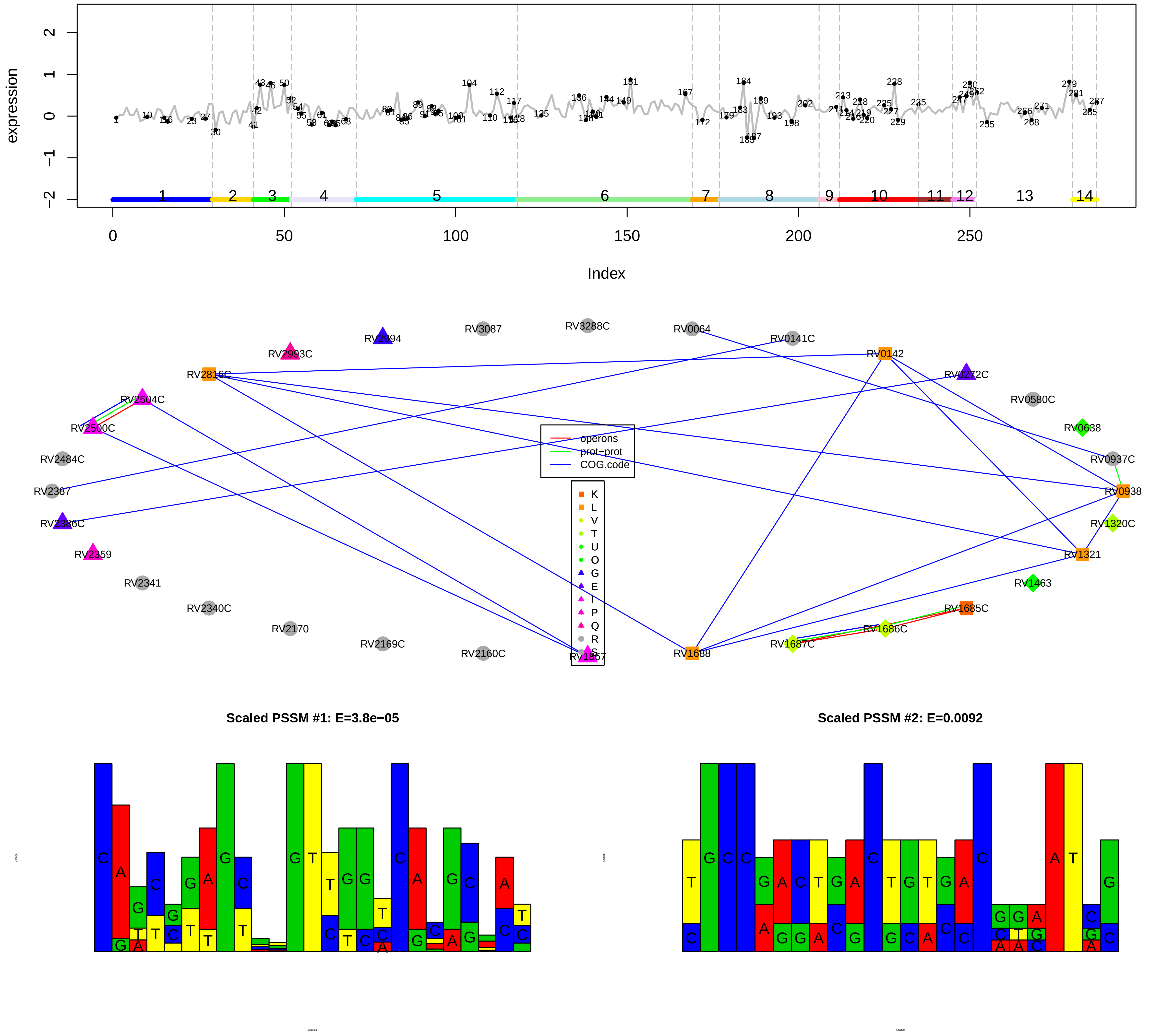

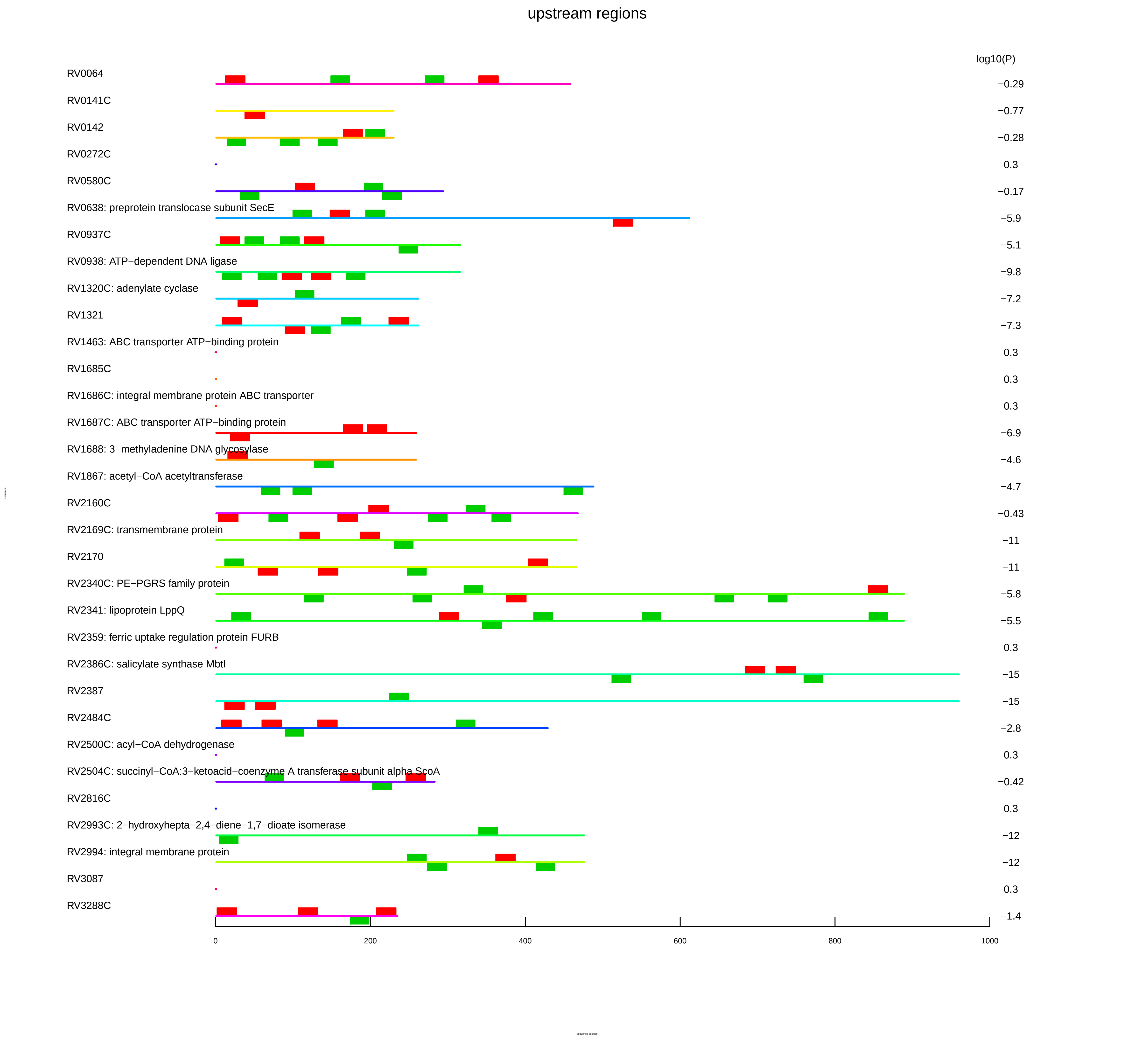

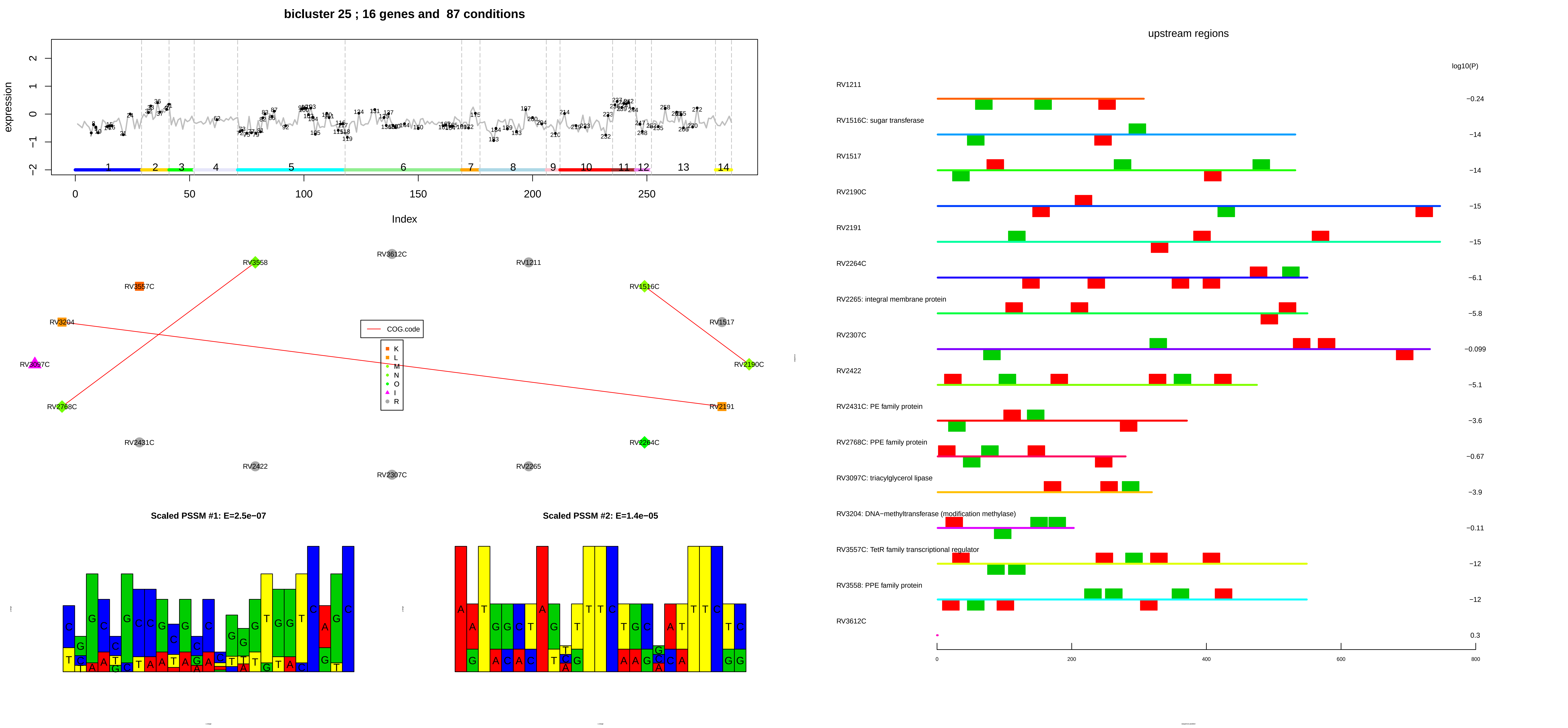
bicluster 25 ; 16 genes and 87 conditions
expression
Index
1 2 3 4 5 6 7 8 9 10 11 12 13 14
RV3612C
RV3558
RV1211
RV3557C
RV1516C
RV3204
RV1517
COG.code
K
L
M
N
O
I
R
RV3097C
RV2190C
RV2768C
RV2191
RV2431C
RV2264C
RV2422
RV2265
RV2307C
Scaled PSSM #1: E=2.5e−07
Scaled PSSM #2: E=1.4e−05
upstream regions
log10(P)
RV1211 −0.24
RV1516C: sugar transferase −14
RV1517 −14
RV2190C −15
RV2191 −15
RV2264C −6.1
RV2265: integral membrane protein −5.8
RV2307C −0.099
RV2422 −5.1
RV2431C: PE family protein −3.6
RV2768C: PPE family protein −0.67
RV3097C: triacylglycerol lipase −3.9
RV3204: DNA−methyltransferase (modification methylase) −0.11
RV3557C: TetR family transcriptional regulator −12
RV3558: PPE family protein −12
RV3612C 0.3

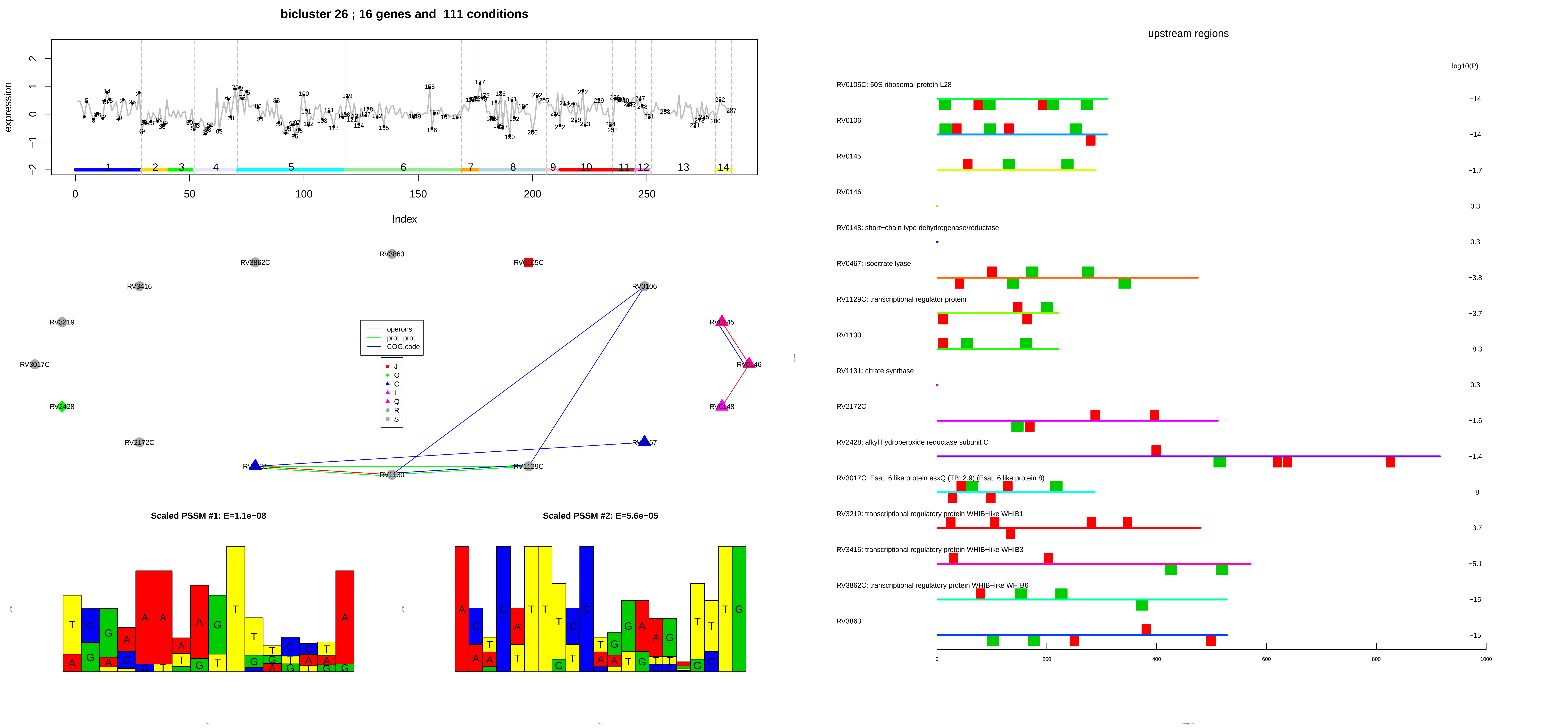

bicluster 26 ; 16 genes and 111 conditions
expression
Index
operons
prot−prot
COG.code
J
O
C
I
Q
R
S
RV3862C
RV3863
RV0105C
RV3416
RV0106
RV3219
RV0145
RV3017C
RV0146
RV2428
RV0148
RV2172C
RV0467
RV1131
RV1130
RV1129C
Scaled PSSM #1: E=1.1e−08
Scaled PSSM #2: E=5.6e−05
upstream regions
log10(P)
RV0105C: 50S ribosomal protein L28
RV0106
RV0145
RV0146
RV0148: short−chain type dehydrogenase/reductase
RV0467: isocitrate lyase
RV1129C: transcriptional regulator protein
RV1130
RV1131: citrate synthase
RV2172C
RV2428: alkyl hydroperoxide reductase subunit C
RV3017C: Esat−6 like protein esxQ (TB12.9) (Esat−6 like protein 8)
RV3219: transcriptional regulatory protein WHIB−like WHIB1
RV3416: transcriptional regulatory protein WHIB−like WHIB3
RV3862C: transcriptional regulatory protein WHIB−like WHIB6
RV3863
−14
−14
−1.7
0.3
0.3
−3.8
−3.7
−8.3
0.3
−1.6
−1.4
−8
−3.7
−5.1
−15
−15

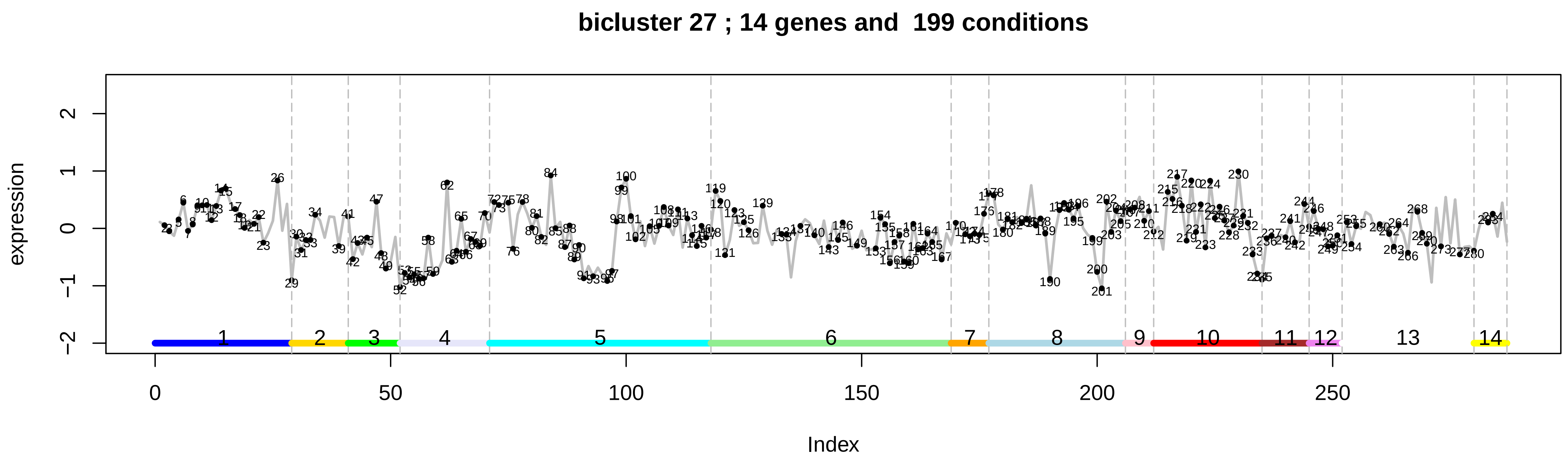
bicluster 27 ; 14 genes and 199 conditions
expression
Index
1 2 3 4 5 6 7 8 9 10 11 12 13 14

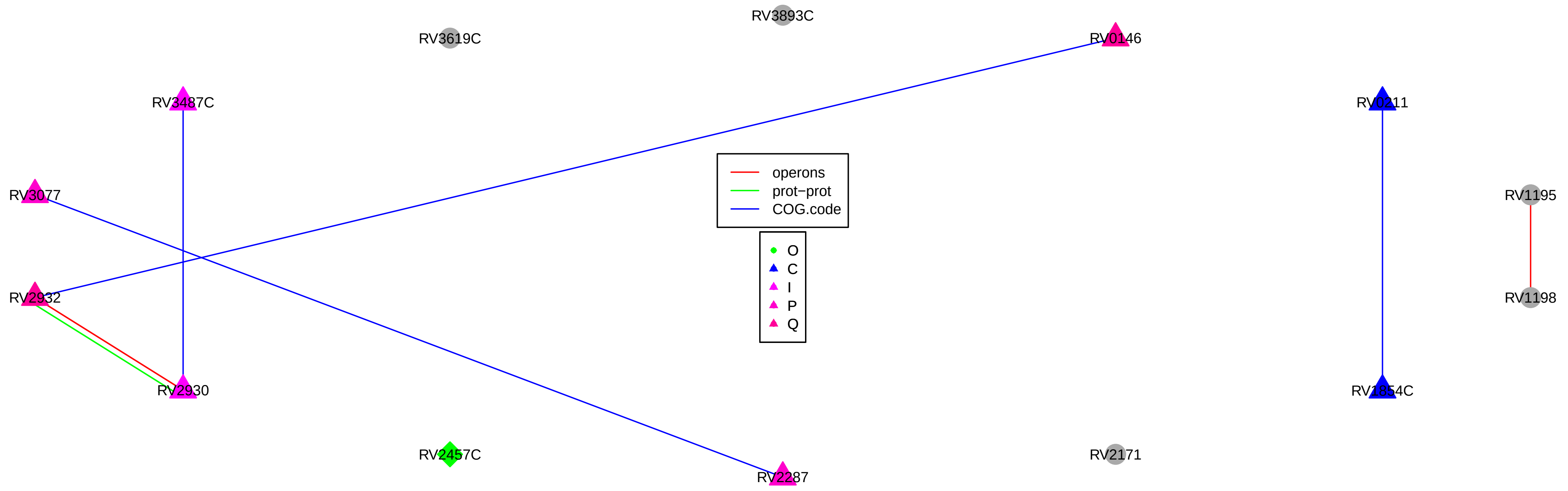
RV3893C
RV3619C
RV0146
RV3487C
RV0211
RV3077
RV1195
RV2932
RV1198
RV2930
RV1854C
RV2457C
RV2171
RV2287
operons
prot−prot
COG.code
O
C
I
P
Q

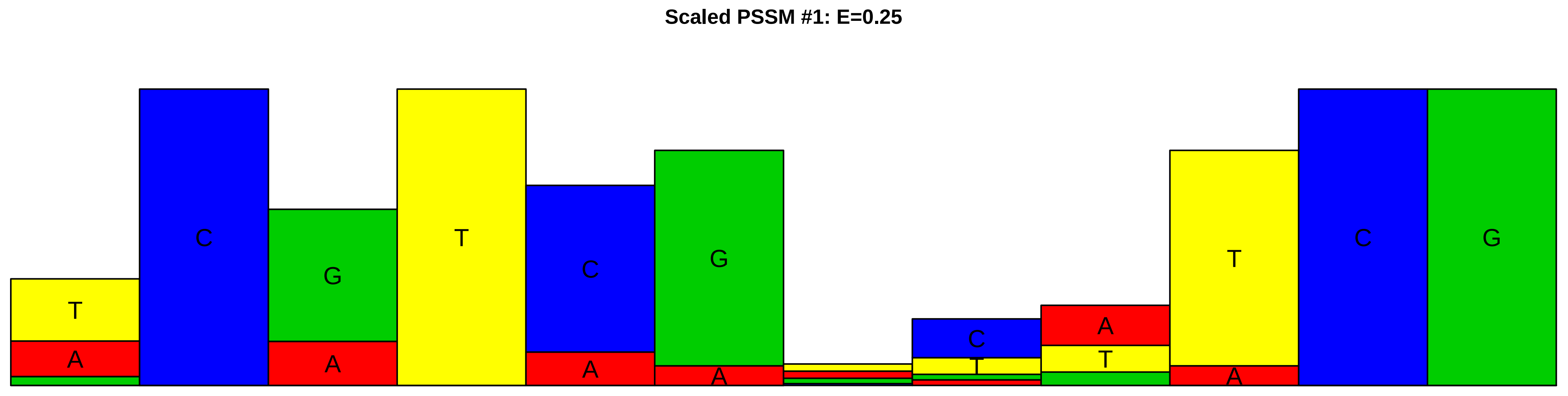
Scaled PSSM #1: E=0.25

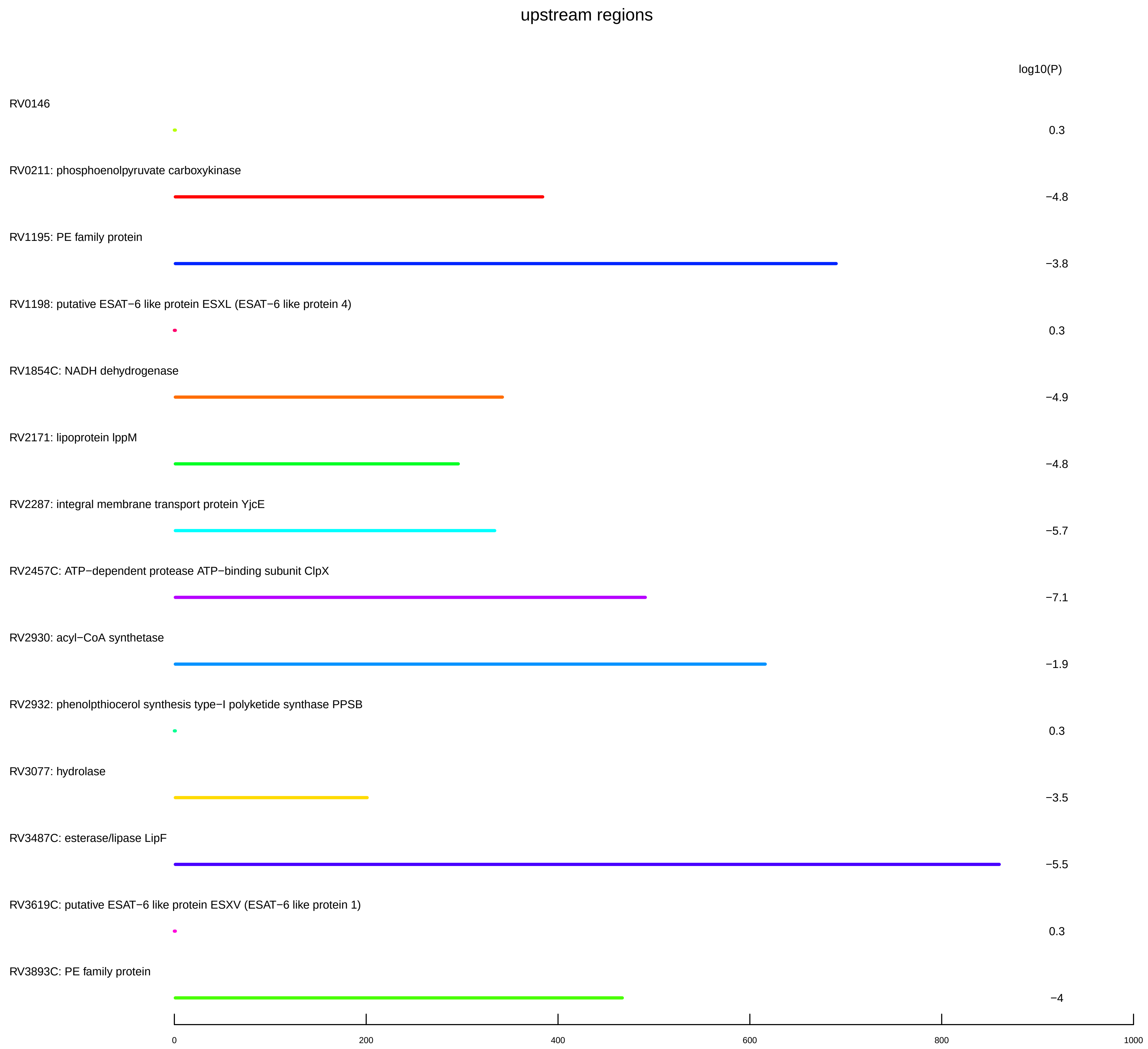
upstream regions
log10(P)
RV0146 0.3
RV0211: phosphoenolpyruvate carboxykinase −4.8
RV1195: PE family protein −3.8
RV1198: putative ESAT−6 like protein ESXL (ESAT−6 like protein 4) 0.3
RV1854C: NADH dehydrogenase −4.9
RV2171: lipoprotein lppM −4.8
RV2287: integral membrane transport protein YjcE −5.7
RV2457C: ATP−dependent protease ATP−binding subunit ClpX −7.1
RV2930: acyl−CoA synthetase −1.9
RV2932: phenolpthiocerol synthesis type−I polyketide synthase PPSB 0.3
RV3077: hydrolase −3.5
RV3487C: esterase/lipase LipF −5.5
RV3619C: putative ESAT−6 like protein ESXV (ESAT−6 like protein 1) 0.3
RV3893C: PE family protein −4
0 200 400 600 800 1000

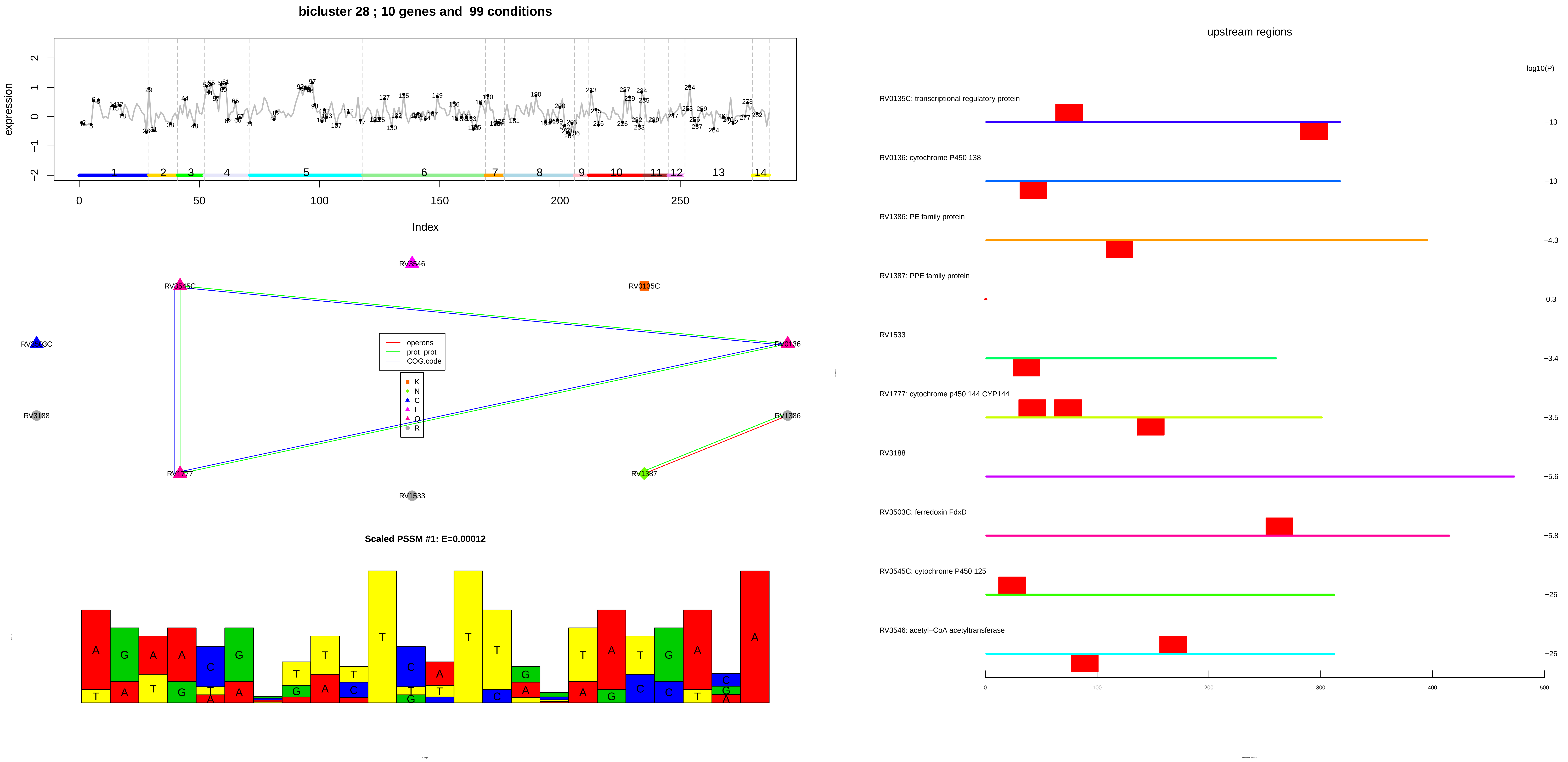

bicluster 28 ; 10 genes and 99 conditions
expression
Index
RV3546
RV3545C
RV0135C
RV3503C
RV0136
RV3188
RV1386
RV1777
RV1387
RV1533
operons
prot−prot
COG.code
K
N
C
I
Q
R
Scaled PSSM #1: E=0.00012
upstream regions
log10(P)
RV0135C: transcriptional regulatory protein
−13
RV0136: cytochrome P450 138
−13
RV1386: PE family protein
−4.3
RV1387: PPE family protein
0.3
RV1533
−3.4
RV1777: cytochrome p450 144 CYP144
−3.5
RV3188
−5.6
RV3503C: ferredoxin FdxD
−5.8
RV3545C: cytochrome P450 125
−26
RV3546: acetyl−CoA acetyltransferase
−26

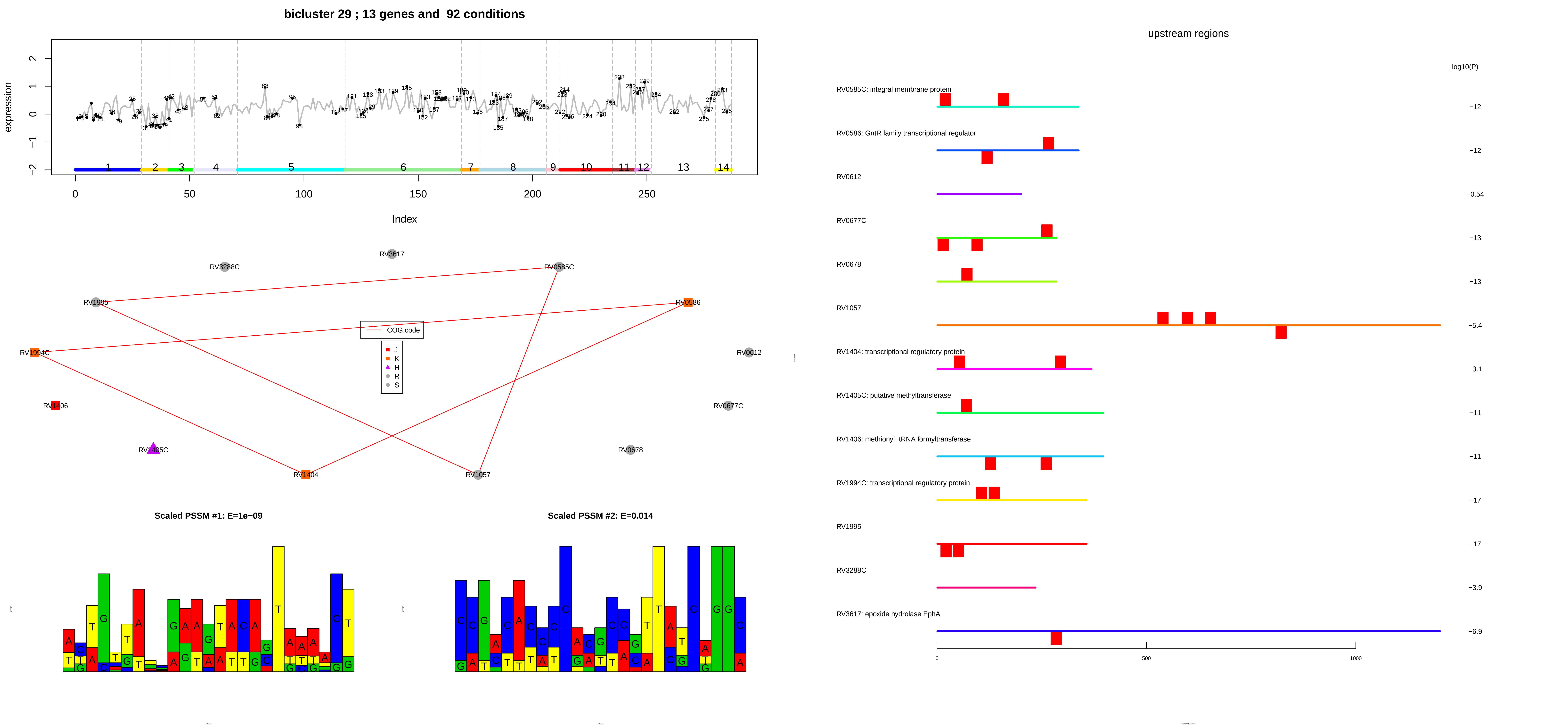

bicluster 29 ; 13 genes and 92 conditions
expression
Index
upstream regions
log10(P)
RV0585C: integral membrane protein
RV0586: GntR family transcriptional regulator
RV0612
RV0677C
RV0678
RV1057
RV1404: transcriptional regulatory protein
RV1405C: putative methyltransferase
RV1406: methionyl−tRNA formyltransferase
RV1994C: transcriptional regulatory protein
RV1995
RV3288C
RV3617: epoxide hydrolase EphA
COG.code
Scaled PSSM #1: E=1e−09
Scaled PSSM #2: E=0.014

bicluster 30 ; 48 genes and 193 conditions

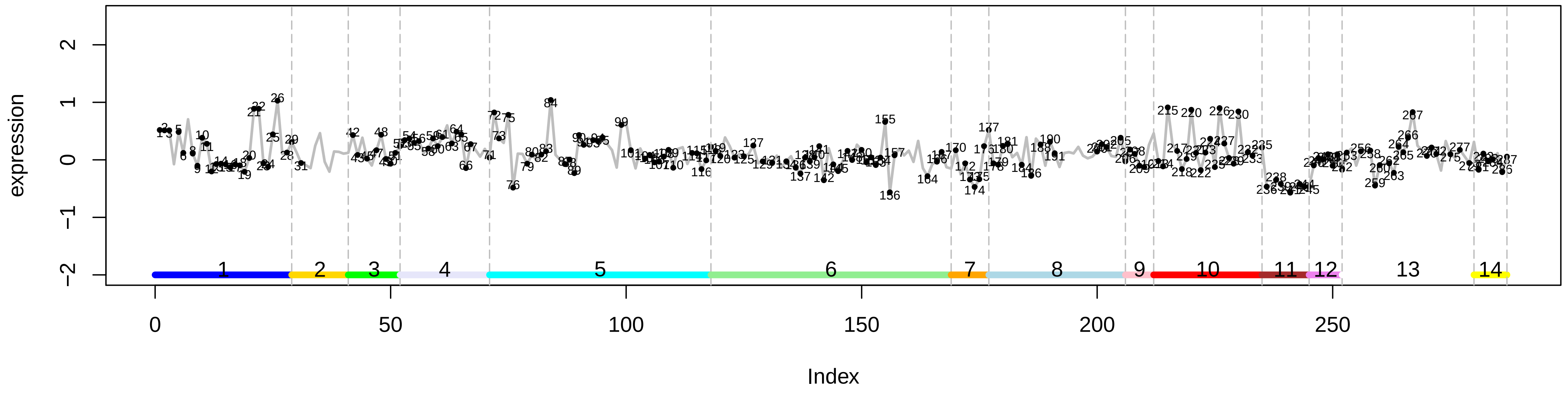

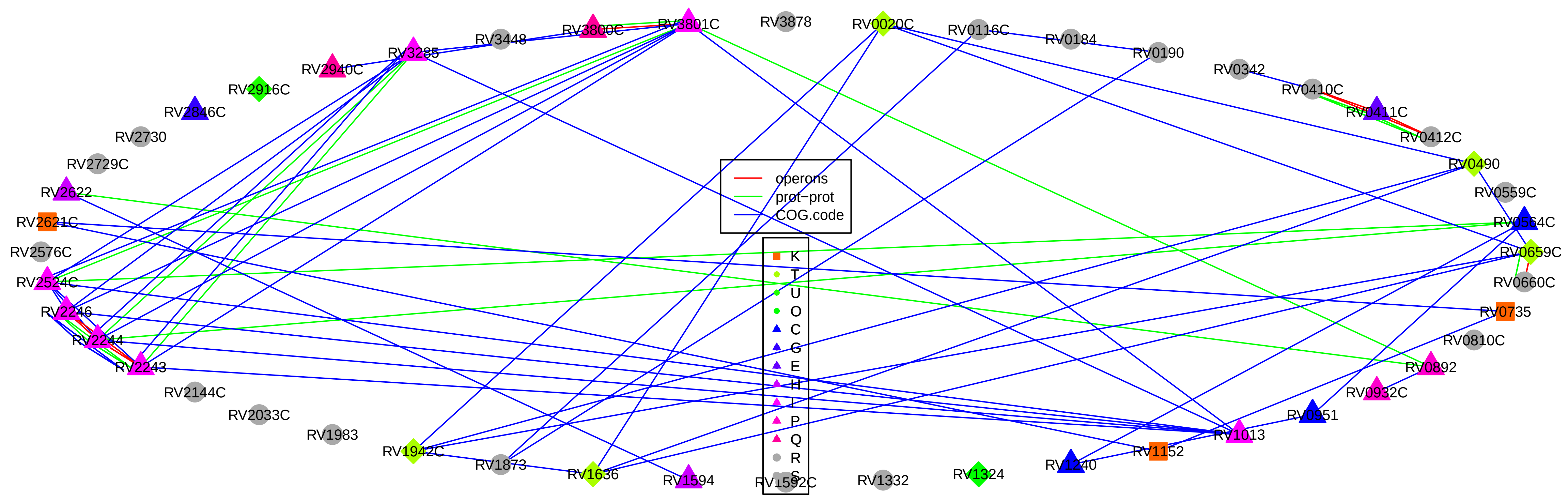

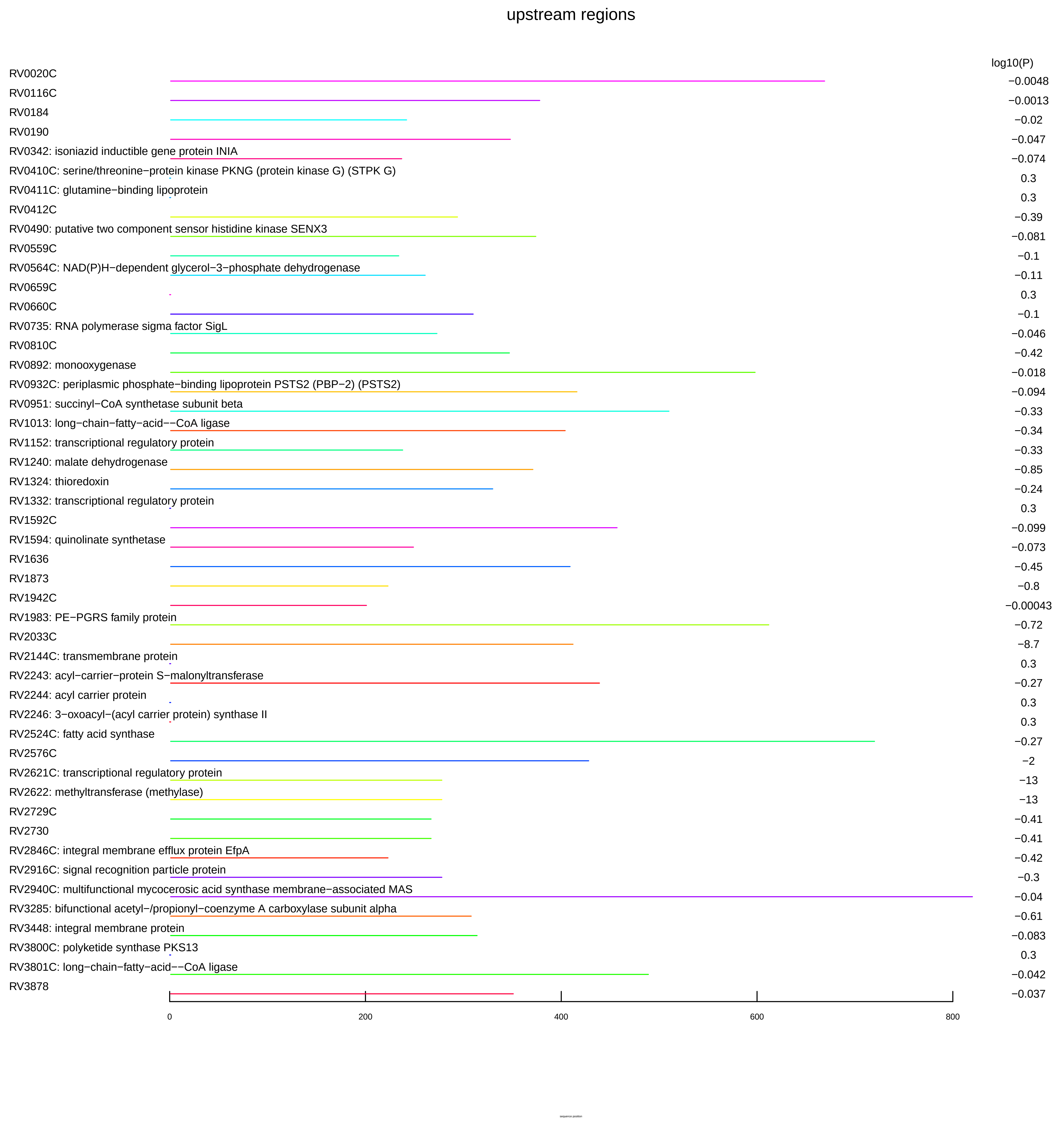

bicluster 31 ; 29 genes and 75 conditions

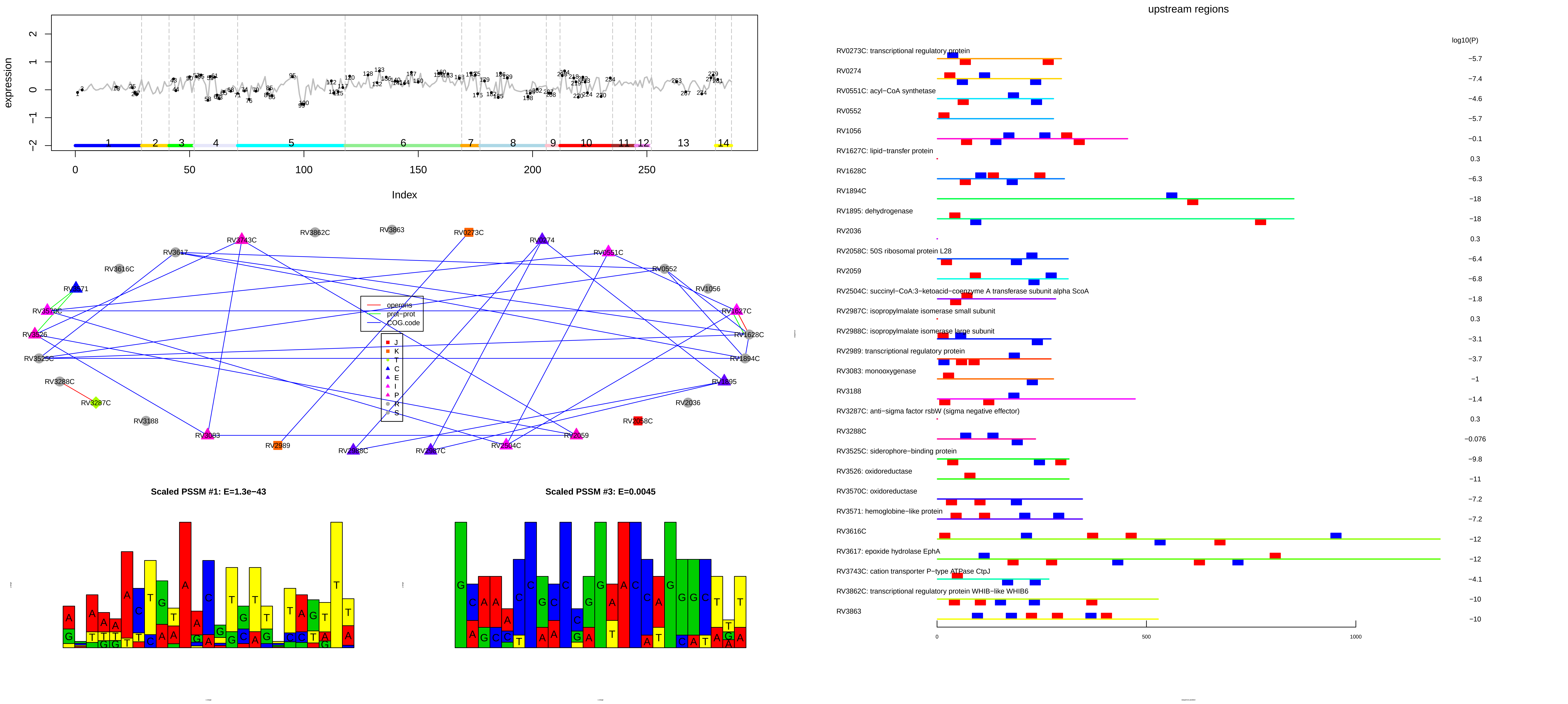

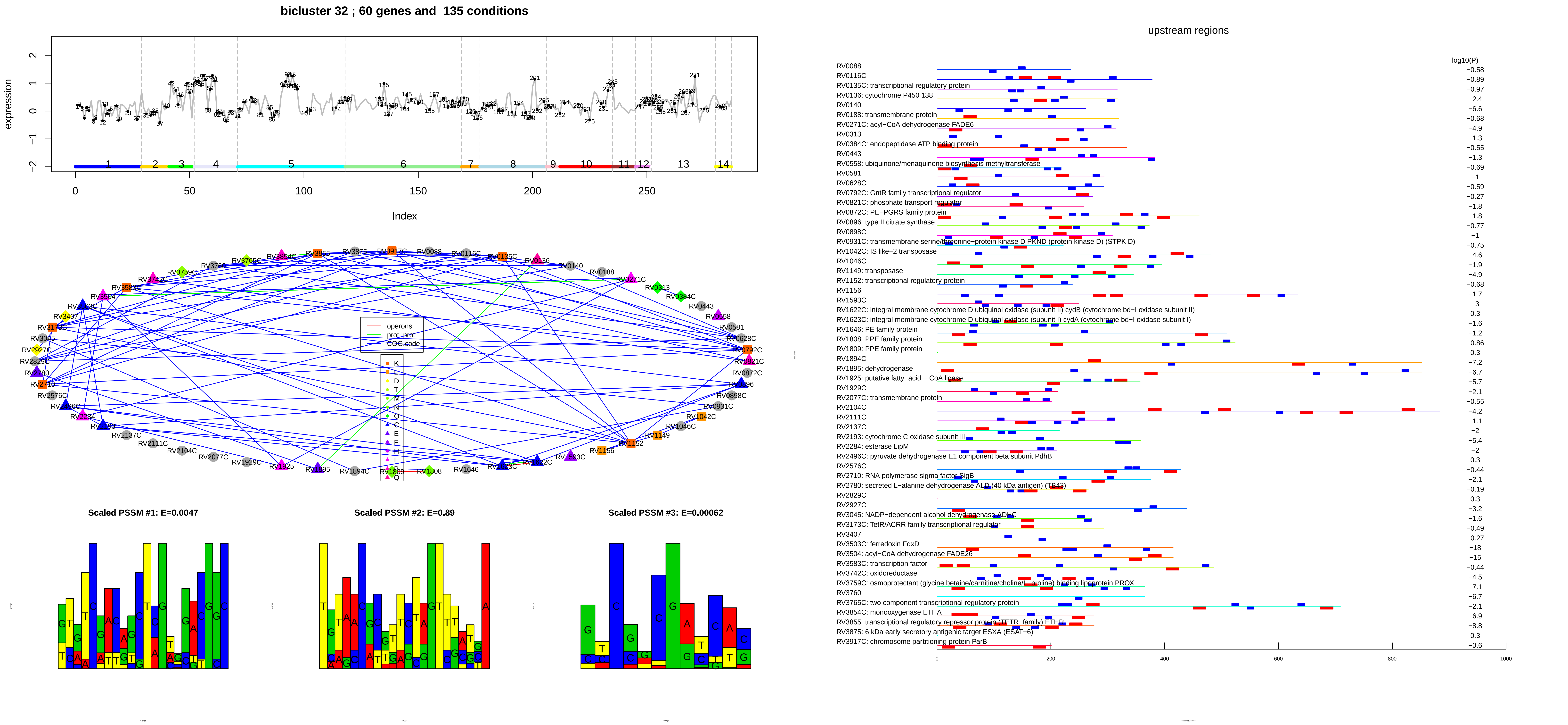
bicluster 32 ; 60 genes and 135 conditions
expression
Index
operons
prot−prot
COG code
Scaled PSSM #1: E=0.0047
Scaled PSSM #2: E=0.89
Scaled PSSM #3: E=0.00062
upstream regions
log10(P)
RV0088
RV0116C
RV0135C: transcriptional regulatory protein
RV0136: cytochrome P450 138
RV0140
RV0188: transmembrane protein
RV0271C: acyl−CoA dehydrogenase FADE6
RV0313
RV0384C: endopeptidase ATP binding protein
RV0443
RV0558: ubiquinone/menaquinone biosynthesis methyltransferase
RV0581
RV0628C
RV0792C: GntR family transcriptional regulator
RV0821C: phosphate transport regulator
RV0872C: PE−PGRS family protein
RV0896: type II citrate synthase
RV0898C
RV0931C: transmembrane serine/threonine−protein kinase D PKND (protein kinase D) (STPK D)
RV1042C: IS like−2 transposase
RV1046C
RV1149: transposase
RV1152: transcriptional regulatory protein
RV1156
RV1593C
RV1622C: integral membrane cytochrome D ubiquinol oxidase (subunit II) cydB (cytochrome bd−I oxidase subunit II)
RV1623C: integral membrane cytochrome D ubiquinol oxidase (subunit I) cydA (cytochrome bd−I oxidase subunit I)
RV1646: PE family protein
RV1808: PPE family protein
RV1809: PPE family protein
RV1894C
RV1895: dehydrogenase
RV1925: putative fatty−acid−−CoA ligase
RV1929C
RV2077C: transmembrane protein
RV2104C
RV2111C
RV2137C
RV2193: cytochrome C oxidase subunit III
RV2284: esterase LipM
RV2496C: pyruvate dehydrogenase E1 component beta subunit PdhB
RV2576C
RV2710: RNA polymerase sigma factor SigB
RV2780: secreted L−alanine dehydrogenase ALD (40 kDa antigen) (TB43)
RV2829C
RV2927C
RV3045: NADP−dependent alcohol dehydrogenase ADHC
RV3173C: TetR/ACRR family transcriptional regulator
RV3407
RV3503C: ferredoxin FdxD
RV3504: acyl−CoA dehydrogenase FADE26
RV3583C: transcription factor
RV3742C: oxidoreductase
RV3759C: osmoprotectant (glycine betaine/carnitine/choline/L−proline) binding lipoprotein PROX
RV3760
RV3765C: two component transcriptional regulatory protein
RV3854C: monooxygenase ETHA
RV3855: transcriptional regulatory repressor protein (TETR−family) ETHR
RV3875: 6 kDa early secretory antigenic target ESXA (ESAT−6)
RV3917C: chromosome partitioning protein ParB

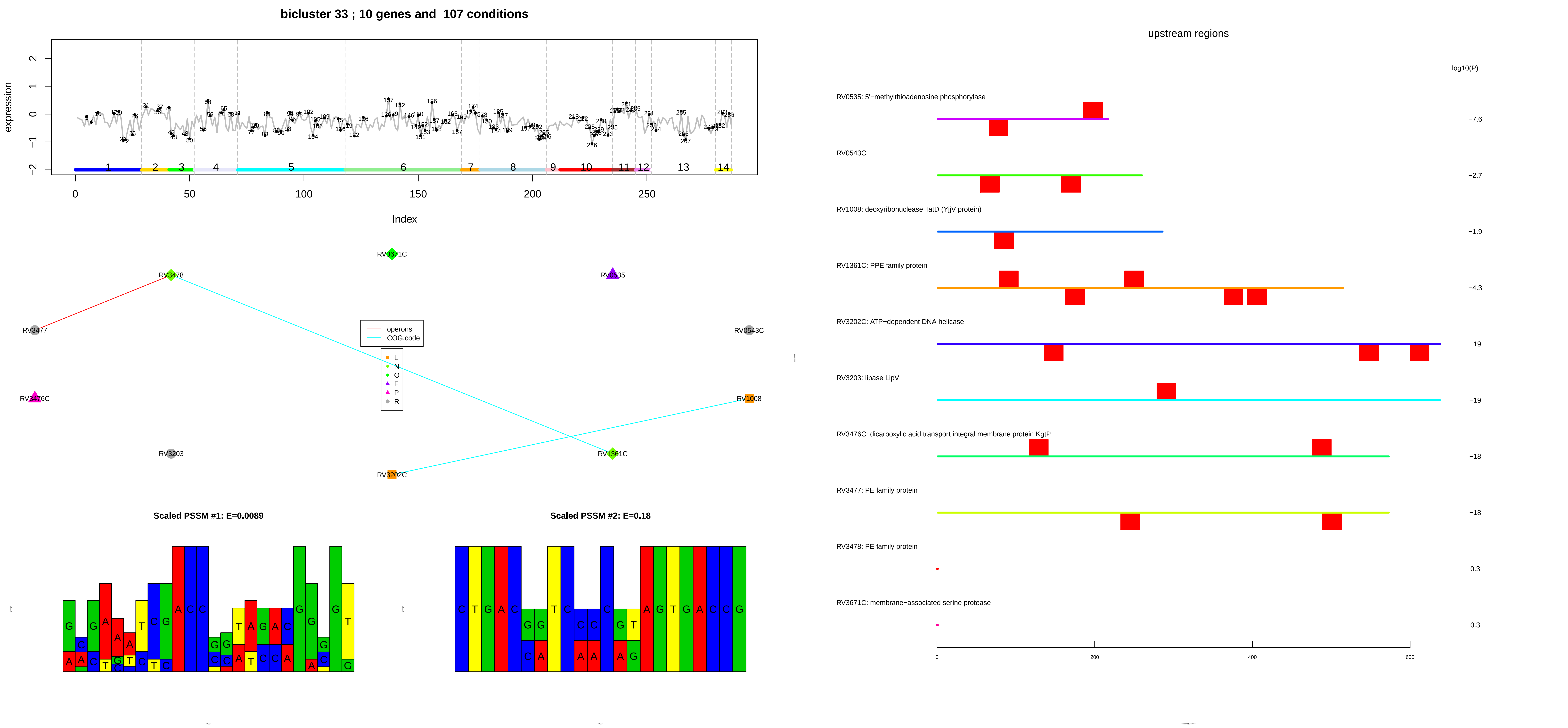

bicluster 33 ; 10 genes and 107 conditions
expression
Index
upstream regions
log10(P)
RV0535: 5'−methylthioadenosine phosphorylase
−7.6
RV0543C
−2.7
RV1008: deoxyribonuclease TatD (YjjV protein)
−1.9
RV1361C: PPE family protein
−4.3
RV3202C: ATP−dependent DNA helicase
−19
RV3203: lipase LipV
−19
RV3476C: dicarboxylic acid transport integral membrane protein KgtP
−18
RV3477: PE family protein
−18
RV3478: PE family protein
0.3
RV3671C: membrane−associated serine protease
0.3
RV3671C
RV3478
RV0535
RV3477
RV0543C
RV3476C
RV1008
RV3203
RV1361C
RV3202C
operons
COG.code
L
N
O
F
P
R
Scaled PSSM #1: E=0.0089
Scaled PSSM #2: E=0.18

bicluster 34 ; 18 genes and 115 conditions

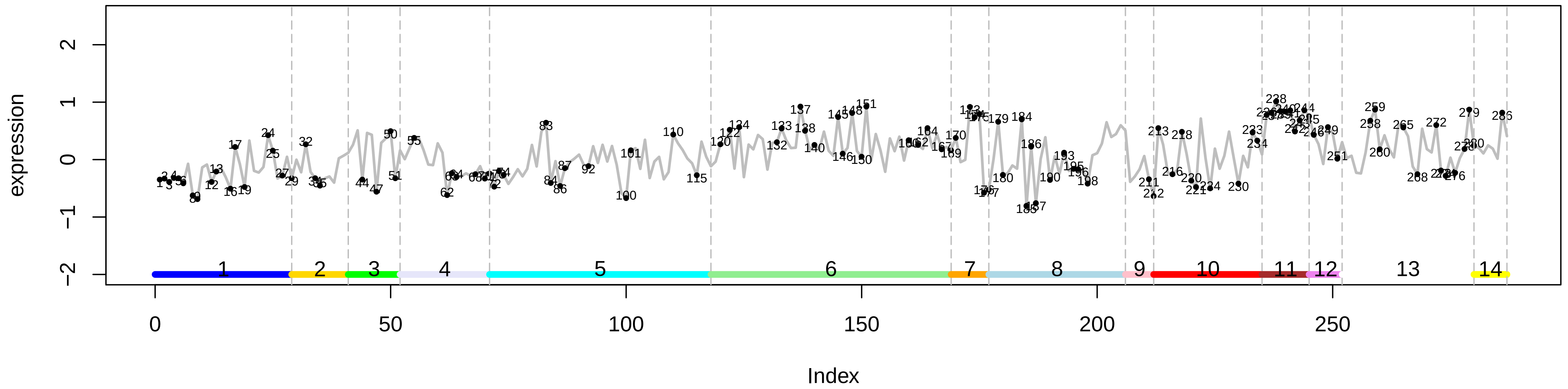

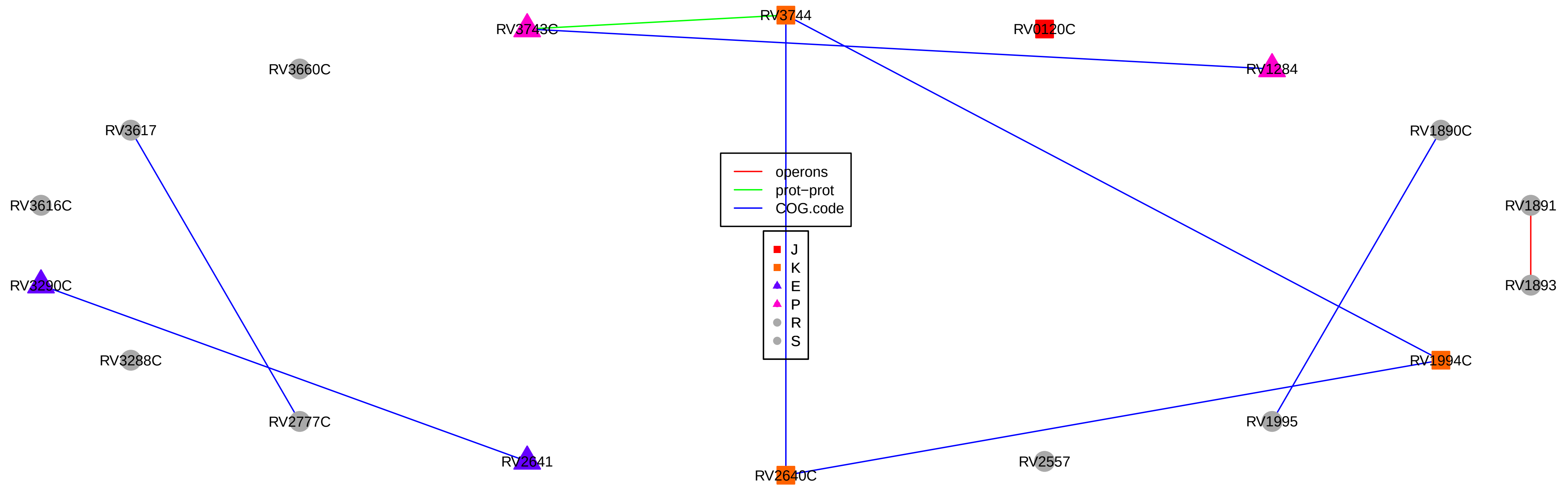

Scaled PSSM #1: E=7.2e−16

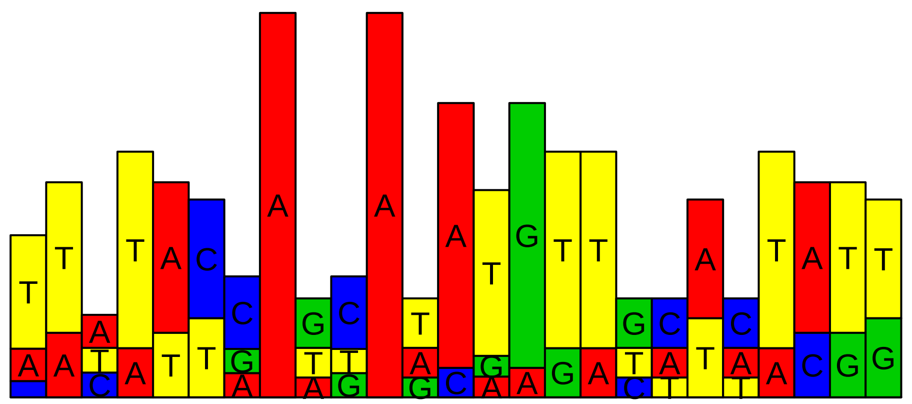

Scaled PSSM #2: E=0.00013

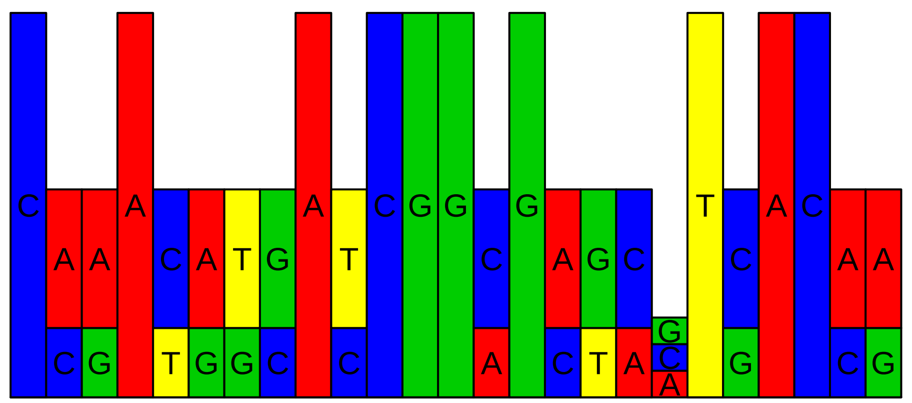

upstream regions

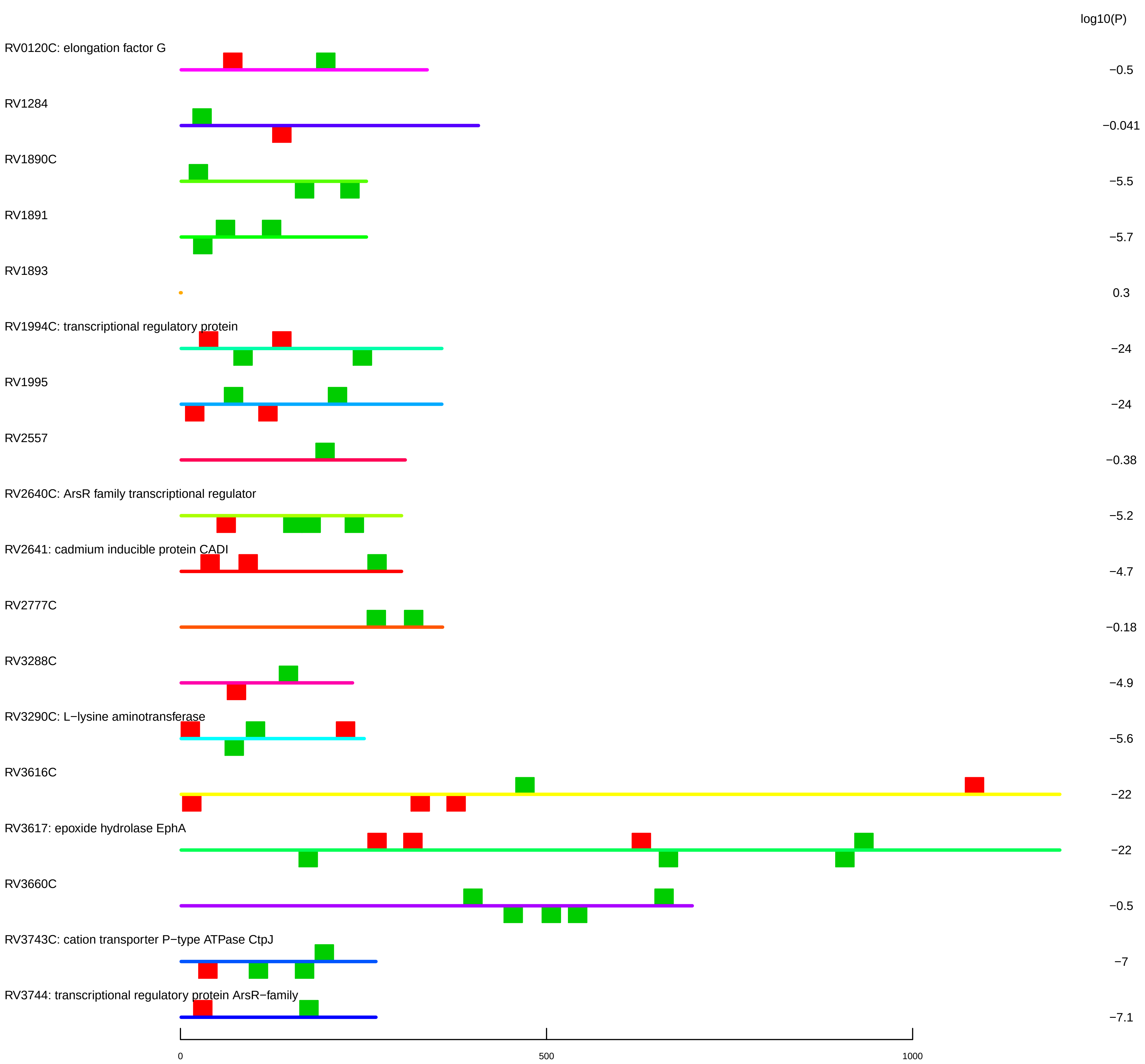

bicluster 35 ; 121 genes and 265 conditions

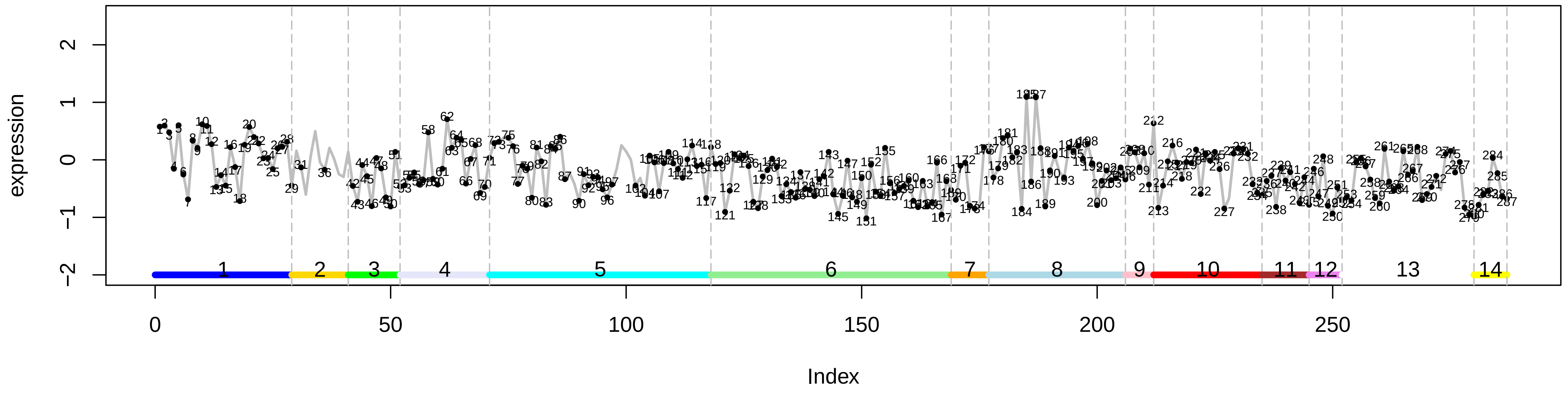

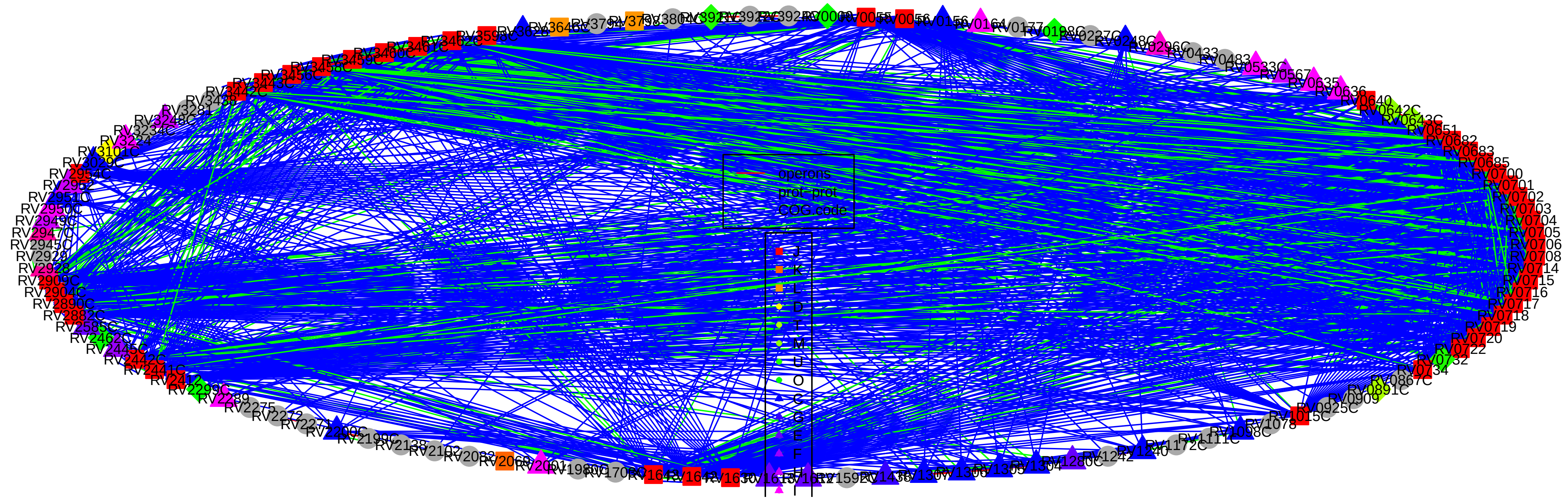

Scaled PSSM #1: E=2.4e−23

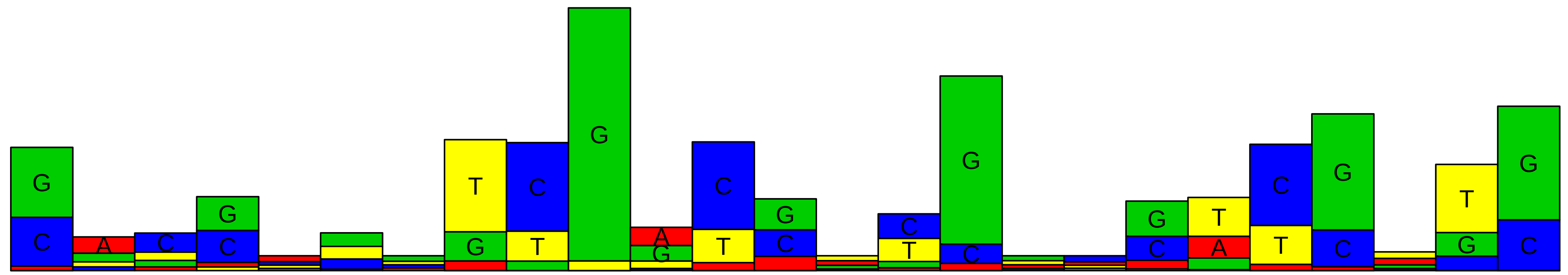

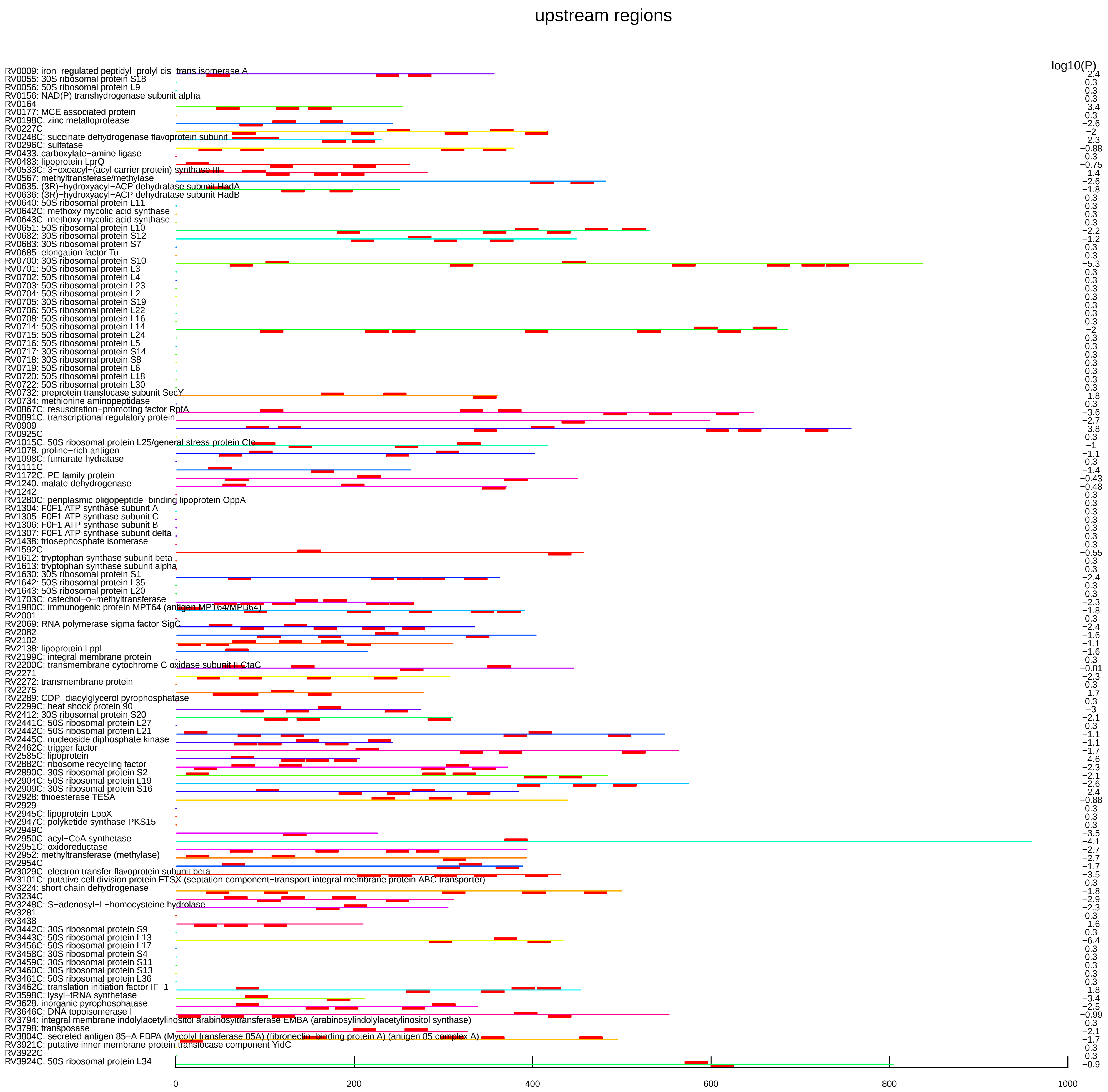

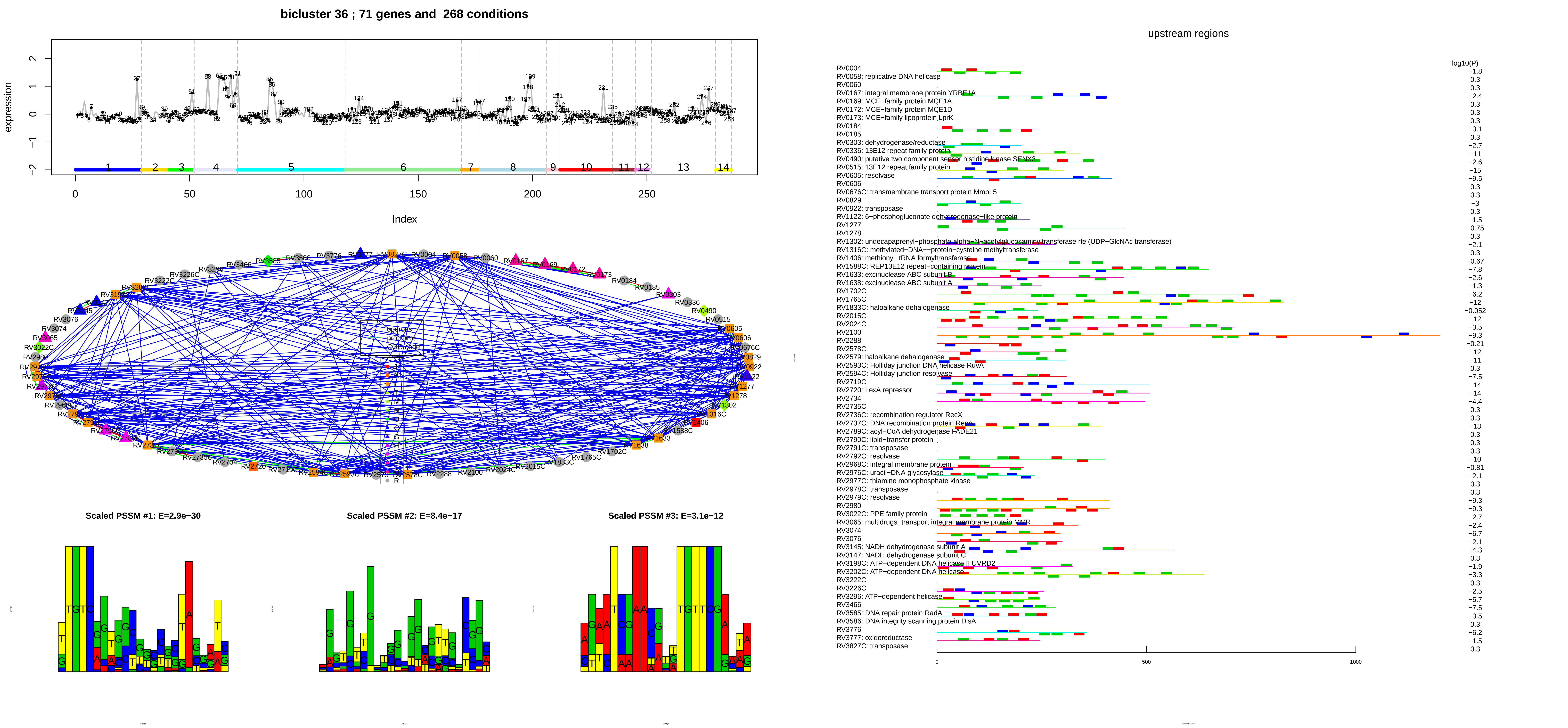

bicluster 36 ; 71 genes and 268 conditions
expression
Index
Scaled PSSM #1: E=2.9e−30
Scaled PSSM #2: E=8.4e−17
Scaled PSSM #3: E=3.1e−12
upstream regions
log10(P)
RV0004
RV0058: replicative DNA helicase
RV0060
RV0167: integral membrane protein YRBE1A
RV0169: MCE−family protein MCE1A
RV0172: MCE−family protein MCE1D
RV0173: MCE−family lipoprotein LprK
RV0184
RV0185
RV0303: dehydrogenase/reductase
RV0336: 13E12 repeat family protein
RV0490: putative two component sensor histidine kinase SENX3
RV0515: 13E12 repeat family protein
RV0605: resolvase
RV0606
RV0676C: transmembrane transport protein MmpL5
RV0829
RV0922: transposase
RV1122: 6−phosphogluconate dehydrogenase−like protein
RV1277
RV1278
RV1302: undecapaprenyl−phosphate alpha−N−acetylglucosaminyltransferase rfe (UDP−GlcNAC transferase)
RV1316C: methylated−DNA−−protein−cysteine methyltransferase
RV1406: methionyl−tRNA formyltransferase
RV1588C: REP13E12 repeat−containing protein
RV1633: excinuclease ABC subunit B
RV1638: excinuclease ABC subunit A
RV1702C
RV1765C
RV1833C: haloalkane dehalogenase
RV2015C
RV2024C
RV2100
RV2288
RV2578C
RV2579: haloalkane dehalogenase
RV2593C: Holliday junction DNA helicase RuvA
RV2594C: Holliday junction resolvase
RV2719C
RV2720: LexA repressor
RV2734
RV2735C
RV2736C: recombination regulator RecX
RV2737C: DNA recombination protein RecA
RV2789C: acyl−CoA dehydrogenase FADE21
RV2790C: lipid−transfer protein
RV2791C: transposase
RV2792C: resolvase
RV2968C: integral membrane protein
RV2976C: uracil−DNA glycosylase
RV2977C: thiamine monophosphate kinase
RV2978C: transposase
RV2979C: resolvase
RV2980
RV3022C: PPE family protein
RV3065: multidrugs−transport integral membrane protein MMR
RV3074
RV3076
RV3145: NADH dehydrogenase subunit A
RV3147: NADH dehydrogenase subunit C
RV3198C: ATP−dependent DNA helicase II UVRD2
RV3202C: ATP−dependent DNA helicase
RV3222C
RV3226C
RV3296: ATP−dependent helicase
RV3466
RV3585: DNA repair protein RadA
RV3586: DNA integrity scanning protein DisA
RV3776
RV3777: oxidoreductase
RV3827C: transposase

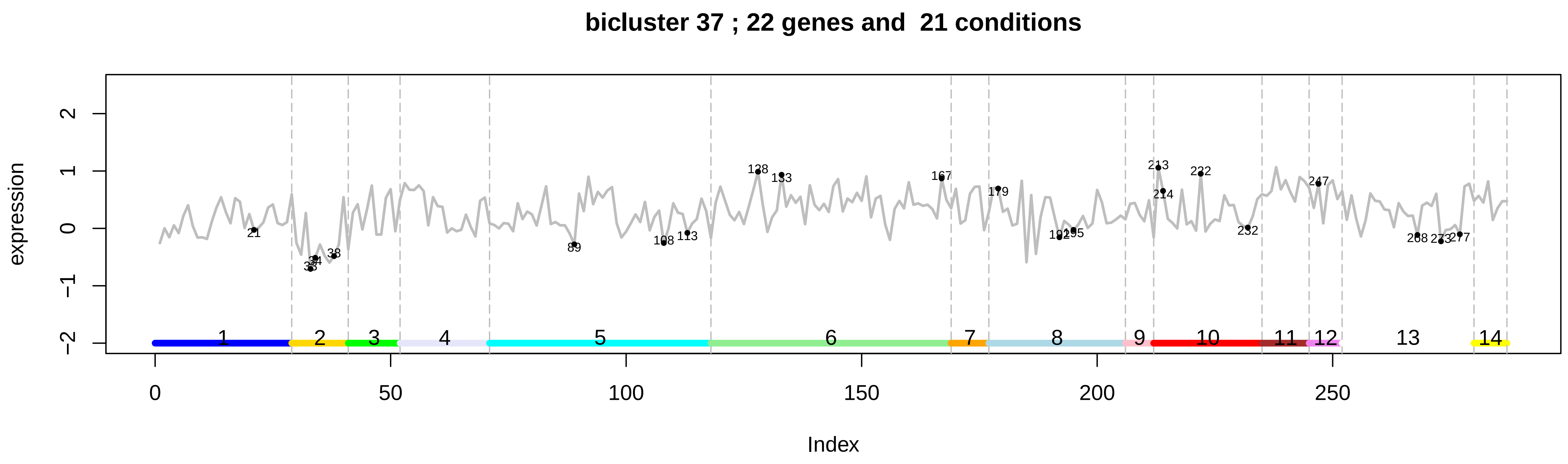
bicluster 37 ; 22 genes and 21 conditions
expression
Index
1
2
3
4
5
6
7
8
9
10
11
12
13
14

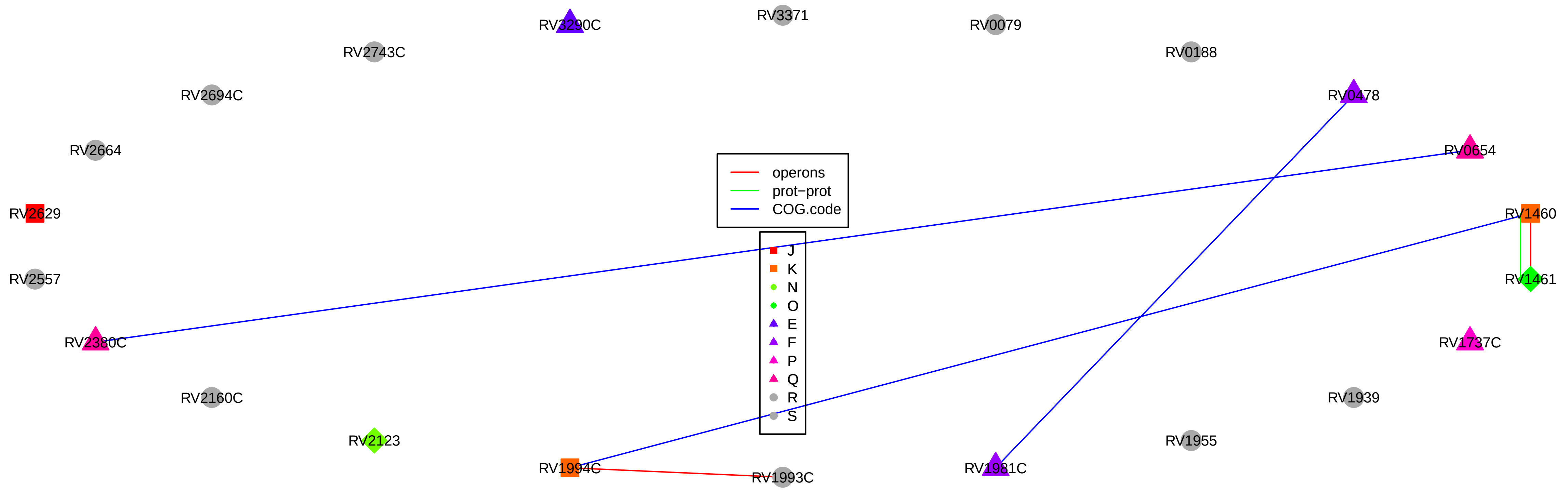
operons
prot−prot
COG.code
RV3290C
RV3371
RV0079
RV2743C
RV0188
RV2694C
RV0478
RV2664
RV0654
RV2629
RV1460
RV2557
RV1461
RV2380C
RV1737C
RV2160C
RV1939
RV2123
RV1955
RV1994C
RV1993C
RV1981C
J
K
N
O
E
F
P
Q
R
S

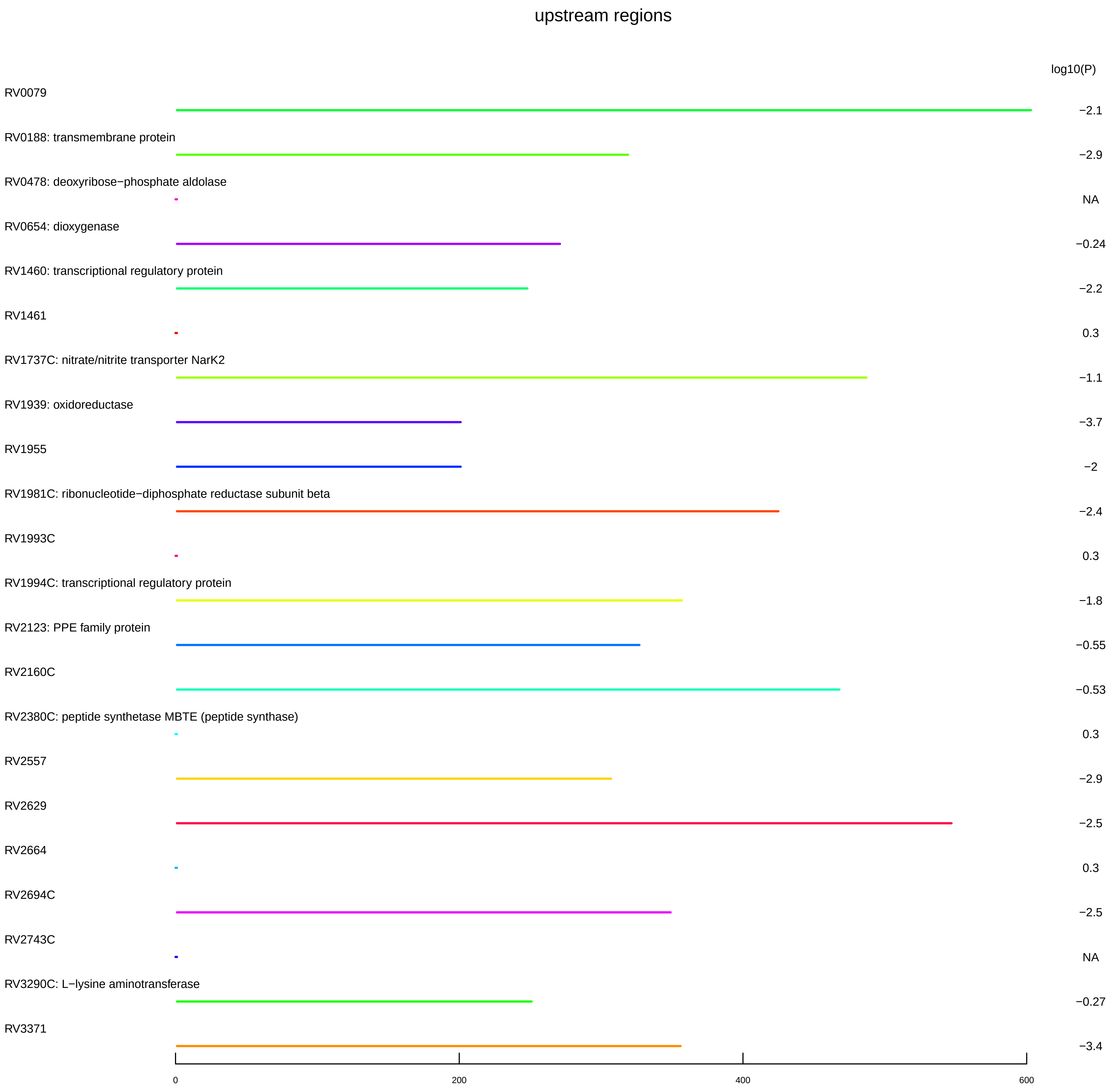
upstream regions
log10(P)
RV0079
−2.1
RV0188: transmembrane protein
−2.9
RV0478: deoxyribose−phosphate aldolase
NA
RV0654: dioxygenase
−0.24
RV1460: transcriptional regulatory protein
−2.2
RV1461
0.3
RV1737C: nitrate/nitrite transporter NarK2
−1.1
RV1939: oxidoreductase
−3.7
RV1955
−2
RV1981C: ribonucleotide−diphosphate reductase subunit beta
−2.4
RV1993C
0.3
RV1994C: transcriptional regulatory protein
−1.8
RV2123: PPE family protein
−0.55
RV2160C
−0.53
RV2380C: peptide synthetase MBTE (peptide synthase)
0.3
RV2557
−2.9
RV2629
−2.5
RV2664
0.3
RV2694C
−2.5
RV2743C
NA
RV3290C: L−lysine aminotransferase
−0.27
RV3371
−3.4
0
200
400
600

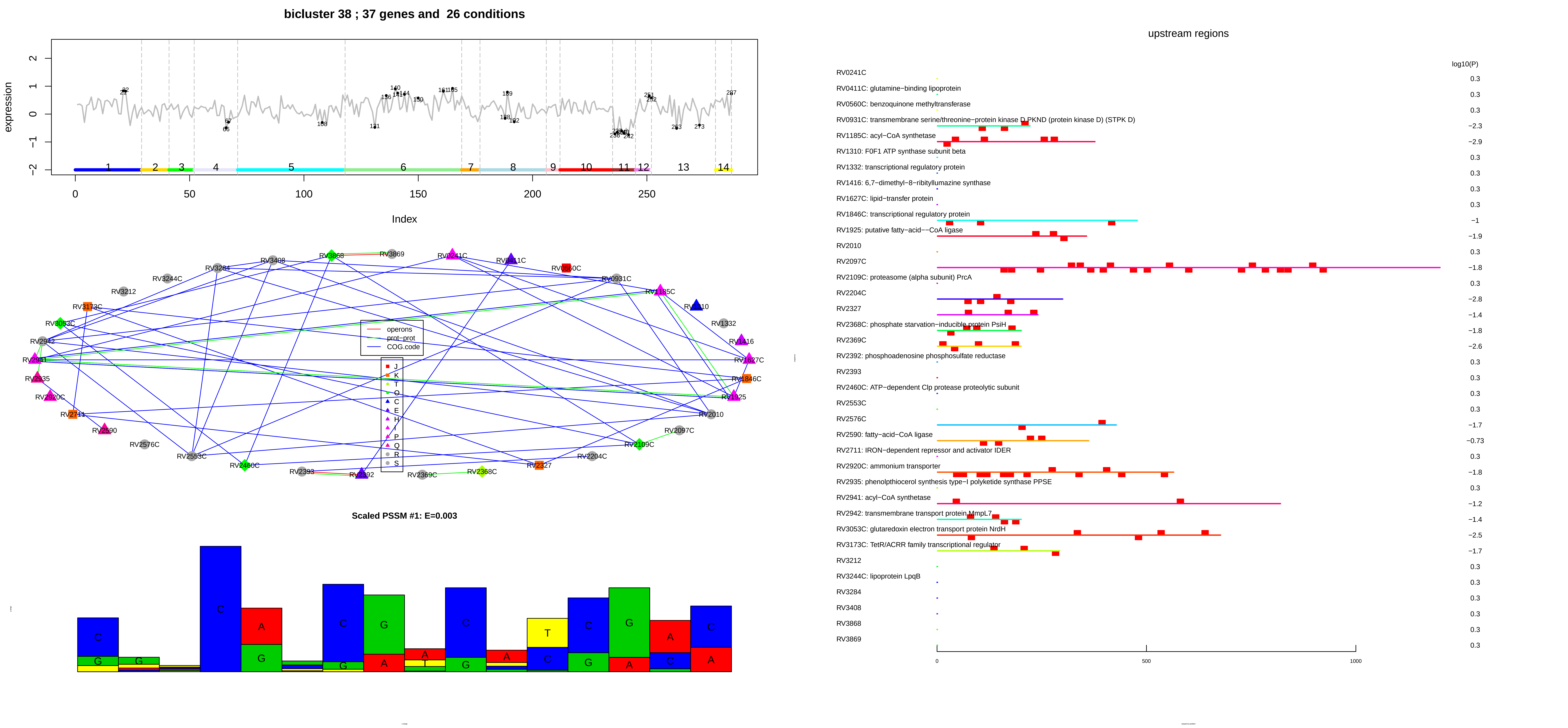
bicluster 38 ; 37 genes and 26 conditions
expression
Index
operons
prot−prot
COG.code
Scaled PSSM #1: E=0.003
upstream regions
log10(P)
RV0241C
RV0411C: glutamine−binding lipoprotein
RV0560C: benzoquinone methyltransferase
RV0931C: transmembrane serine/threonine−protein kinase D PKND (protein kinase D) (STPK D)
RV1185C: acyl−CoA synthetase
RV1310: F0F1 ATP synthase subunit beta
RV1332: transcriptional regulatory protein
RV1416: 6,7−dimethyl−8−ribityllumazine synthase
RV1627C: lipid−transfer protein
RV1846C: transcriptional regulatory protein
RV1925: putative fatty−acid−−CoA ligase
RV2010
RV2097C
RV2109C: proteasome (alpha subunit) PrcA
RV2204C
RV2327
RV2368C: phosphate starvation−inducible protein PsiH
RV2369C
RV2392: phosphoadenosine phosphosulfate reductase
RV2393
RV2460C: ATP−dependent Clp protease proteolytic subunit
RV2553C
RV2576C
RV2590: fatty−acid−CoA ligase
RV2711: IRON−dependent repressor and activator IDER
RV2920C: ammonium transporter
RV2935: phenolpthiocerol synthesis type−I polyketide synthase PPSE
RV2941: acyl−CoA synthetase
RV2942: transmembrane transport protein MmpL7
RV3053C: glutaredoxin electron transport protein NrdH
RV3173C: TetR/ACRR family transcriptional regulator
RV3212
RV3244C: lipoprotein LpqB
RV3284
RV3408
RV3868
RV3869

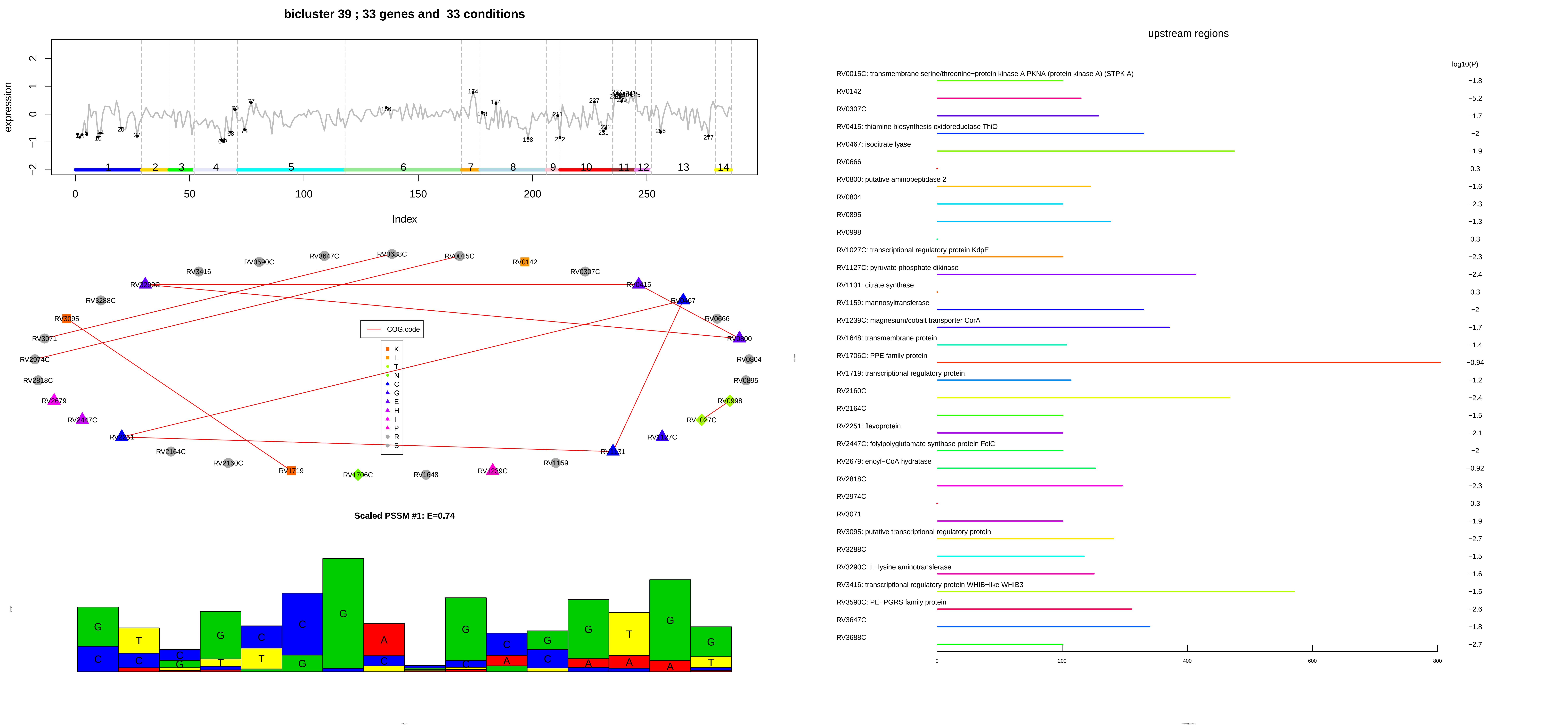
bicluster 39 ; 33 genes and 33 conditions
expression
Index
Scaled PSSM #1: E=0.74
COG.code
upstream regions
log10(P)
RV0015C: transmembrane serine/threonine−protein kinase A PKNA (protein kinase A) (STPK A)
RV0142
RV0307C
RV0415: thiamine biosynthesis oxidoreductase ThiO
RV0467: isocitrate lyase
RV0666
RV0800: putative aminopeptidase 2
RV0804
RV0895
RV0998
RV1027C: transcriptional regulatory protein KdpE
RV1127C: pyruvate phosphate dikinase
RV1131: citrate synthase
RV1159: mannosyltransferase
RV1239C: magnesium/cobalt transporter CorA
RV1648: transmembrane protein
RV1706C: PPE family protein
RV1719: transcriptional regulatory protein
RV2160C
RV2164C
RV2251: flavoprotein
RV2447C: folylpolyglutamate synthase protein FolC
RV2679: enoyl−CoA hydratase
RV2818C
RV2974C
RV3071
RV3095: putative transcriptional regulatory protein
RV3288C
RV3290C: L−lysine aminotransferase
RV3416: transcriptional regulatory protein WHIB−like WHIB3
RV3590C: PE−PGRS family protein
RV3647C
RV3688C
−1.8
−5.2
−1.7
−2
−1.9
0.3
−1.6
−2.3
−1.3
0.3
−2.3
−2.4
0.3
−2
−1.7
−1.4
−0.94
−1.2
−2.4
−1.5
−2.1
−2
−0.92
−2.3
0.3
−1.9
−2.7
−1.5
−1.6
−1.5
−2.6
−1.8
−2.7

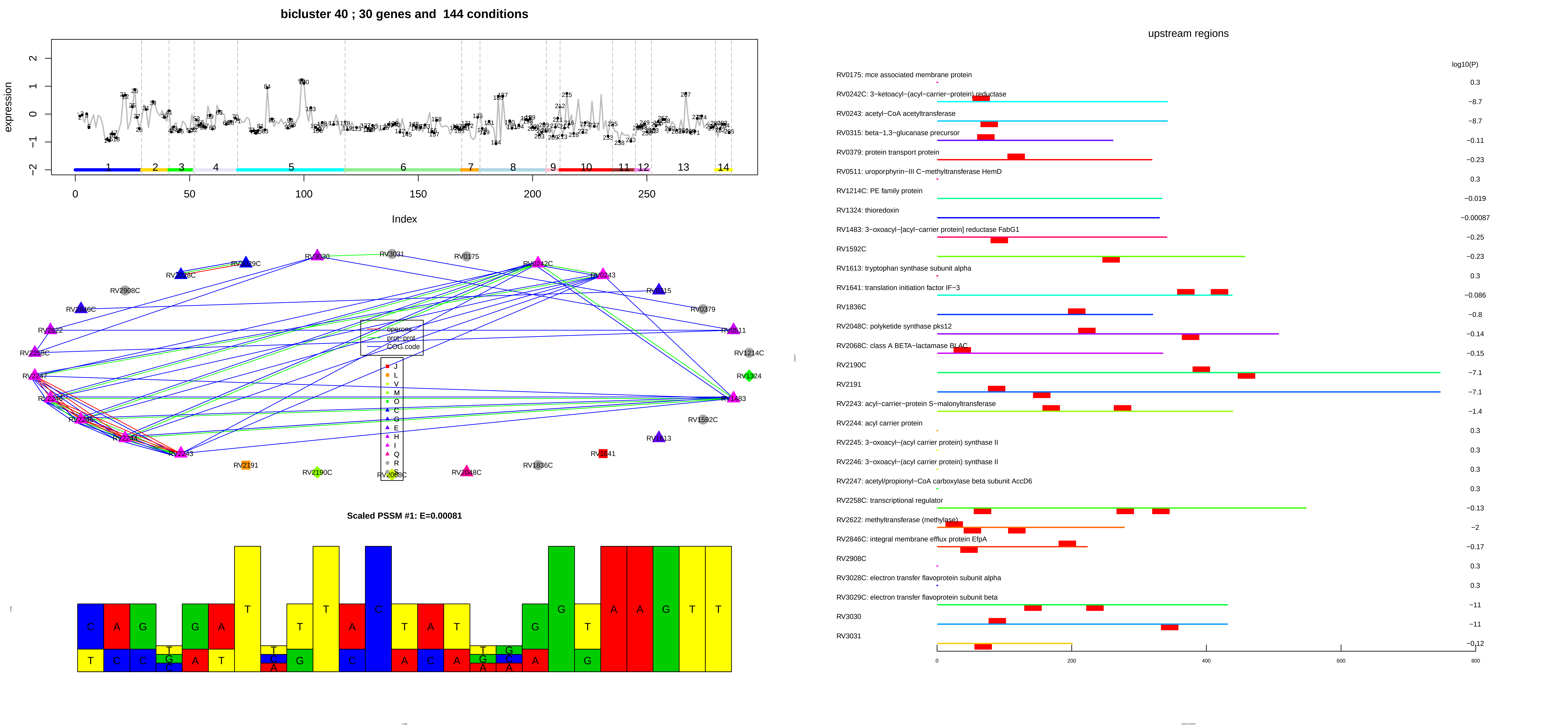
bicluster 40 ; 30 genes and 144 conditions
expression
Index
Scaled PSSM #1: E=0.00081
upstream regions
log10(P)
RV0175: mce associated membrane protein
RV0242C: 3−ketoacyl−(acyl−carrier−protein) reductase
RV0243: acetyl−CoA acetyltransferase
RV0315: beta−1,3−glucanase precursor
RV0379: protein transport protein
RV0511: uroporphyrin−III C−methyltransferase HemD
RV1214C: PE family protein
RV1324: thioredoxin
RV1483: 3−oxoacyl−[acyl−carrier protein] reductase FabG1
RV1592C
RV1613: tryptophan synthase subunit alpha
RV1641: translation initiation factor IF−3
RV1836C
RV2048C: polyketide synthase pks12
RV2068C: class A BETA−lactamase BLAC
RV2190C
RV2191
RV2243: acyl−carrier−protein S−malonyltransferase
RV2244: acyl carrier protein
RV2245: 3−oxoacyl−(acyl carrier protein) synthase II
RV2246: 3−oxoacyl−(acyl carrier protein) synthase II
RV2247: acetyl/propionyl−CoA carboxylase beta subunit AccD6
RV2258C: transcriptional regulator
RV2622: methyltransferase (methylase)
RV2846C: integral membrane efflux protein EfpA
RV2908C
RV3028C: electron transfer flavoprotein subunit alpha
RV3029C: electron transfer flavoprotein subunit beta
RV3030
RV3031
0.3
−8.7
−8.7
−0.11
−0.23
0.3
−0.019
−0.00087
−0.25
−0.23
0.3
−0.086
−0.8
−0.14
−0.15
−7.1
−7.1
−1.4
0.3
0.3
0.3
0.3
−0.13
−2
−0.17
0.3
0.3
−11
−11
−0.12
operons
prot−prot
COG.code

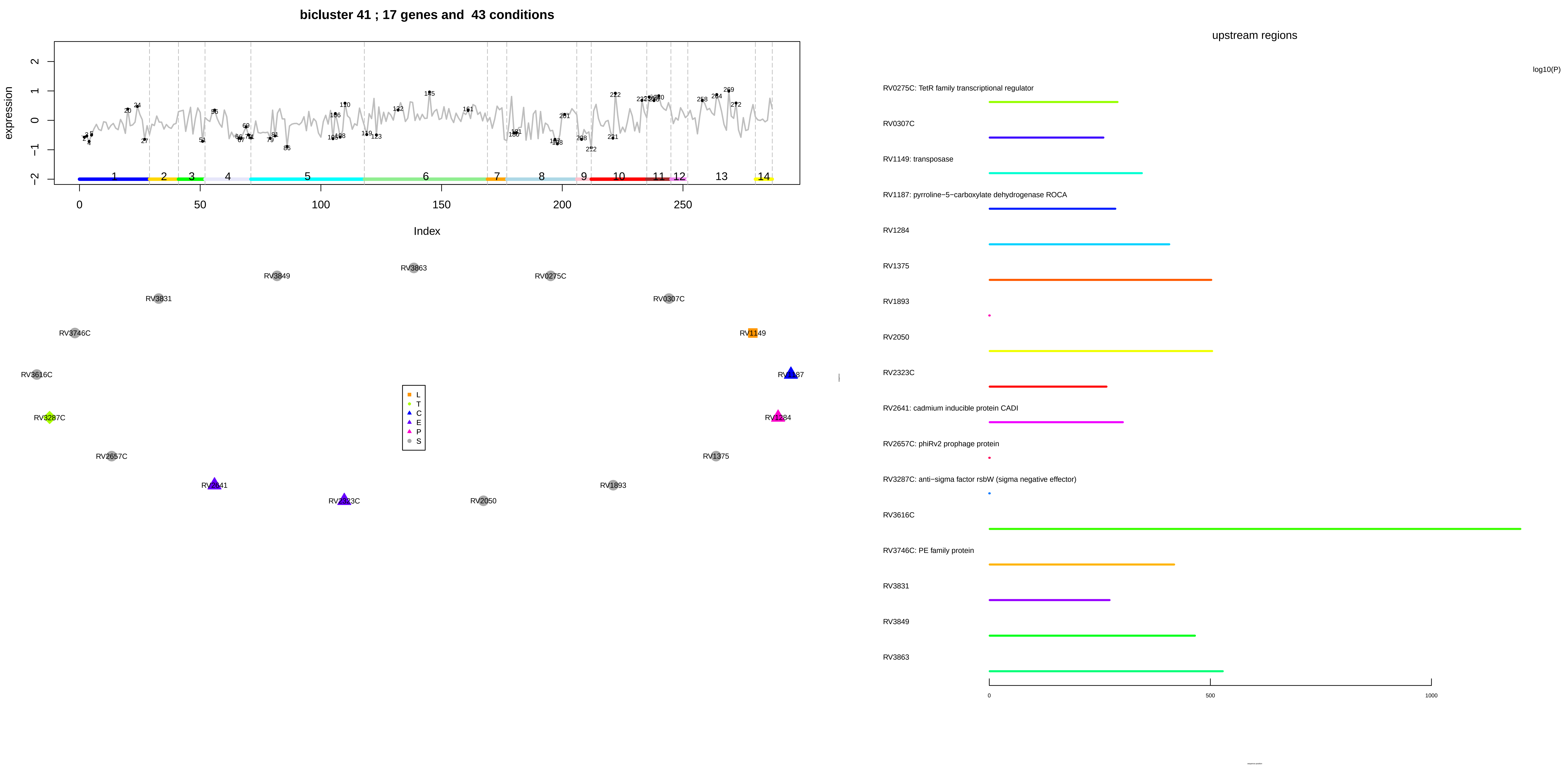

bicluster 41 ; 17 genes and 43 conditions
expression
Index
upstream regions
log10(P)
RV0275C: TetR family transcriptional regulator
RV0307C
RV1149: transposase
RV1187: pyrroline−5−carboxylate dehydrogenase ROCA
RV1284
RV1375
RV1893
RV2050
RV2323C
RV2641: cadmium inducible protein CADI
RV2657C: phiRv2 prophage protein
RV3287C: anti−sigma factor rsbW (sigma negative effector)
RV3616C
RV3746C: PE family protein
RV3831
RV3849
RV3863
L
T
C
E
P
S
RV3849
RV3863
RV0275C
RV3831
RV0307C
RV3746C
RV1149
RV3616C
RV3287C
RV1284
RV2657C
RV1375
RV1893
RV2050

bicluster 42 ; 25 genes and 54 conditions

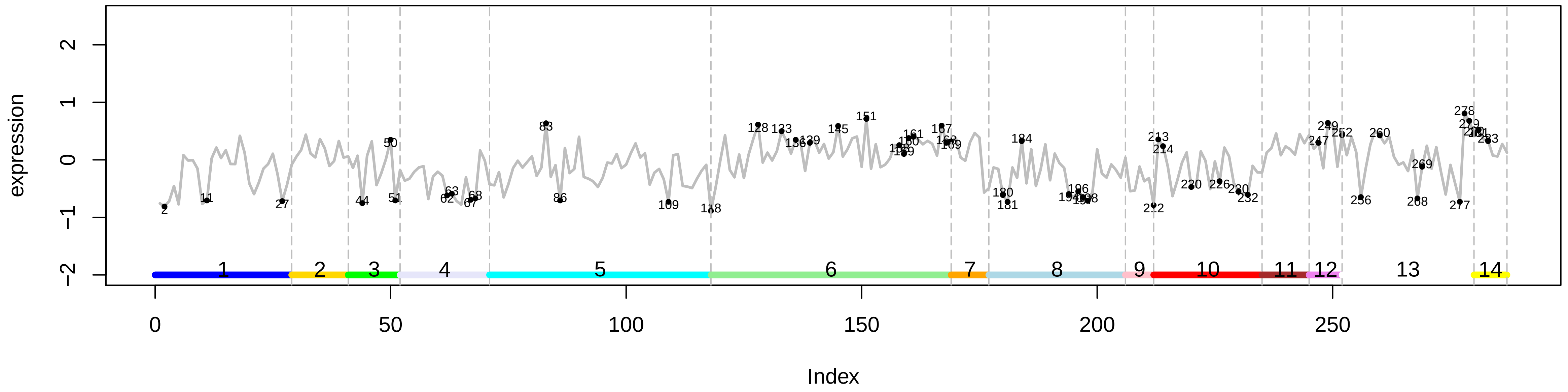

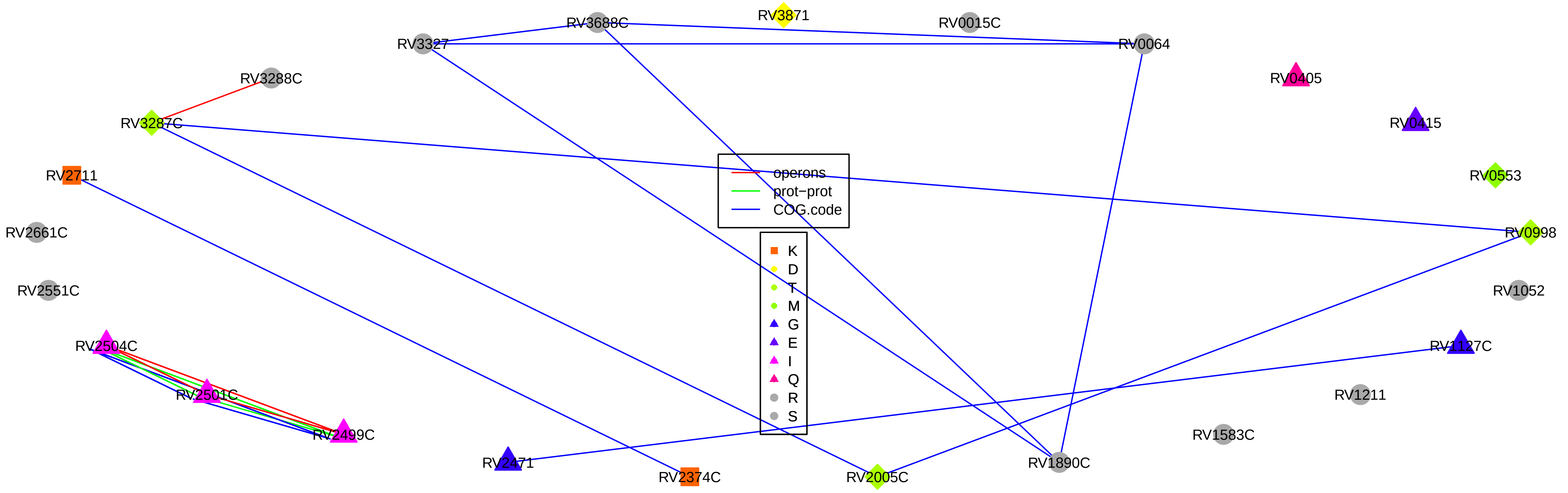

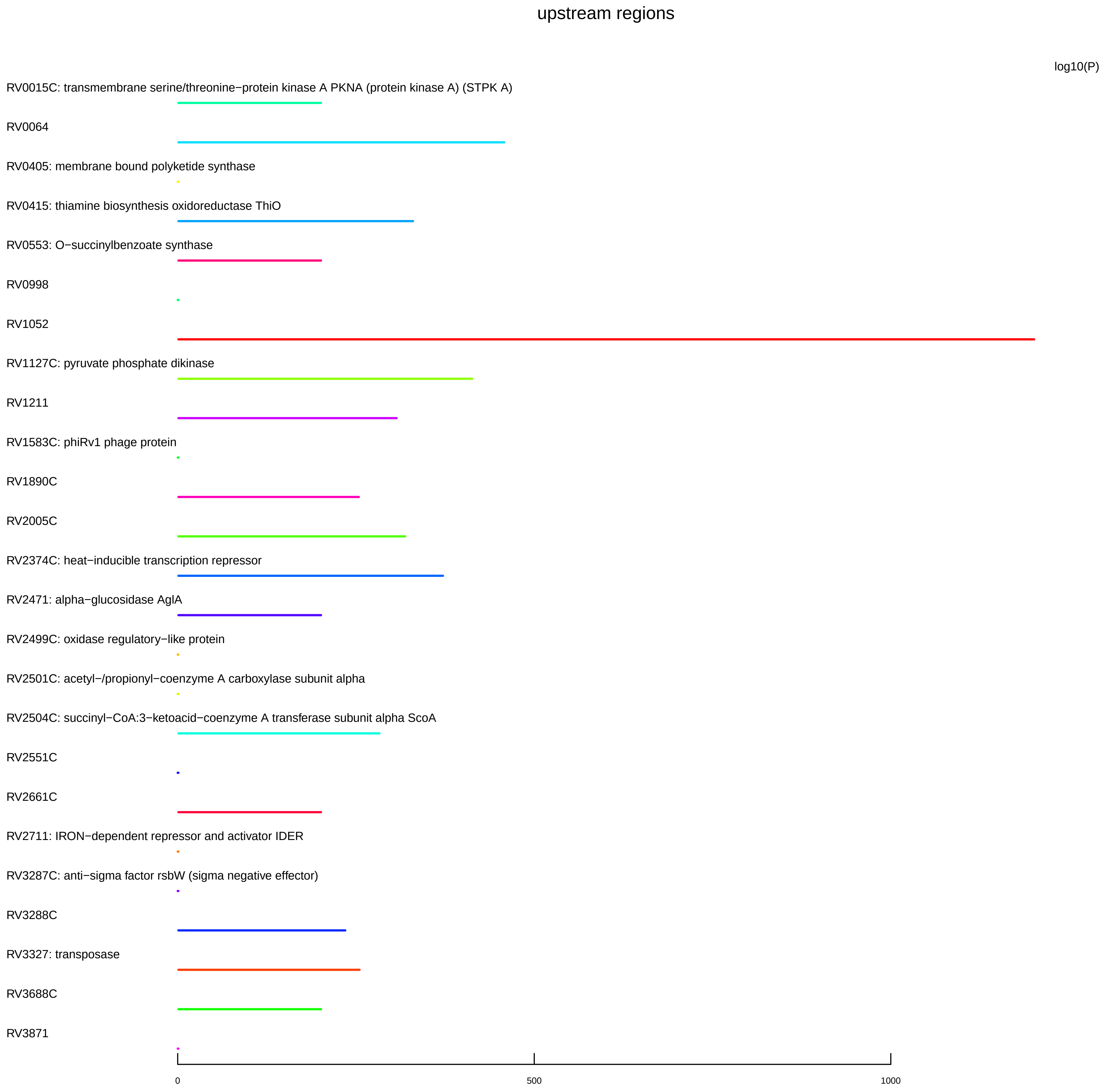

bicluster 43 ; 29 genes and 131 conditions

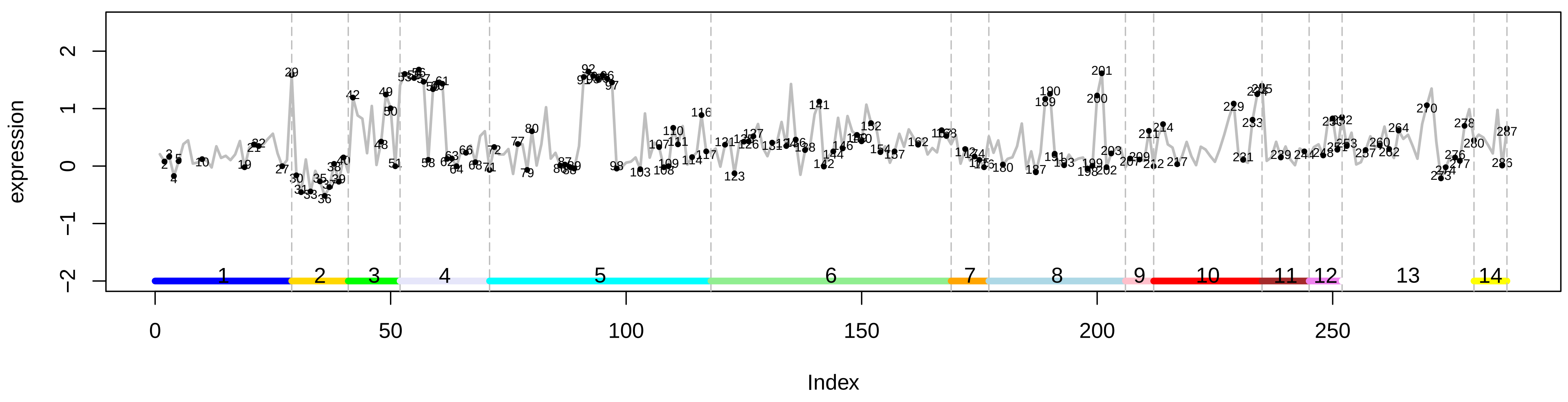

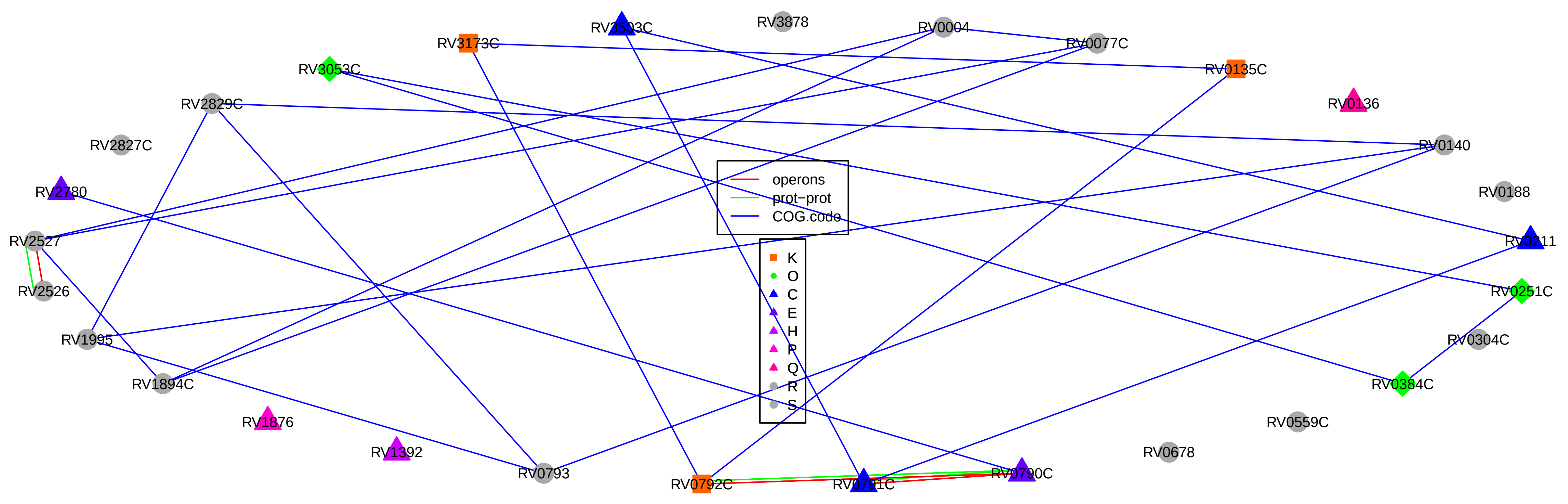

upstream regions

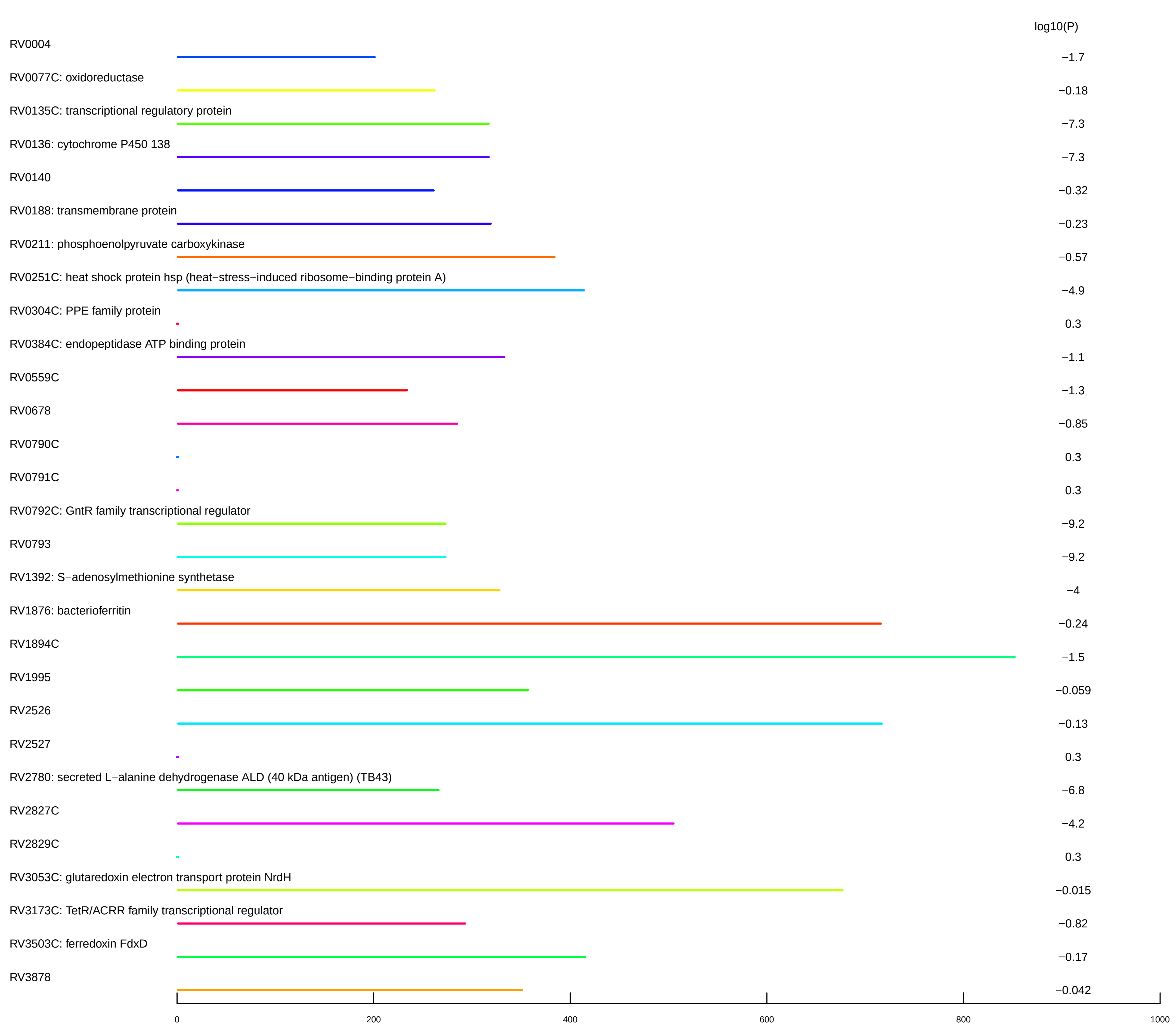

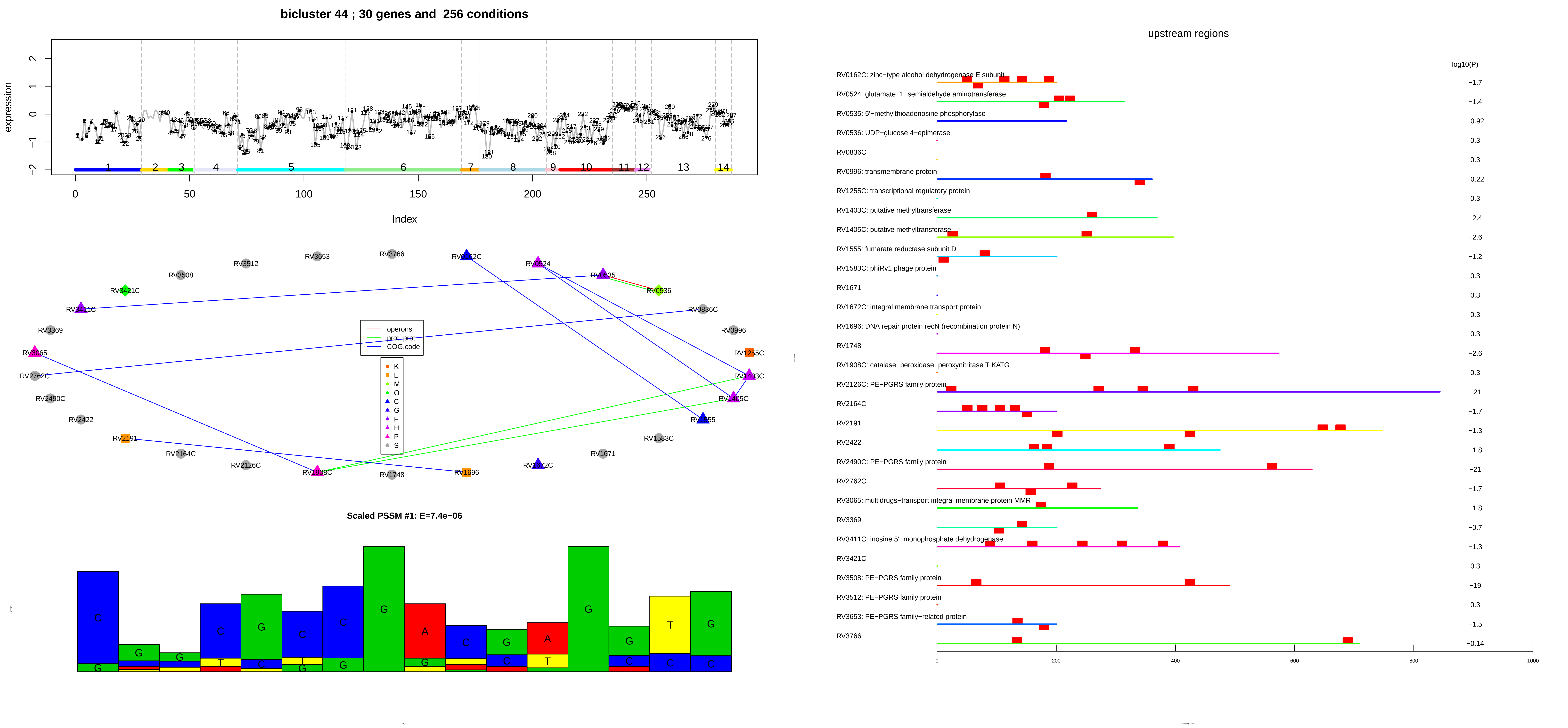

bicluster 44 ; 30 genes and 256 conditions
expression
Index
operons
prot−prot
COG.code
K
L
M
O
C
G
F
H
P
S
Scaled PSSM #1: E=7.4e−06
upstream regions
log10(P)
RV0162C: zinc−type alcohol dehydrogenase E subunit −1.7
RV0524: glutamate−1−semialdehyde aminotransferase −1.4
RV0535: 5'−methylthioadenosine phosphorylase −0.92
RV0536: UDP−glucose 4−epimerase 0.3
RV0836C 0.3
RV0996: transmembrane protein −0.22
RV1255C: transcriptional regulatory protein 0.3
RV1403C: putative methyltransferase −2.4
RV1405C: putative methyltransferase −2.6
RV1555: fumarate reductase subunit D −1.2
RV1583C: phiRv1 phage protein 0.3
RV1671 0.3
RV1672C: integral membrane transport protein 0.3
RV1696: DNA repair protein recN (recombination protein N) 0.3
RV1748 −2.6
RV1908C: catalase−peroxidase−peroxynitritase T KATG 0.3
RV2126C: PE−PGRS family protein −21
RV2164C −1.7
RV2191 −1.3
RV2422 −1.8
RV2490C: PE−PGRS family protein −21
RV2762C −1.7
RV3065: multidrugs−transport integral membrane protein MMR −1.8
RV3369 −0.7
RV3411C: inosine 5'−monophosphate dehydrogenase −1.3
RV3421C 0.3
RV3508: PE−PGRS family protein −19
RV3512: PE−PGRS family protein 0.3
RV3653: PE−PGRS family−related protein −1.5
RV3766 −0.14

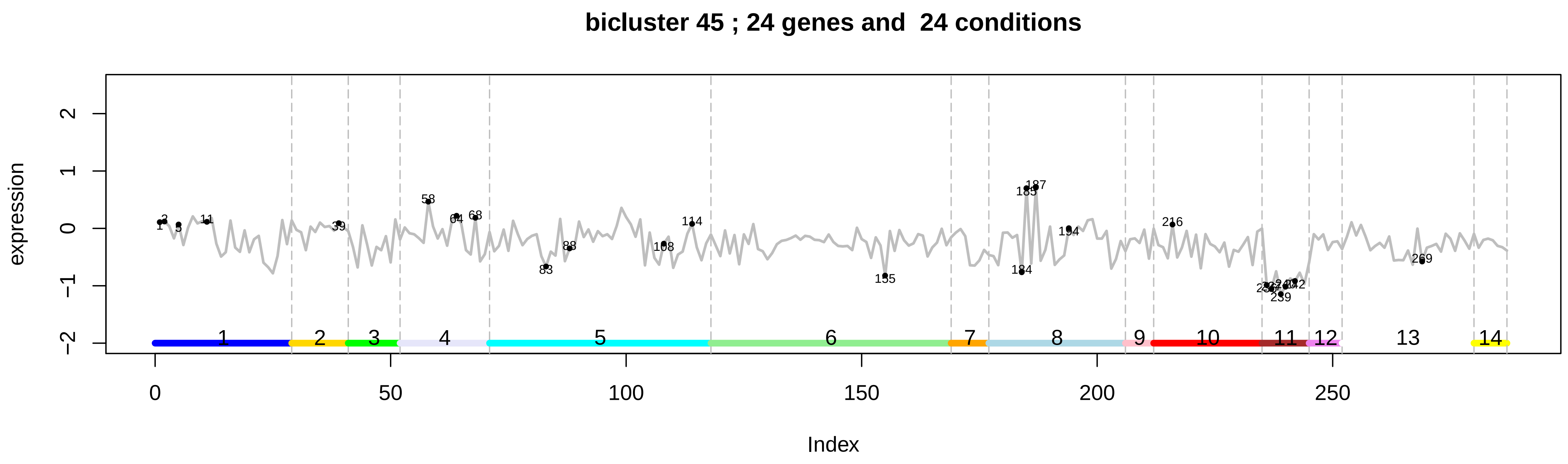
bicluster 45 ; 24 genes and 24 conditions
expression
Index
1
2
3
4
5
6
7
8
9
10
11
12
13
14

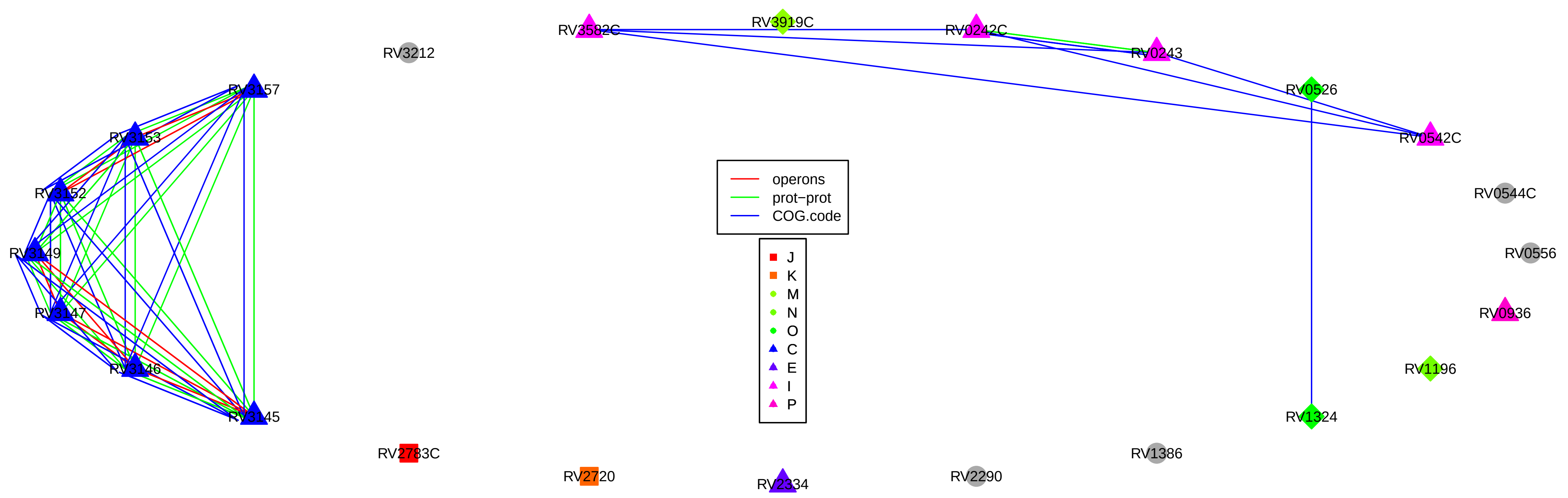
operons
prot−prot
COG.code
J
K
M
N
O
C
E
I
P
RV3212
RV3582C
RV3919C
RV0242C
RV0243
RV0526
RV0542C
RV0544C
RV0556
RV0936
RV1196
RV1324
RV1386
RV2290
RV2334
RV2720
RV2783C

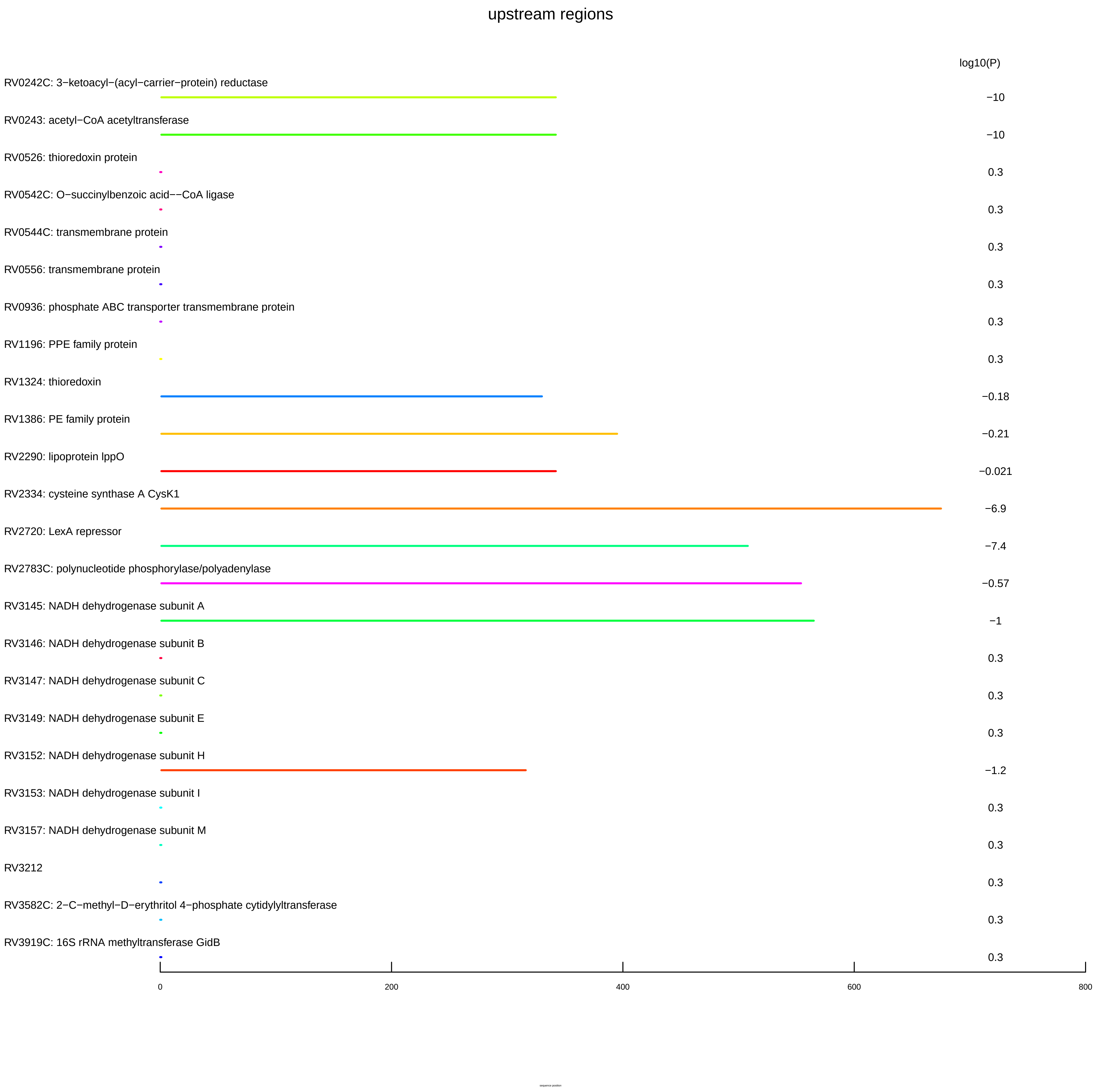
upstream regions
log10(P)
RV0242C: 3−ketoacyl−(acyl−carrier−protein) reductase
RV0243: acetyl−CoA acetyltransferase
RV0526: thioredoxin protein
RV0542C: O−succinylbenzoic acid−−CoA ligase
RV0544C: transmembrane protein
RV0556: transmembrane protein
RV0936: phosphate ABC transporter transmembrane protein
RV1196: PPE family protein
RV1324: thioredoxin
RV1386: PE family protein
RV2290: lipoprotein lppO
RV2334: cysteine synthase A CysK1
RV2720: LexA repressor
RV2783C: polynucleotide phosphorylase/polyadenylase
RV3145: NADH dehydrogenase subunit A
RV3146: NADH dehydrogenase subunit B
RV3147: NADH dehydrogenase subunit C
RV3149: NADH dehydrogenase subunit E
RV3152: NADH dehydrogenase subunit H
RV3153: NADH dehydrogenase subunit I
RV3157: NADH dehydrogenase subunit M
RV3212
RV3582C: 2−C−methyl−D−erythritol 4−phosphate cytidylyltransferase
RV3919C: 16S rRNA methyltransferase GidB
−10
−10
0.3
0.3
0.3
0.3
0.3
0.3
−0.18
−0.21
−0.021
−6.9
−7.4
−0.57
−1
0.3
0.3
0.3
−1.2
0.3
0.3
0.3
0.3
0.3

bicluster 46 ; 39 genes and 264 conditions

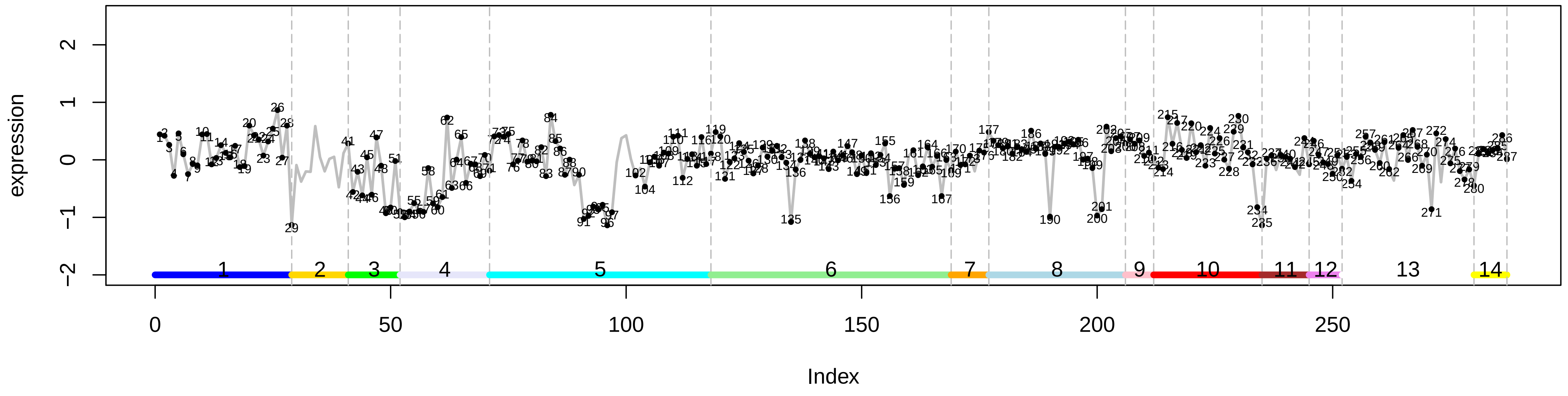

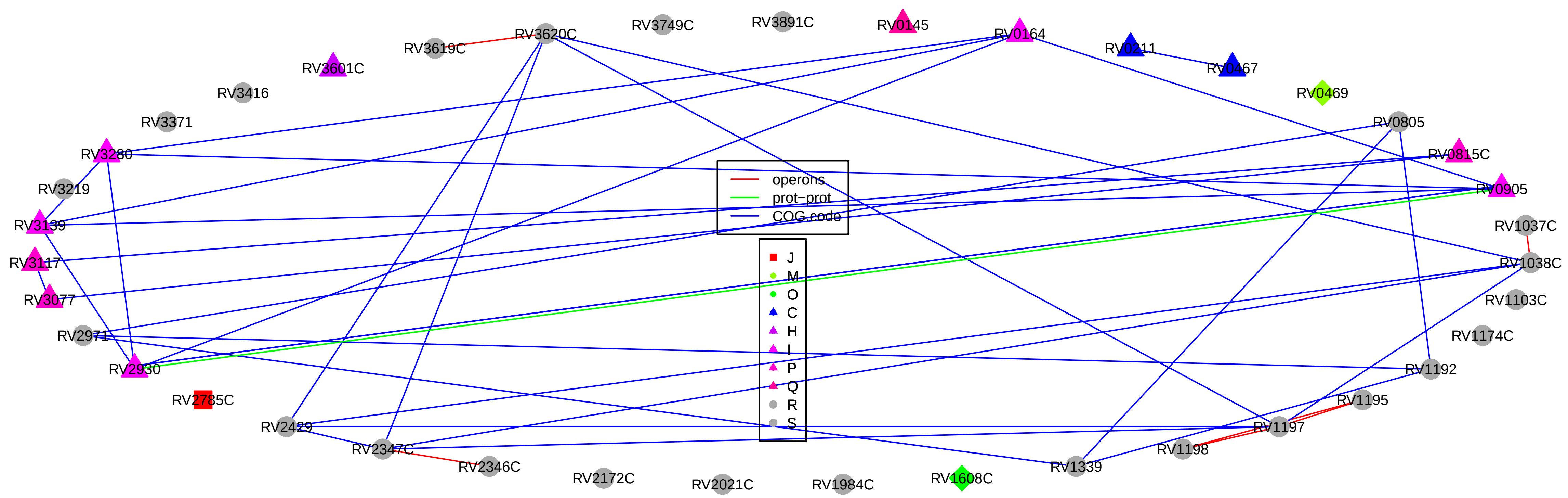

Scaled PSSM #1: E=3.7e−05

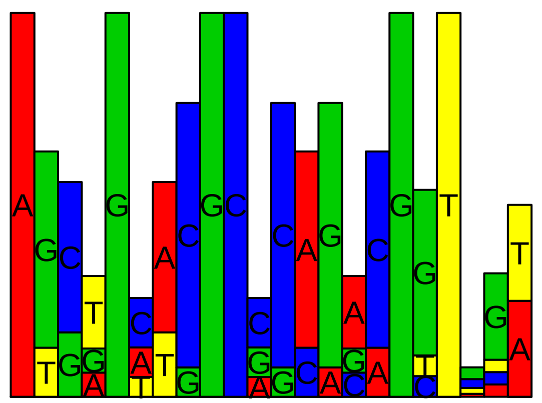

Scaled PSSM #2: E=8.7e−05

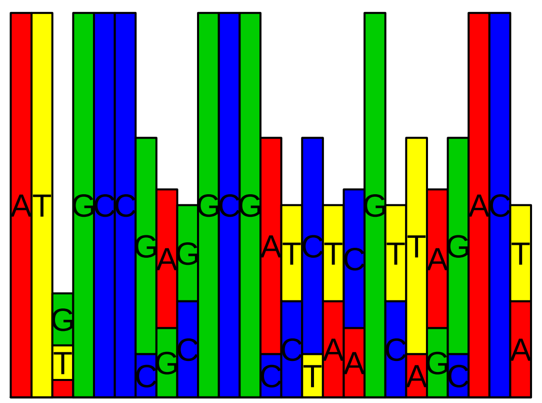

Scaled PSSM #3: E=0.012

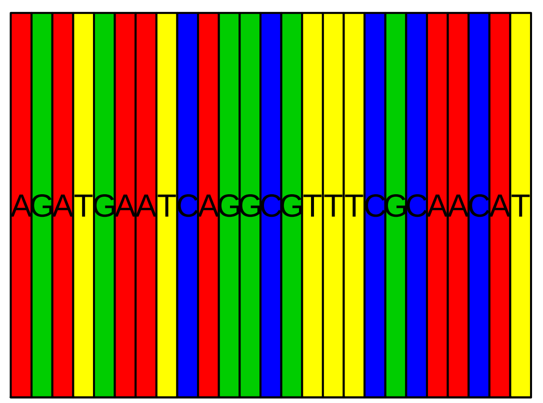

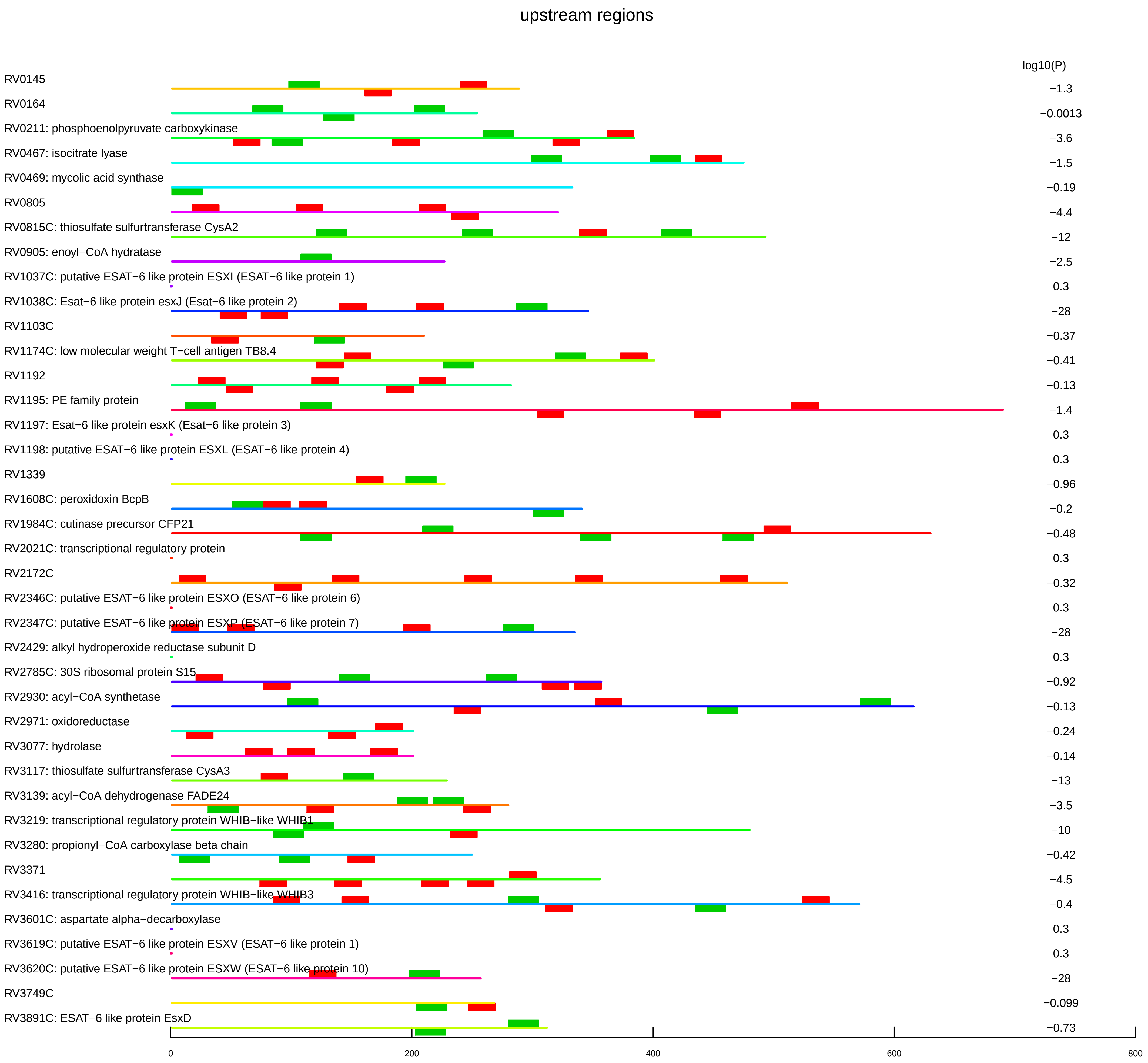

bicluster 47 ; 50 genes and 207 conditions

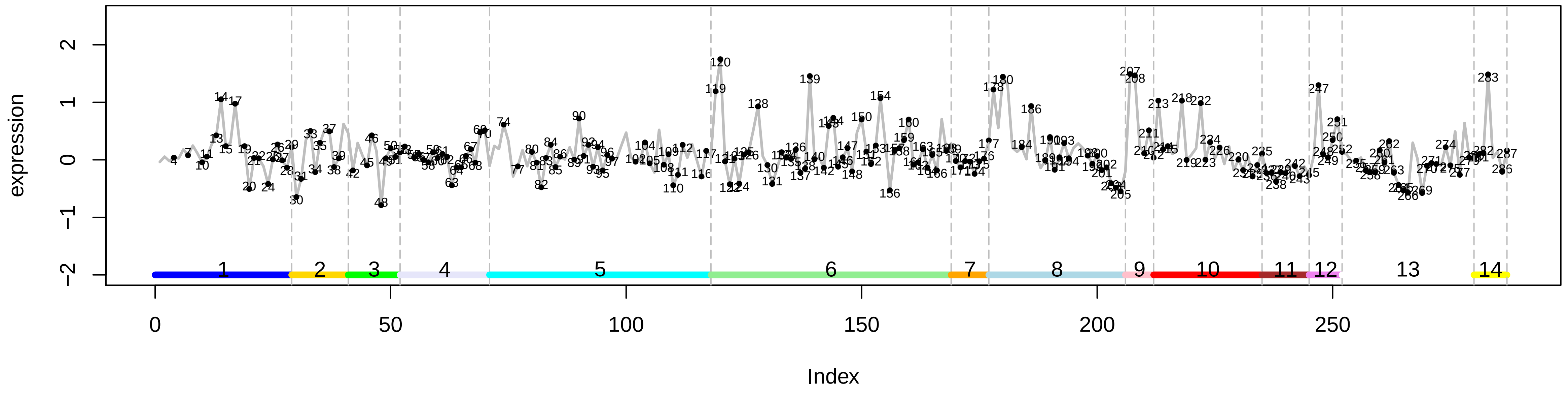

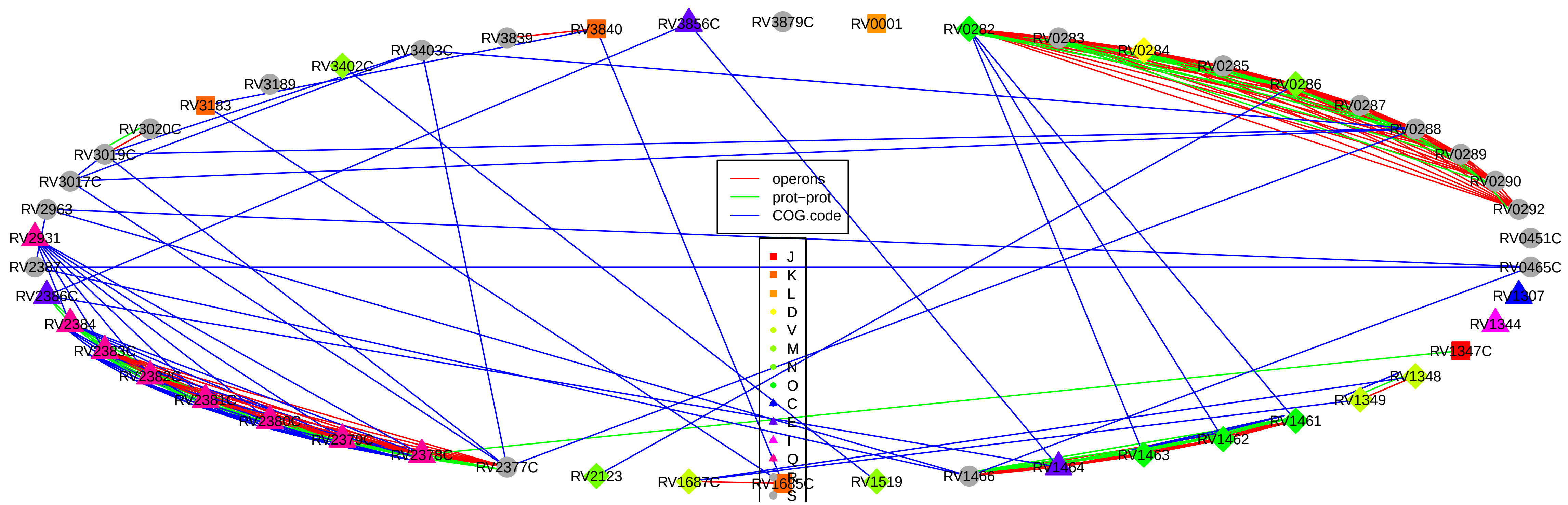

Scaled PSSM #1: E=1.8e−23

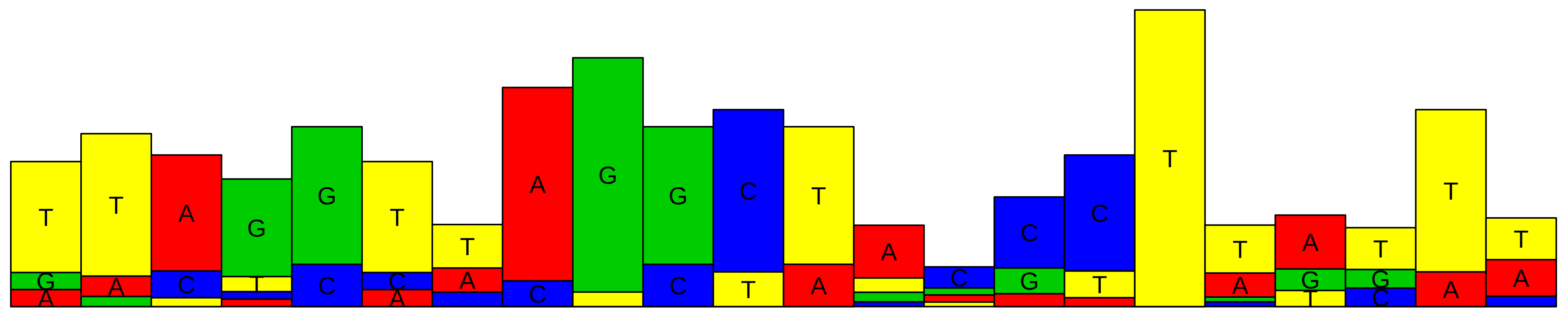

upstream regions

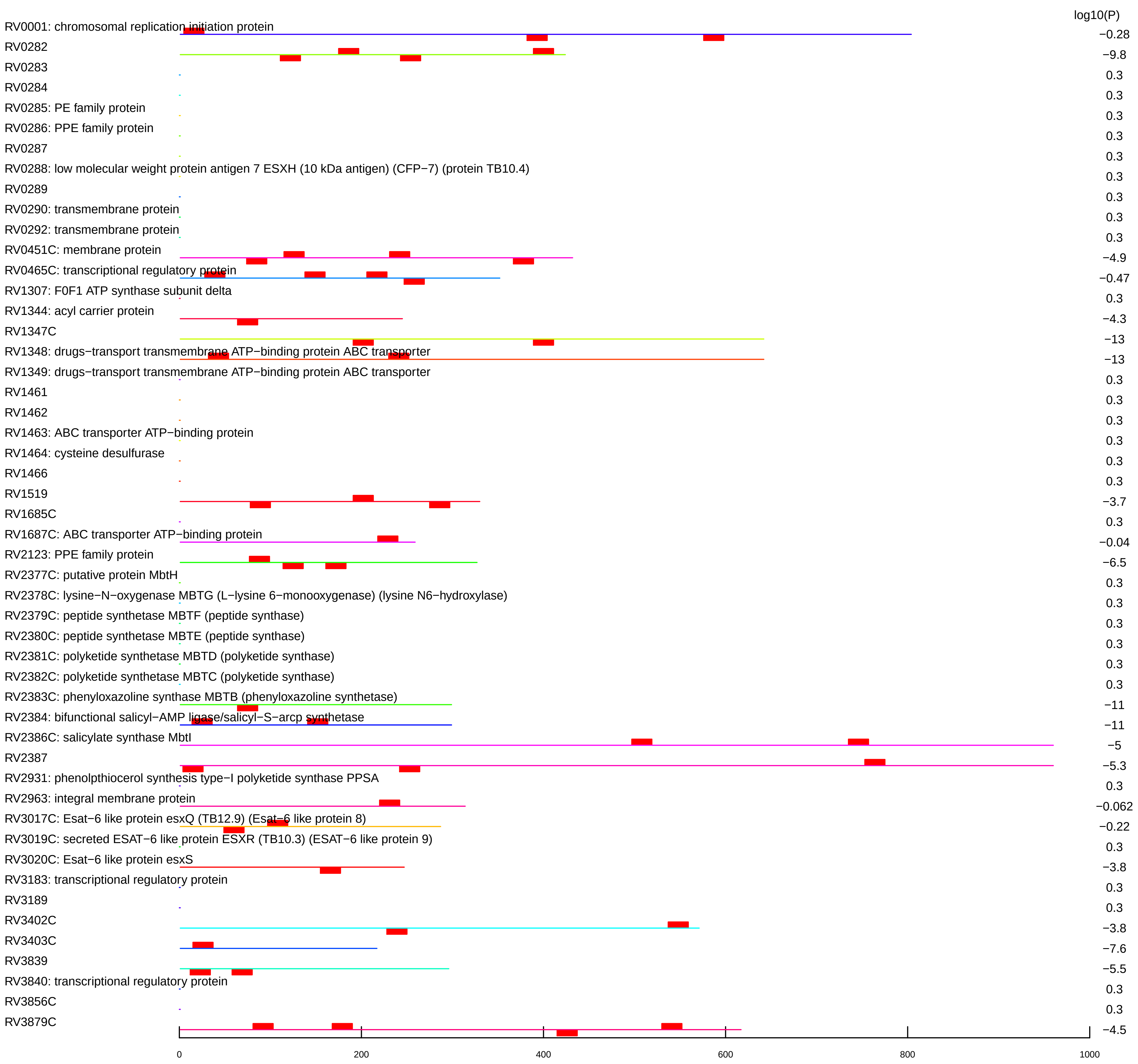

bicluster 48 ; 9 genes and 41 conditions

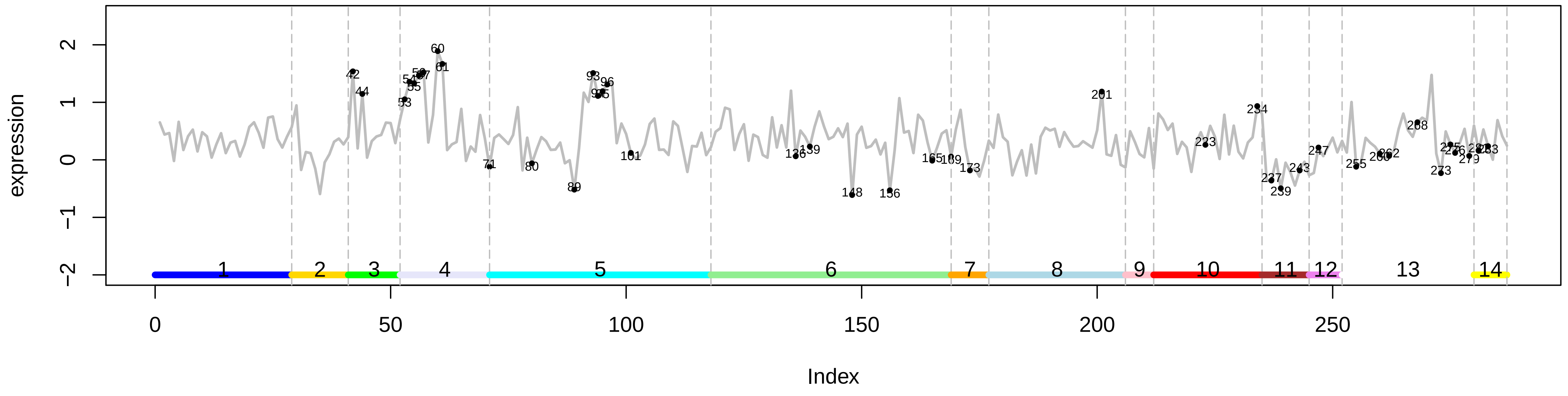

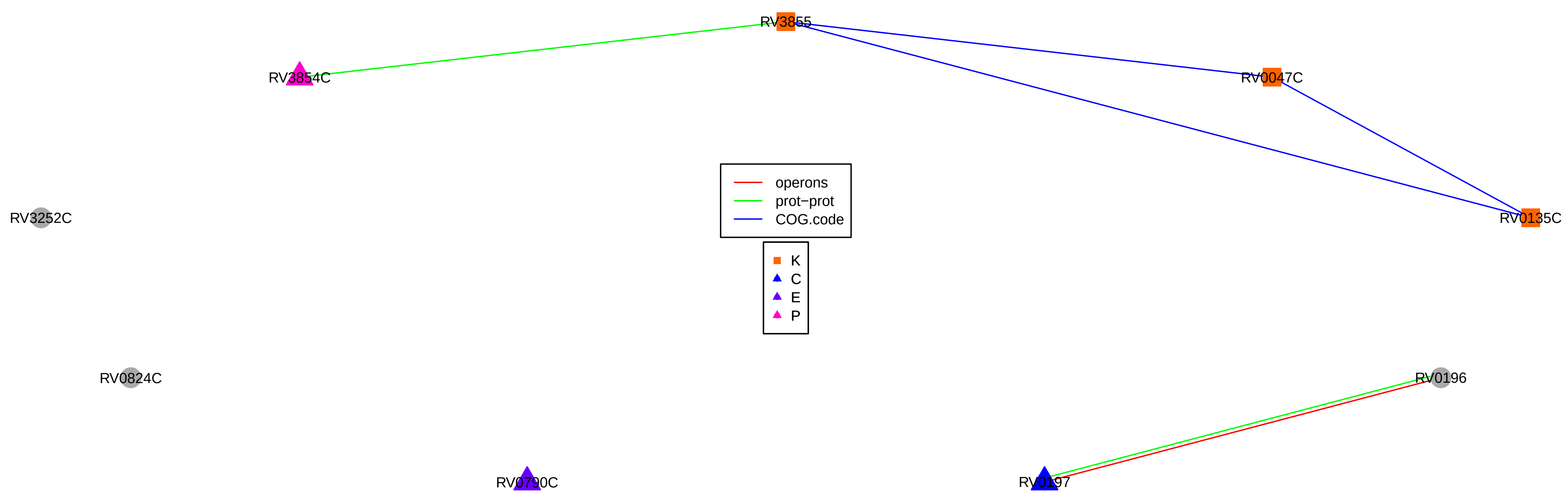

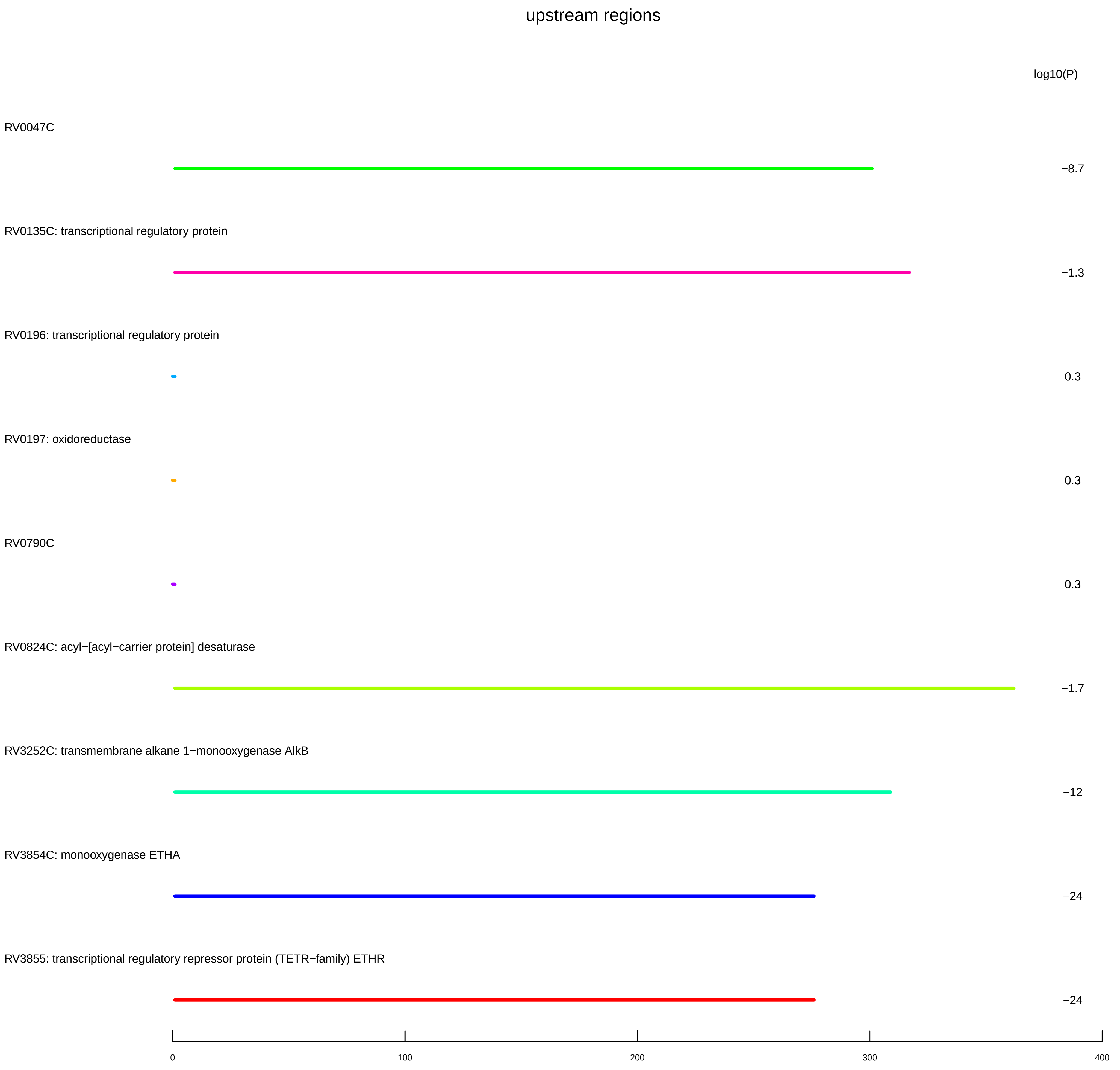

bicluster 49 ; 65 genes and 71 conditions

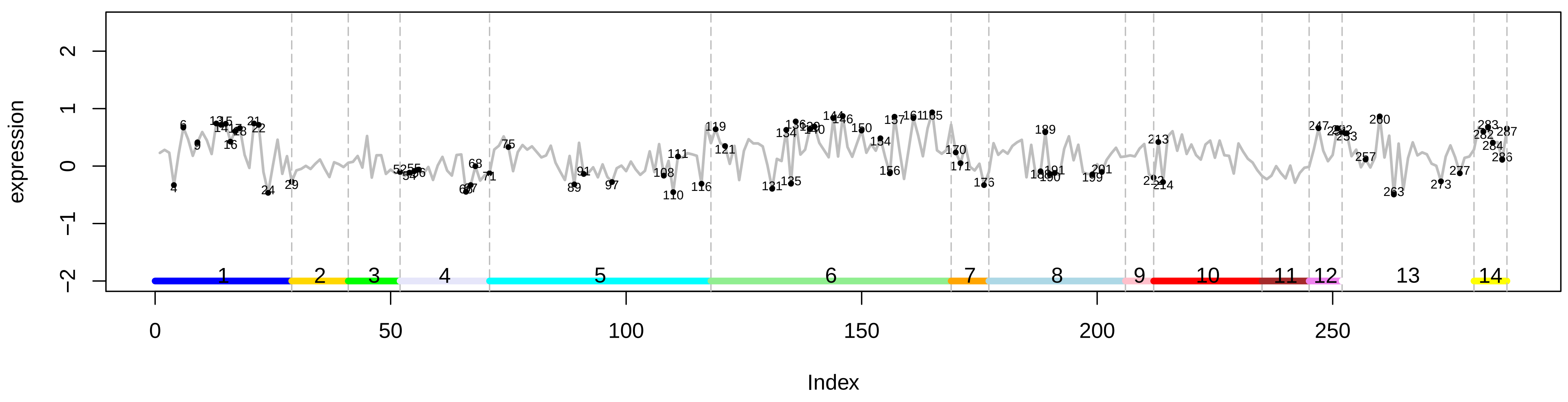

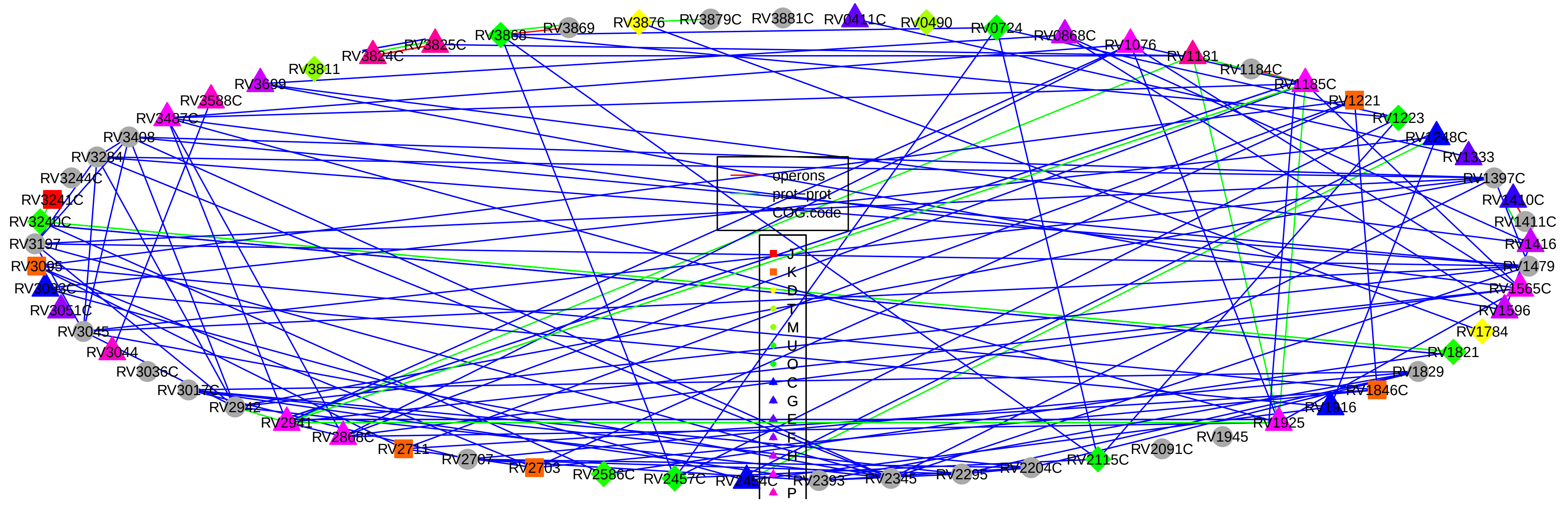

Scaled PSSM #1: E=3.3e−06

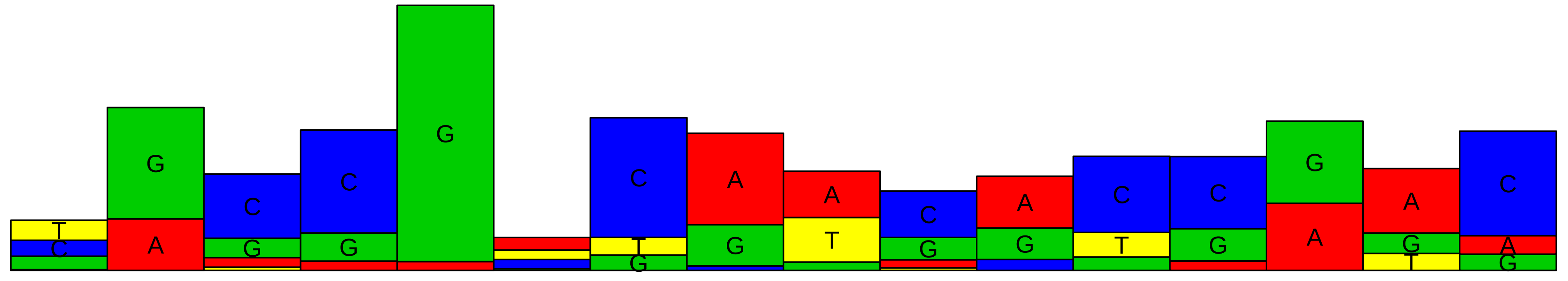

upstream regions

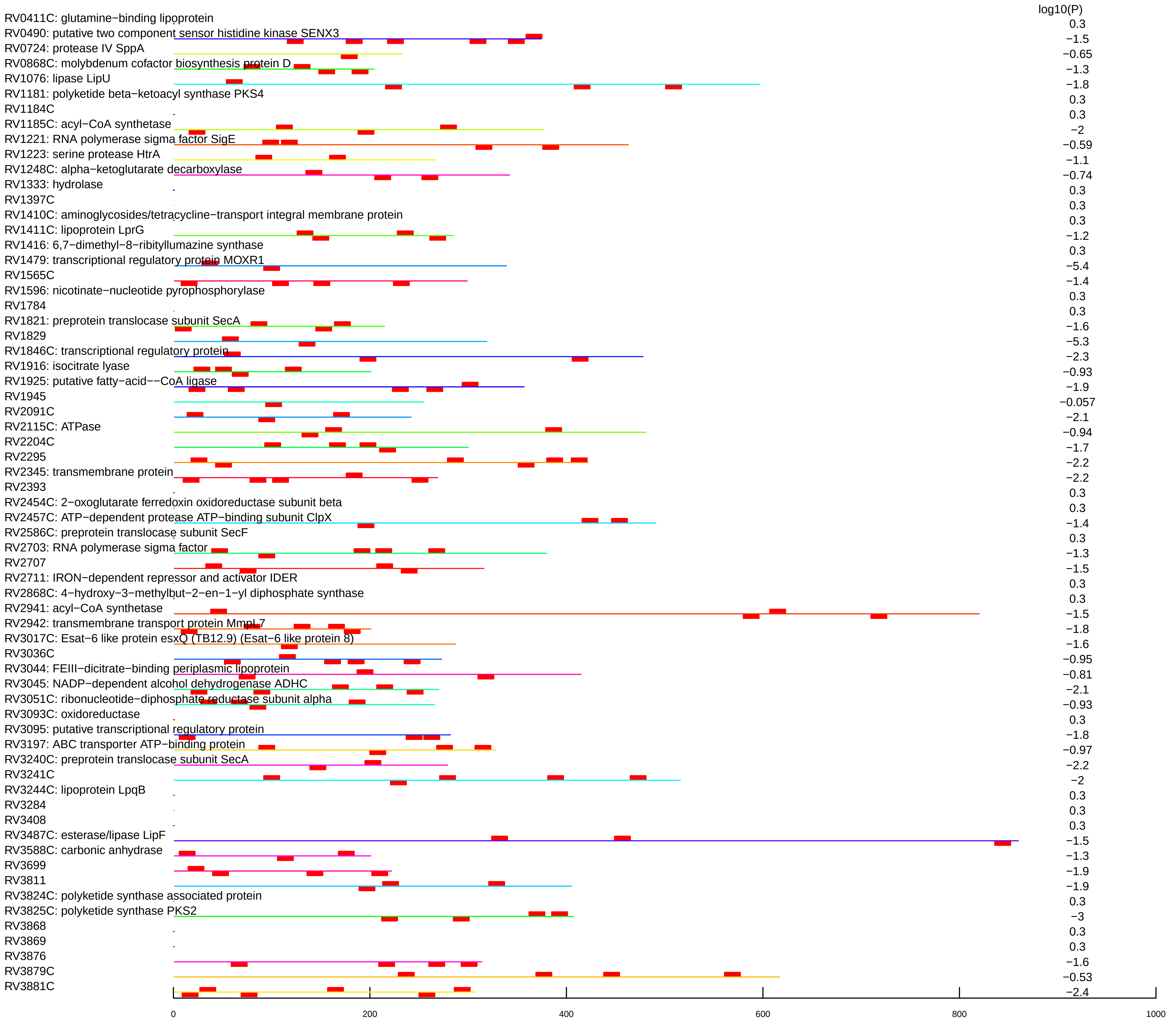

bicluster 50 ; 46 genes and 48 conditions

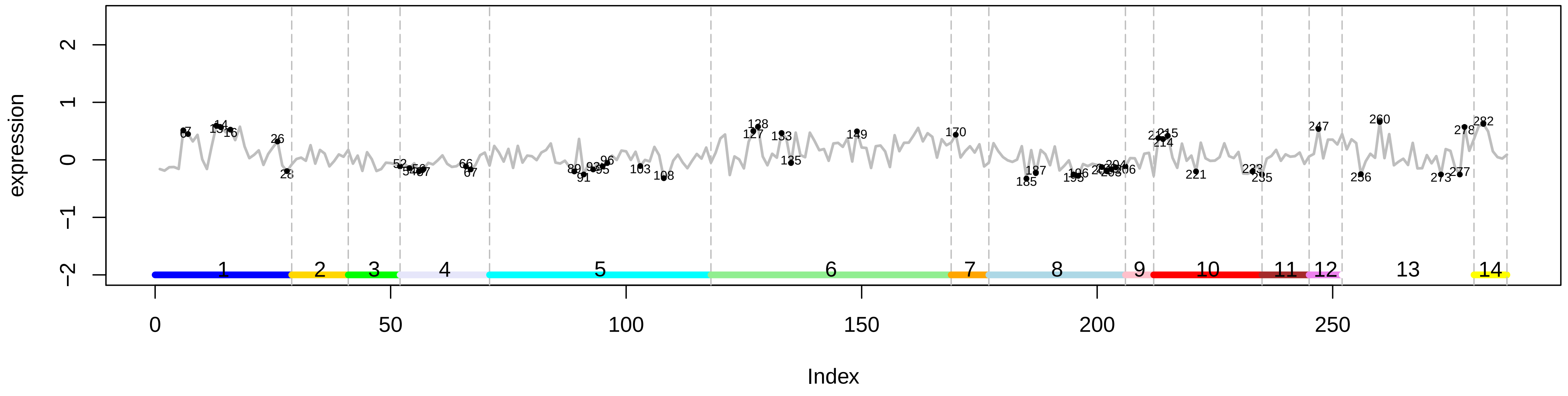

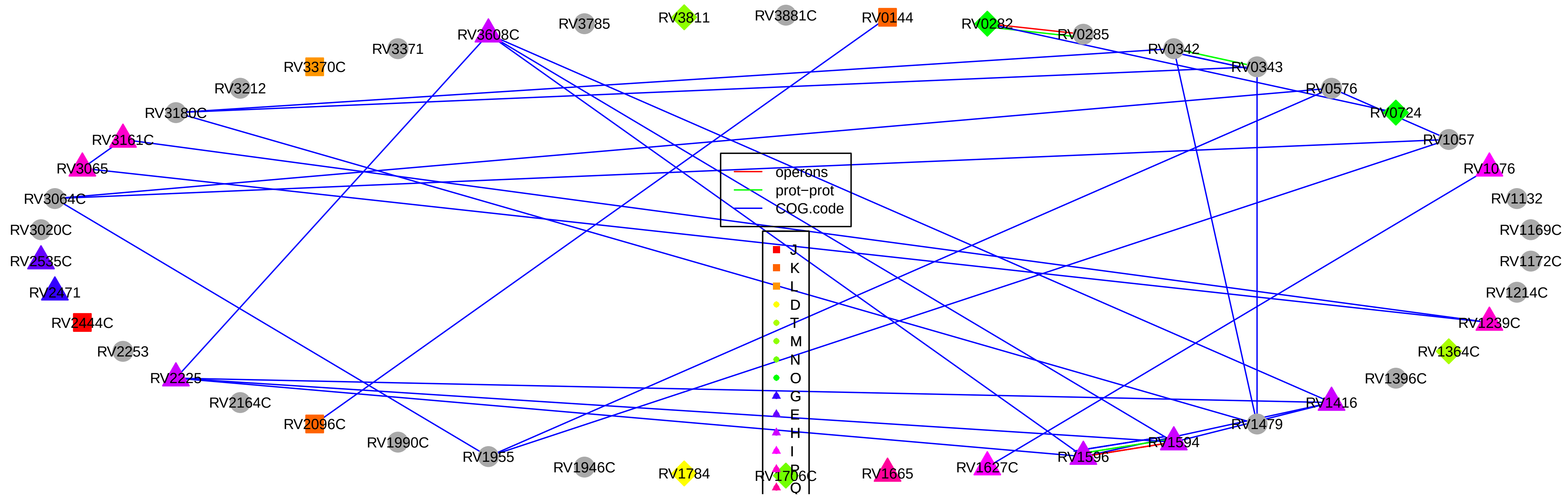

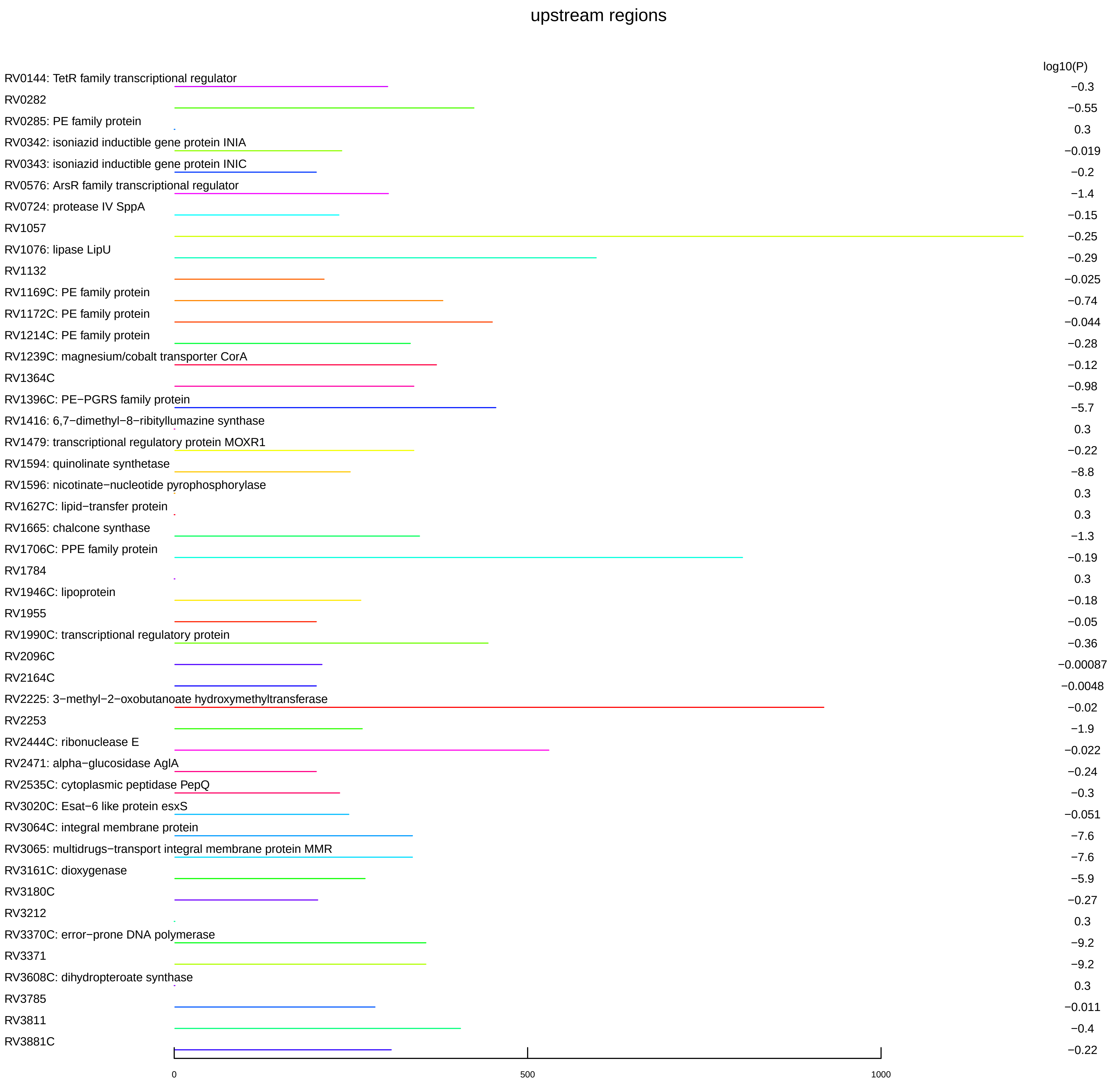

bicluster 51 ; 22 genes and 62 conditions

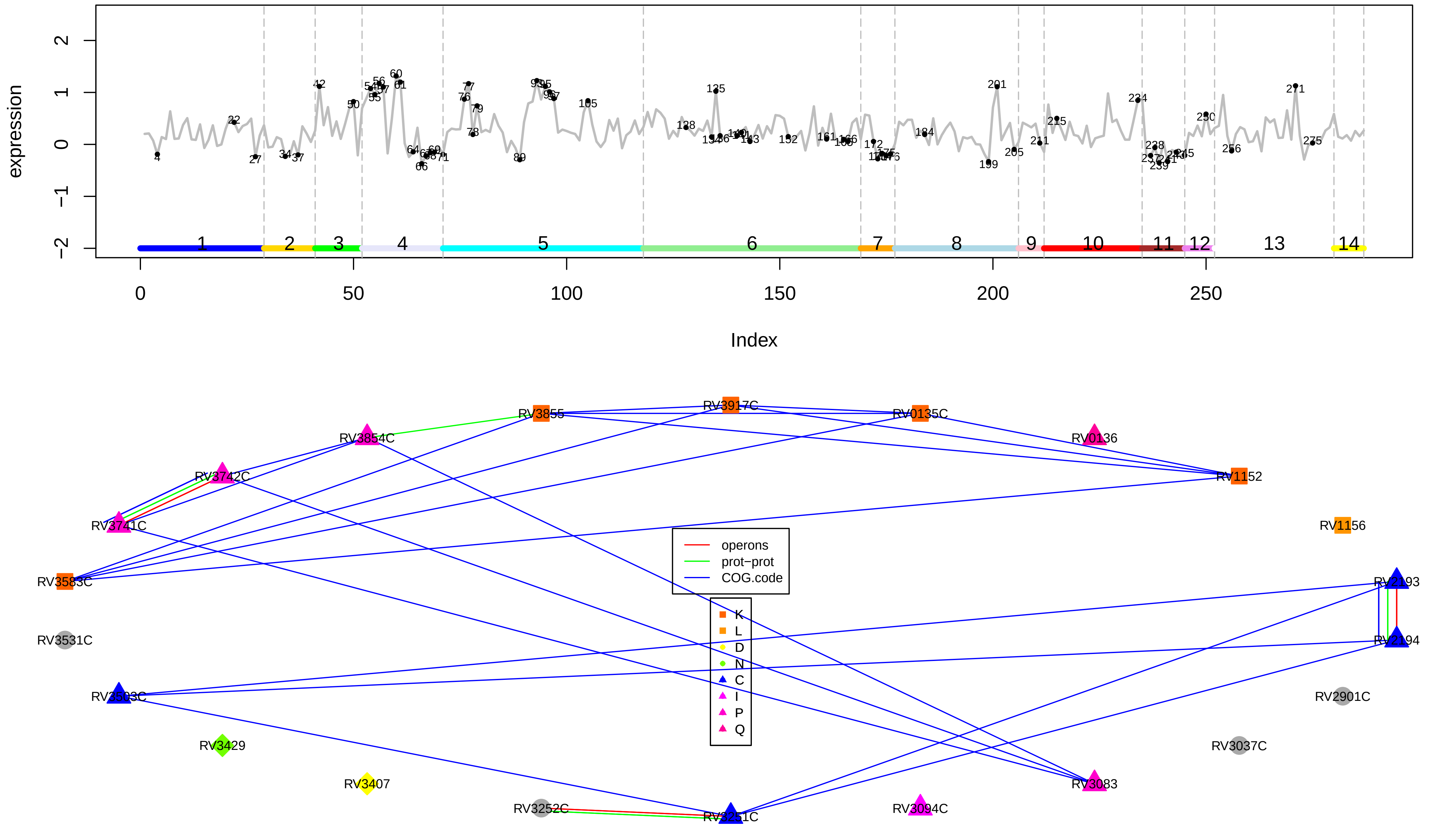

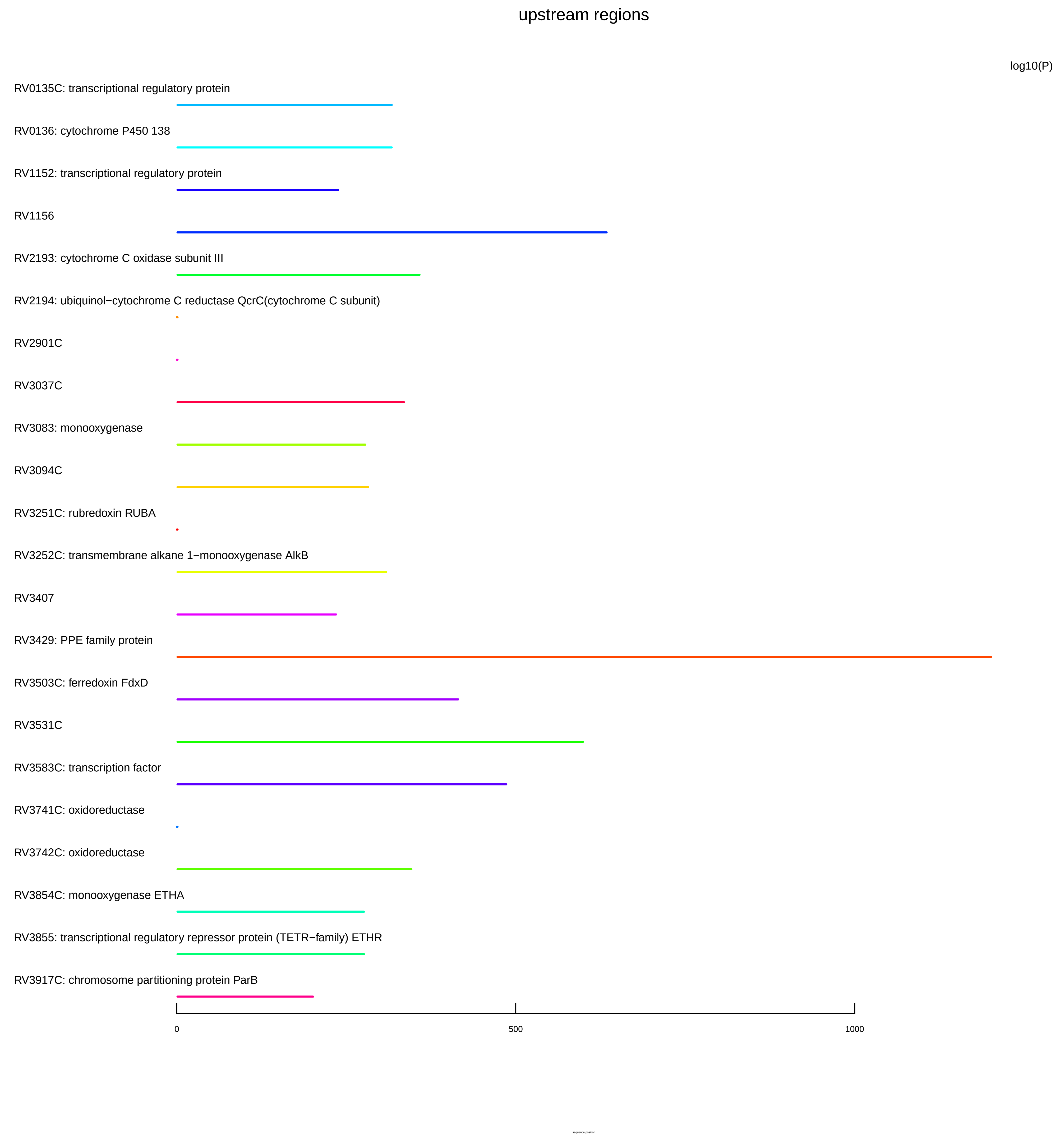

bicluster 52 ; 9 genes and 88 conditions

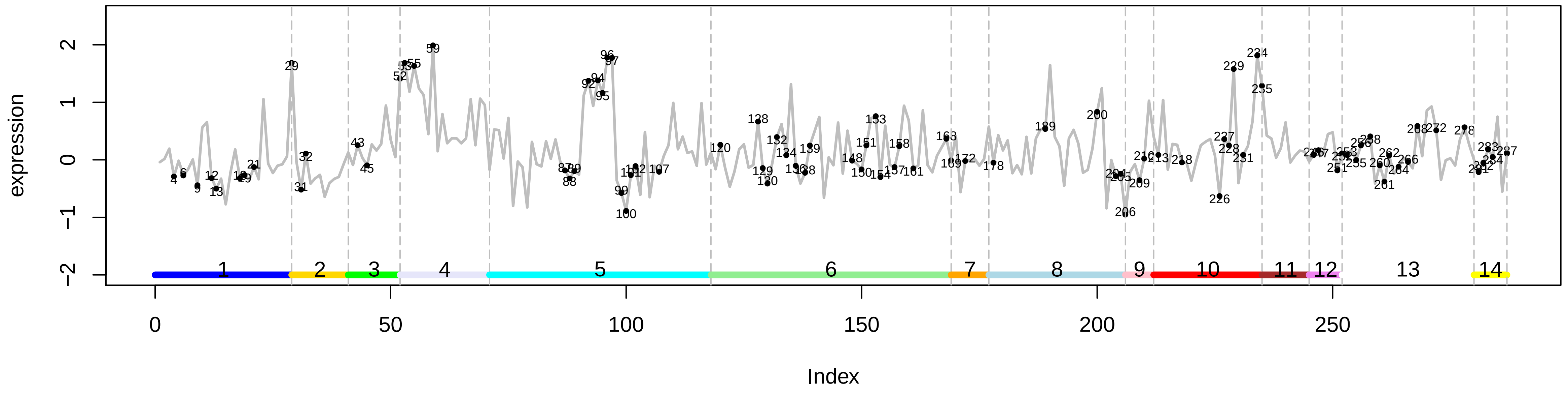

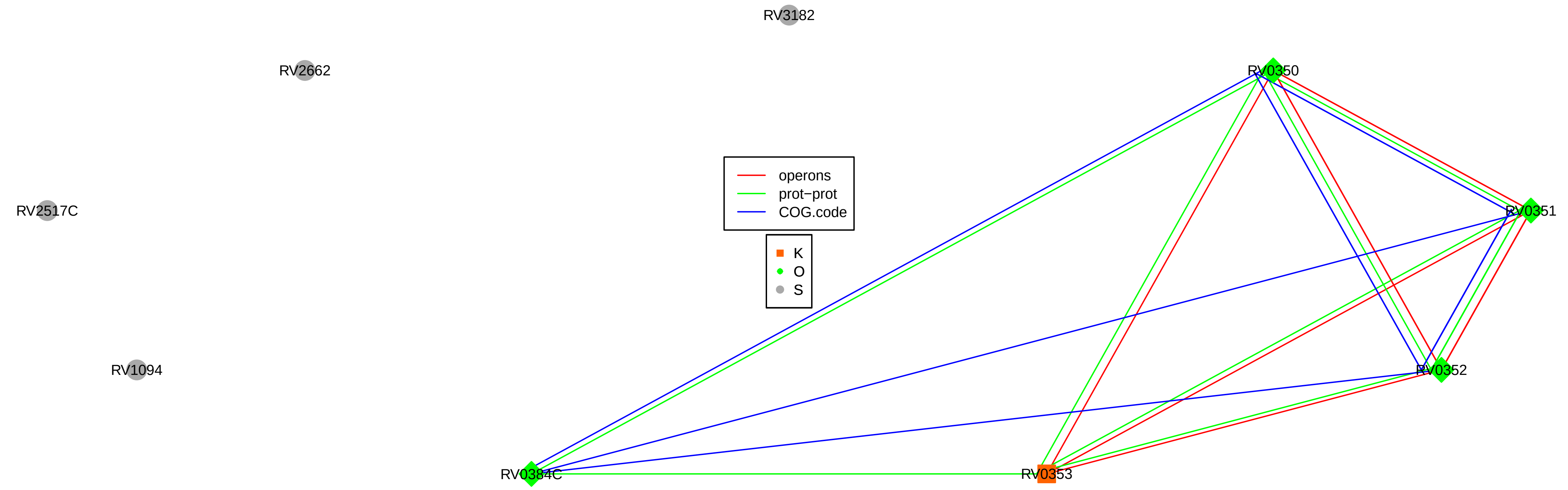

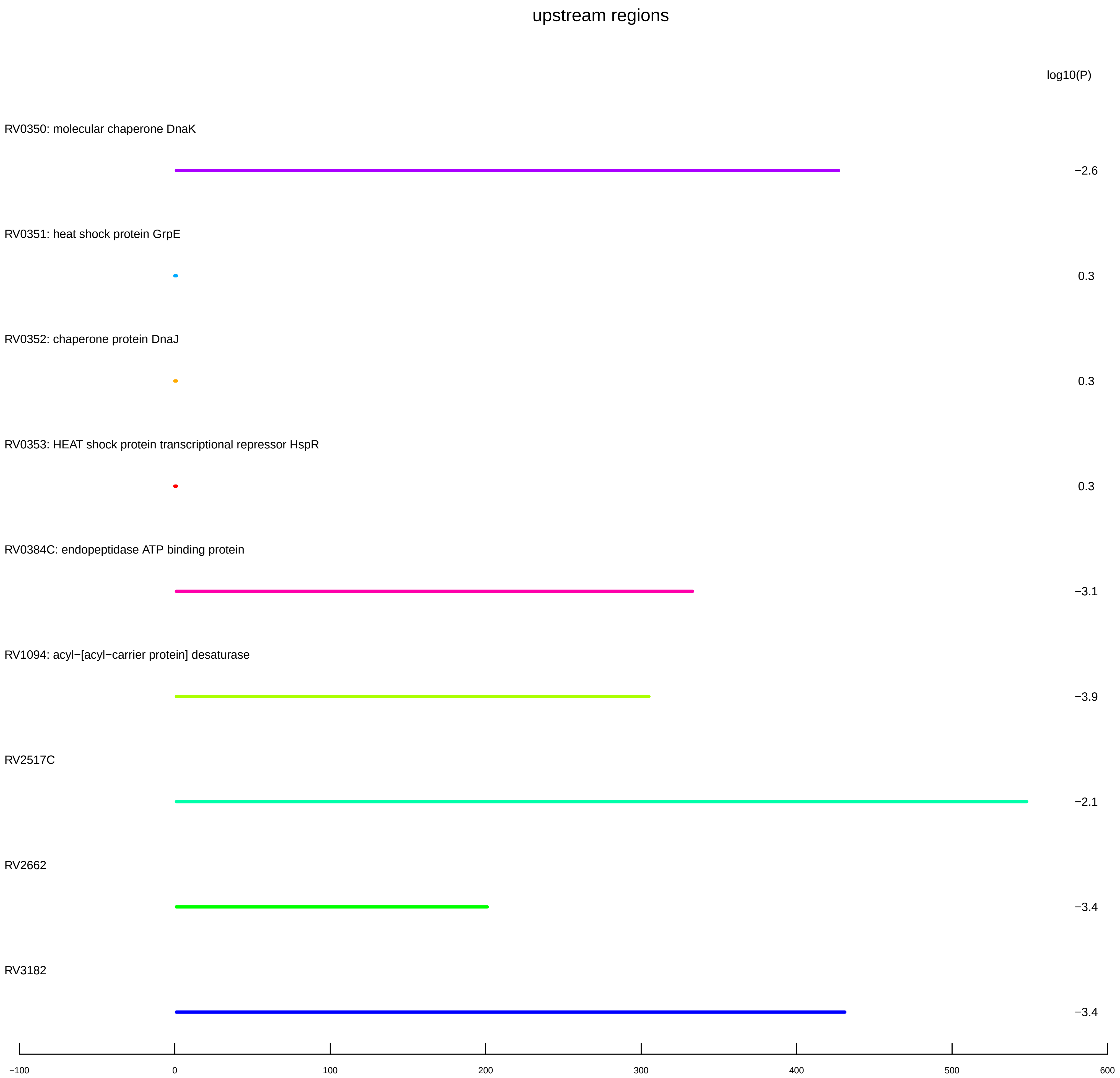

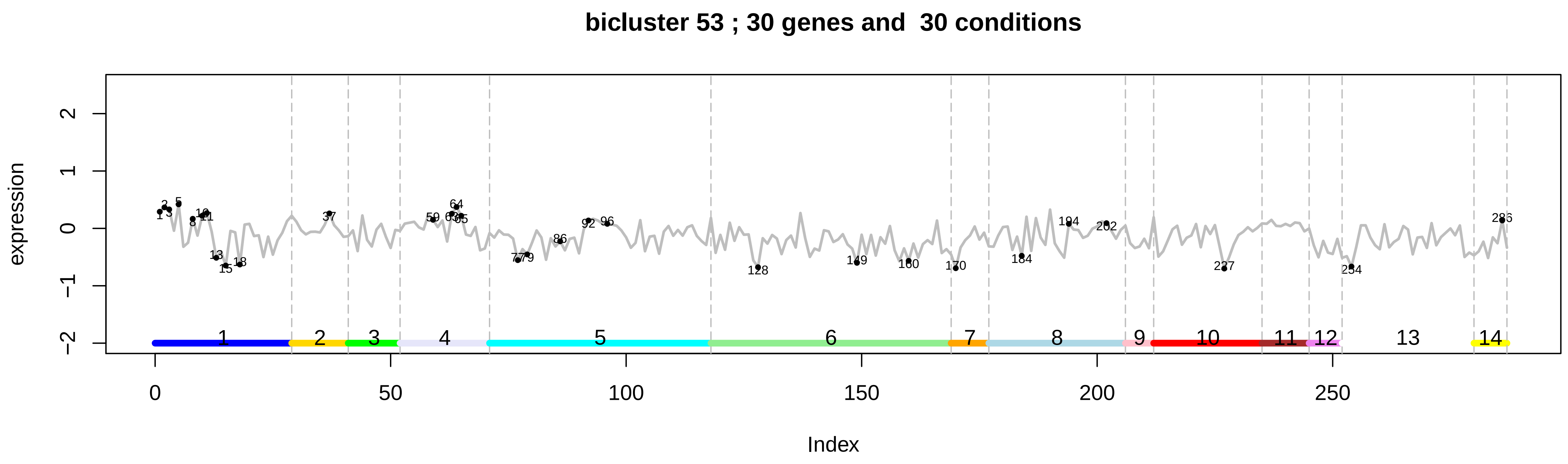

bicluster 53 ; 30 genes and 30 conditions
expression
Index

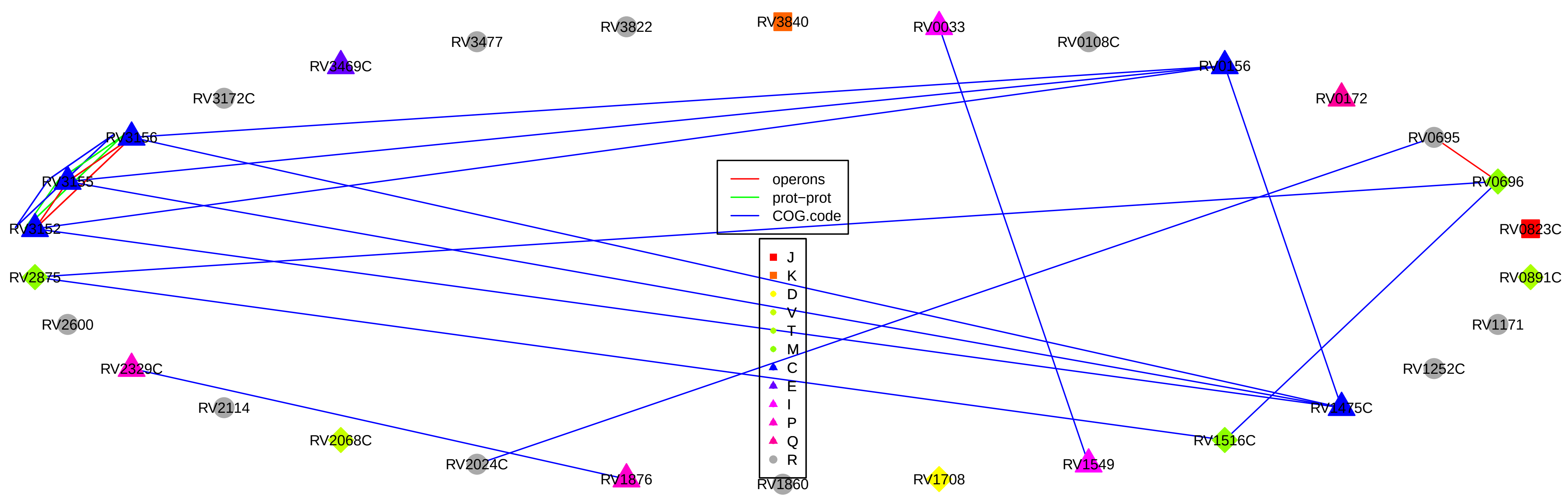

operons
prot−prot
COG.code
J
K
D
V
T
M
C
E
I
P
Q
R

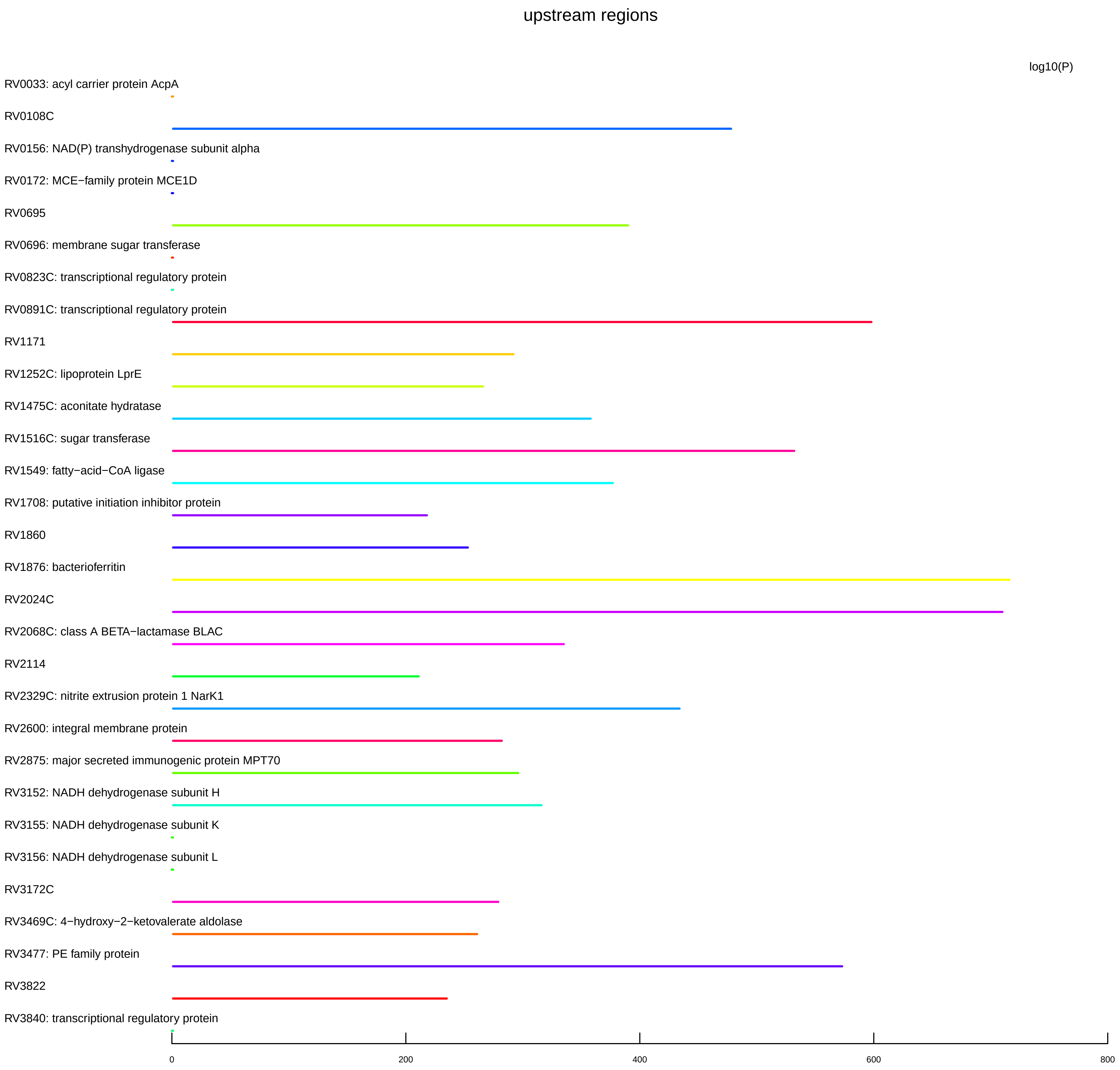

upstream regions
log10(P)
RV0033: acyl carrier protein AcpA
RV0108C
RV0156: NAD(P) transhydrogenase subunit alpha
RV0172: MCE−family protein MCE1D
RV0695
RV0696: membrane sugar transferase
RV0823C: transcriptional regulatory protein
RV0891C: transcriptional regulatory protein
RV1171
RV1252C: lipoprotein LprE
RV1475C: aconitate hydratase
RV1516C: sugar transferase
RV1549: fatty−acid−CoA ligase
RV1708: putative initiation inhibitor protein
RV1860
RV1876: bacterioferritin
RV2024C
RV2068C: class A BETA−lactamase BLAC
RV2114
RV2329C: nitrite extrusion protein 1 NarK1
RV2600: integral membrane protein
RV2875: major secreted immunogenic protein MPT70
RV3152: NADH dehydrogenase subunit H
RV3155: NADH dehydrogenase subunit K
RV3156: NADH dehydrogenase subunit L
RV3172C
RV3469C: 4−hydroxy−2−ketovalerate aldolase
RV3477: PE family protein
RV3822
RV3840: transcriptional regulatory protein

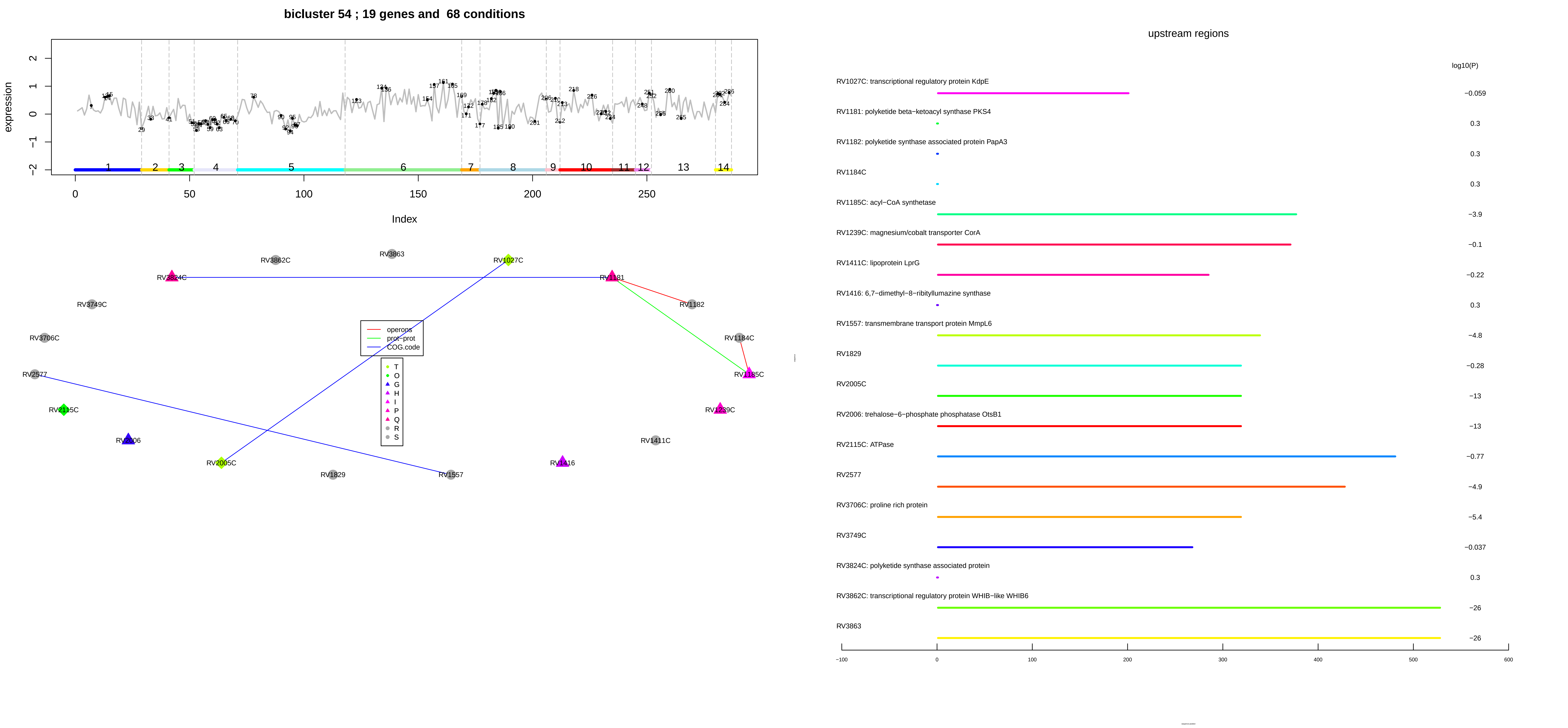

bicluster 54 ; 19 genes and 68 conditions
expression
Index
operons
prot−prot
COG.code
T
O
G
H
I
P
Q
R
S
RV3862C
RV3863
RV1027C
RV3824C
RV1181
RV3749C
RV1182
RV3706C
RV1184C
RV2577
RV1185C
RV2115C
RV1239C
RV2006
RV1411C
RV2005C
RV1829
RV1557
RV1416
upstream regions
log10(P)
RV1027C: transcriptional regulatory protein KdpE
-0.059
RV1181: polyketide beta−ketoacyl synthase PKS4
0.3
RV1182: polyketide synthase associated protein PapA3
0.3
RV1184C
0.3
RV1185C: acyl−CoA synthetase
-3.9
RV1239C: magnesium/cobalt transporter CorA
-0.1
RV1411C: lipoprotein LprG
-0.22
RV1416: 6,7−dimethyl−8−ribityllumazine synthase
0.3
RV1557: transmembrane transport protein MmpL6
-4.8
RV1829
-0.28
RV2005C
-13
RV2006: trehalose−6−phosphate phosphatase OtsB1
-13
RV2115C: ATPase
-0.77
RV2577
-4.9
RV3706C: proline rich protein
-5.4
RV3749C
-0.037
RV3824C: polyketide synthase associated protein
0.3
RV3862C: transcriptional regulatory protein WHIB−like WHIB6
-26
RV3863
-26

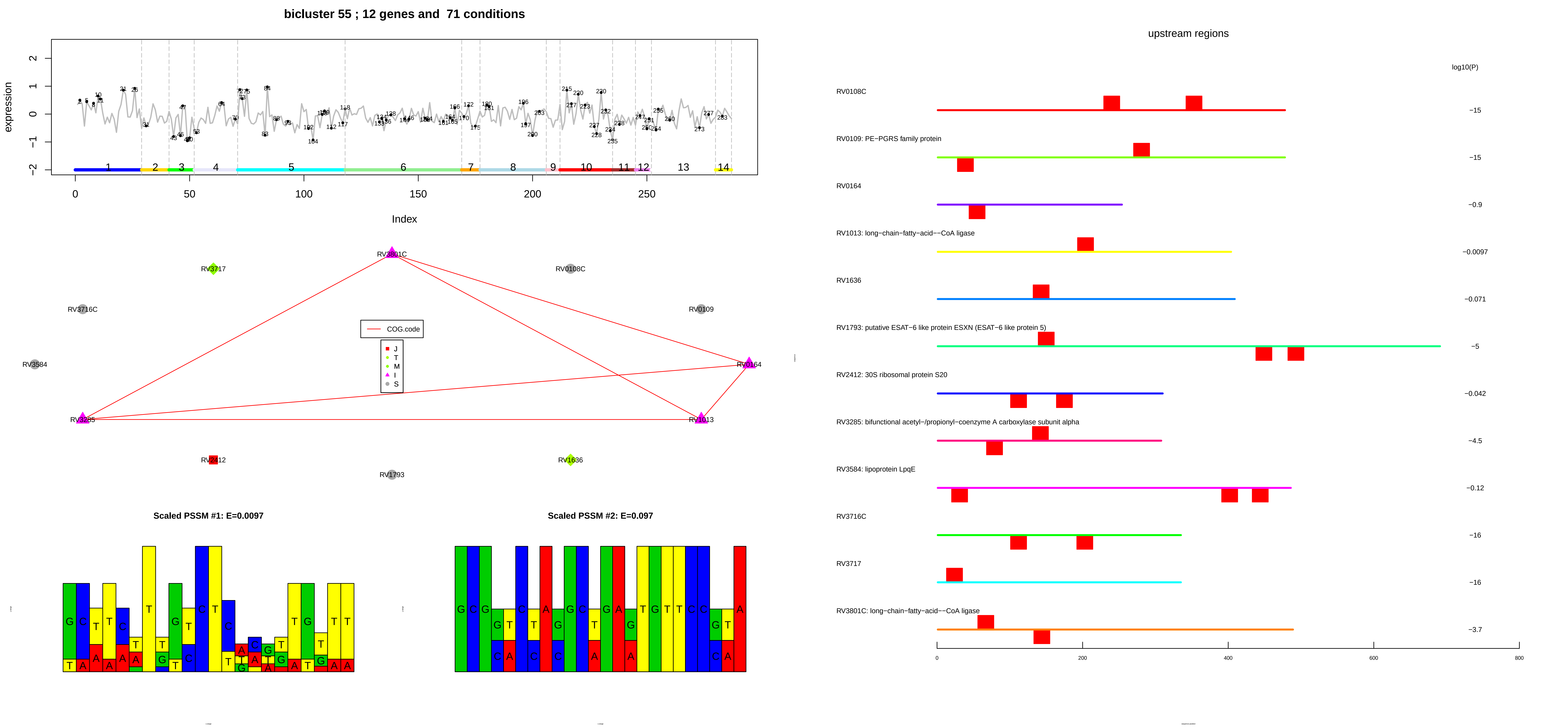

bicluster 55 ; 12 genes and 71 conditions
expression
Index
upstream regions
log10(P)
RV0108C
RV0109: PE−PGRS family protein
RV0164
RV1013: long−chain−fatty−acid−−CoA ligase
RV1636
RV1793: putative ESAT−6 like protein ESXN (ESAT−6 like protein 5)
RV2412: 30S ribosomal protein S20
RV3285: bifunctional acetyl−/propionyl−coenzyme A carboxylase subunit alpha
RV3584: lipoprotein LpqE
RV3716C
RV3717
RV3801C: long−chain−fatty−acid−−CoA ligase
−15
−15
−0.9
−0.0097
−0.071
−5
−0.042
−4.5
−0.12
−16
−16
−3.7
COG.code
J
T
M
I
S
RV3801C
RV3717
RV0108C
RV3716C
RV0109
RV3584
RV0164
RV3285
RV1013
RV2412
RV1636
RV1793
Scaled PSSM #1: E=0.0097
Scaled PSSM #2: E=0.097

bicluster 56 ; 33 genes and 42 conditions

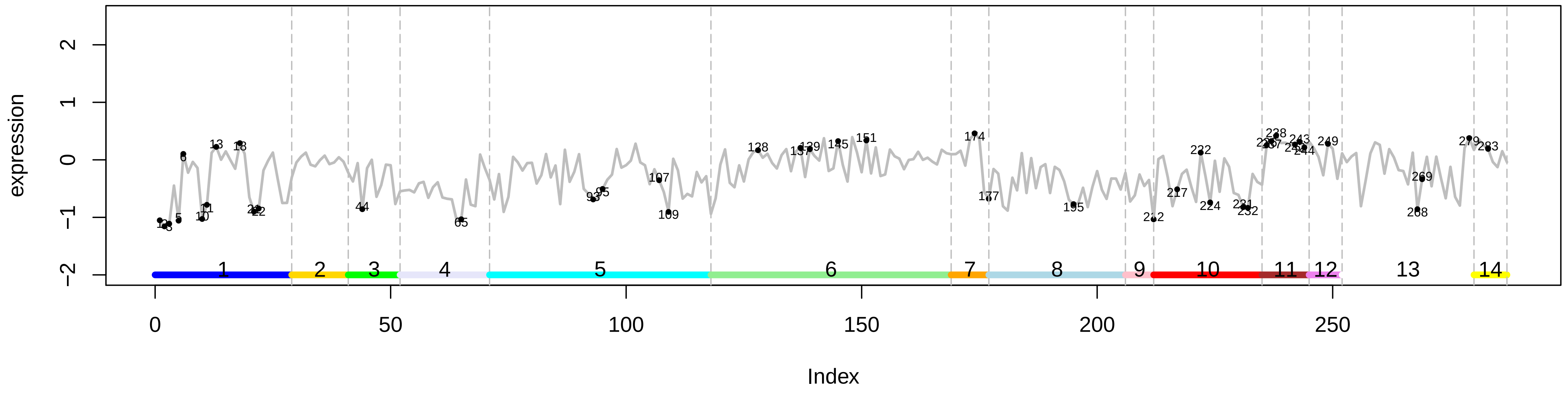

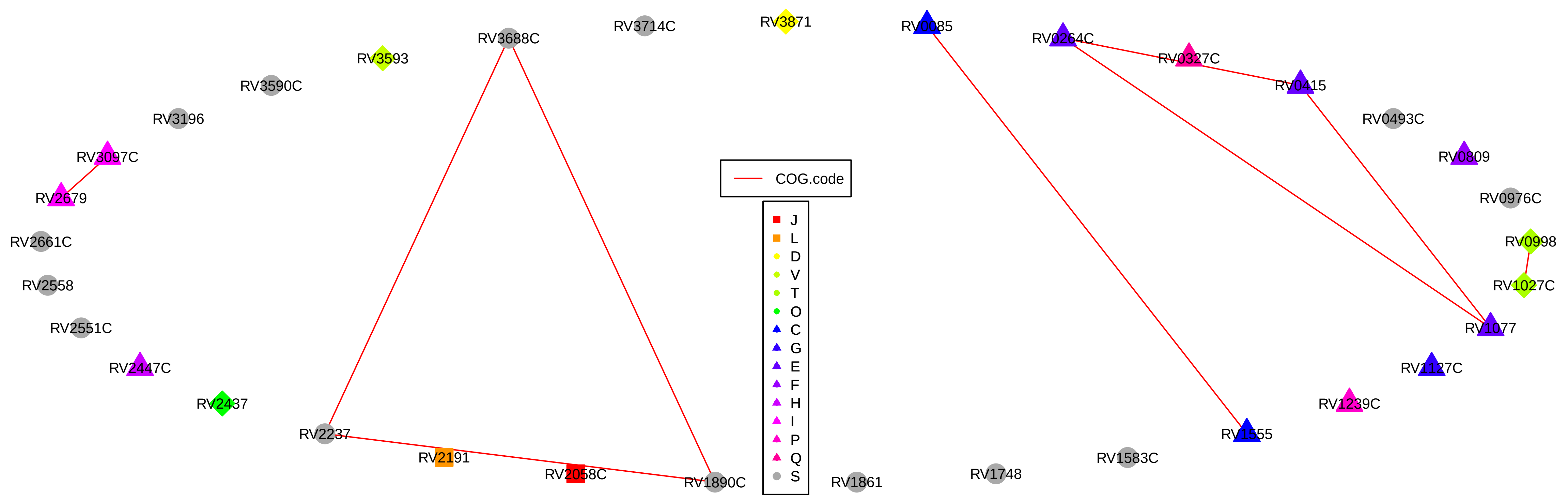

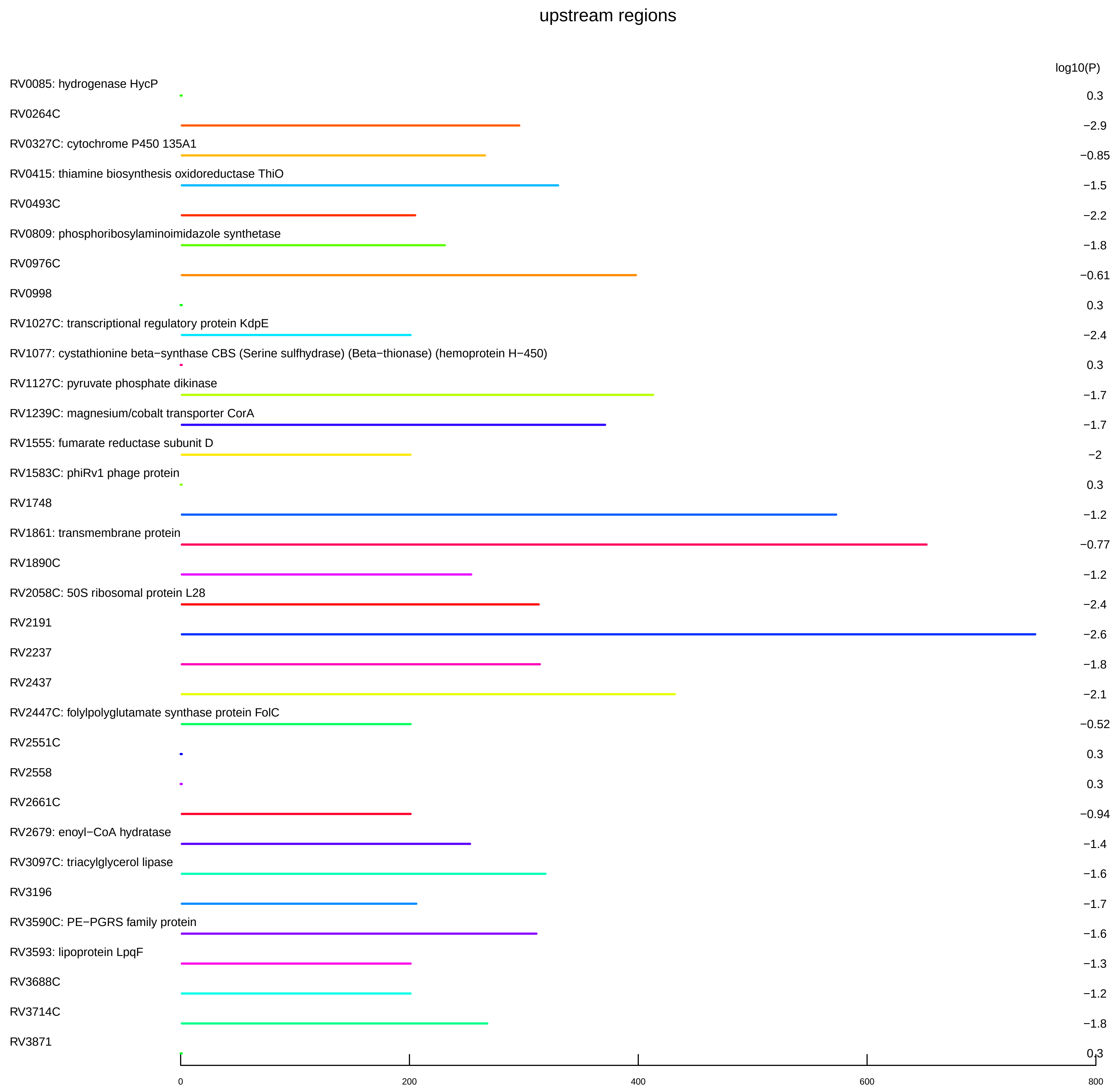

bicluster 57 ; 24 genes and 48 conditions

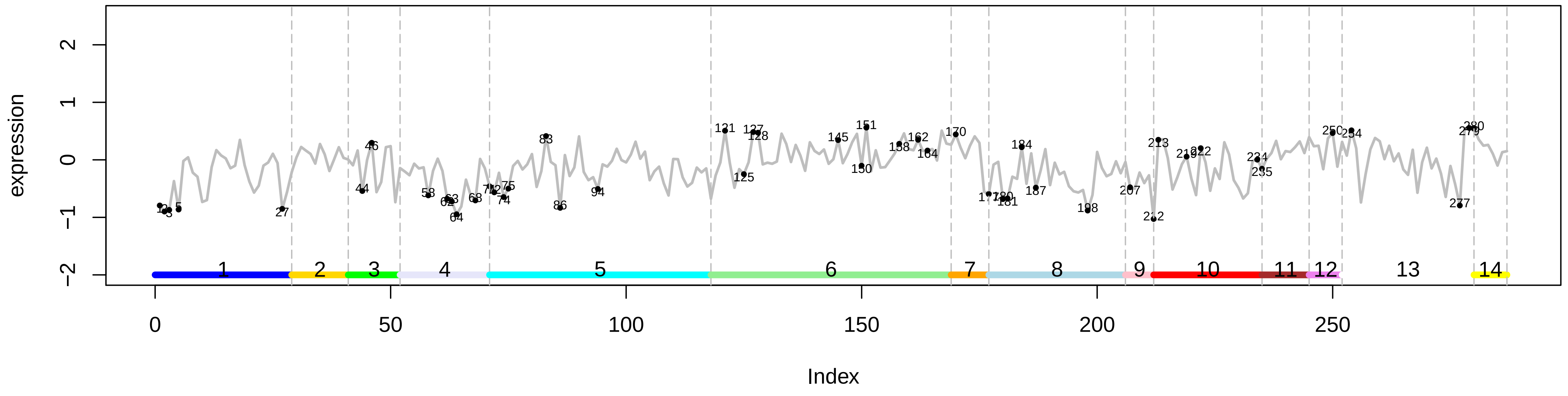

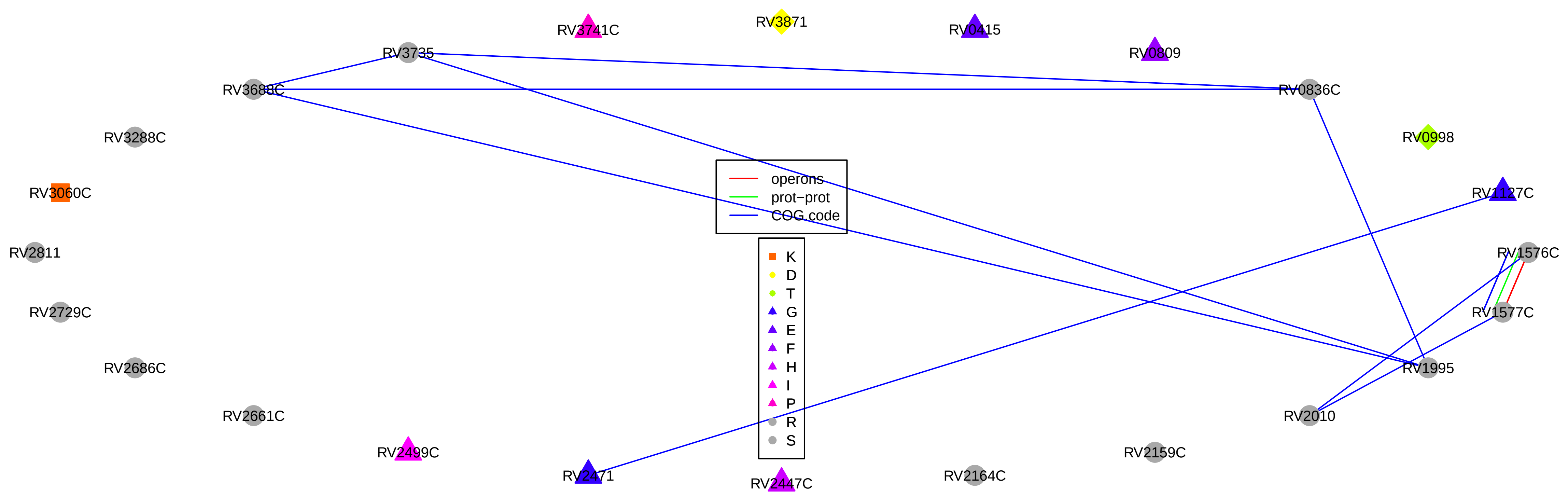

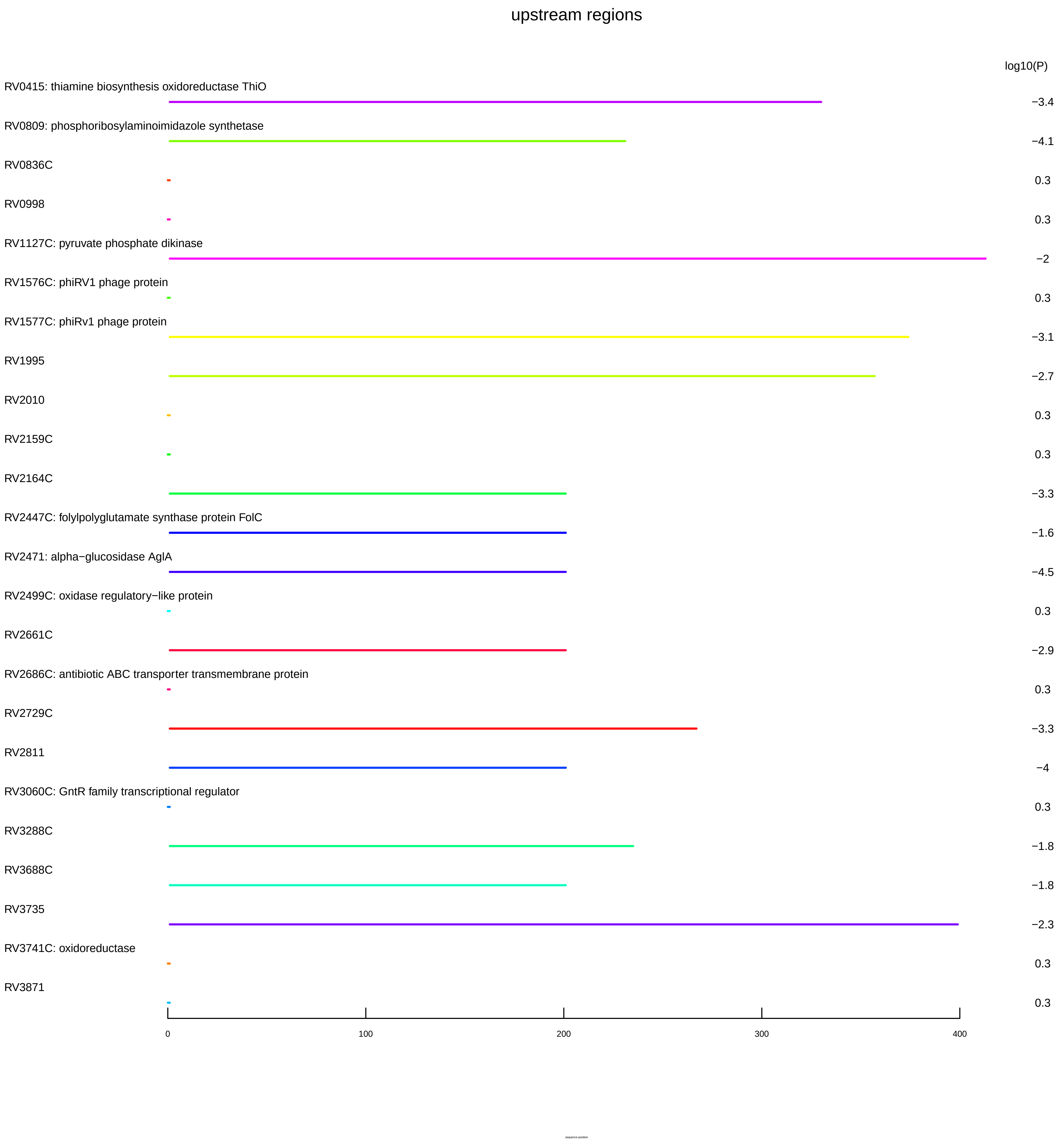

bicluster 58 ; 48 genes and 98 conditions

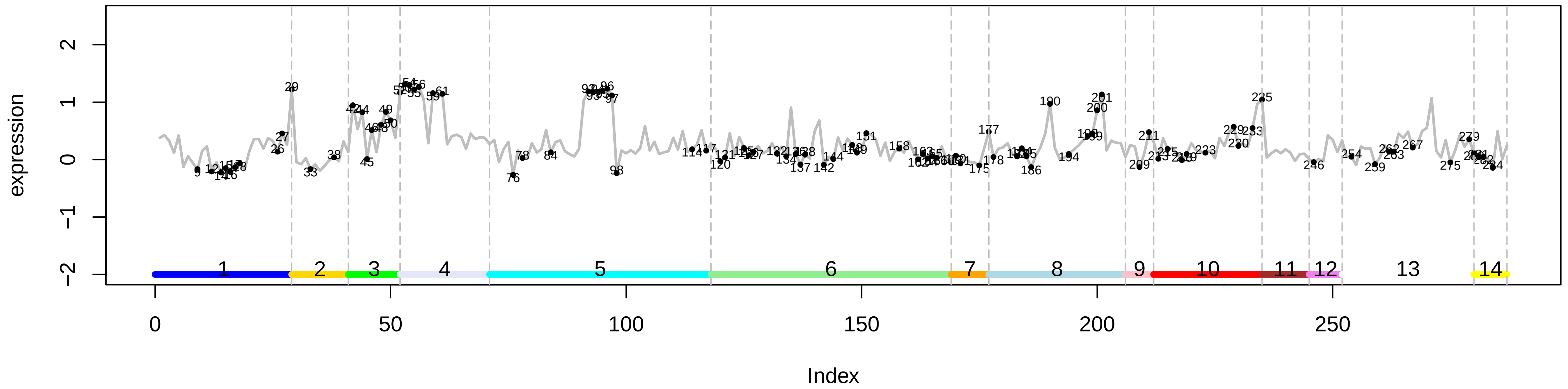

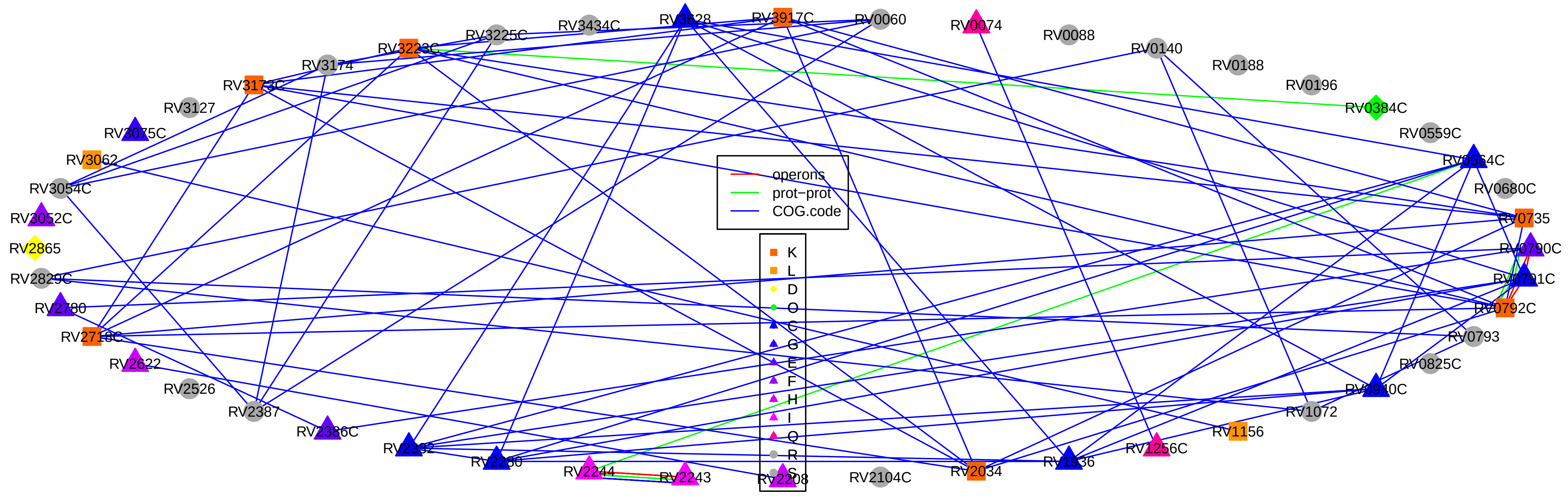

Scaled PSSM #1: E=1.1e−10

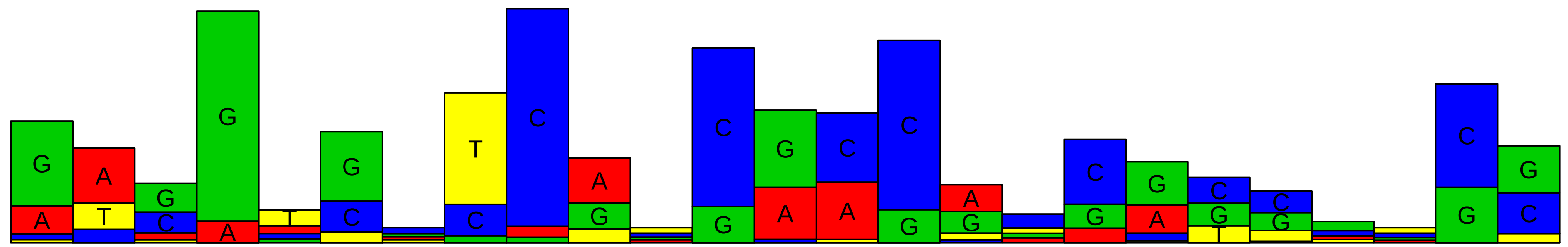

upstream regions

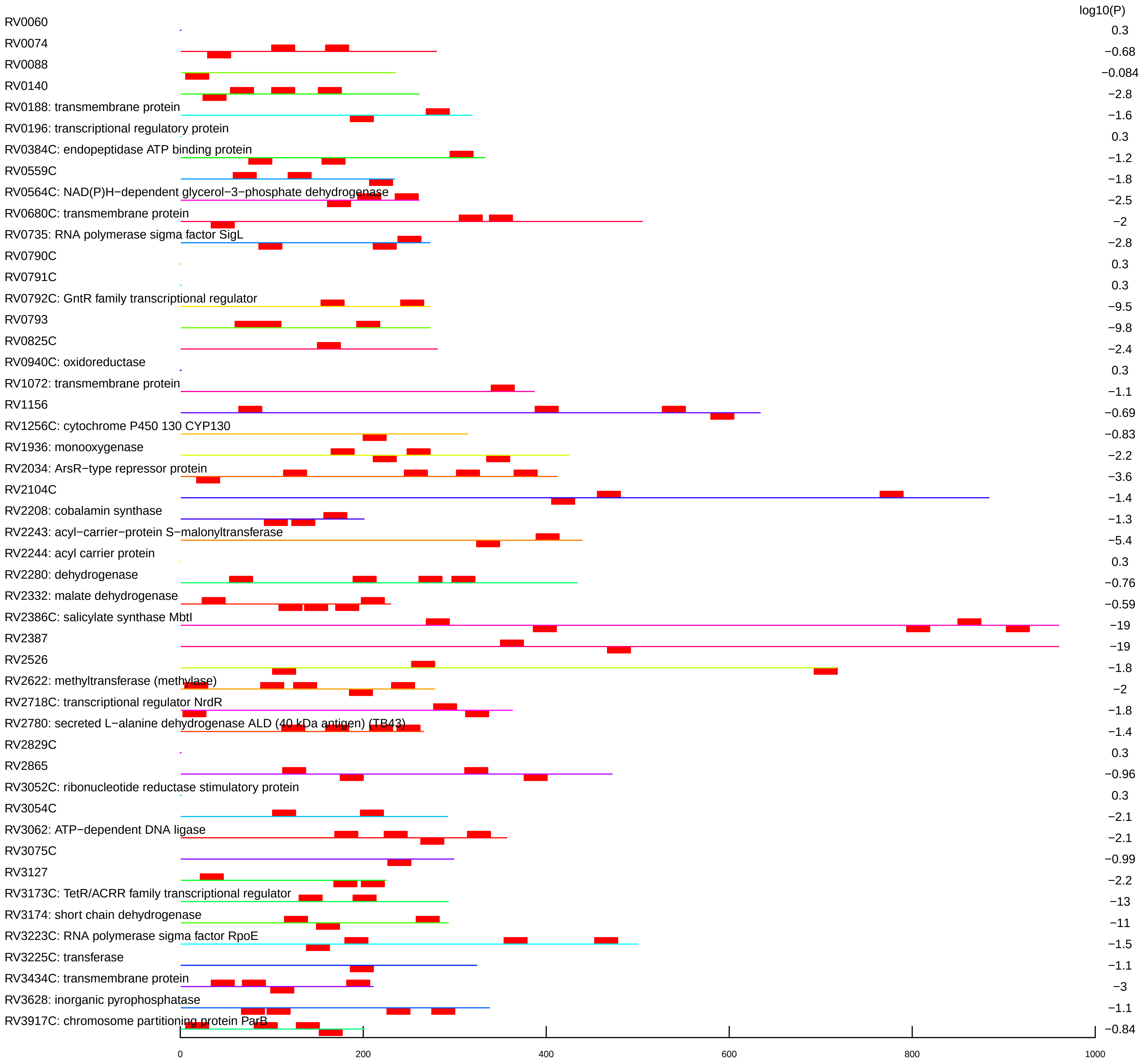

bicluster 59 ; 32 genes and 83 conditions

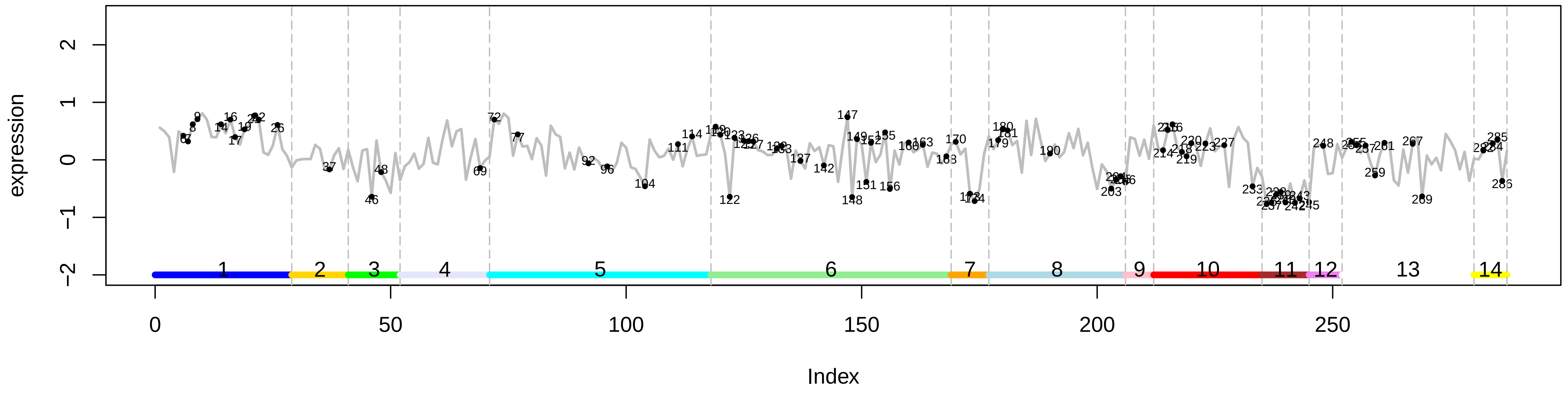

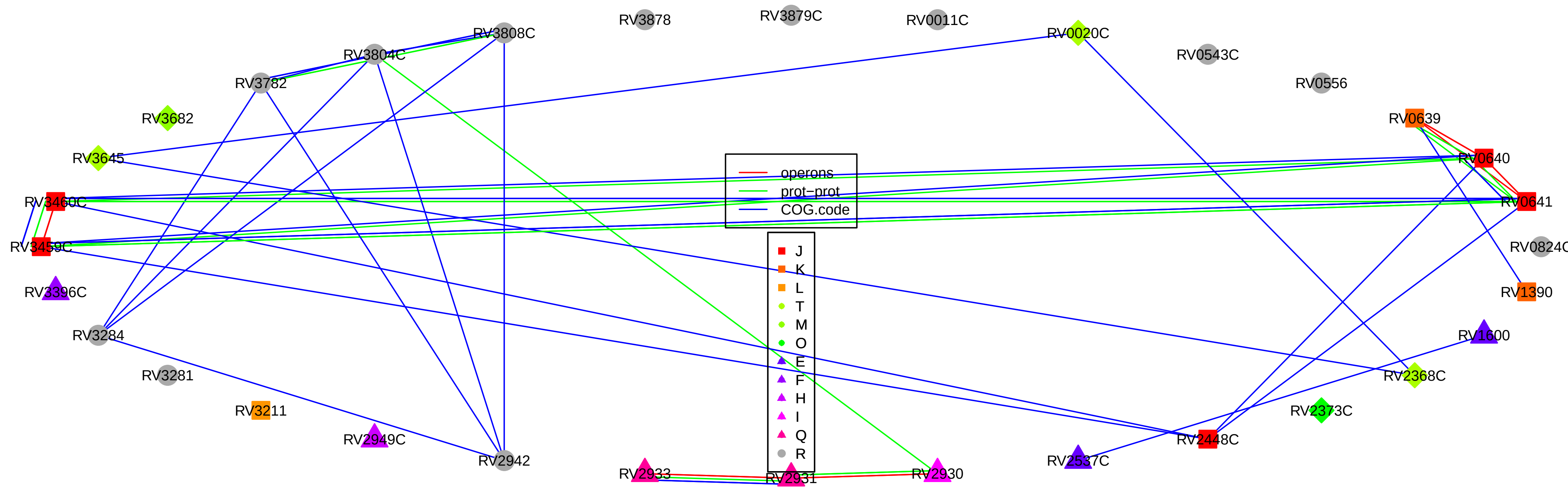

upstream regions

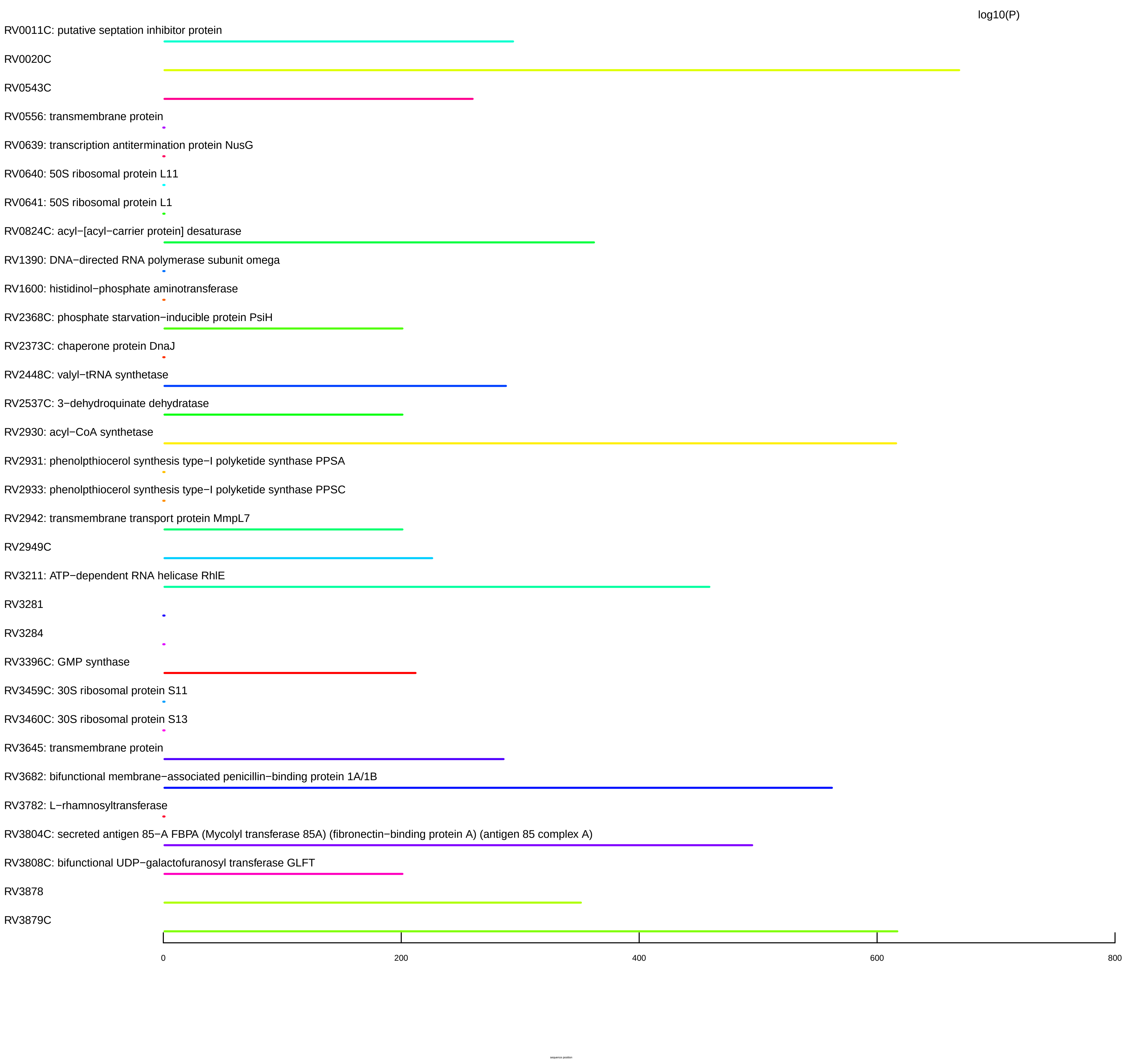

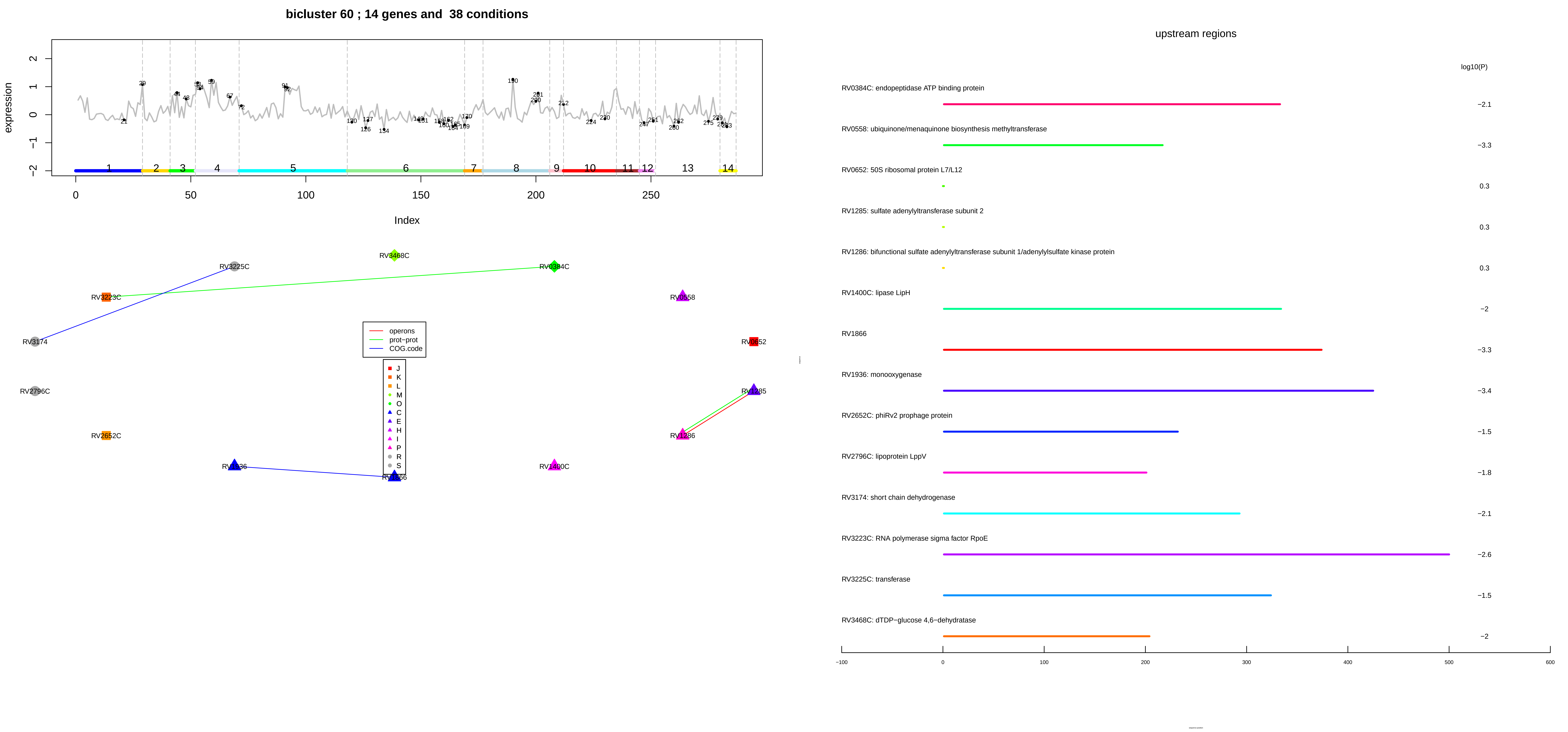

bicluster 60 ; 14 genes and 38 conditions
expression
Index
operons
prot−prot
COG.code
upstream regions
log10(P)
RV0384C: endopeptidase ATP binding protein
RV0558: ubiquinone/menaquinone biosynthesis methyltransferase
RV0652: 50S ribosomal protein L7/L12
RV1285: sulfate adenylyltransferase subunit 2
RV1286: bifunctional sulfate adenylyltransferase subunit 1/adenylylsulfate kinase protein
RV1400C: lipase LipH
RV1866
RV1936: monooxygenase
RV2652C: phiRv2 prophage protein
RV2796C: lipoprotein LppV
RV3174: short chain dehydrogenase
RV3223C: RNA polymerase sigma factor RpoE
RV3225C: transferase
RV3468C: dTDP−glucose 4,6−dehydratase

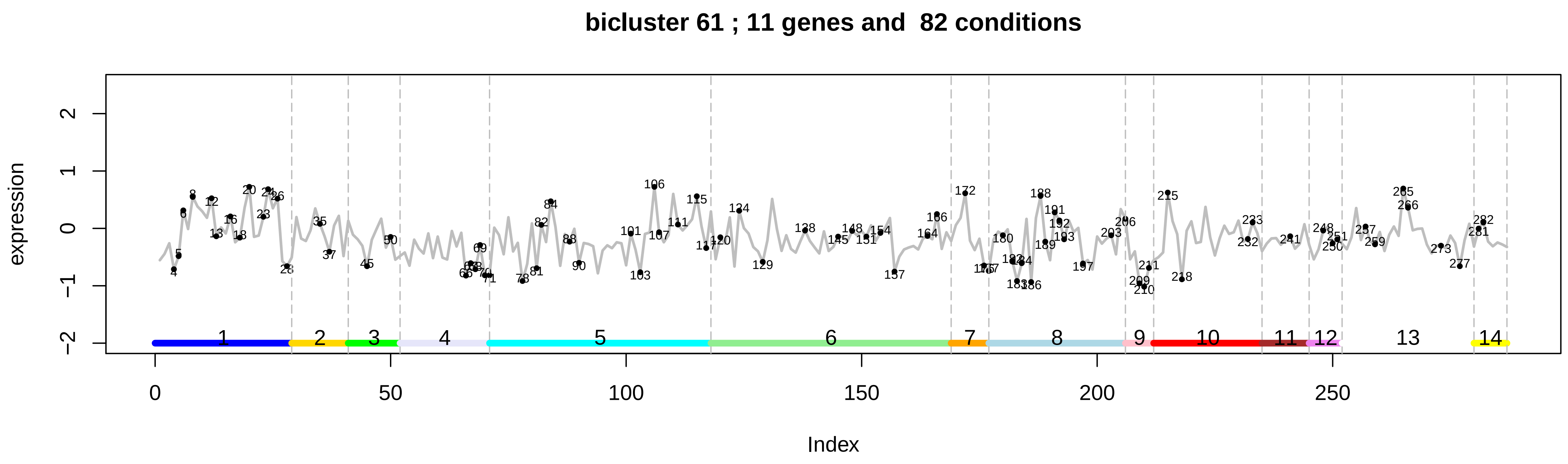
bicluster 61 ; 11 genes and 82 conditions
expression
Index
1 2 3 4 5 6 7 8 9 10 11 12 13 14
0 50 100 150 200 250

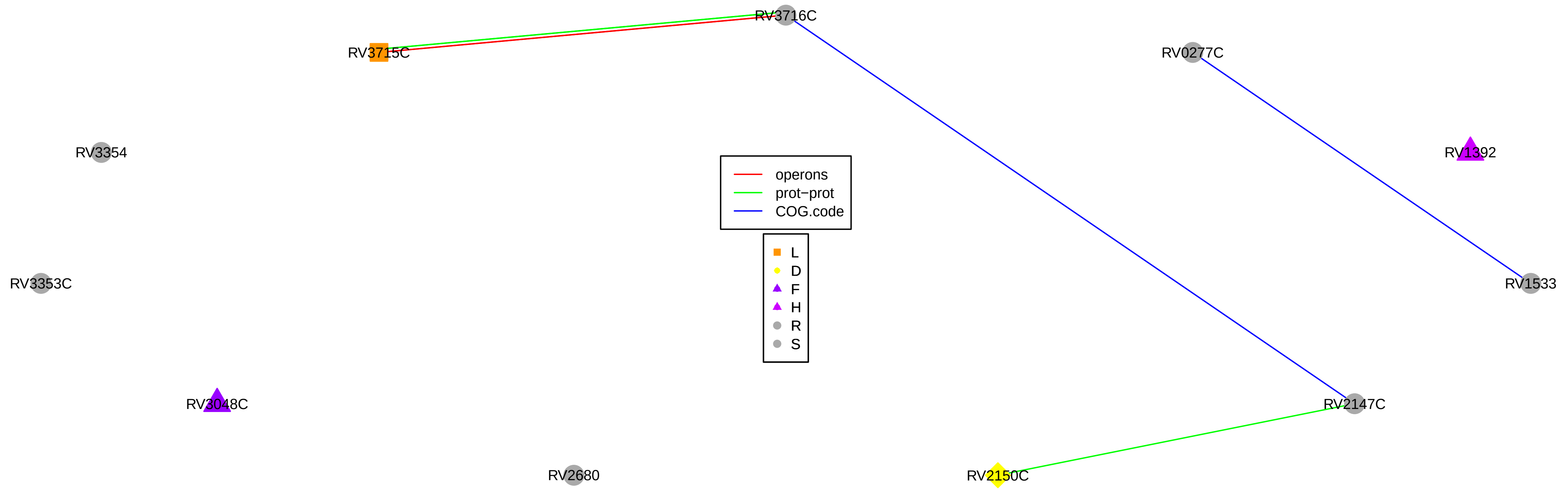
RV3716C
RV3715C
RV0277C
RV3354
RV1392
RV3353C
RV1533
RV3048C
RV2147C
RV2680
RV2150C
operons
prot−prot
COG.code
L
D
F
H
R
S

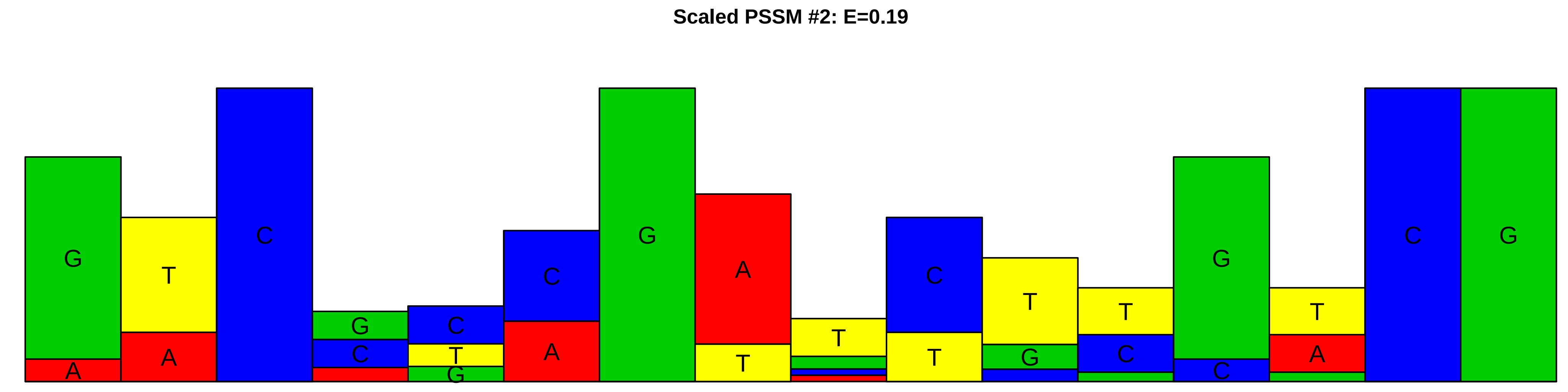
Scaled PSSM #2: E=0.19

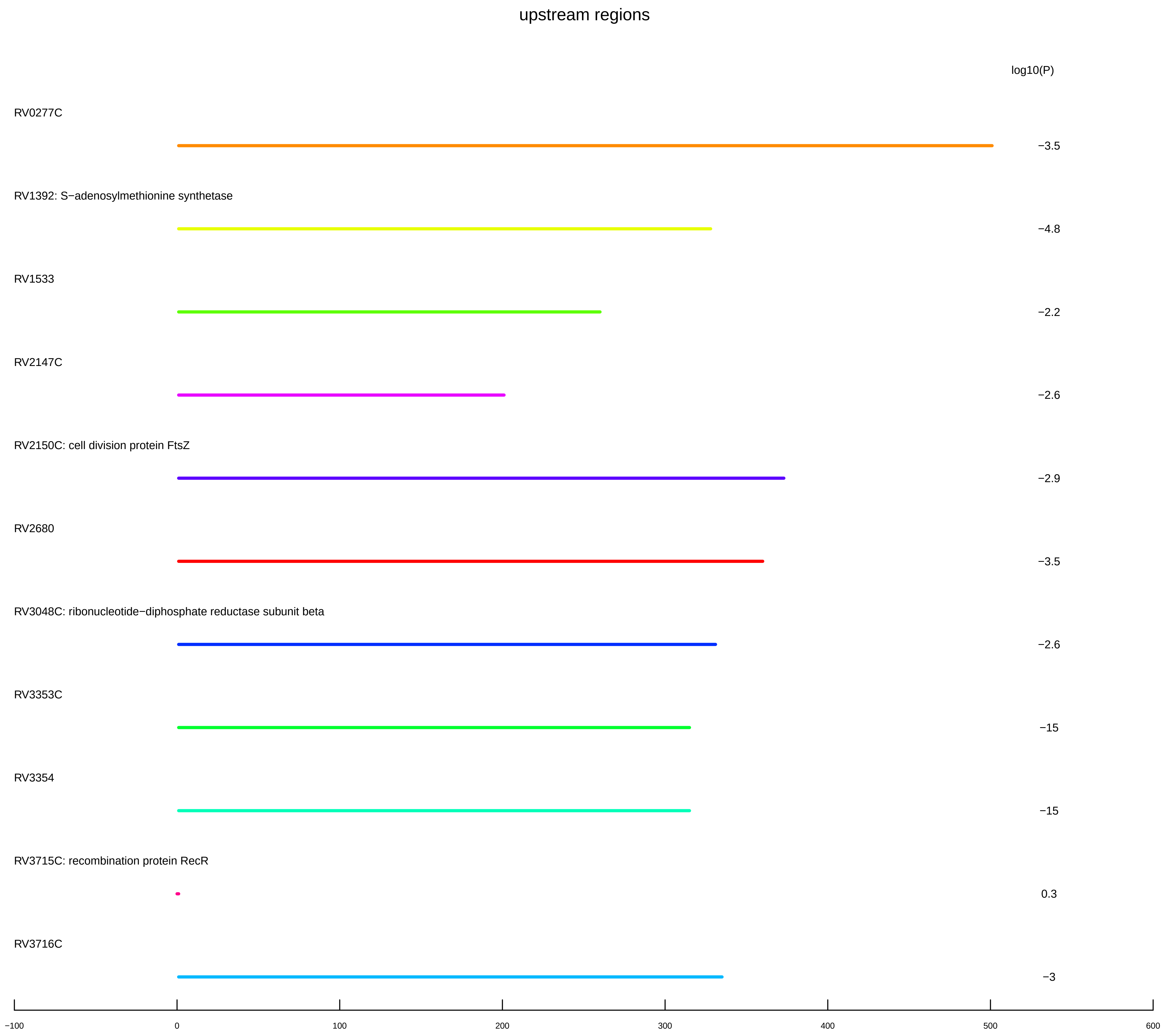
upstream regions
log10(P)
RV0277C −3.5
RV1392: S−adenosylmethionine synthetase −4.8
RV1533 −2.2
RV2147C −2.6
RV2150C: cell division protein FtsZ −2.9
RV2680 −3.5
RV3048C: ribonucleotide−diphosphate reductase subunit beta −2.6
RV3353C −15
RV3354 −15
RV3715C: recombination protein RecR 0.3
RV3716C −3
−100 0 100 200 300 400 500 600

bicluster 62 ; 13 genes and 40 conditions

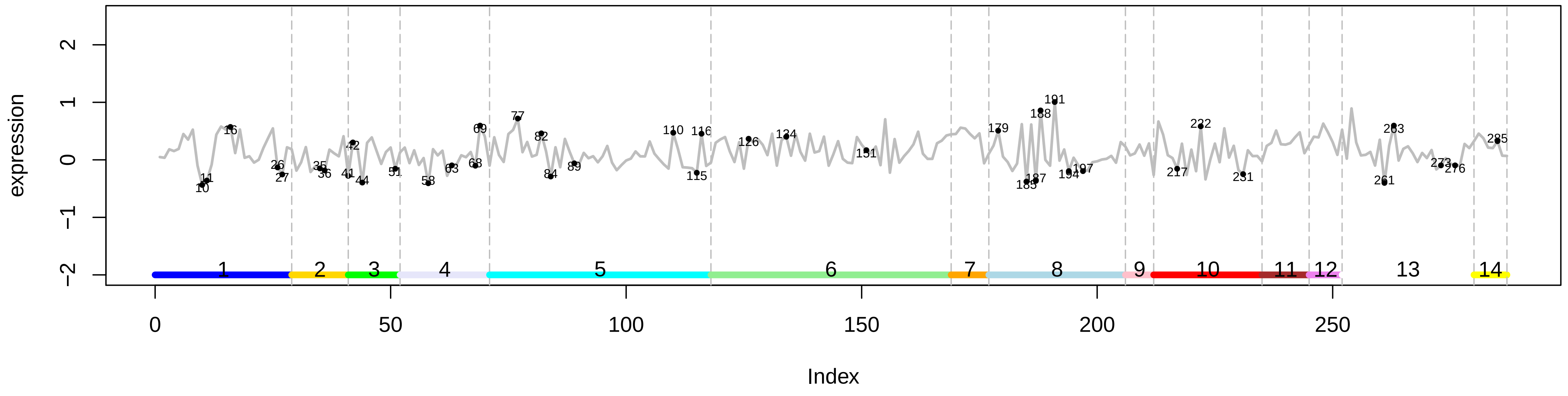

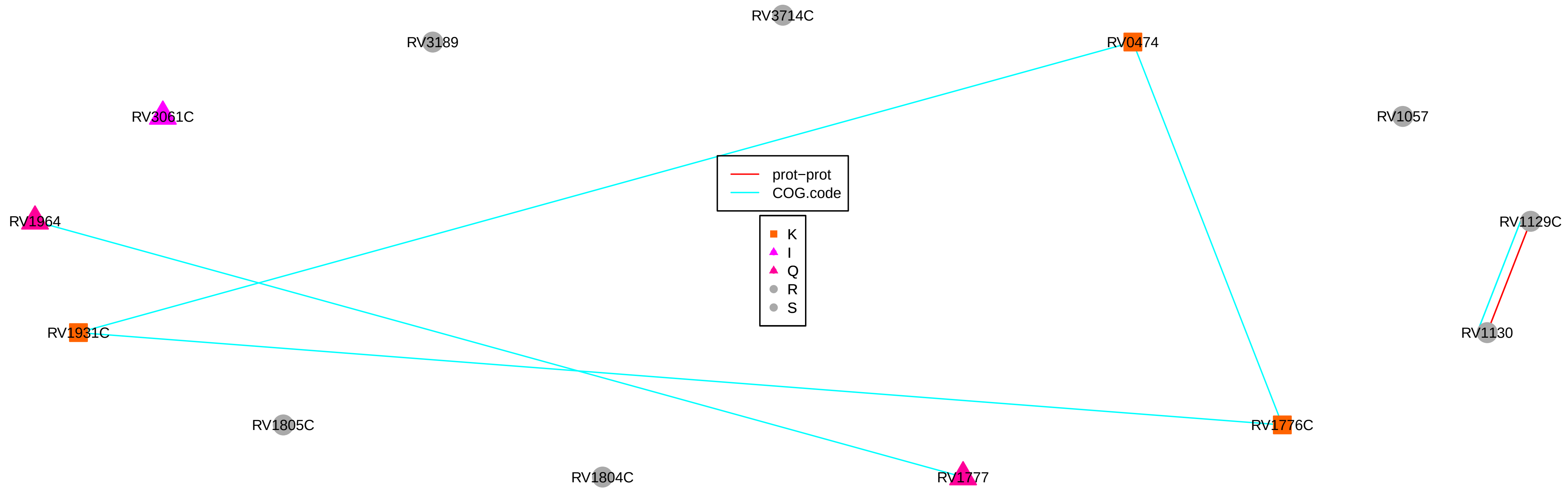

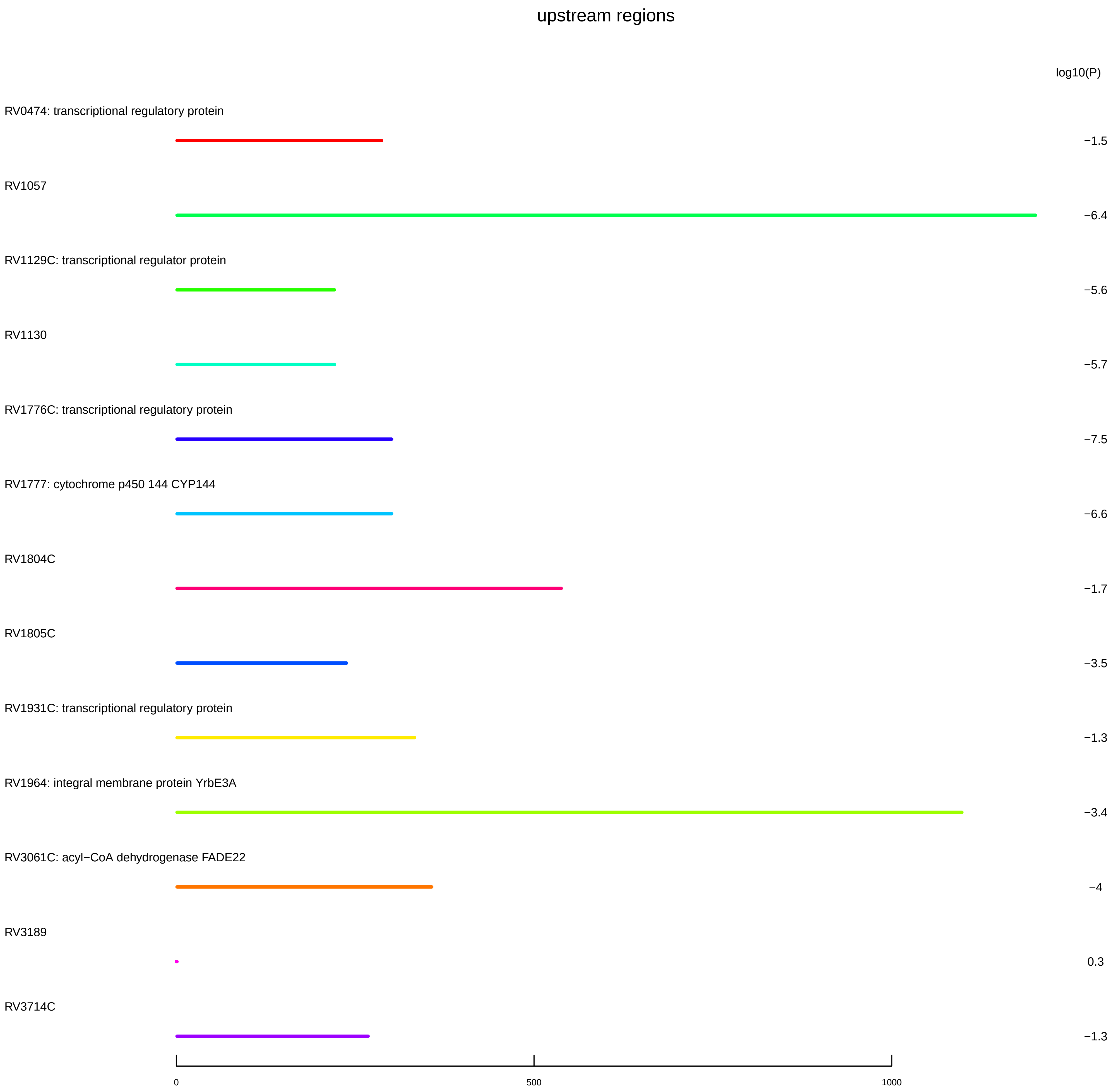

bicluster 63 ; 22 genes and 38 conditions

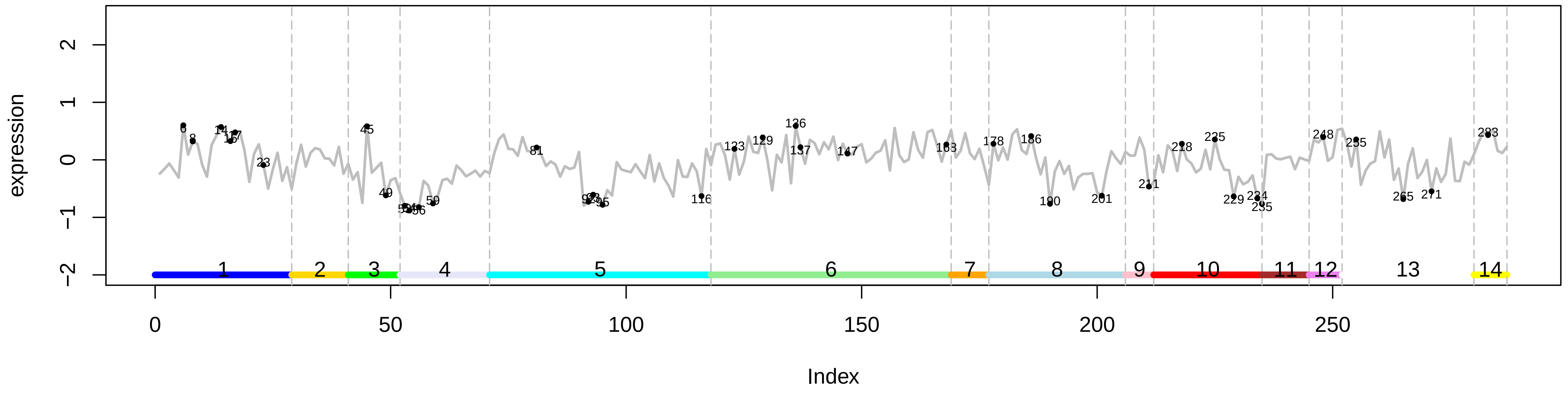

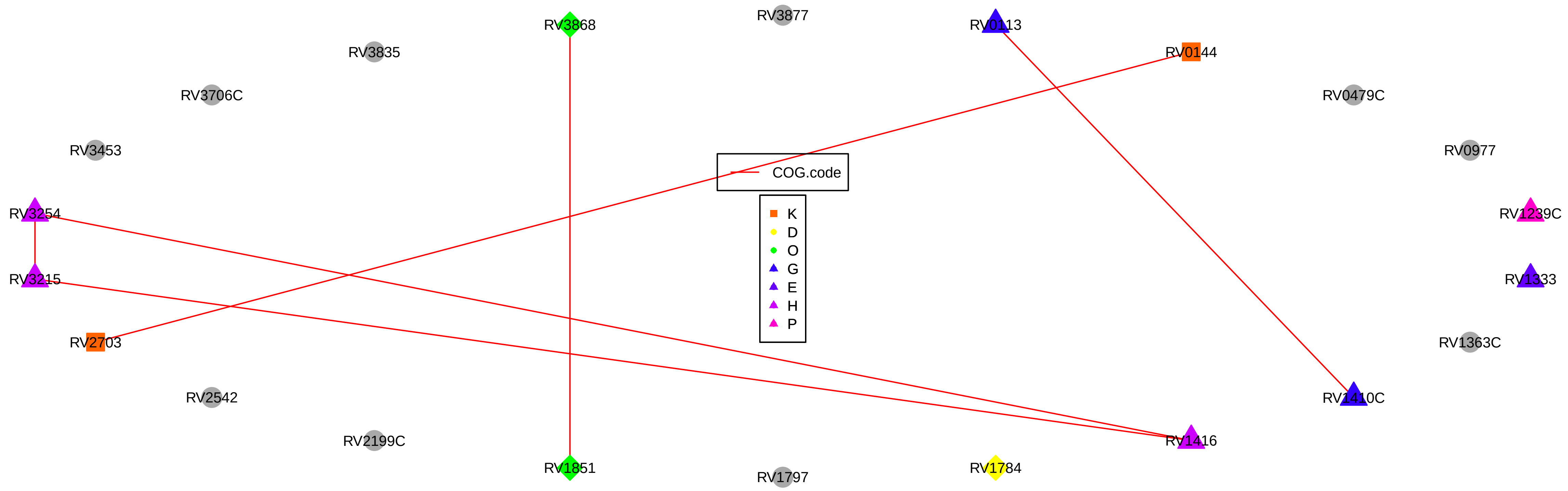

Scaled PSSM #1: E=0.51

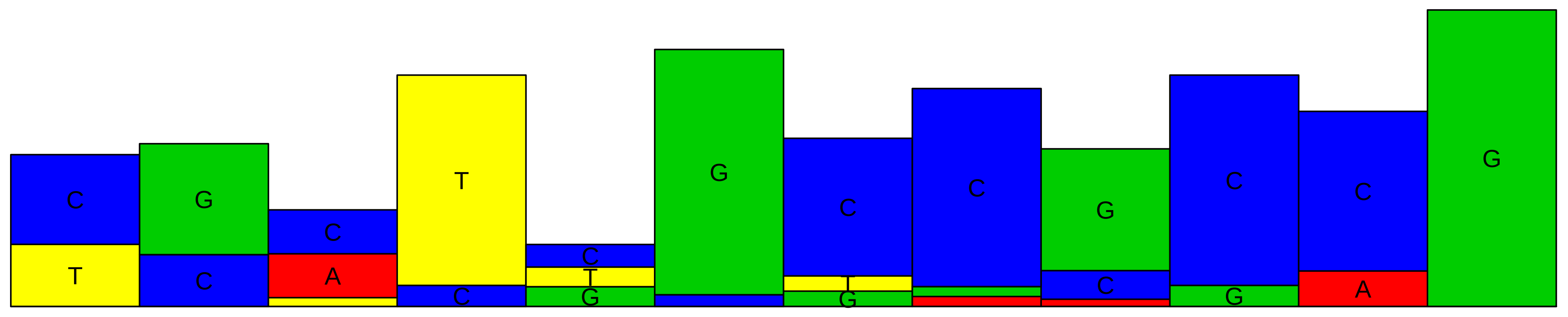

upstream regions

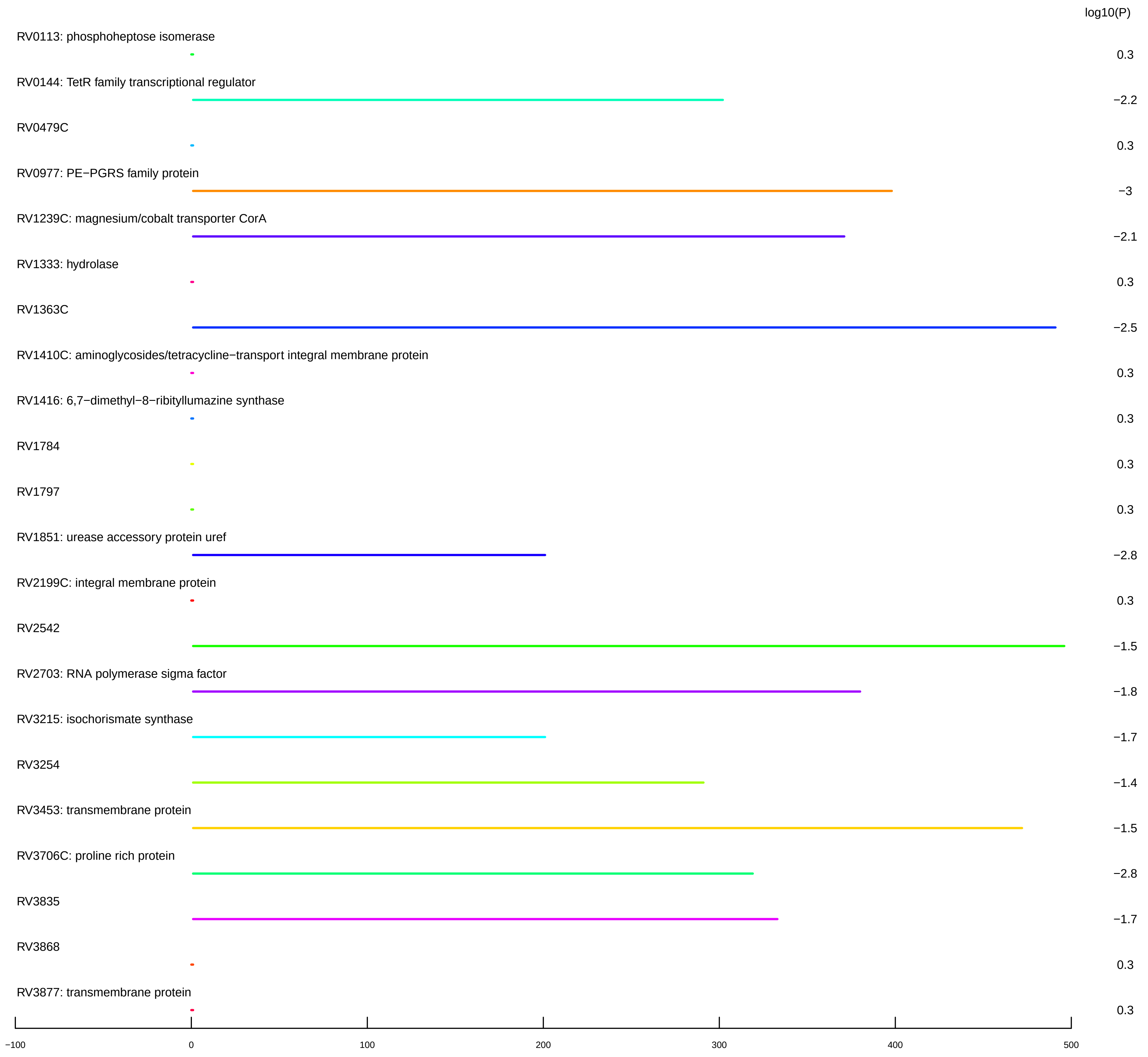

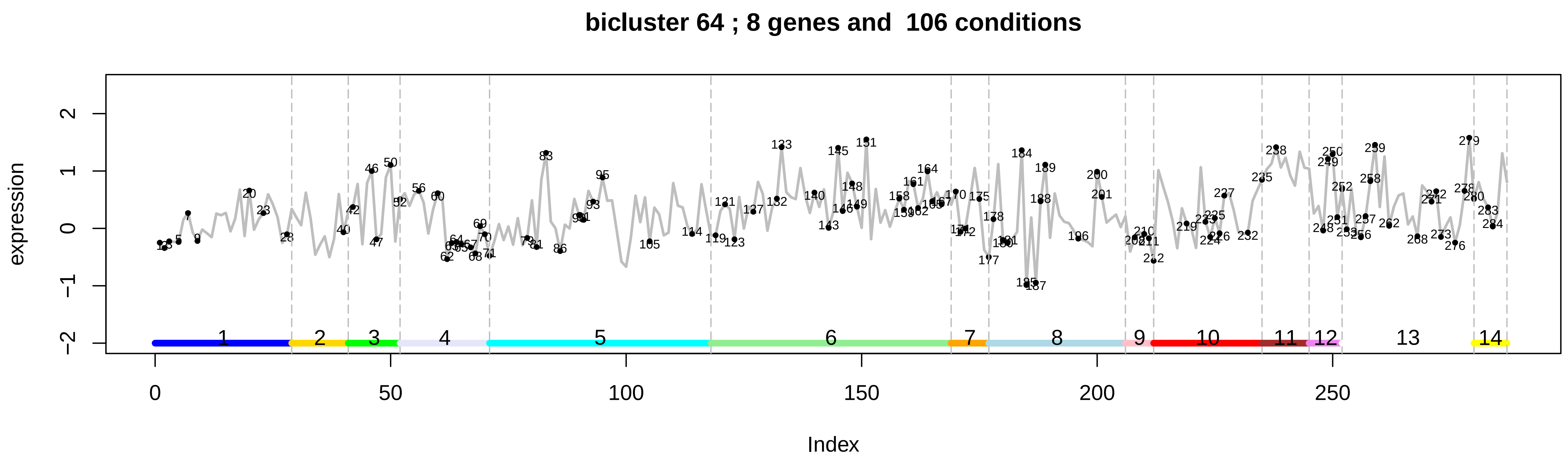
bicluster 64 ; 8 genes and 106 conditions
expression
Index
1
2
3
4
5
6
7
8
9
10
11
12
13
14

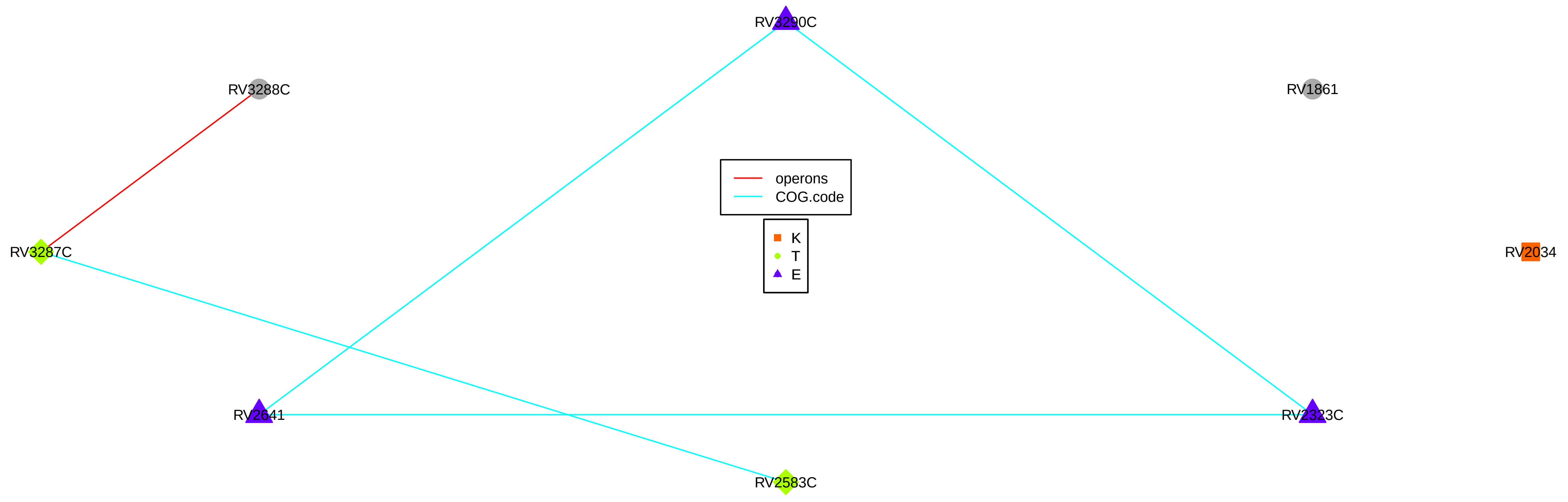
RV3290C
RV3288C
RV1861
RV3287C
RV2034
RV2641
RV2323C
RV2583C
operons
COG.code
K
T
E

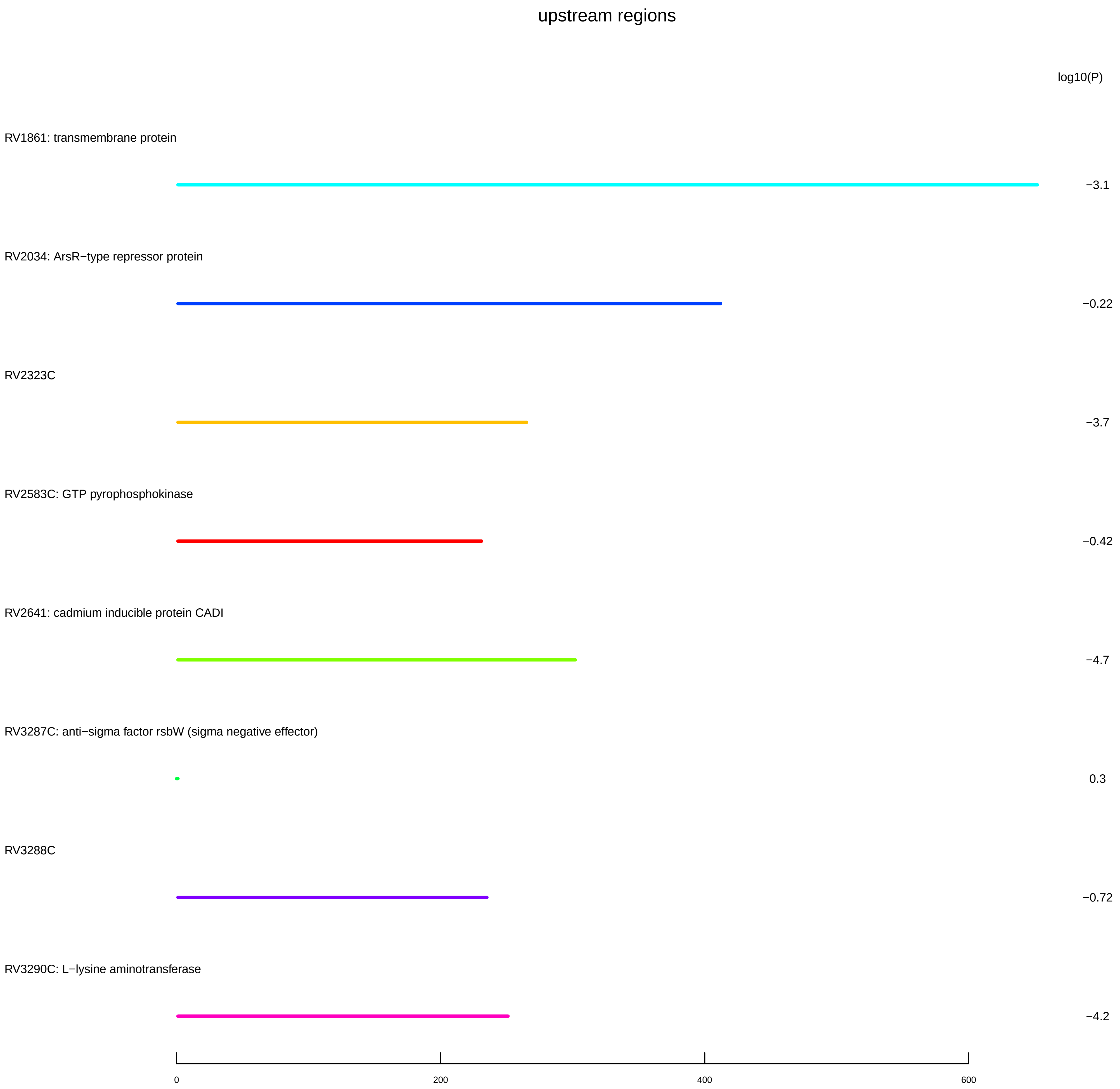
upstream regions
log10(P)
RV1861: transmembrane protein
-3.1
RV2034: ArsR-type repressor protein
-0.22
RV2323C
-3.7
RV2583C: GTP pyrophosphokinase
-0.42
RV2641: cadmium inducible protein CADI
-4.7
RV3287C: anti-sigma factor rsbW (sigma negative effector)
0.3
RV3288C
-0.72
RV3290C: L-lysine aminotransferase
-4.2
0
200
400
600

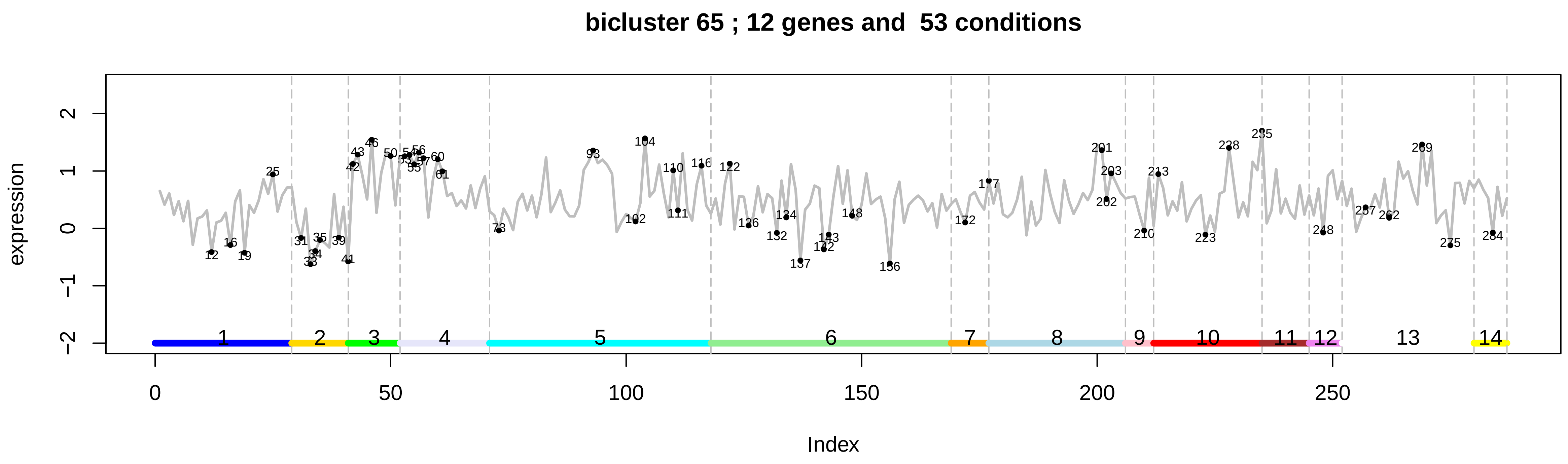
bicluster 65 ; 12 genes and 53 conditions
expression
Index
0
50
100
150
200
250
1
2
3
4
5
6
7
8
9
10
11
12
13
14

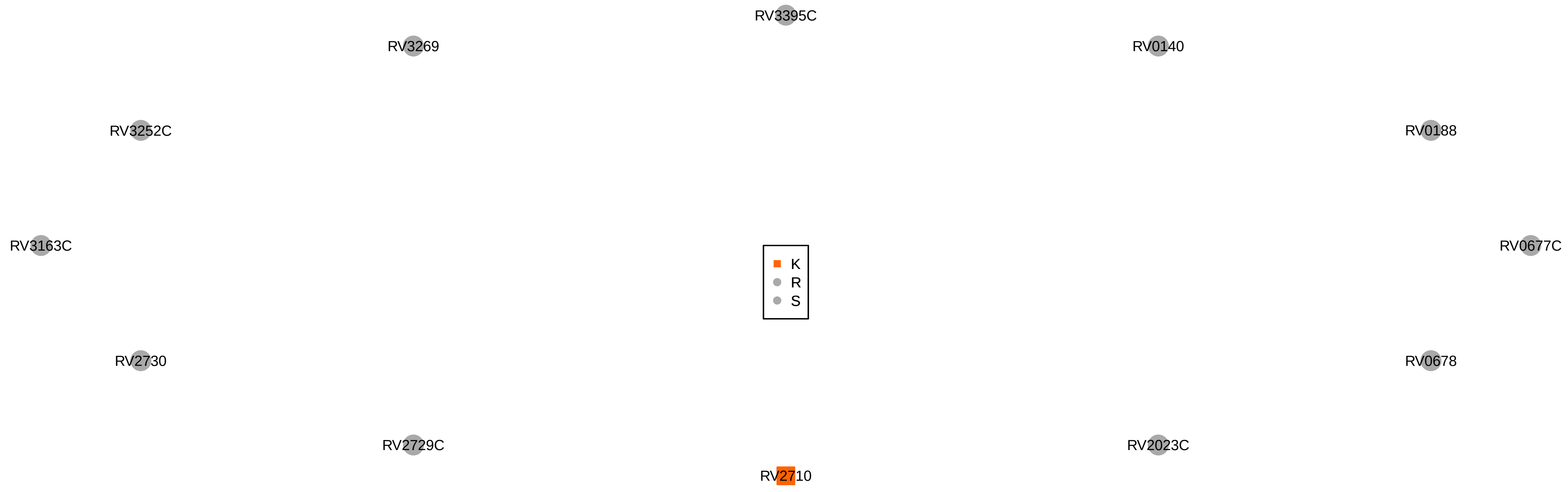
RV3395C
RV3269
RV0140
RV3252C
RV0188
RV3163C
RV0677C
K
R
S
RV2730
RV0678
RV2729C
RV2023C
RV2710

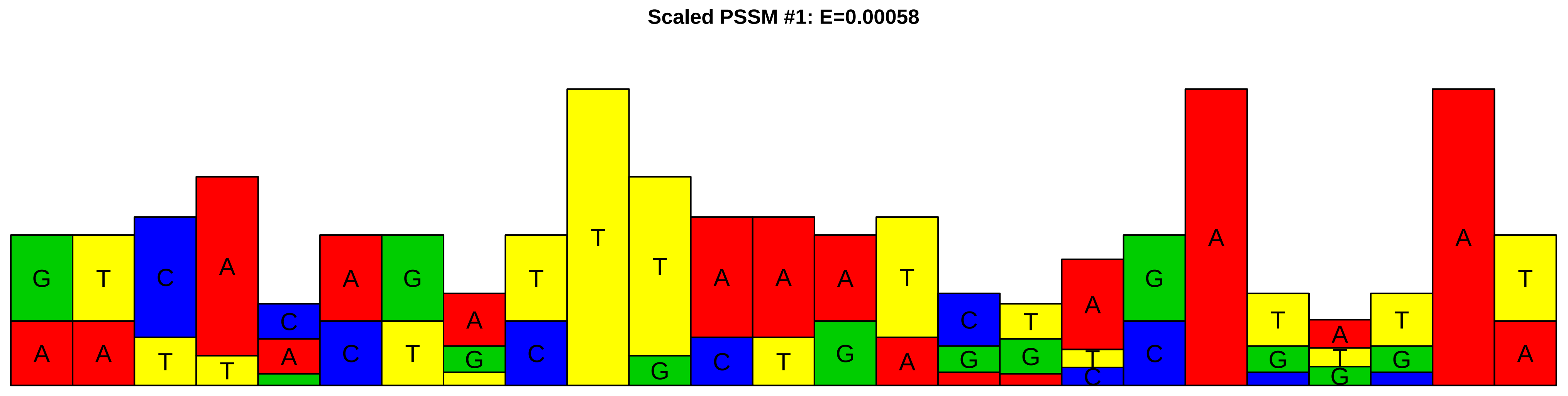
Scaled PSSM #1: E=0.00058

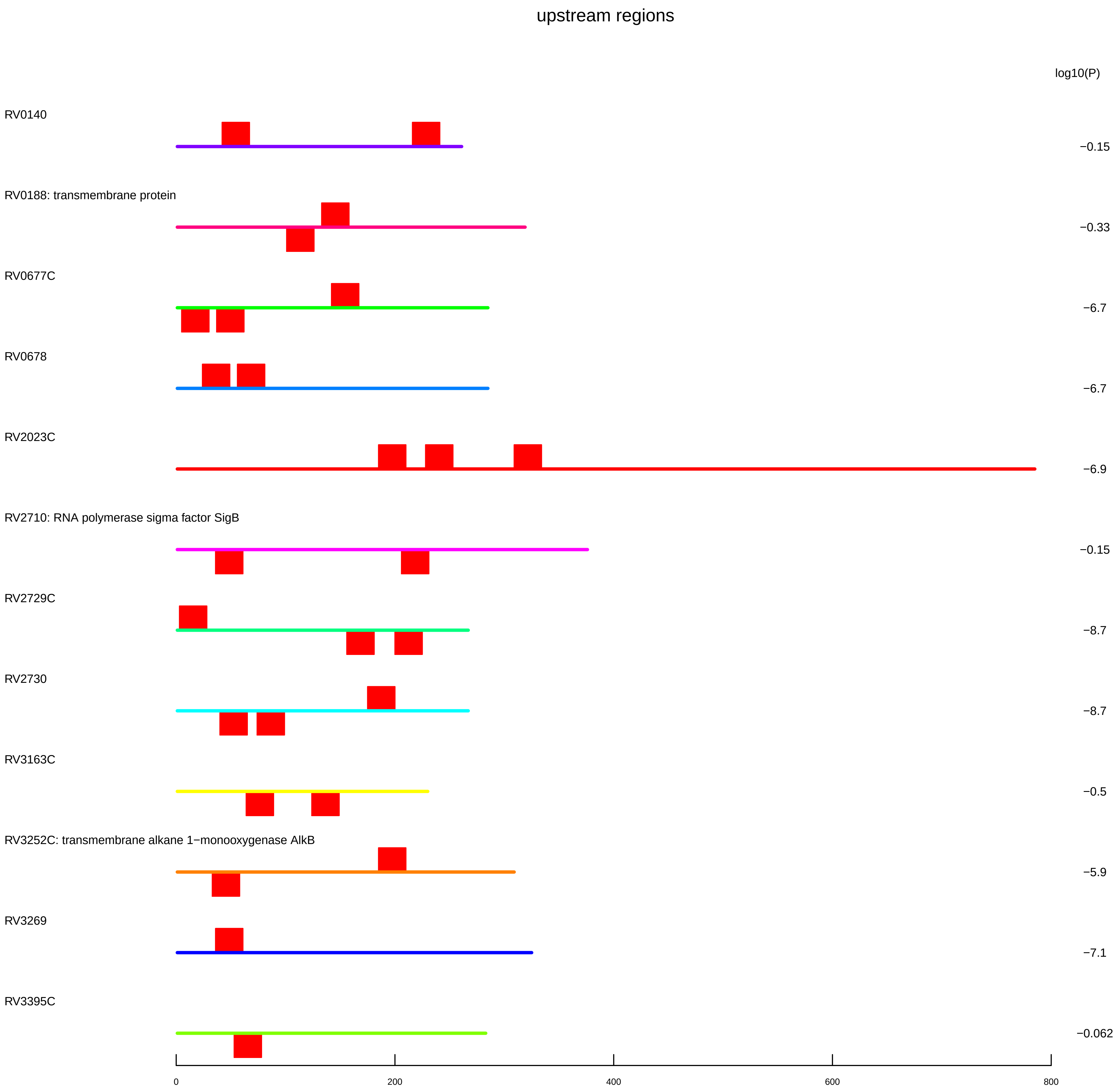
upstream regions
log10(P)
RV0140
-0.15
RV0188: transmembrane protein
-0.33
RV0677C
-6.7
RV0678
-6.7
RV2023C
-6.9
RV2710: RNA polymerase sigma factor SigB
-0.15
RV2729C
-8.7
RV2730
-8.7
RV3163C
-0.5
RV3252C: transmembrane alkane 1-monooxygenase AlkB
-5.9
RV3269
-7.1
RV3395C
-0.062
0
200
400
600
800

bicluster 66 ; 14 genes and 76 conditions

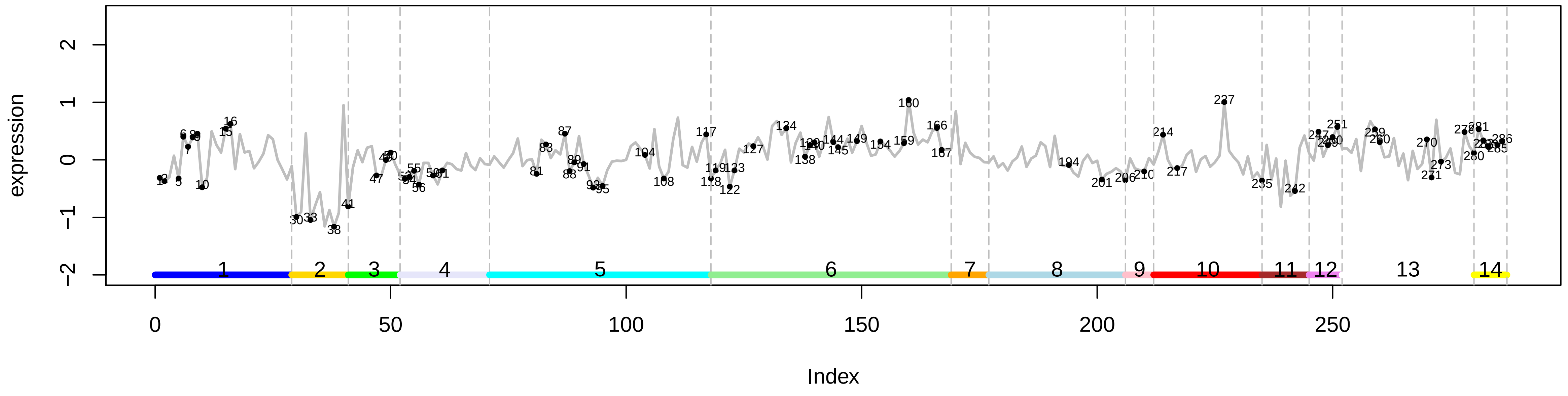

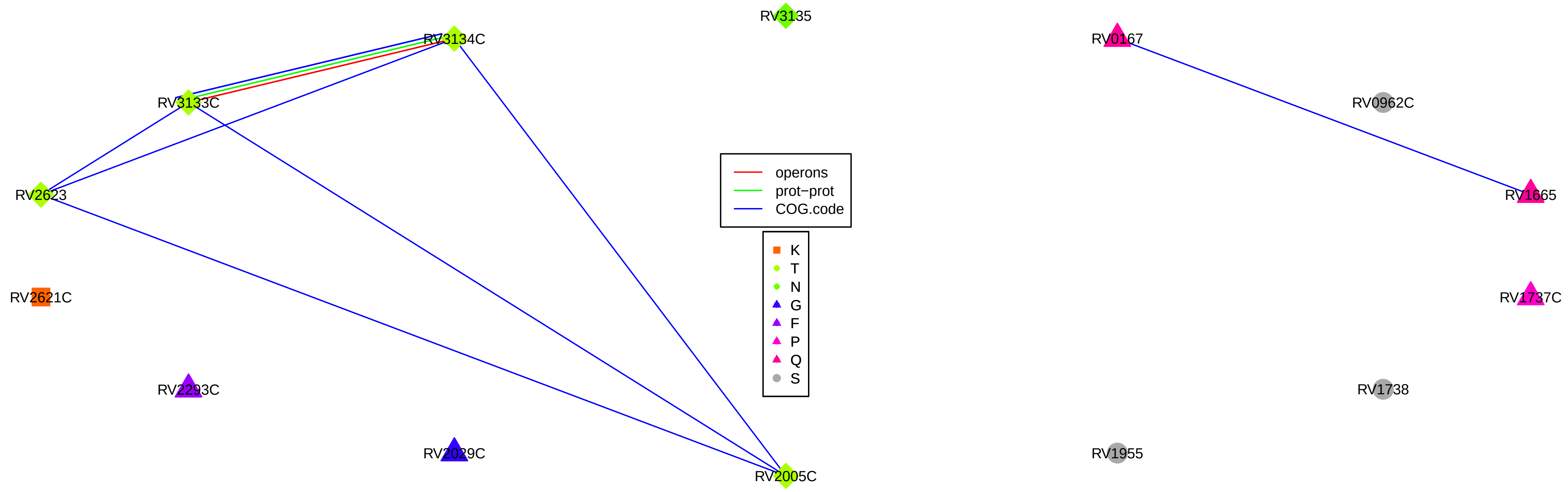

Scaled PSSM #1: E=0.00014

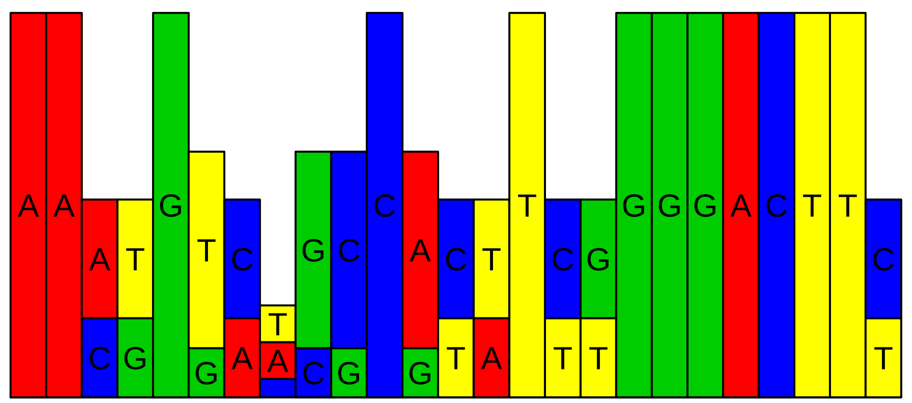

Scaled PSSM #2: E=0.024

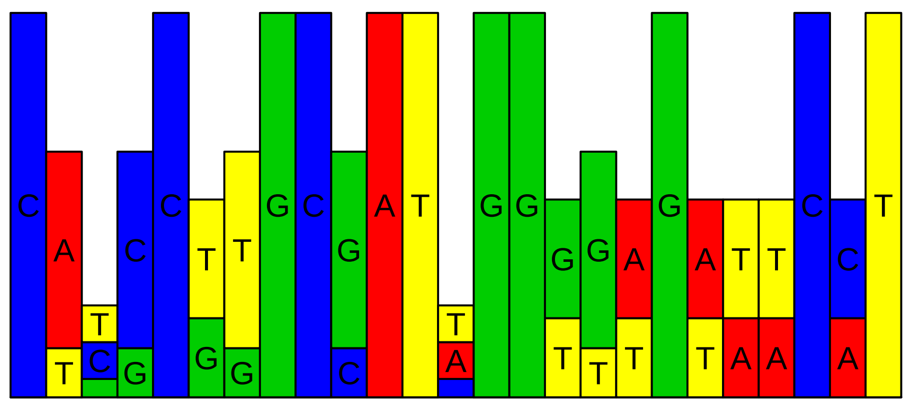

upstream regions

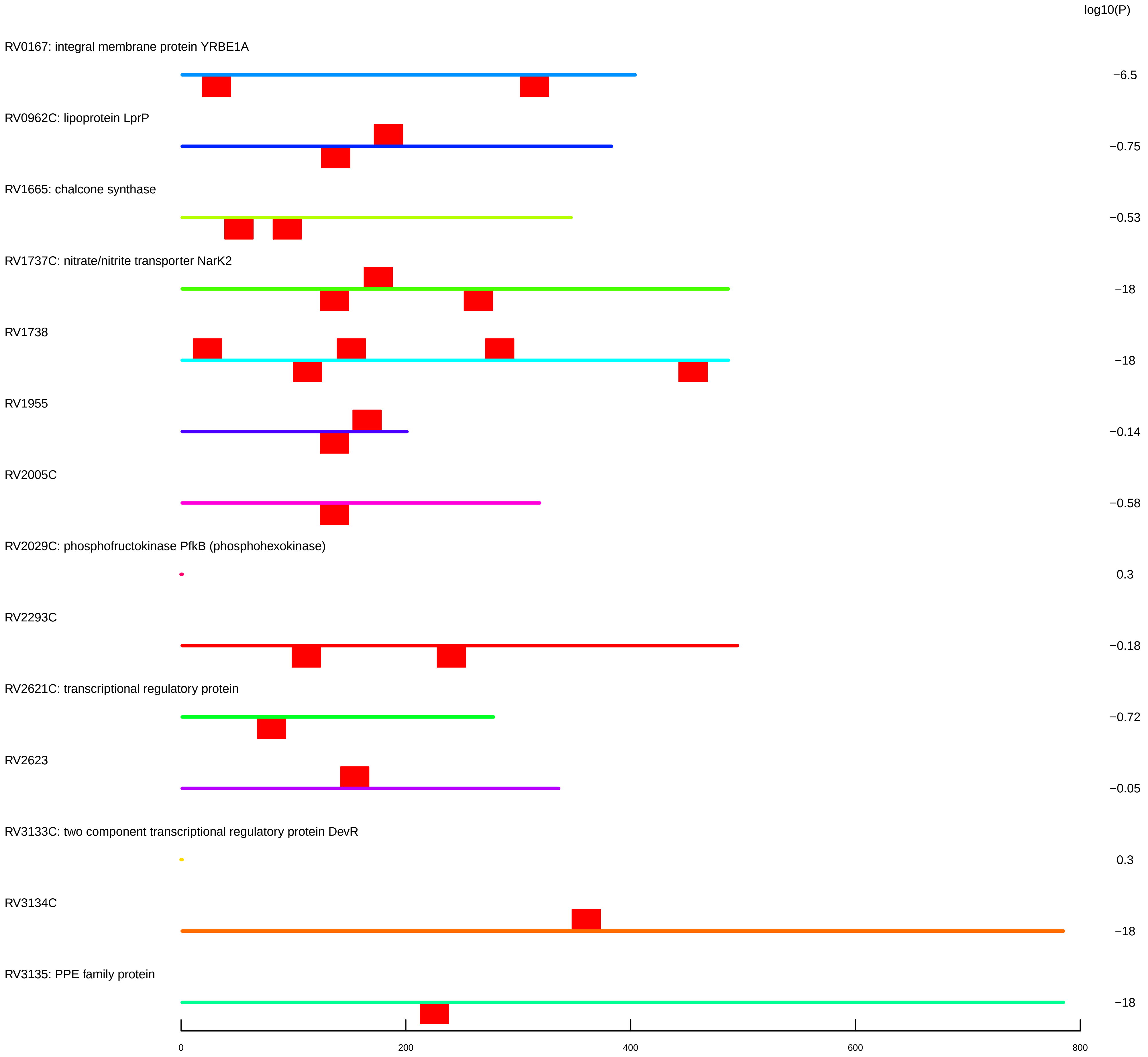

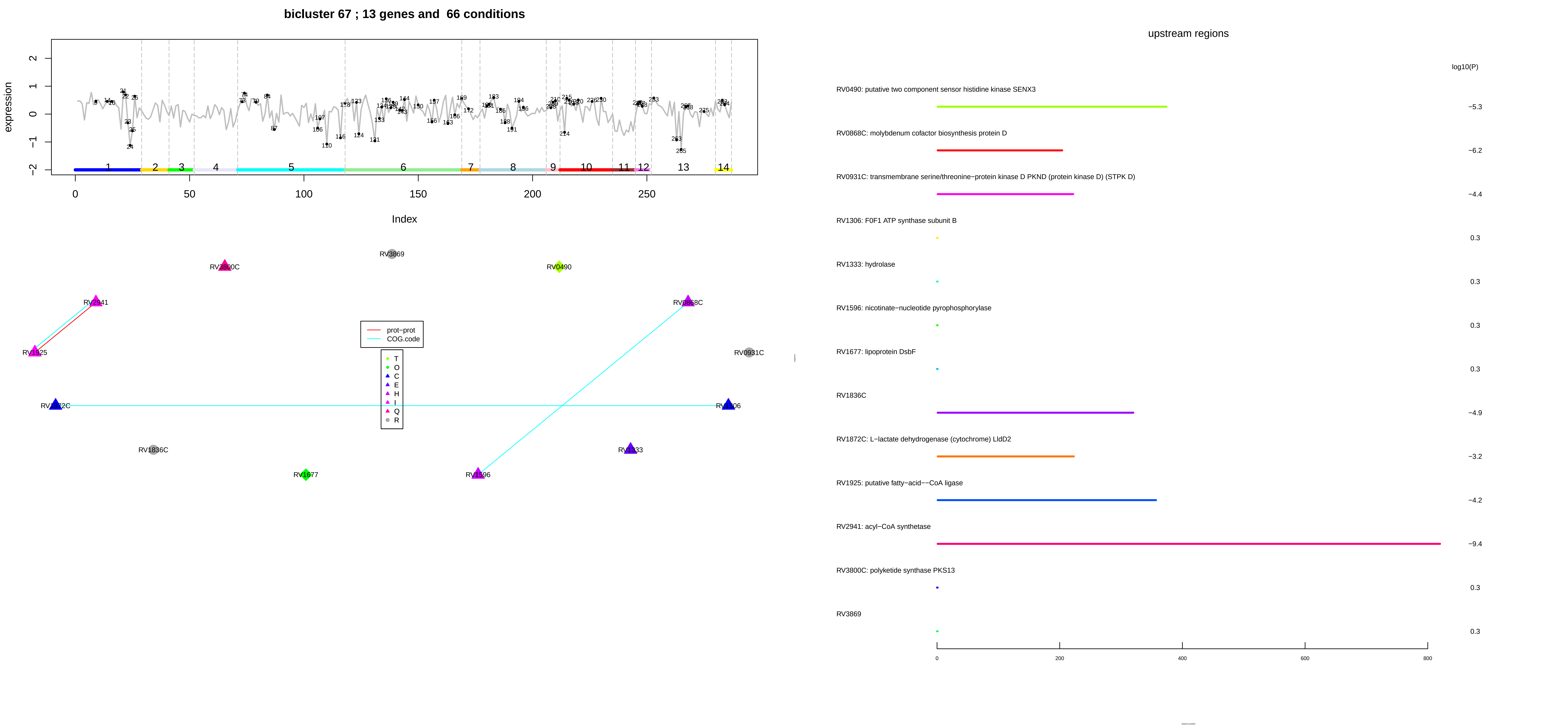

bicluster 67 ; 13 genes and 66 conditions
expression
Index
prot−prot
COG.code
T
O
C
E
H
I
Q
R
RV3869
RV3800C
RV0490
RV2941
RV0868C
RV1925
RV0931C
RV1872C
RV1306
RV1836C
RV1333
RV1677
RV1596
upstream regions
log10(P)
RV0490: putative two component sensor histidine kinase SENX3
−5.3
RV0868C: molybdenum cofactor biosynthesis protein D
−6.2
RV0931C: transmembrane serine/threonine−protein kinase D PKND (protein kinase D) (STPK D)
−4.4
RV1306: F0F1 ATP synthase subunit B
0.3
RV1333: hydrolase
0.3
RV1596: nicotinate−nucleotide pyrophosphorylase
0.3
RV1677: lipoprotein DsbF
0.3
RV1836C
−4.9
RV1872C: L−lactate dehydrogenase (cytochrome) LldD2
−3.2
RV1925: putative fatty−acid−−CoA ligase
−4.2
RV2941: acyl−CoA synthetase
−9.4
RV3800C: polyketide synthase PKS13
0.3
RV3869
0.3

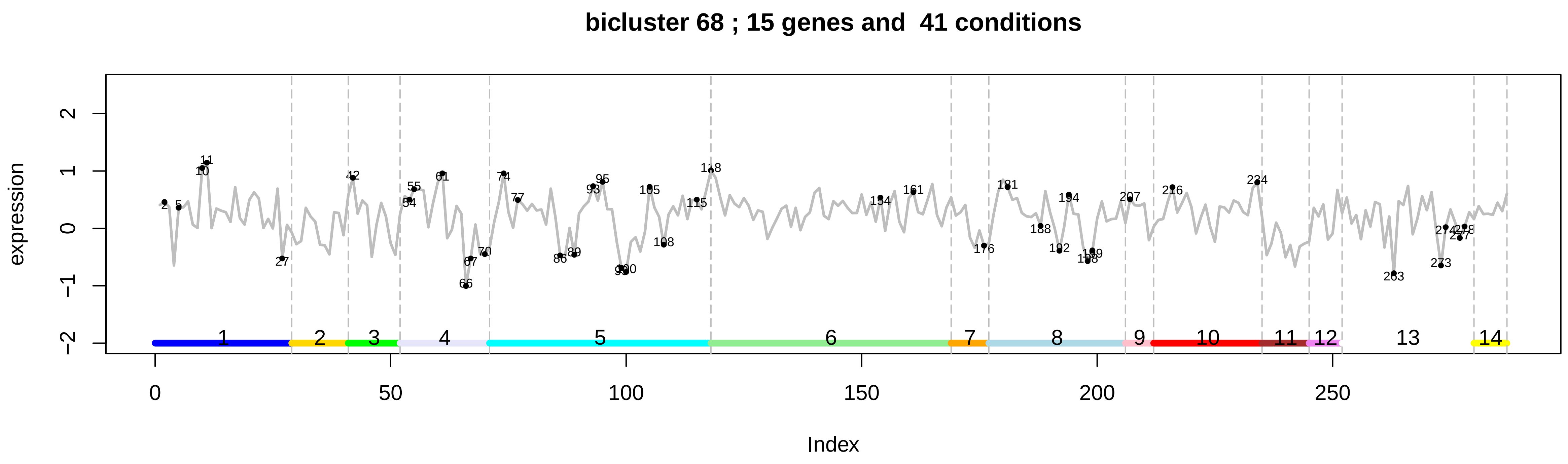
bicluster 68 ; 15 genes and 41 conditions
expression
Index
1
2
3
4
5
6
7
8
9
10
11
12
13
14

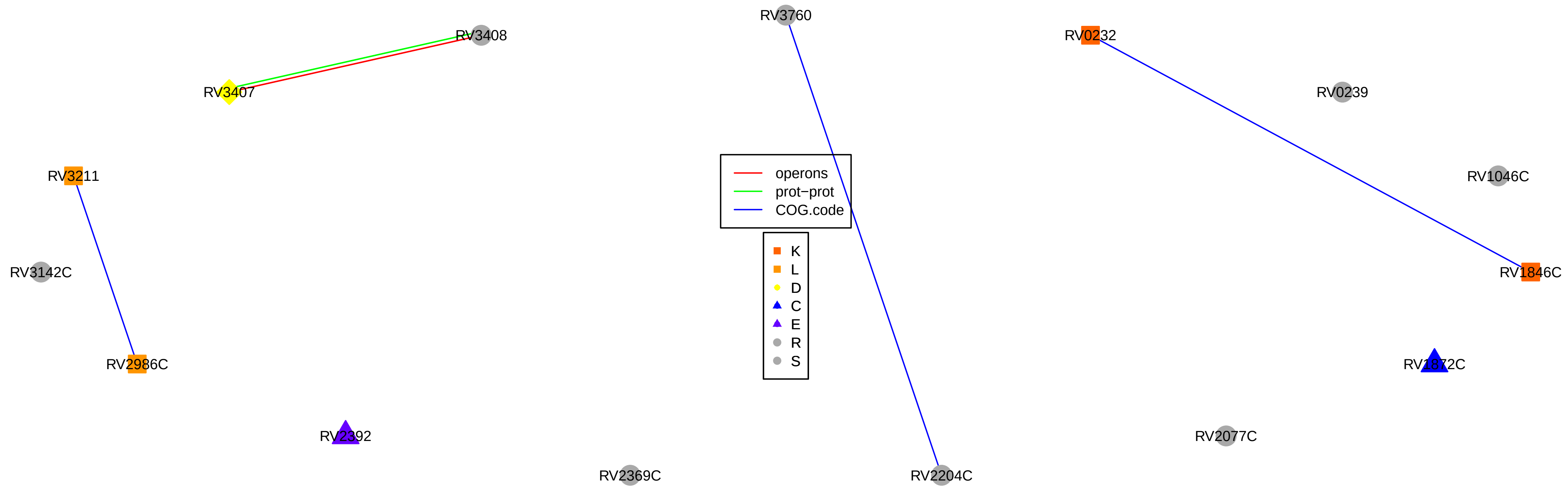
RV3408
RV3407
RV3760
RV0232
RV0239
RV1046C
RV3211
RV3142C
RV1846C
RV2986C
RV2392
RV2369C
RV2204C
RV2077C
operons
prot−prot
COG.code
K
L
D
C
E
R
S

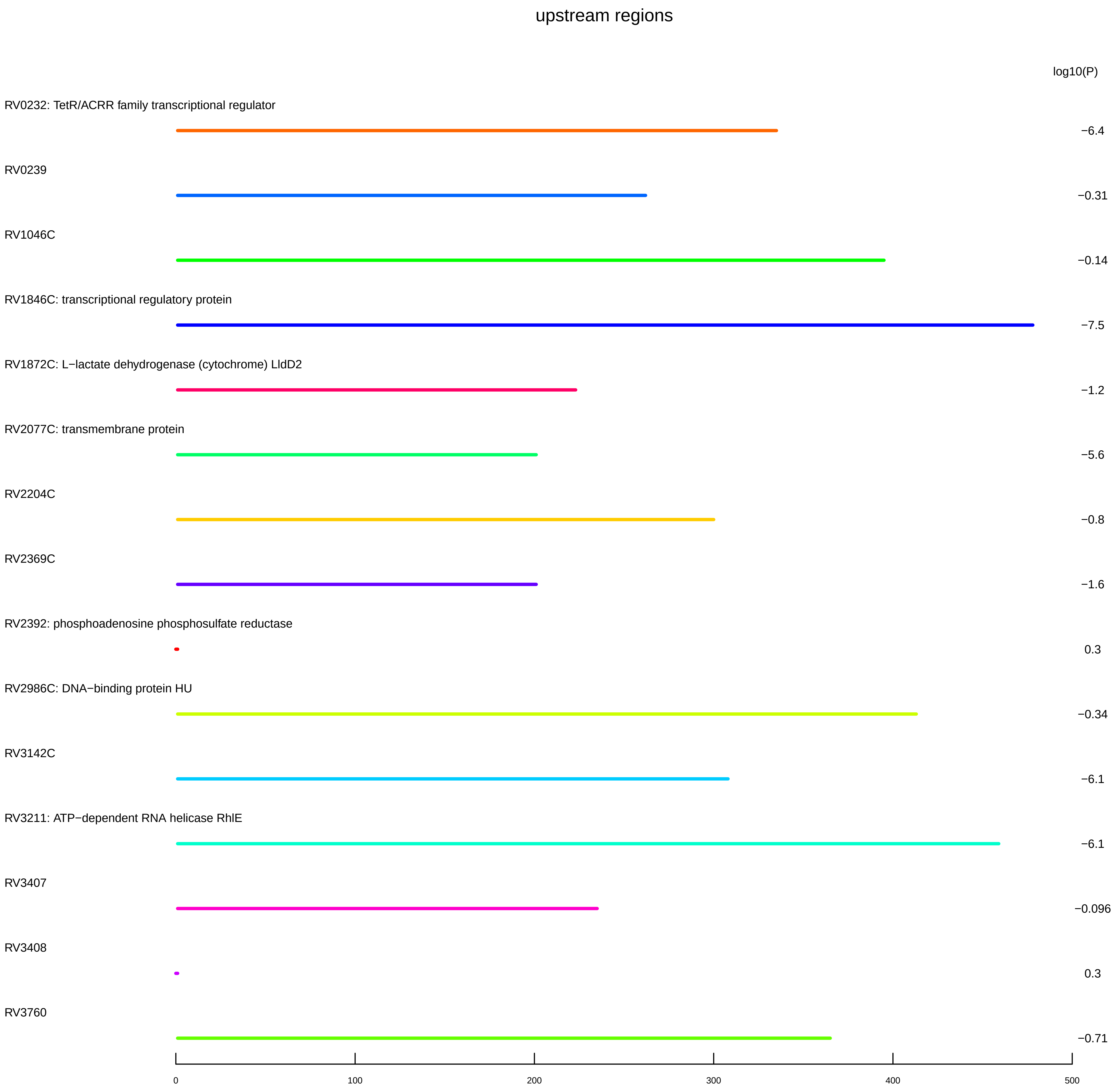
upstream regions
log10(P)
RV0232: TetR/ACRR family transcriptional regulator
−6.4
RV0239
−0.31
RV1046C
−0.14
RV1846C: transcriptional regulatory protein
−7.5
RV1872C: L−lactate dehydrogenase (cytochrome) LldD2
−1.2
RV2077C: transmembrane protein
−5.6
RV2204C
−0.8
RV2369C
−1.6
RV2392: phosphoadenosine phosphosulfate reductase
0.3
RV2986C: DNA−binding protein HU
−0.34
RV3142C
−6.1
RV3211: ATP−dependent RNA helicase RhlE
−6.1
RV3407
−0.096
RV3408
0.3
RV3760
−0.71
0
100
200
300
400
500

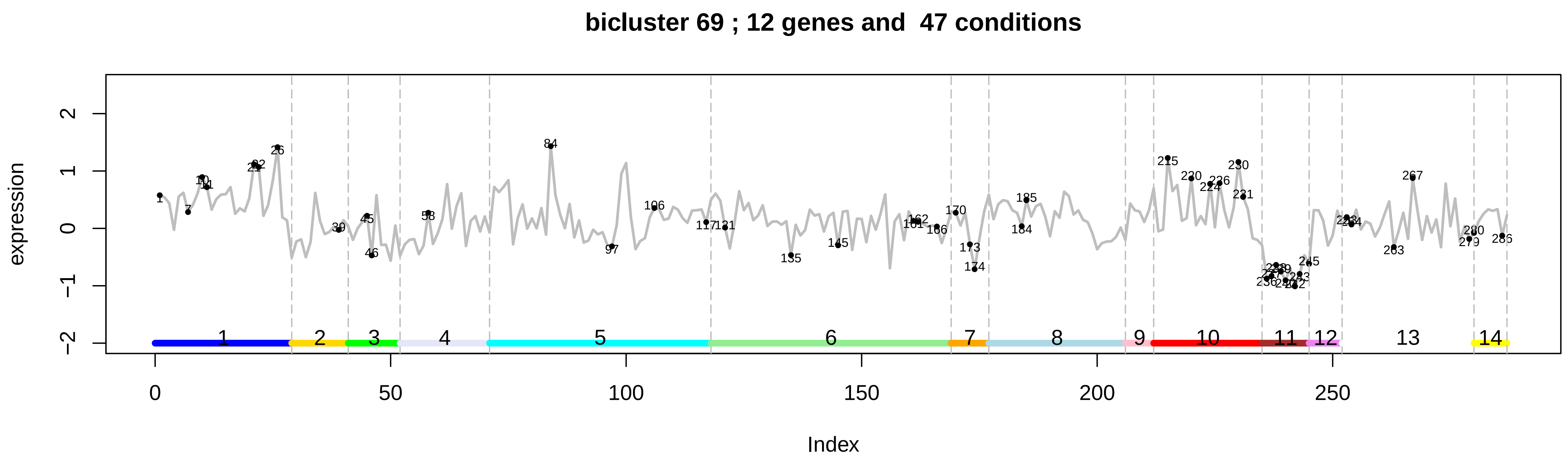
bicluster 69 ; 12 genes and 47 conditions
expression
Index
1
2
3
4
5
6
7
8
9
10
11
12
13
14

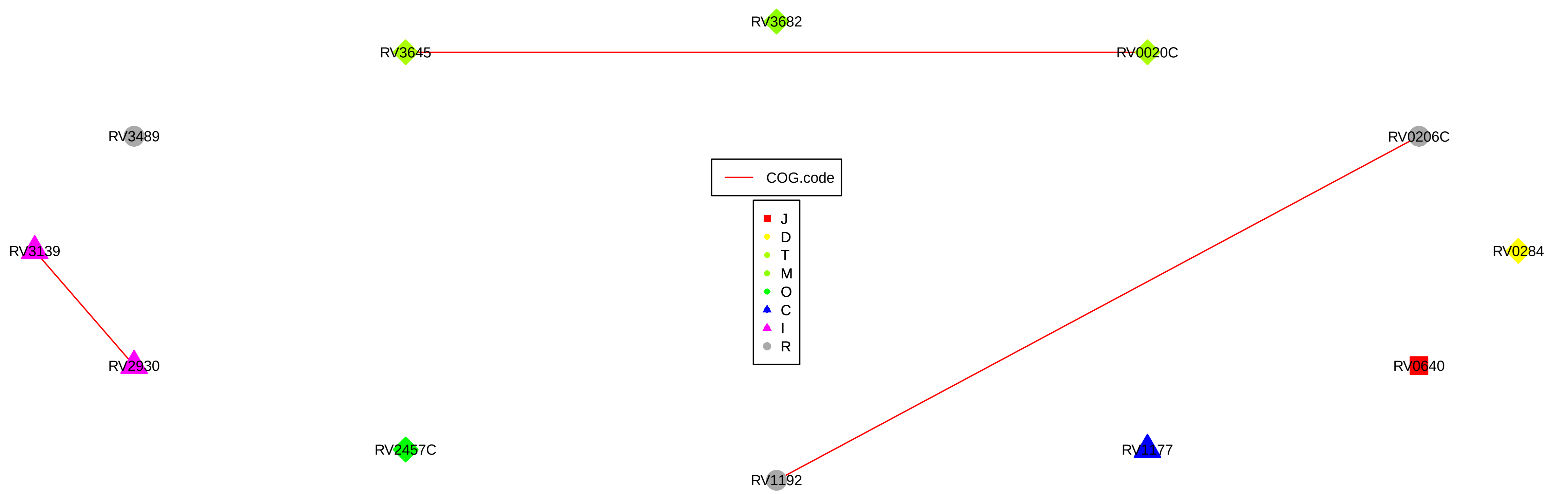
RV3682
RV3645
RV0020C
RV3489
RV0206C
COG.code
J
D
T
M
O
C
I
R
RV3139
RV0284
RV2930
RV0640
RV2457C
RV1192

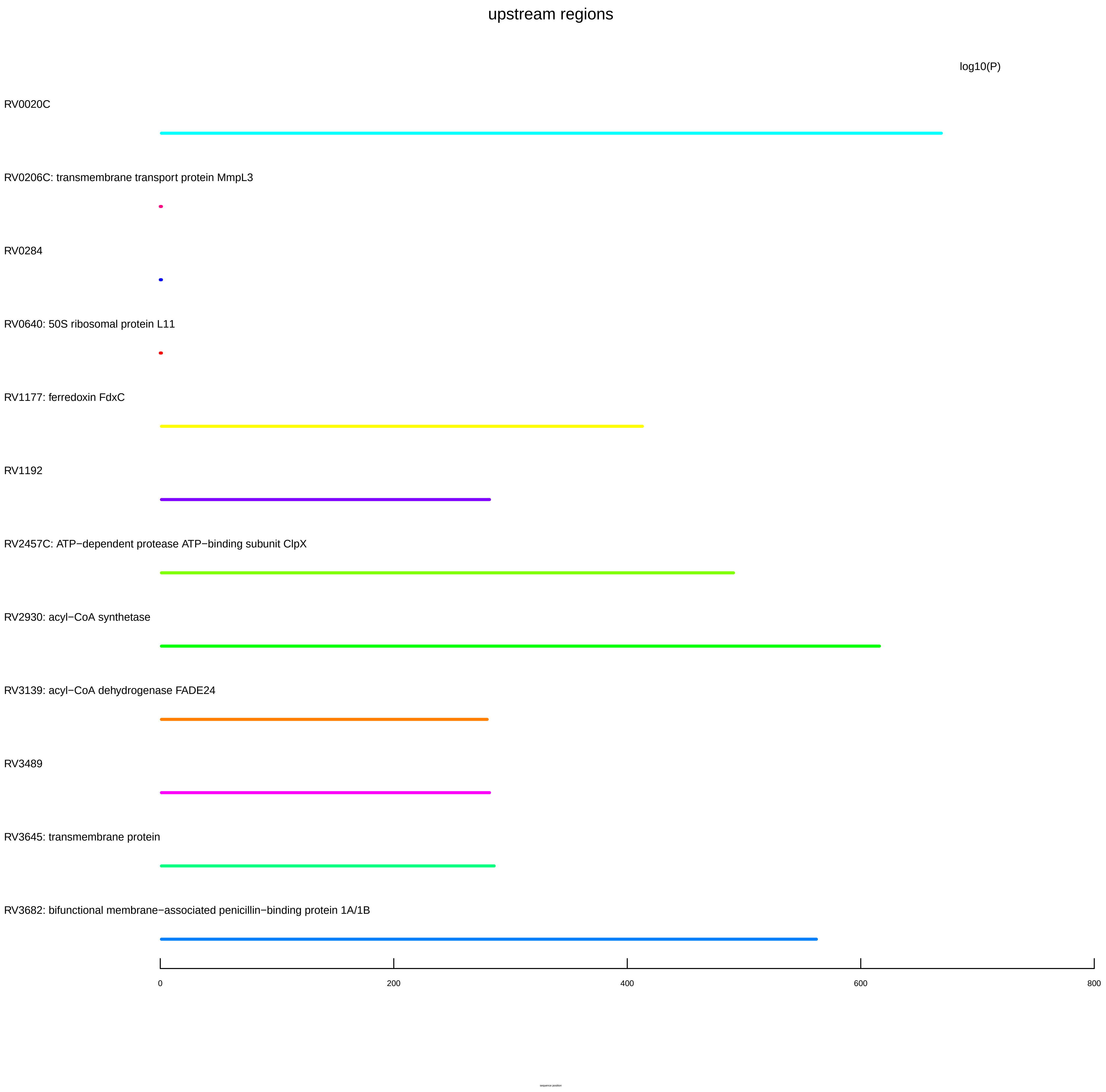
upstream regions
log10(P)
RV0020C
RV0206C: transmembrane transport protein MmpL3
RV0284
RV0640: 50S ribosomal protein L11
RV1177: ferredoxin FdxC
RV1192
RV2457C: ATP−dependent protease ATP−binding subunit ClpX
RV2930: acyl−CoA synthetase
RV3139: acyl−CoA dehydrogenase FADE24
RV3489
RV3645: transmembrane protein
RV3682: bifunctional membrane−associated penicillin−binding protein 1A/1B
0
200
400
600
800

bicluster 70 ; 10 genes and 84 conditions

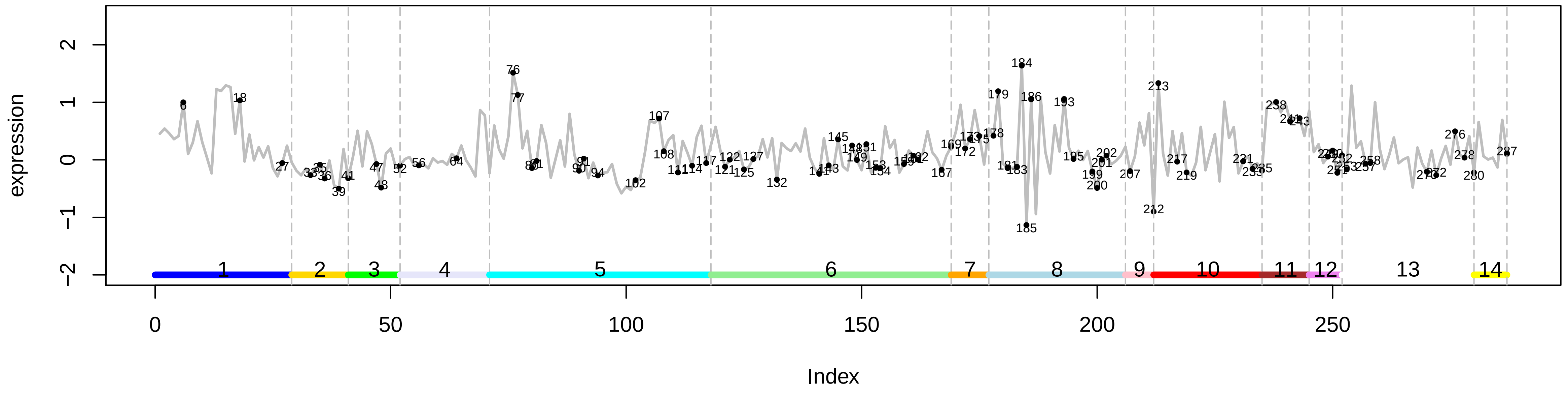

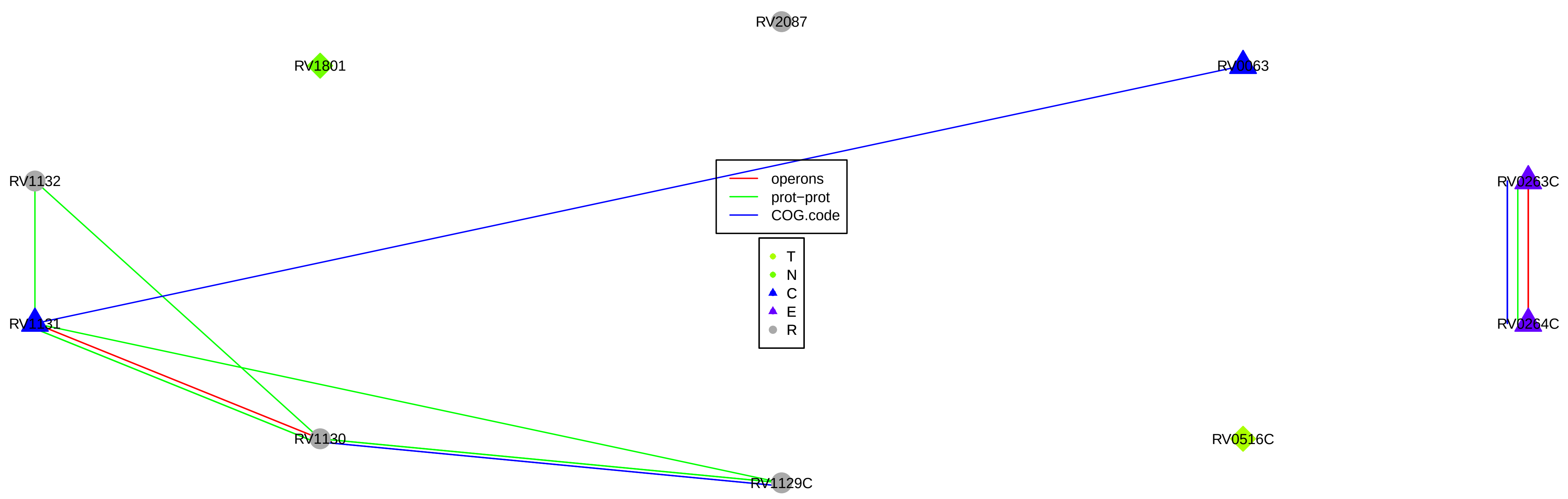

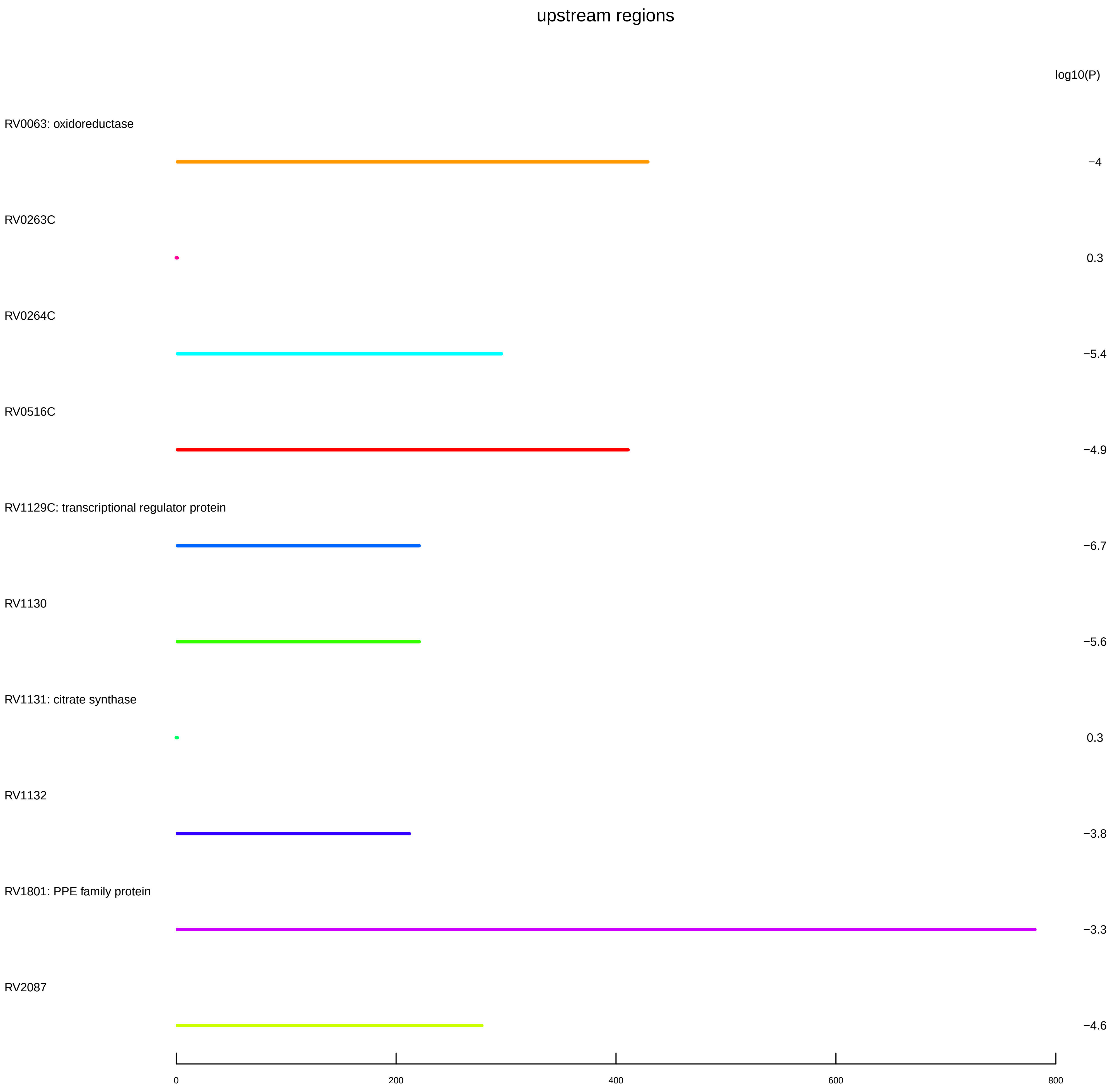

bicluster 71 ; 9 genes and 48 conditions

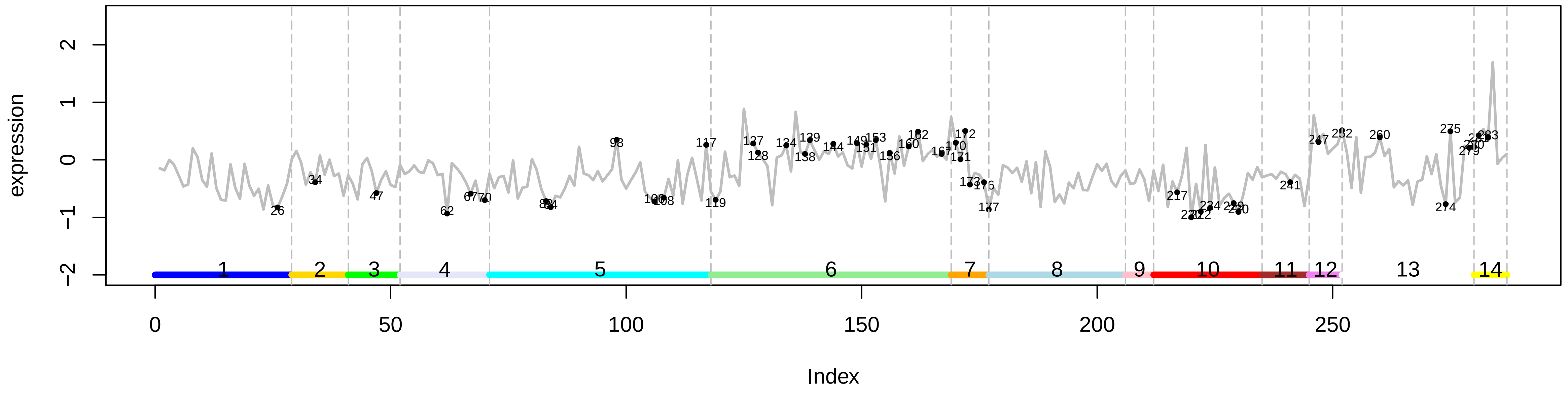

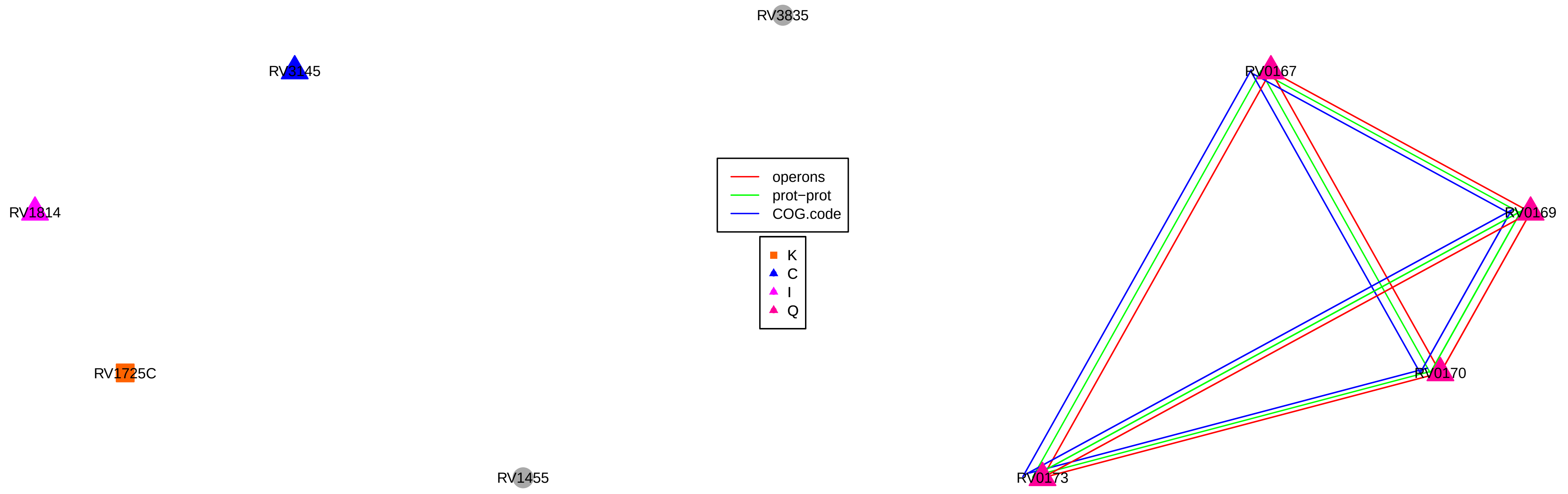

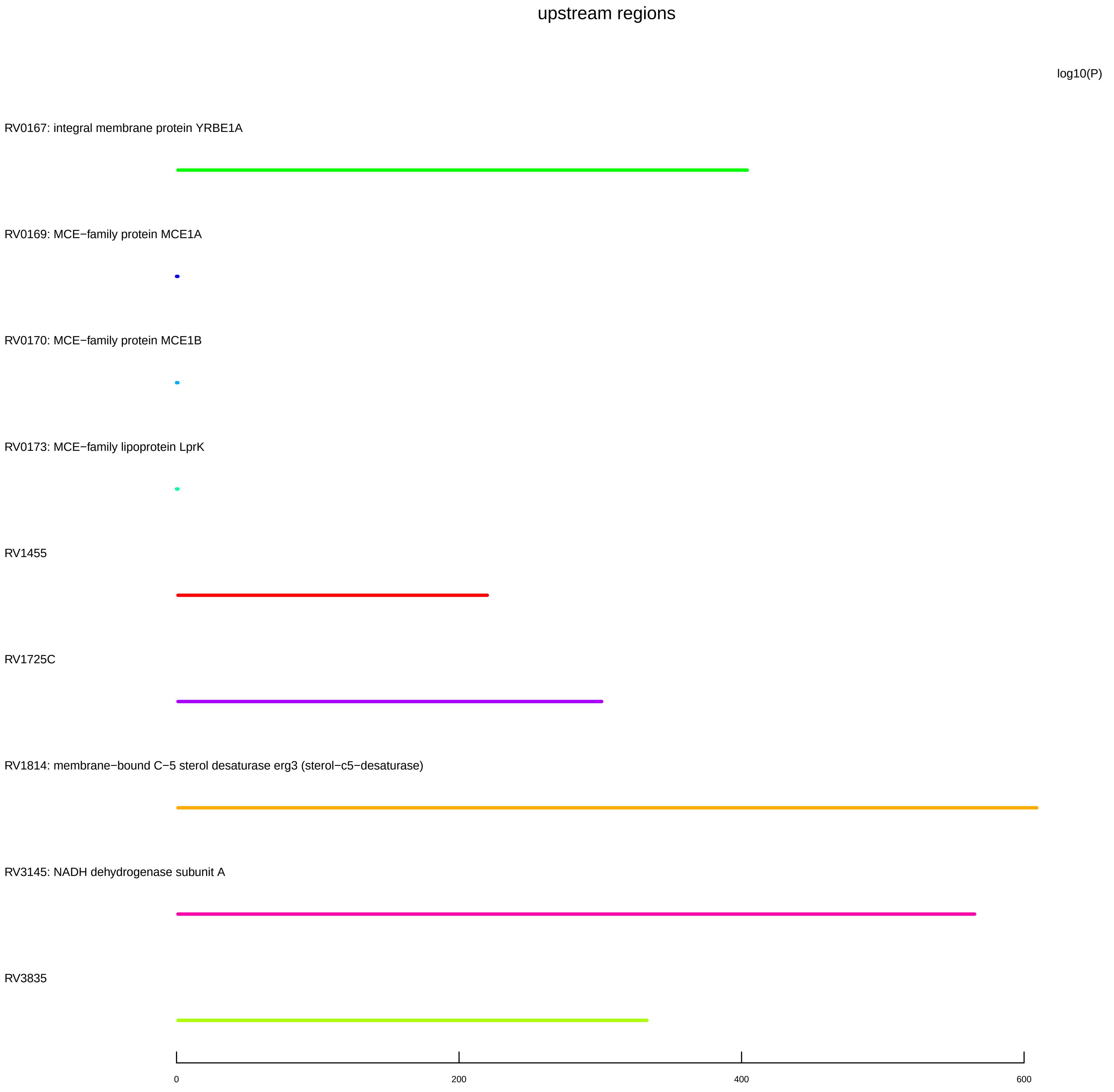

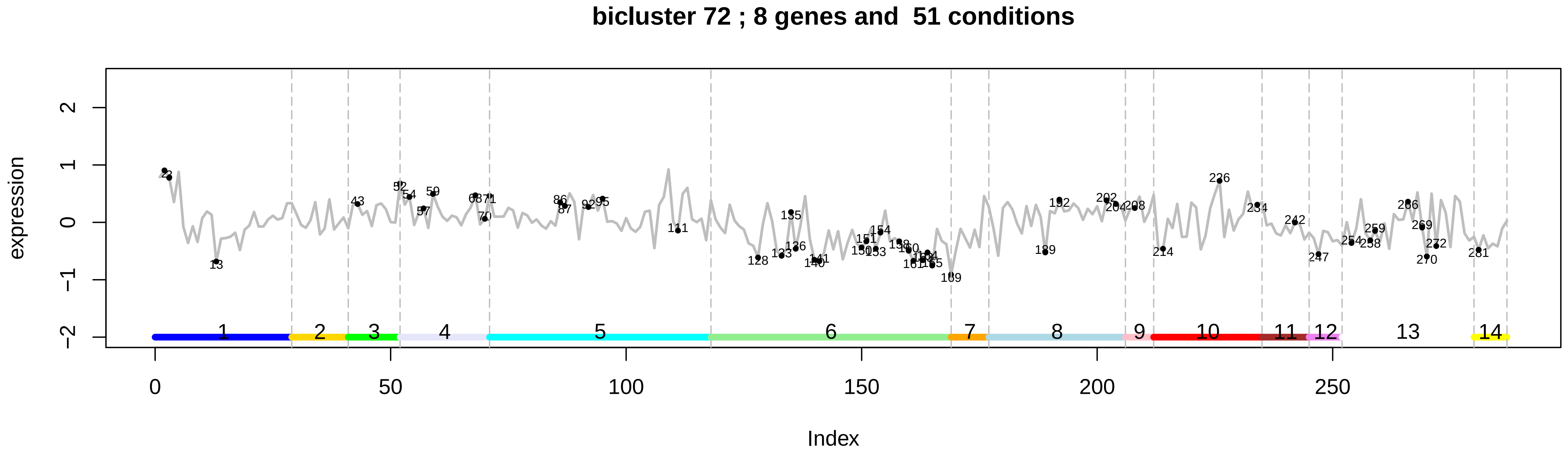
bicluster 72 ; 8 genes and 51 conditions
expression
Index
0
50
100
150
200
250
−2
−1
0
1
2
1
2
3
4
5
6
7
8
9
10
11
12
13
14

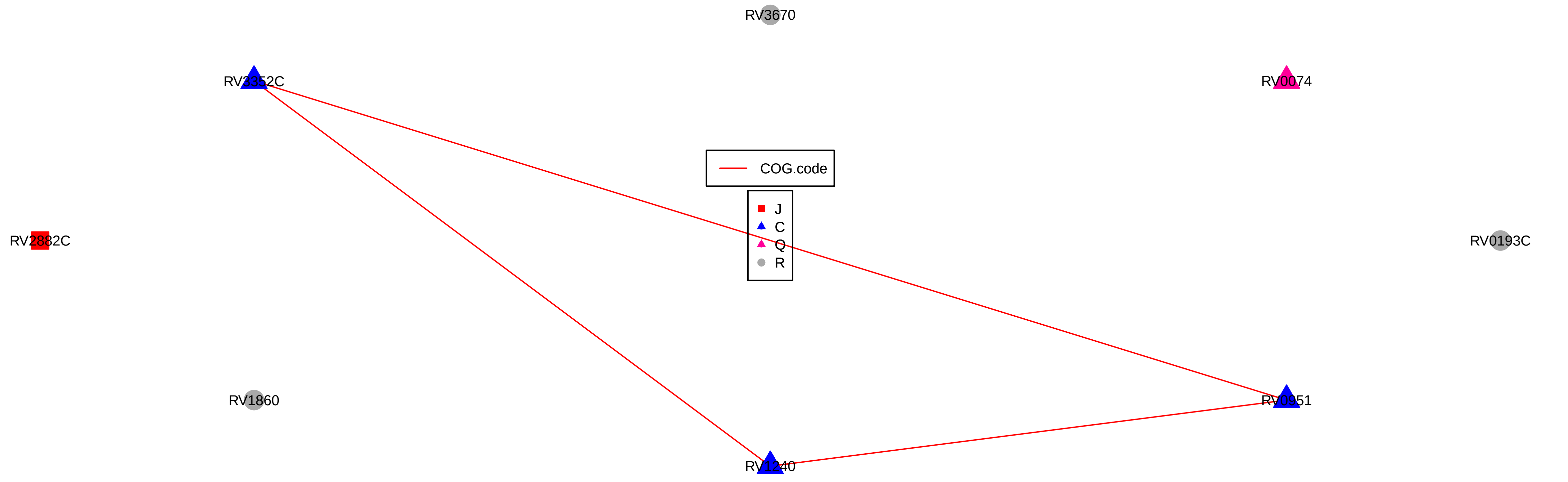
RV3670
RV3352C
RV0074
COG.code
J
C
Q
R
RV2882C
RV0193C
RV1860
RV0951
RV1240

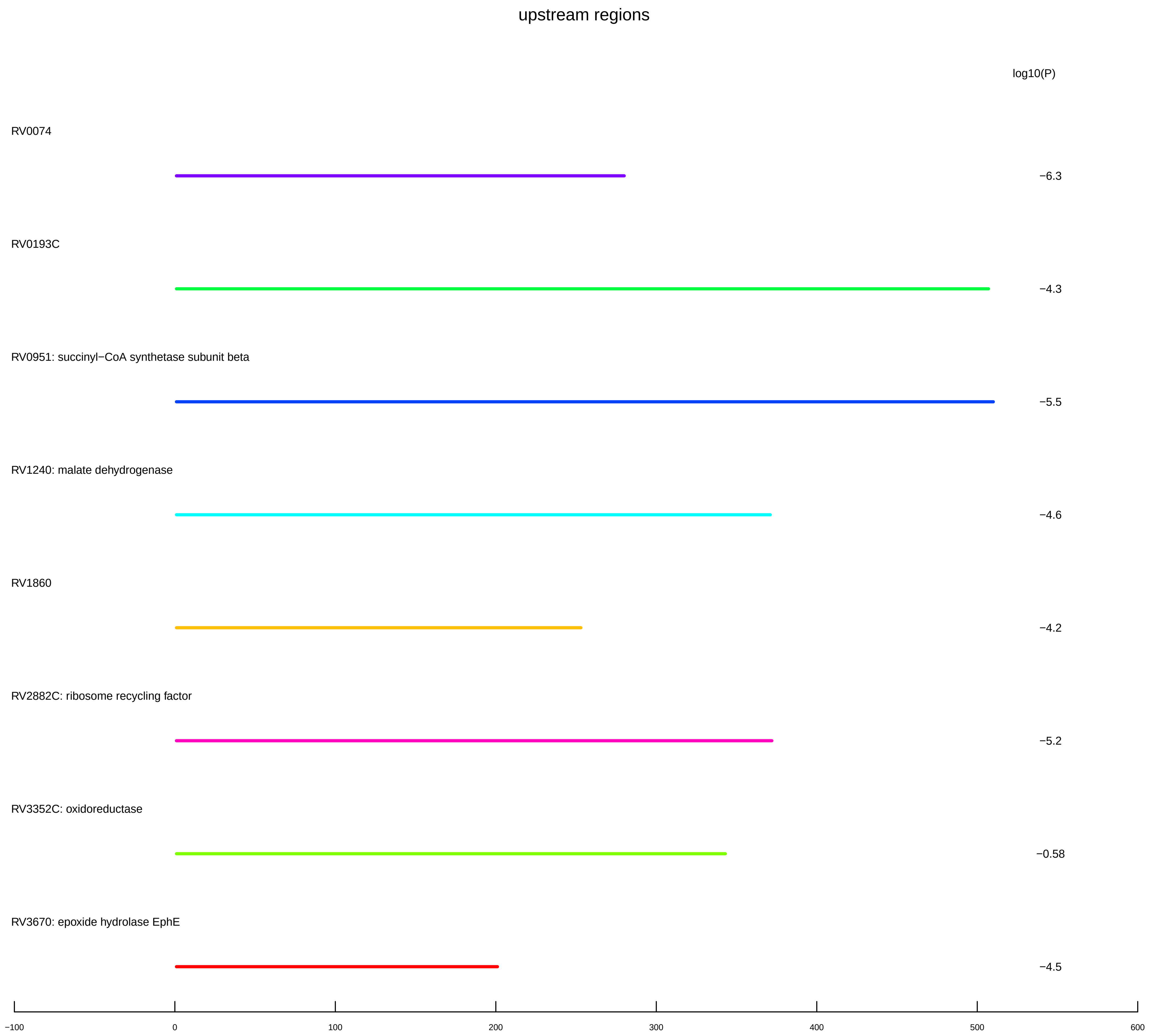
upstream regions
log10(P)
RV0074
−6.3
RV0193C
−4.3
RV0951: succinyl−CoA synthetase subunit beta
−5.5
RV1240: malate dehydrogenase
−4.6
RV1860
−4.2
RV2882C: ribosome recycling factor
−5.2
RV3352C: oxidoreductase
−0.58
RV3670: epoxide hydrolase EphE
−4.5
−100
0
100
200
300
400
500
600

bicluster 73 ; 7 genes and 61 conditions

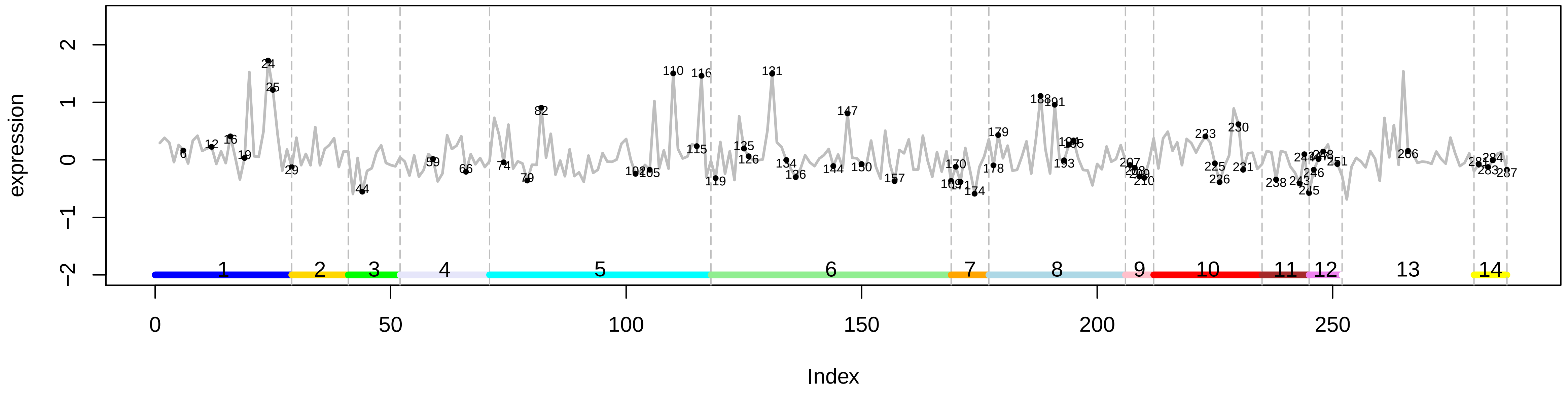

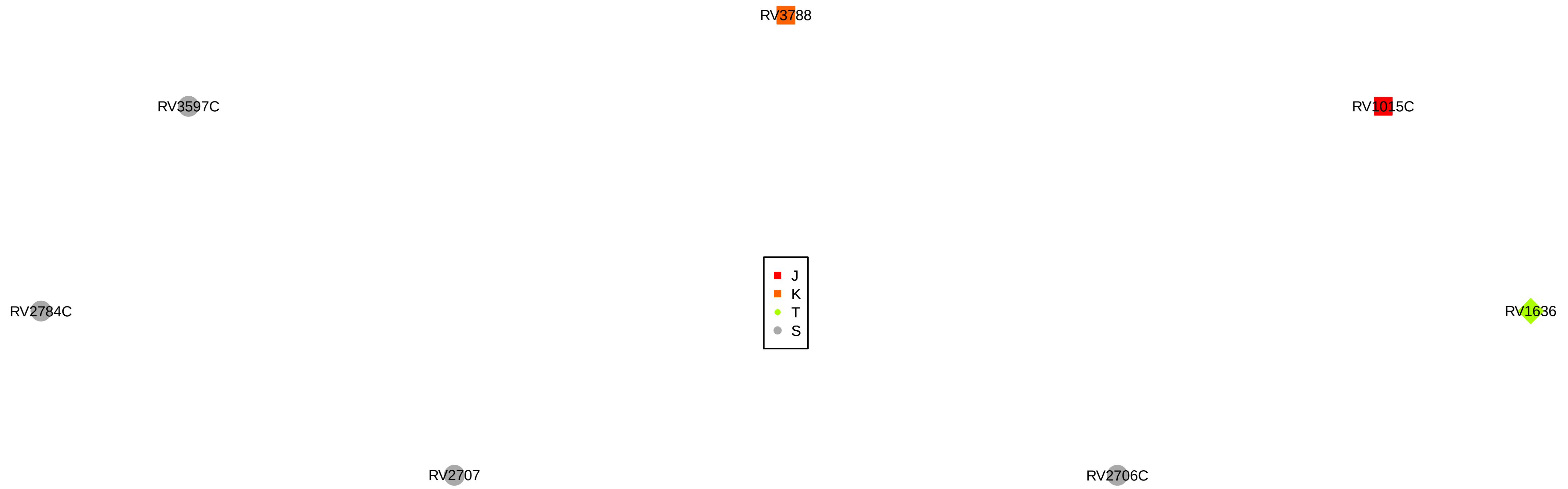

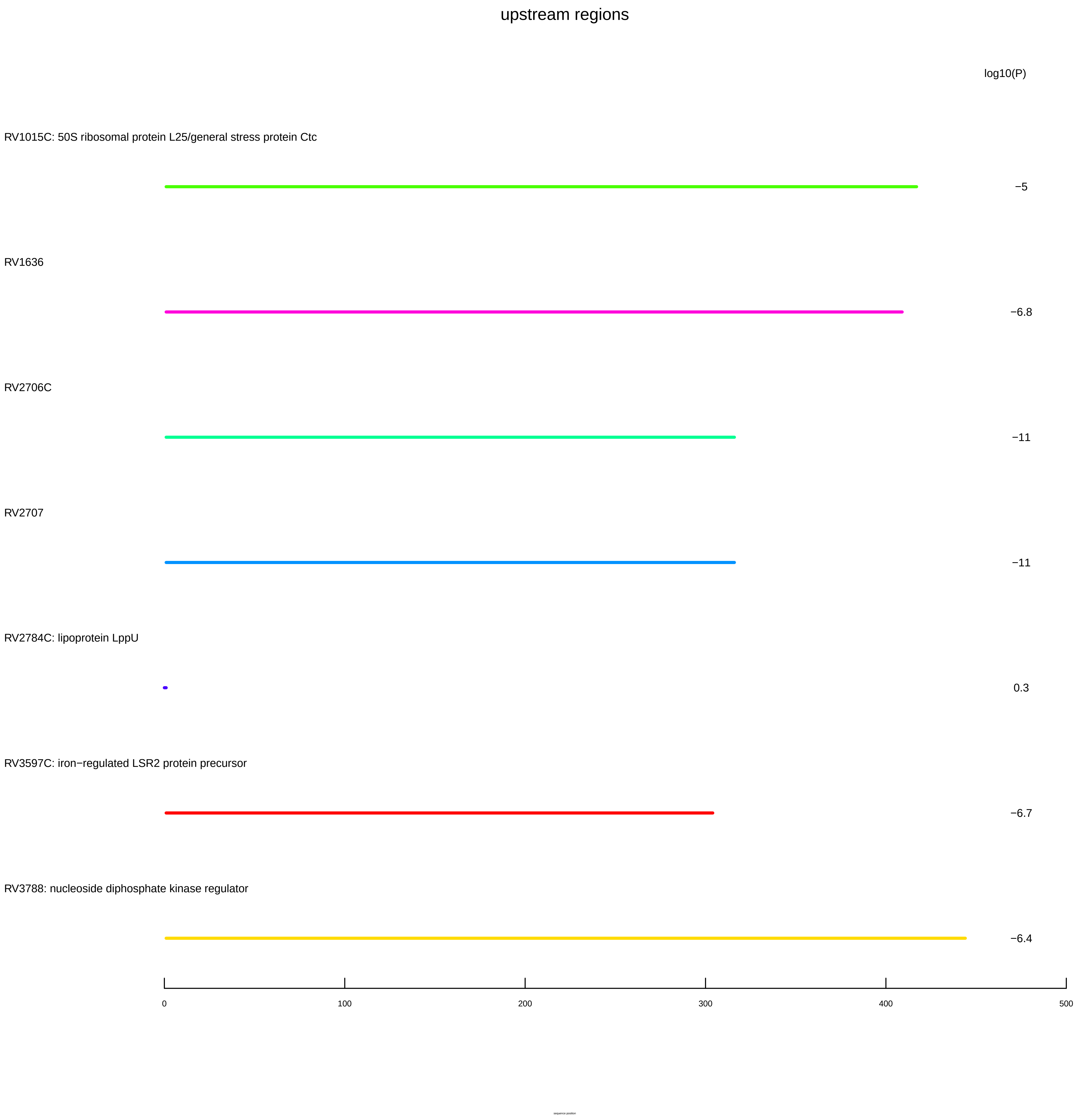

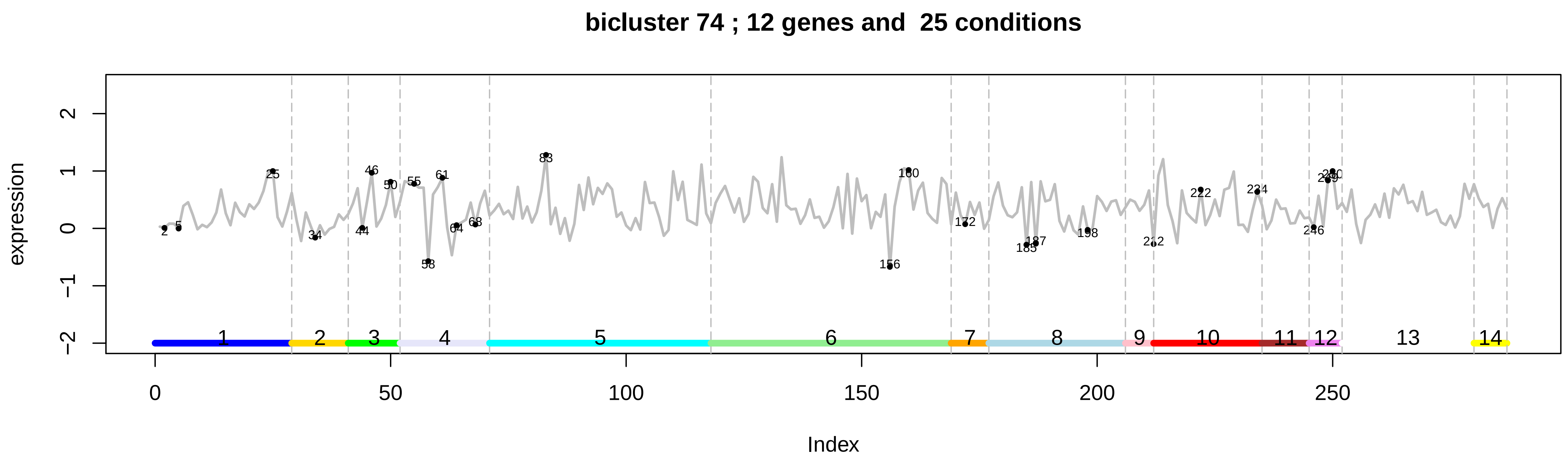
bicluster 74 ; 12 genes and 25 conditions
expression
Index
1
2
3
4
5
6
7
8
9
10
11
12
13
14

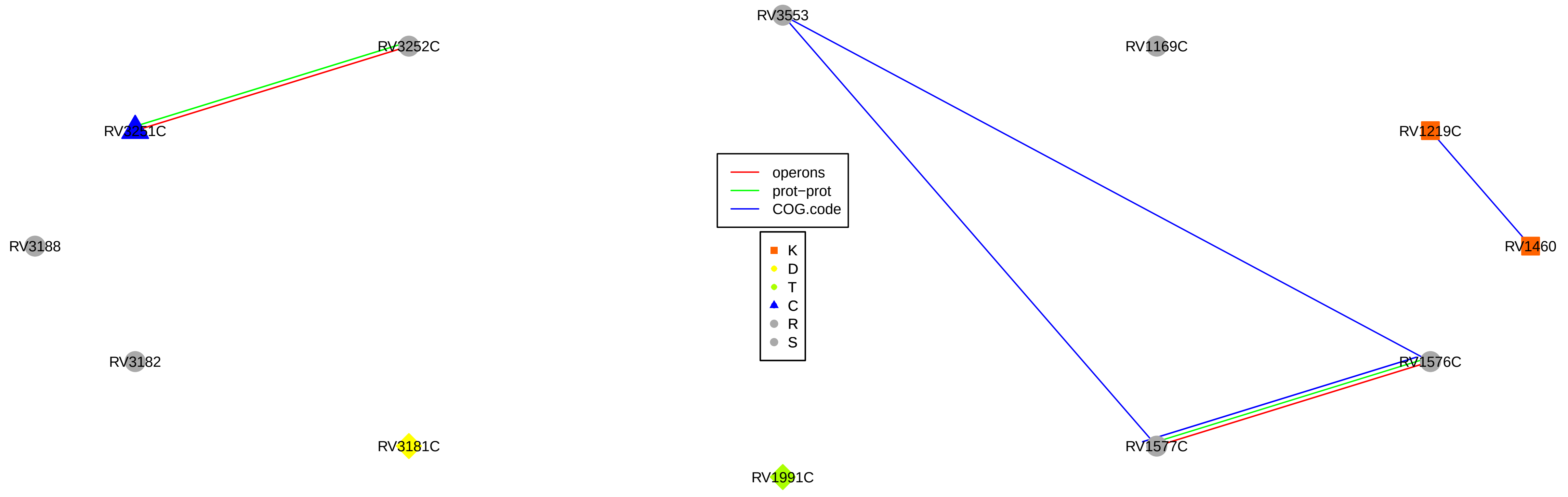
RV3553
RV3252C
RV1169C
RV3251C
RV1219C
RV3188
RV1460
RV3182
RV1576C
RV3181C
RV1577C
RV1991C
operons
prot−prot
COG.code
K
D
T
C
R
S

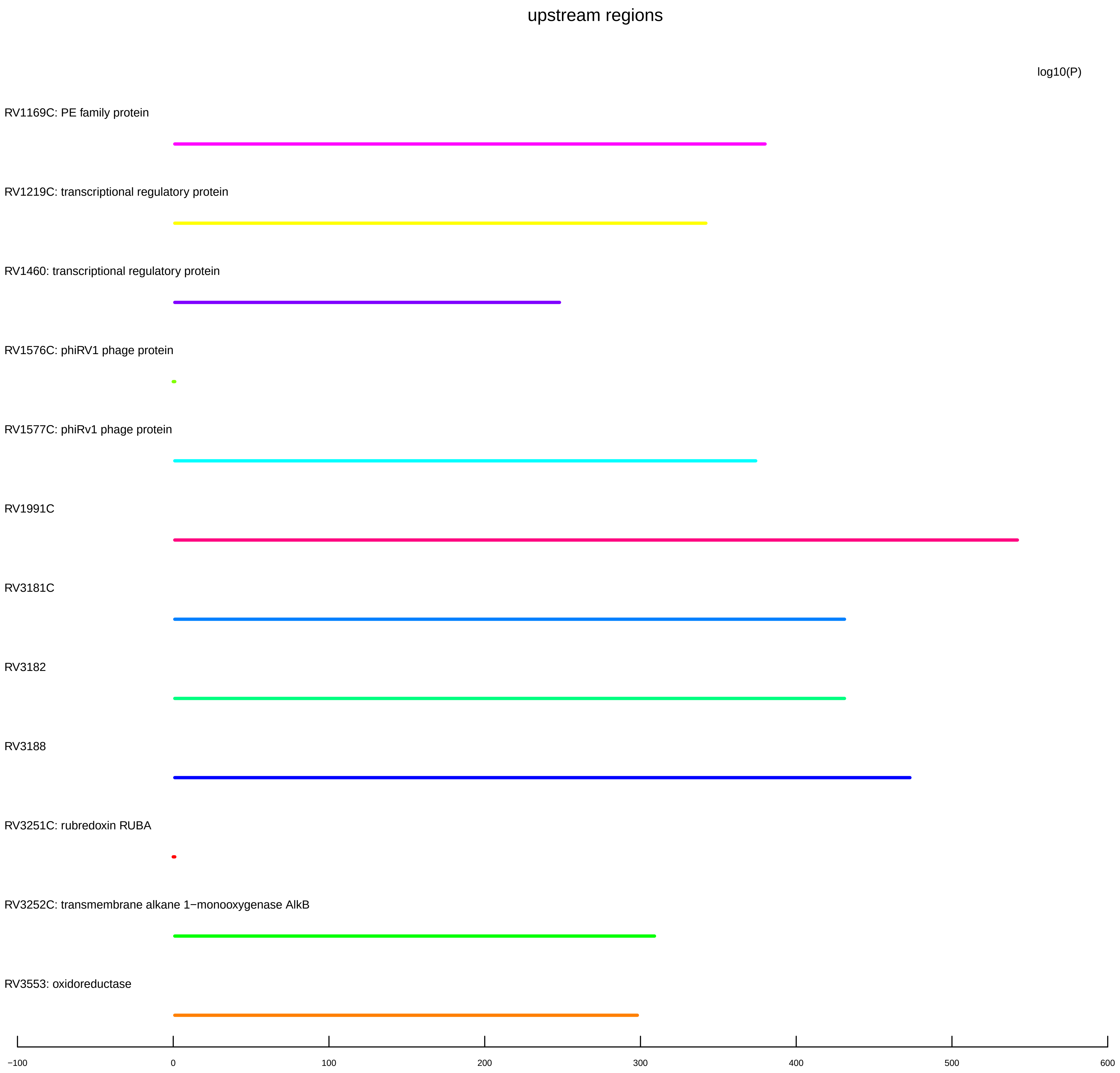
upstream regions
log10(P)
RV1169C: PE family protein
RV1219C: transcriptional regulatory protein
RV1460: transcriptional regulatory protein
RV1576C: phiRV1 phage protein
RV1577C: phiRv1 phage protein
RV1991C
RV3181C
RV3182
RV3188
RV3251C: rubredoxin RUBA
RV3252C: transmembrane alkane 1−monooxygenase AlkB
RV3553: oxidoreductase
−100
0
100
200
300
400
500
600

bicluster 75 ; 19 genes and 45 conditions

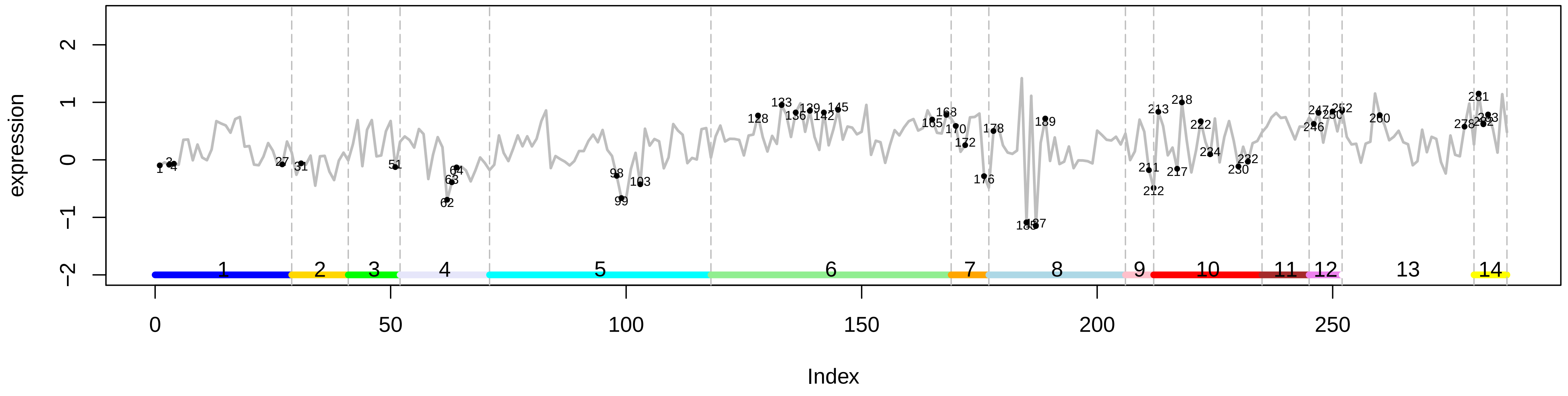

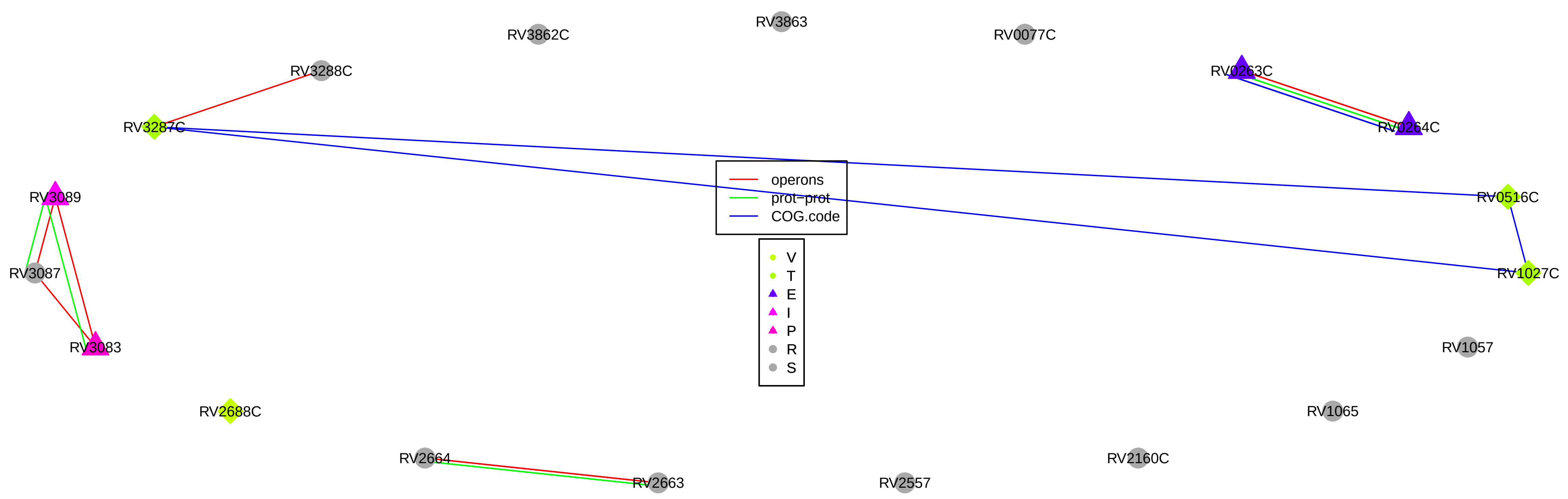

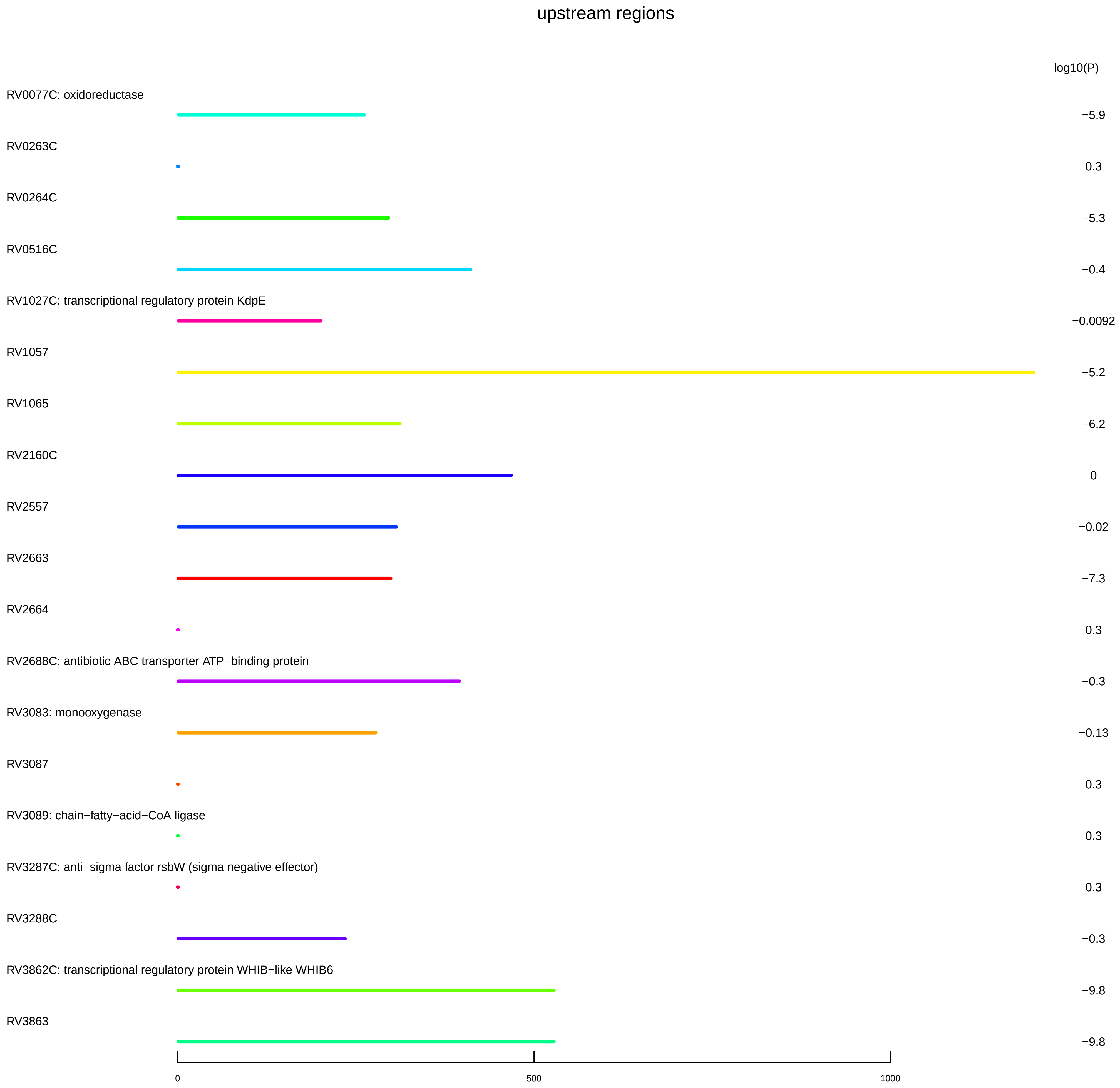

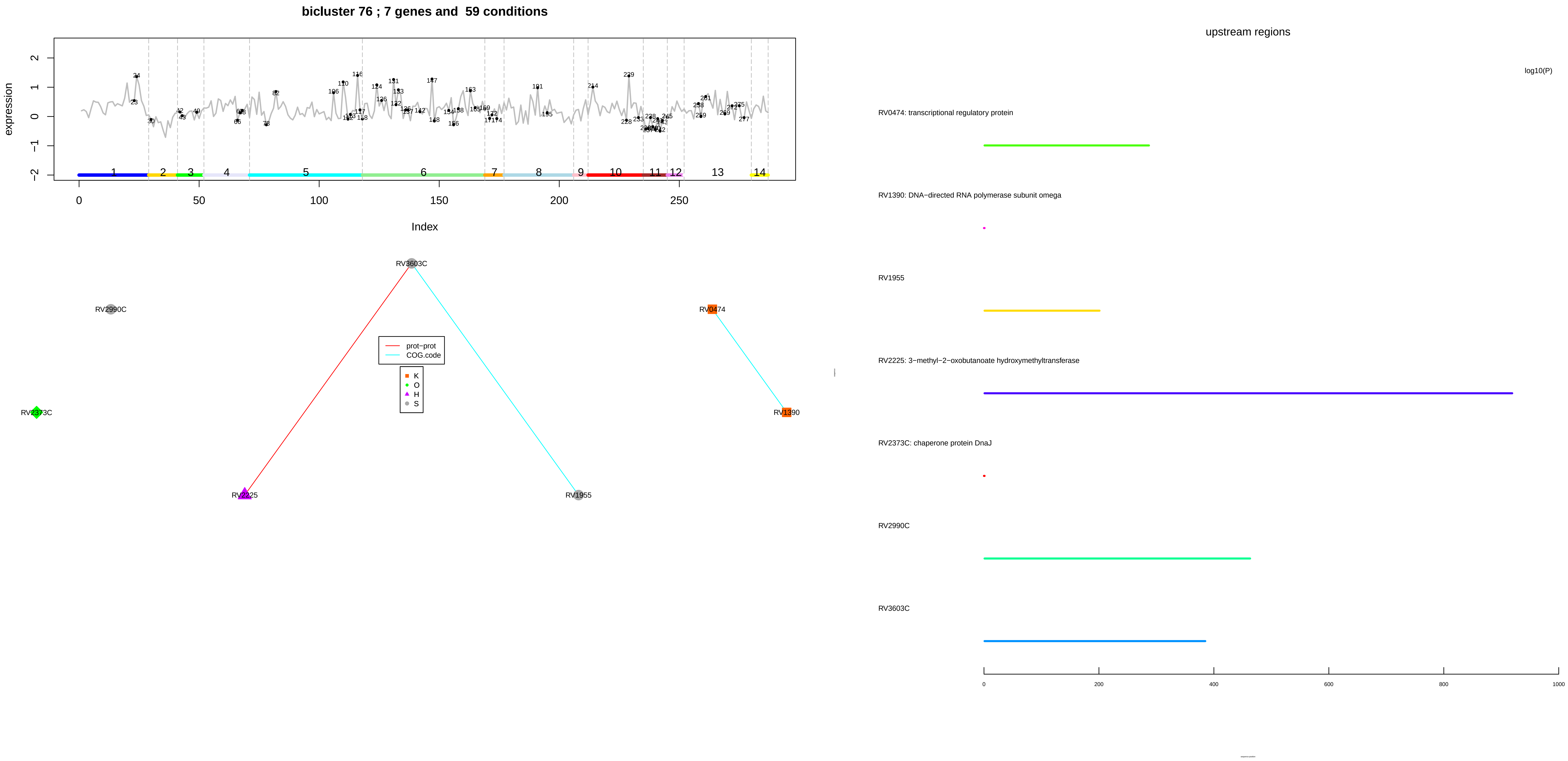

bicluster 76 ; 7 genes and 59 conditions
expression
Index
1
2
3
4
5
6
7
8
9
10
11
12
13
14
RV3603C
RV2990C
RV0474
RV2373C
RV1390
RV2225
RV1955
prot−prot
COG.code
K
O
H
S
upstream regions
log10(P)
RV0474: transcriptional regulatory protein
RV1390: DNA−directed RNA polymerase subunit omega
RV1955
RV2225: 3−methyl−2−oxobutanoate hydroxymethyltransferase
RV2373C: chaperone protein DnaJ
RV2990C
RV3603C
