# Supplementary material for: Integration of heterogeneous molecular networks to unravel gene-regulation in Mycobacterium tuberculosis
Source: BMC Syst Biol. 2014 Sep 26;8:111. doi: 10.1186/s12918-014-0111-5 (PMC4181829; doi:10.1186/s12918-014-0111-5)
Supplement: Additional file 4: — Technical characteristics of the visualization tool. Technical description of the network visualization and comparison tool used. [file 12918_2014_111_MOESM4_ESM.pdf]

Data Integration, Visualization and Analysis (DIVA) tool

DIVA is a standalone, customizable and web based tool that can be integrated into other web based tools.

The networks are loaded from custom generated XGMML files. Each network view can be independently zoomed in or out moved and the size of the nodes can be separately modified independently of the zoom level. A unique id linked to the locus tag is used to bind the same node in all the different networks and is stored as an attribute of the node. Optional names for the nodes (aliases) are also stored as node attributes. The metabolic network is imported from Pathway Tools (see Materials and Methods). Initially, the lines representing reactions with known associated genes are coloured in black whereas the rest are coloured in green. On a mouseover event information about the name and MetaCyc (Caspi, R *et al*. 2011. *Nucleic Acids Research* 40) identifier of each compound is presented. When mapping expression data, the reactions will be coloured according the highest expression value of the related genes. The use of a unique id in all the networks, allows to share selections between the networks, so that when a node is selected in a network, all the nodes in the other networks sharing the same id will be selected. Furthermore, the user can configure the layout of the application and select the networks that are loaded, see Figure 1. A multitude of networks can be presented simultaneously and the user can decide among the different schemes and networks what the best methods to use for the available data and the intended analysis are.

Selections of multiple nodes can be made either graphically, using the mouse or by entering a list of genes (ids or aliases) in text mode and they can be stored for later use.. For each selection the information about the selected genes such as locus tag, aliases and annotations is presented. Three modes allow to combine two selections using the logic operators *or*, *and*, or *xor*. An additional selection mechanism linked to expression data is available, so that only those nodes with expression values above or below a selected threshold are chosen. Selections can be stored and modified and comments can be stored for each selection. In addition, DIVA contains a tool that allows to compute the overlap between the stored selections and the and current selection. Analysis tools such as motif identification, GO enrichment analysis or expression profiling can be run on a selection, the outcome of the analysis is stored and can be linked to the selection’s comments.

The pipe line to create a draft metabolic map from the annotated genome of the *Mtb* H37Rv strain and integrate it into our visualization tool is show in Figure 2. We used Pathway tools (P. D. Karp et al. 2010) to create an initial map. Pathway tools allows to export the attributes for each metabolite and reaction into flat files and is able to generate prints of the pathway overview both in PDF and HTML. We created a set of Java programs, Python scripts and SPARQL queries to convert the pathway tools export files into SVG and RDF files The PDF file is converted to SVG; the attribute files are converted to RDF, so that they can be queried with SPARQL. One SPARQL query is used to create the resulting RDF file, which holds for each reaction information about the related genes, product and substrates. The HTML file is converted to XHTML, so that it can be parsed, and holds the information between the location of a reaction in the SVG file and its identifier in the resulting RDF file.

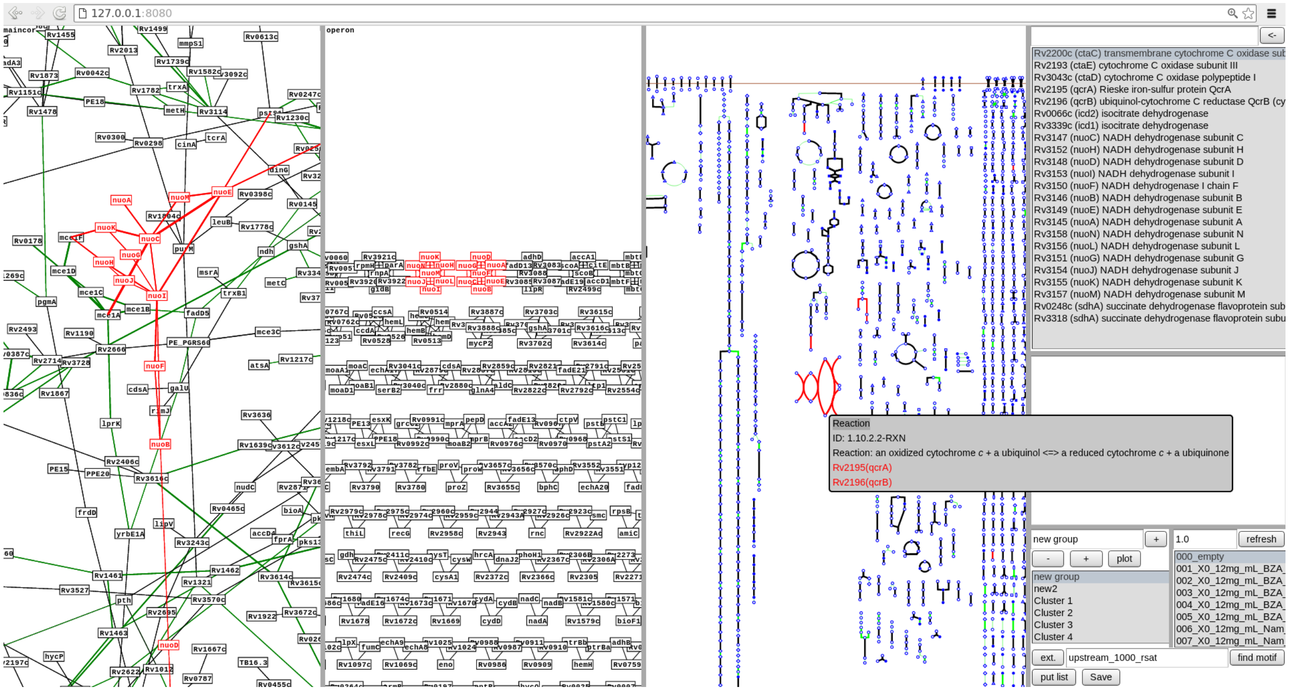

Figure 1: Screenshot of DIVA. Left: co-expression network (obtained from the combination of R^λ and C^λ with $\lambda = \sqrt{2}$); Middle Left: network of operons; Middle Right: metabolic network with a pop-up with information about the reaction linked to the selected genes; Right: control panel, Right top: currently selected genes, marked by red squares (co-expression and operon network) or red lines (metabolic network) and their annotation. Right bottom: on the left list of previously stored gene selections ; on the right a list of previously stored colouring schemes.

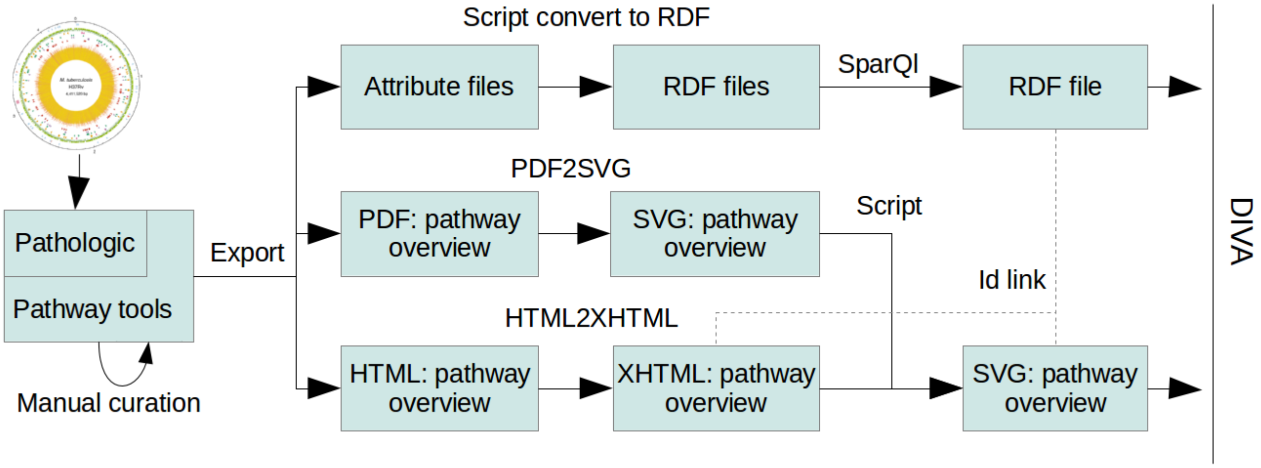

Figure 2: The attribute files, the PDF and HTML are exported from pathway tools. The PDF is converted into a SVG file, which will be displayed in DIVA. To link each element in the SVG to the information in the attribute files, the HTML file is used after it is converted to XHTML. The attribute files contain all the compound and reaction information. These are converted to a RDF file, which can be directly read by our visualization tool.
