# Supplementary figures and images for: Integration of heterogeneous molecular networks to unravel gene-regulation in Mycobacterium tuberculosis
Source: BMC Syst Biol. 2014 Sep 26;8:111. doi: 10.1186/s12918-014-0111-5 (PMC4181829; doi:10.1186/s12918-014-0111-5)

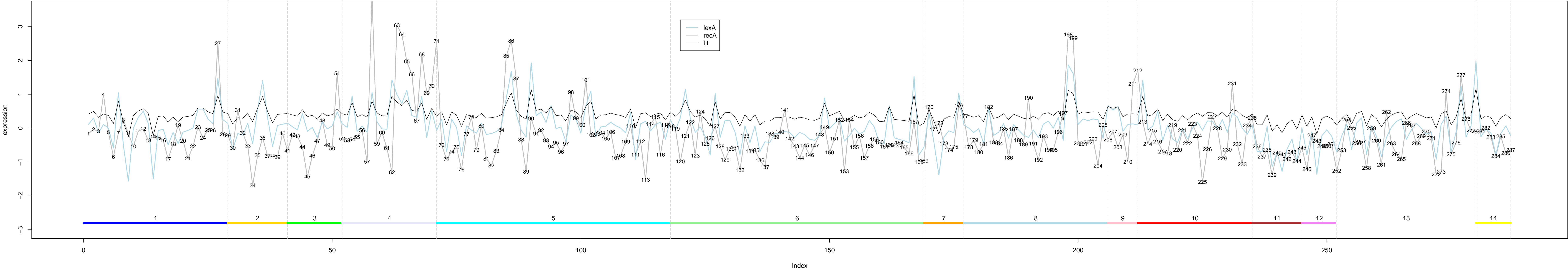

Supplement: Additional file 5: — Relationship between the expression levels of recA and lexA. The fit corresponds to a model, based on ordinary differential equations, of LexA mediated induction of recA. The numbers signal the conditions where this model actually holds. For clarity, expression values have been scaled, so that the mean value for each gene, when all conditions are considered, is zero. The horizontal bar includes the overall categorization of the conditions (see Materials and Methods). Notice that the conditions were LexA is regulating recA expression are mainly linked to 1: aromatic amides intra cellularly hydrolysed, low pH; 4: Acidified medium; 5: Cell wall synthesis inhibition; and 8: DNA damage. [file 12918_2014_111_MOESM5_ESM.pdf]

A

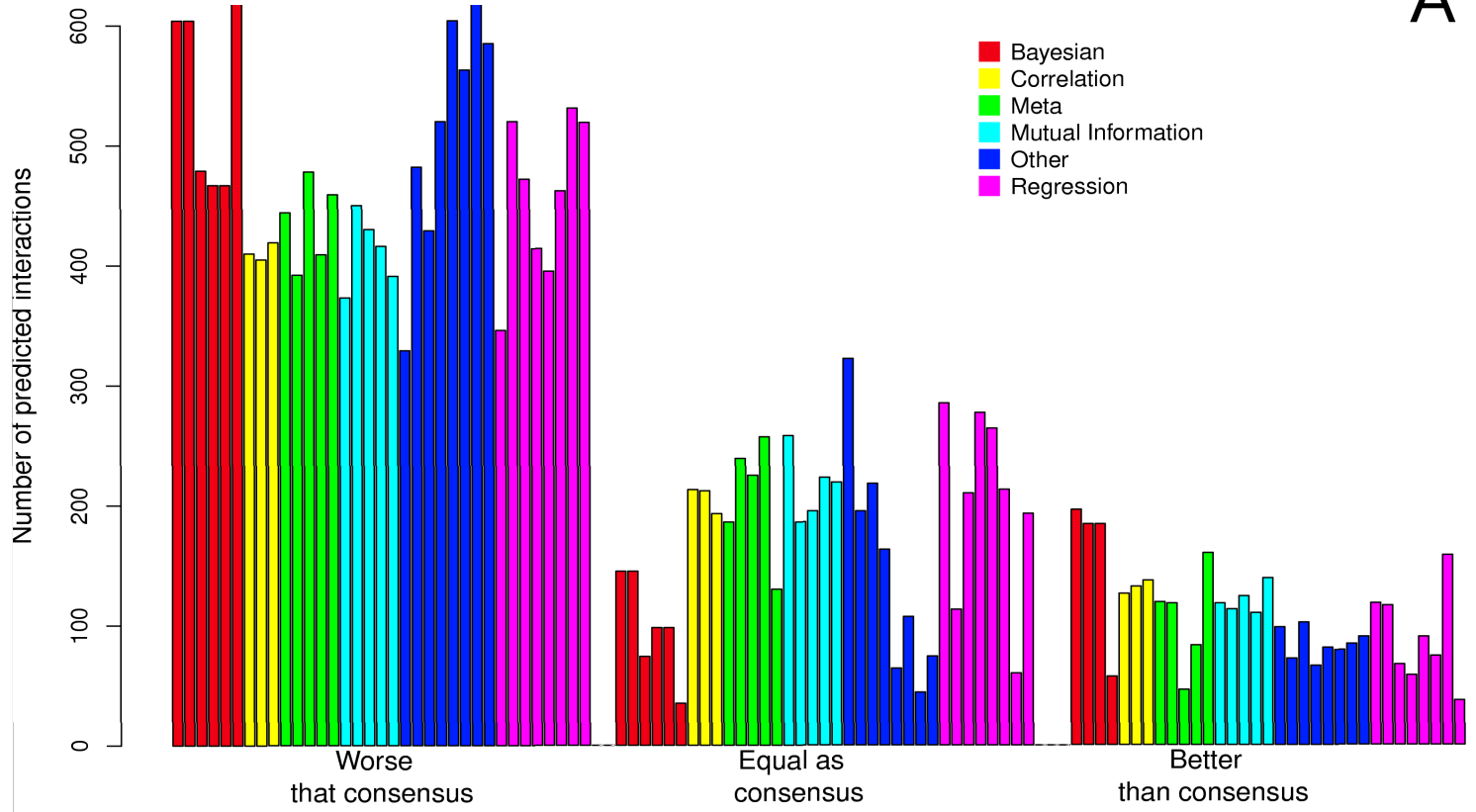

B

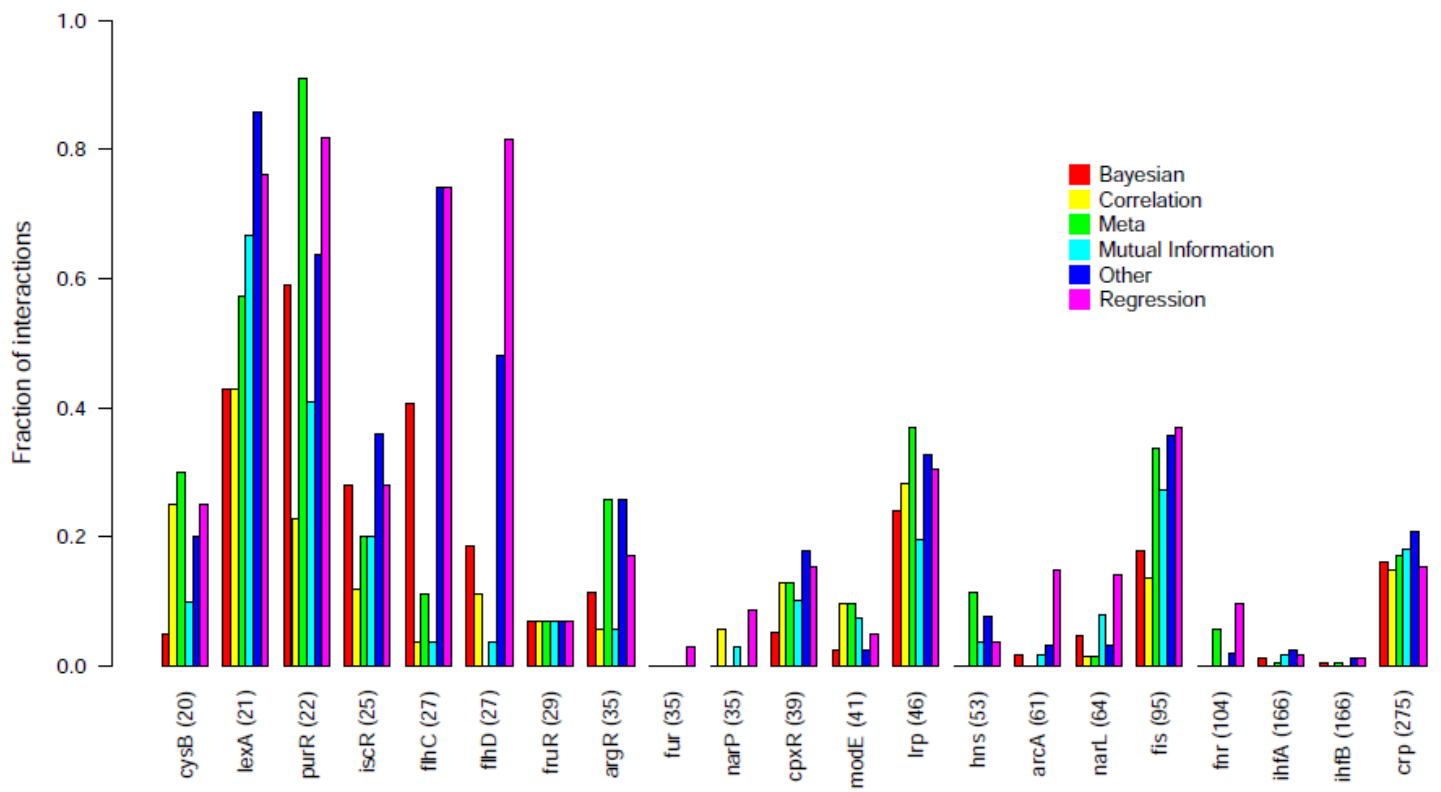

Supplement: Additional file 6: — Comparison of predictions for individual methods to consensus network. A) Overall predictions. The plot shows the number of interactions from the gold standard that were predicted worse, equally or better by each individual method than by the consensus network [15]. The predictions were assessed comparing the rank of the predictions with a 10.000 cutoff. Only the E. coli network has been considered. B) Predictions for each TF. Fraction of the interactions that are better predicted by each type of method than by the consensus network. Only TF with more than 20 interactions have been considered. These plots have been built using the methods described in [15]. [file 12918_2014_111_MOESM6_ESM.pdf]

# E. coli ZRC network

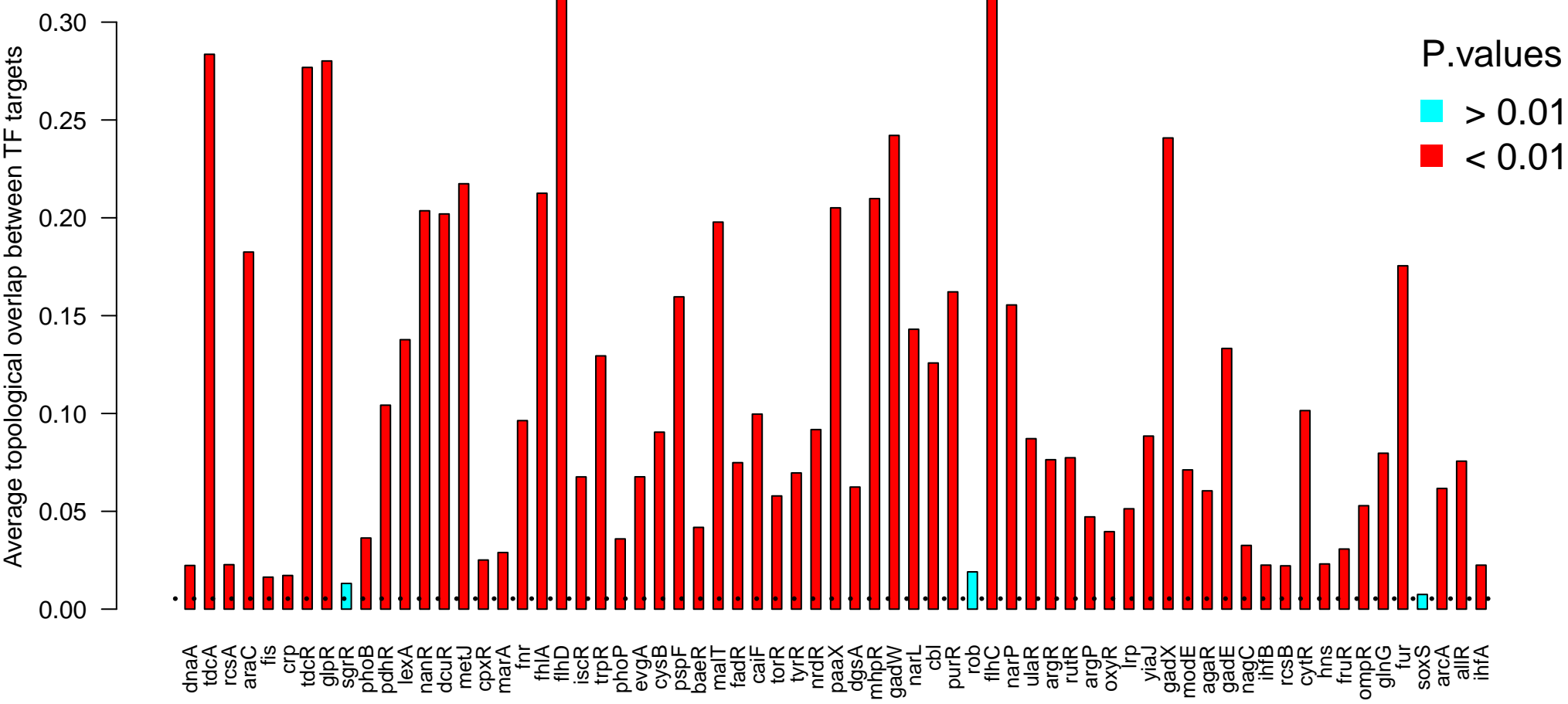

# E. coli Mixed network

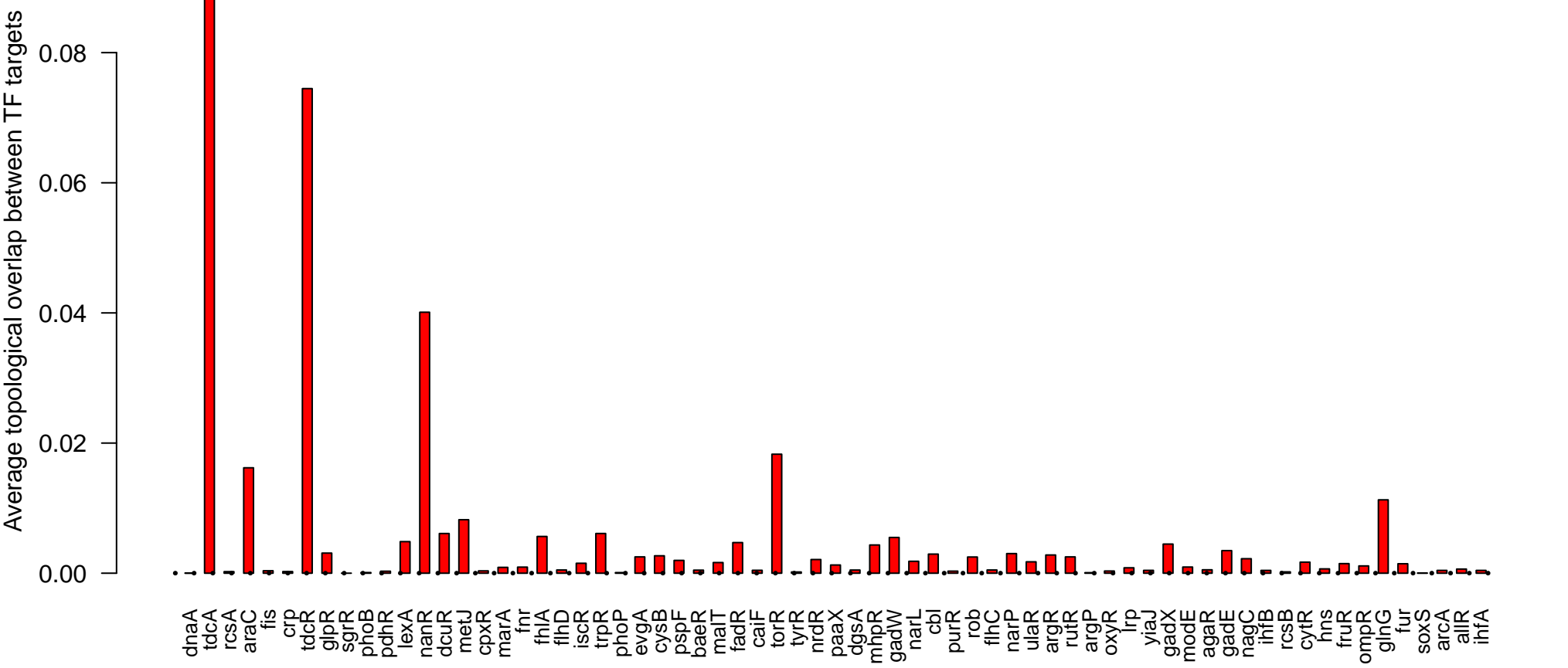

# S. cerevisiae ZRC network

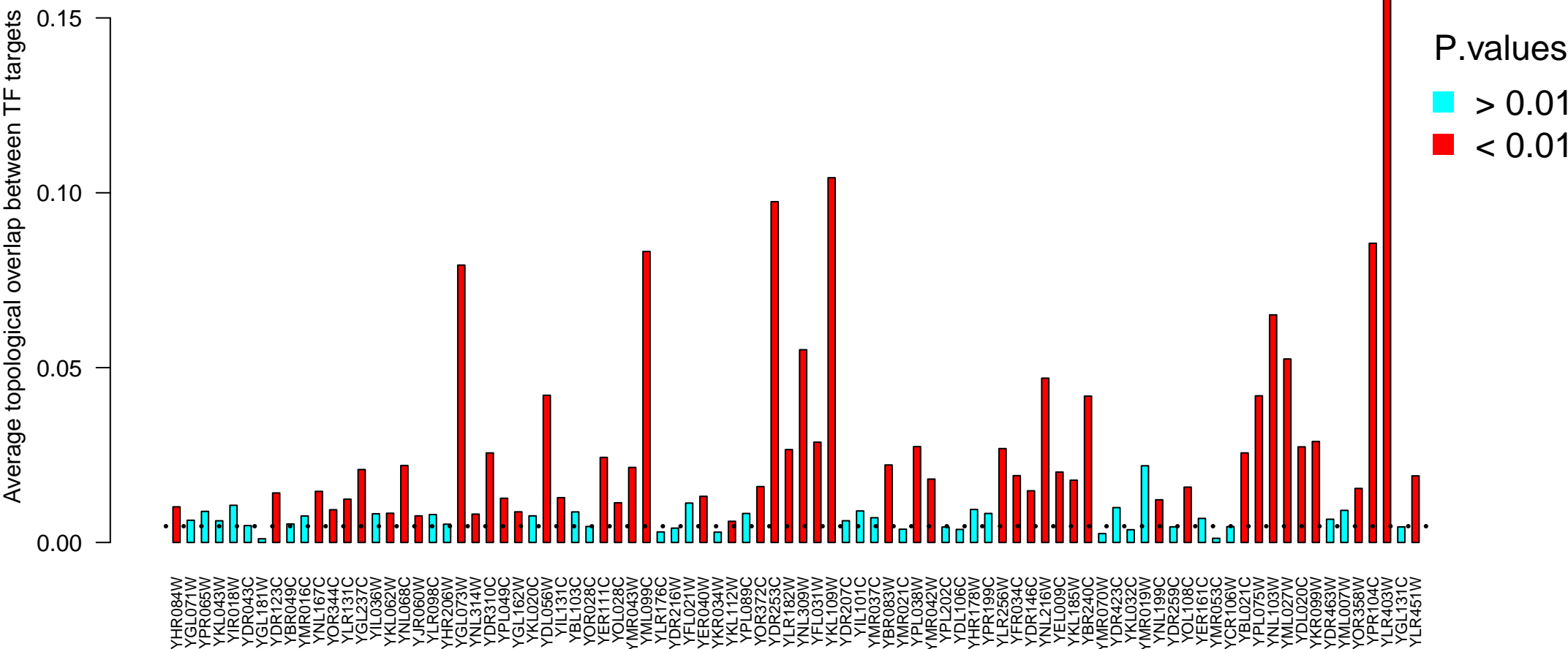

# S. cerevisiae Mixed network

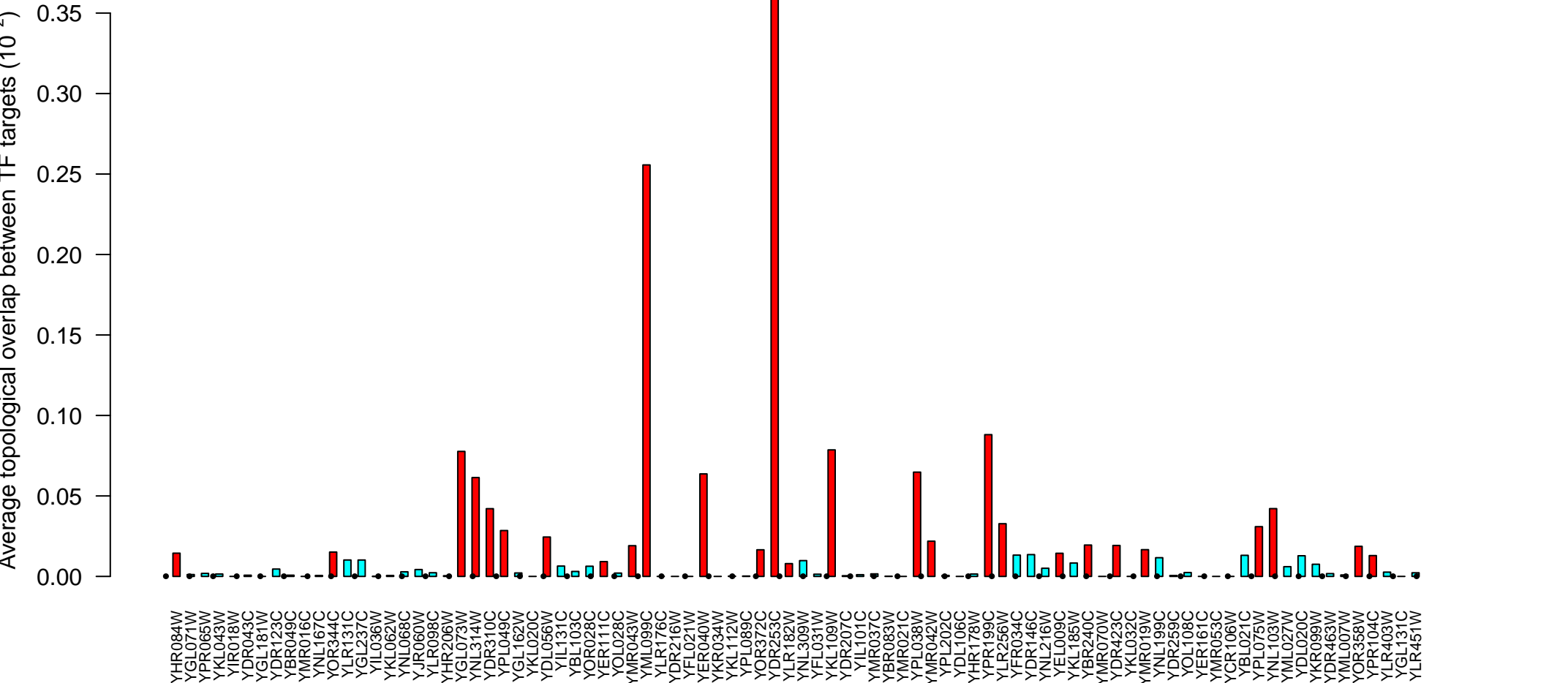

Supplement: Additional file 7: — Topological overlap of TF targets in the co-expression networks obtain for the DREAM5 challenge datasets. For each dataset (synthetic, Escherichia coli and Saccharomyces cerevisiae) the ZRC and mixed networks were built. Only TF with more than 5 experimentally verified targets (in the gold standards) were considered. Dashed line represents the average topological overlap in each network. [file 12918_2014_111_MOESM7_ESM.pdf]

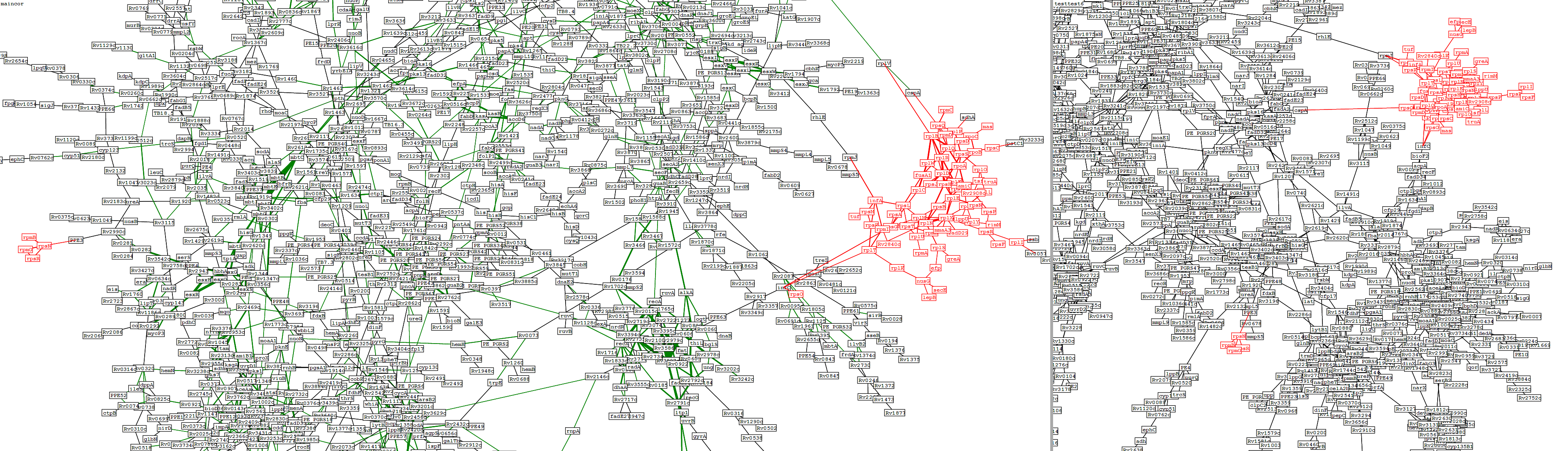

Supplement: Additional file 8: — Relative locations of the cluster of ribosomal proteins and the operon of ribosomal proteins rpmB2-rpmG1-rpsN2-rpsR2 in different co-expression networks. [file 12918_2014_111_MOESM8_ESM.png]

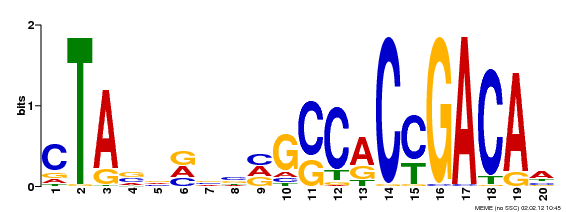

Supplement: Additional file 11: — Identified motif in the upstream regions of the genes in the RecA_ND DNA repair system. Logo generated using MEME [54]. E-value 6.0 E-60. The location of the hits is included in Additional file 10. [file 12918_2014_111_MOESM11_ESM.png]

number of conditions: 27

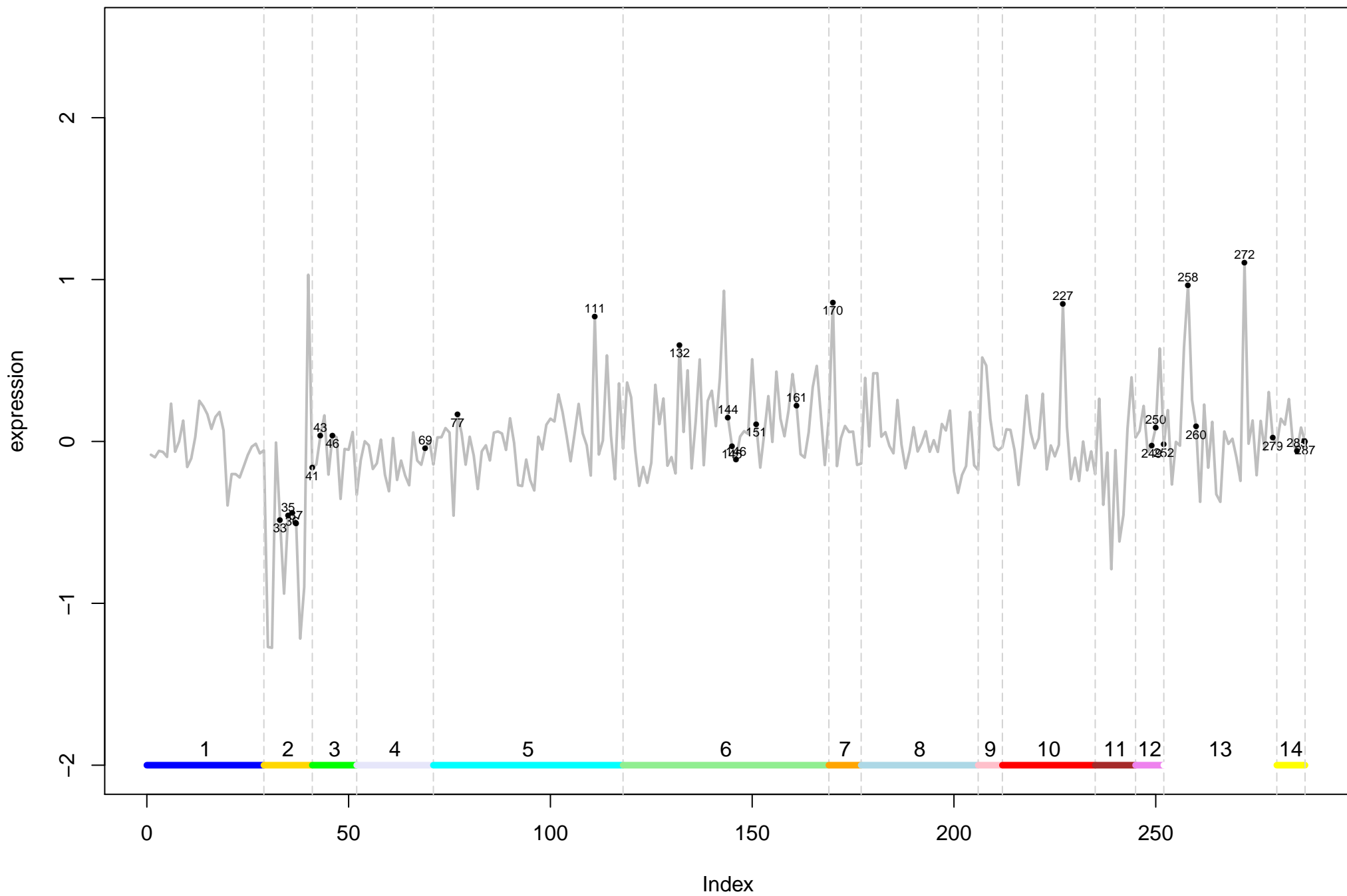

Supplement: Additional file 14: — Bicluster formed by members of DevR regulon and the type VII secretion system (Esx-3) genes Rv282-Rv290. The grey line represents the average expression levels of these genes in the conditions in our compendium. The numbers identify the 27 conditions that have been included in the bicluster (Additional file 1). [file 12918_2014_111_MOESM14_ESM.pdf]

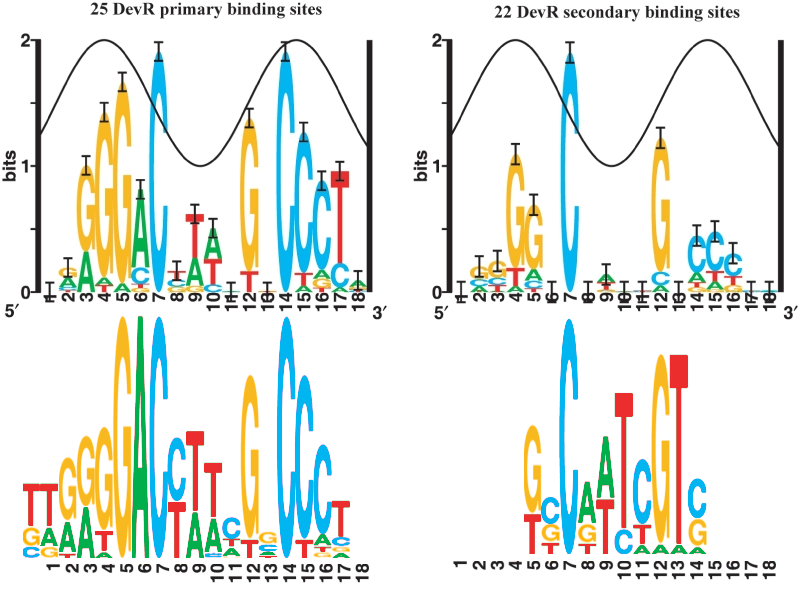

Supplement: Additional file 15: — DevR Motifs Top: Previously identified primary and secondary motifs for DevR [ 84 ] Bottom: Newly proposed motifs. Main differences in primary motif can be seen at positions 6, 8, 1 and 2. Main differences in secondary motif can be seen at position 13 and 8 to 11. [file 12918_2014_111_MOESM15_ESM.png]
